# Synthesis of 2‑Fluoroalkylated Pyridines and Fluoropyridines by Thermal Denitrogenation of *N*‑Fluoroalkyl-1,2,3-triazoles and Cyclization of Ketenimines

**DOI:** 10.1021/acs.joc.5c03046

**Published:** 2026-01-22

**Authors:** Svatava Voltrová, Blanka Klepetářová, Petr Beier

**Affiliations:** Institute of Organic Chemistry and Biochemistry, 89220 Czech Academy of Sciences, Flemingovo nám. 2, 160 00 Prague, Czechia

## Abstract

2-Fluoro-6-fluoroalkylpyridines and their ring-fused analogues were synthesized from *N*-fluoroalkyl-4-alkenyl-1,2,3-triazoles by thermal denitrogenation, fluorine shift, cyclization, and hydrogen fluoride elimination. This reaction proceeds via ketenimine intermediates. Conversely, 2-fluoroalkylpyridines were prepared starting from *N*-fluoroalkyl-4-alkyl-1,2,3-triazoles and proceeded by denitrogenation, fluorine shift, hydride shift, cyclization, and hydrogen fluoride elimination. Nucleophilic aromatic substitution of 2-fluoro-6-fluoroalkylpyridines afforded highly functionalized 2-fluoroalkylpyridines.

## Graphical abstract

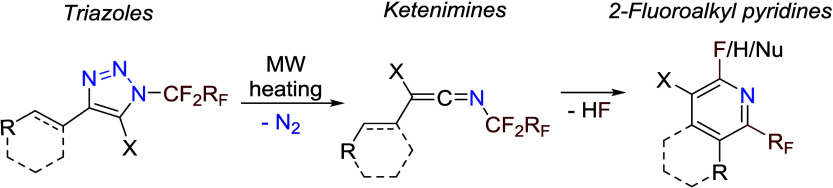

## Introduction

2-Trifluoromethylated pyridines are key building blocks and intermediates in the production of pharmaceuticals, agrochemicals, and specialty chemicals.^1^ Numerous insecticides, herbicides, and fungicides featuring these structural elements are in use (Figure 1). The synthesis of 2-trifluoromethylated pyridines is traditionally based on deoxofluorination of pyridinecarboxylic acids with SF_4_/hydrogen fluoride (HF),^2^ hetero-Diels–Alder reaction of butadiene with CF_3_CN in flow,^3^ Cu­(II)-catalyzed cyclization of ynones with vinyl azides,^4^ cyclization of chalcones with 1-(3,3,3-trifluoro-2-oxopropyl)­pyridine-1-ium,^5^ and more recently on cross-coupling of halogenated pyridines with CuCF_3_ species.^6–10^ A recent report describes C–H trifluoromethylation in position 2 of *N*-methylpyridinium salts with trifluoroacetic acid mediated by silver salts.^11^ Despite these advances, novel synthetic approaches to selectively fluoroalkylated pyridines and fluoropyridines are in demand.

**Figure 1:**
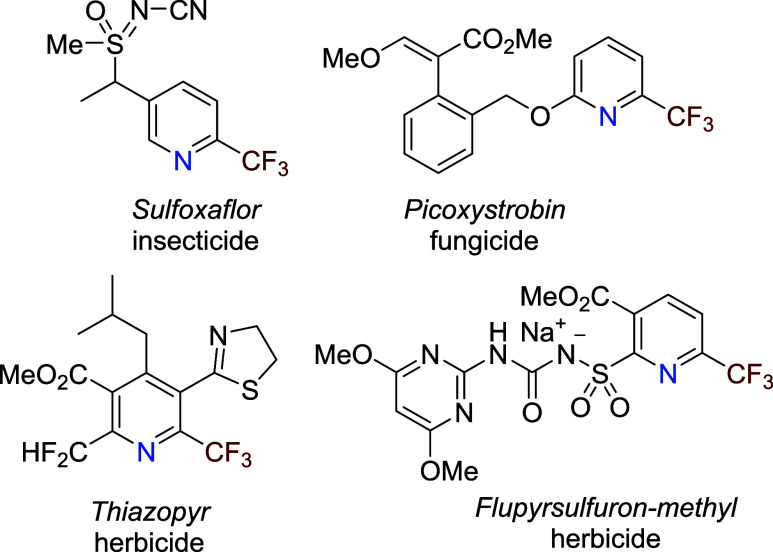
Examples of 2-trifluoromethylated pyridines in agrochemistry.

We have recently described a thermal denitrogenation of *N*-fluoroalkylated 1,2,3-triazoles **1**, derived from azido­(per)­fluoroalkanes, to ketenimines **2**, which in the presence of KF underwent a fluorine shift, followed by S_E_Ar cyclization to 1-fluoroalkyl-3-fluoroisoquinolines **5** (Scheme 1A).^12,13^ We envisaged a related process of utilization of triazole-derived ketenimines,^14^ starting from alkenyl triazoles **6**, where the nitrilium intermediate **9** would react with the sp2 carbon of the alkenyl moiety in a 6-endo-dig cyclization to afford 2-fluoro-6-fluoroalkylpyridines **11** (Scheme 1B). Conversely, alkyl triazoles **12**, which after rearrangement, fluorine shift, HF elimination, and hydride shift might form azatriene **16**. After cyclization and another HF elimination, the final products would be 2-fluoroalkylpyridines **18** (Scheme 1C). Both processes, if feasible, would represent new synthetic methodologies to selectively substitute 2-fluoroalkylpyridines and their ring-fused analogues.

**Scheme 1:**
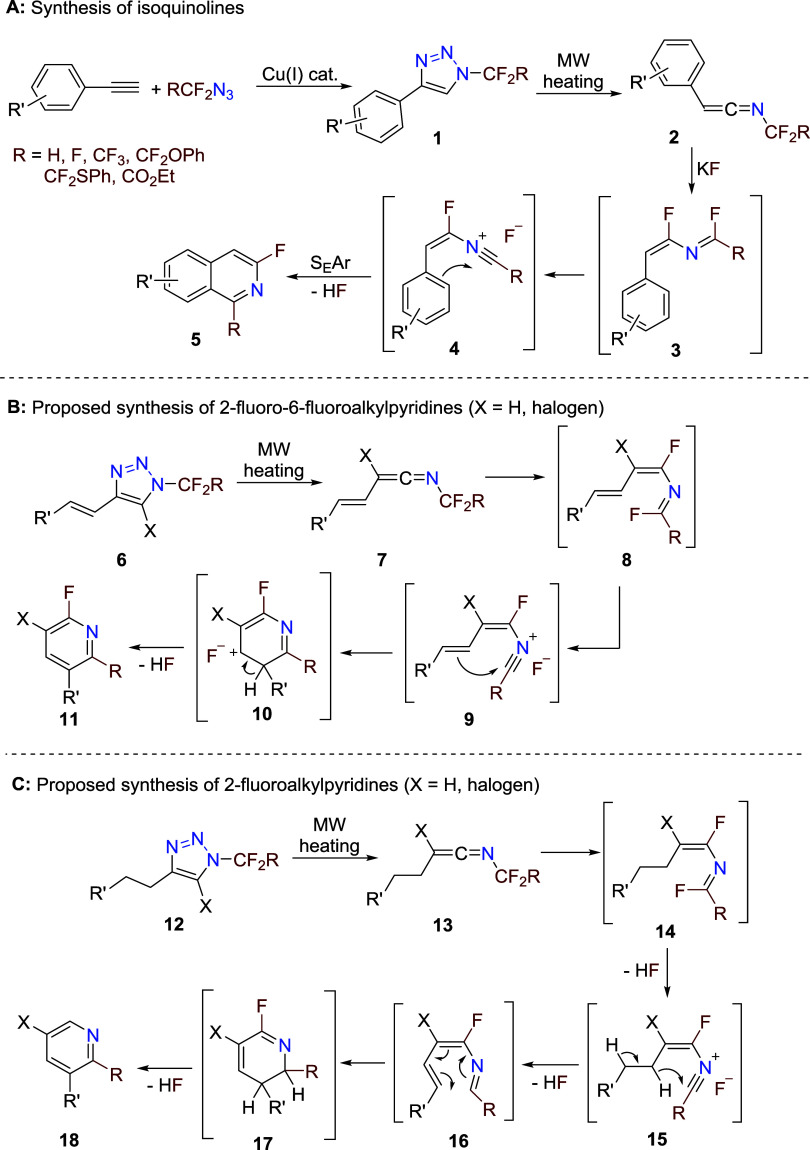
Triazoles and Ketenimines as Key Intermediates in the Synthesis of Isoquinolines **5** and 2-Fluoroalkylated Pyridines **11** and **18**

## Results and Discussion

Starting triazoles **6** and **12** were prepared in good to high yields by azide–alkyne cycloaddition catalyzed with Cu­(I) salts (see the Supporting Information). Optimization of the formation of **11a** was conducted using model triazole **6a** (Table 1). The optimization was performed in 1,2-dichloroethane (DCE) solution or neat with or without additives.

**Table 1:**
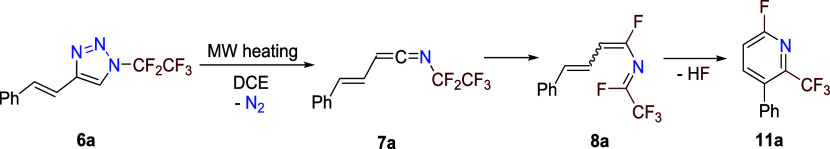
Optimization of the Formation of Fluoropyridine **11a** from Triazole **6a** ^a^

				yield^b^ (%)		
entry	Temp. (°C)	time (min)	additive (1 equiv)	**7a**	**8a**	**11a**
1	140	30		64	22	4
2	160	30		7	2	4
3	140	30	K_2_CO_3_	7	7	59
4	140	30	CsF	0	0	^c^
5	140	30	KF	11	26	62
6	140	60	KF	0	2	83
7^d^	140	1	KF	0	18	57
8^d^	140	1		0	25	34

a Scale 0.25 mmol, DCE (1.5 mL).

b ^19^F NMR yield using PhCF_3_ as an internal standard.

c Product formed but decomposed.

d No solvent.

The triazole denitrogenation to ketenimine required microwave (MW) heating to at least 140 °C. Without an additive, the major product was ketenimine **7a** (Entry 1), and heating to 160 °C only provided low product yields (Entry 2). A good product yield was obtained with the K_2_CO_3_ additive (Entry 3), while with CsF, the product **11a** formed but decomposed (Entry 4). The highest product yield was achieved using DCE solution with KF additive (Entry 6), which is believed to facilitate the fluorine shift to species **8** by fluoride addition to the sp-hybridized carbon triggering fluoride elimination from the CF_2_ group. Using solvent-free conditions proved to be suboptimal (Entries 7 and 8).

Structurally diverse triazoles **6** were subjected to the thermal process in the presence of KF in order to establish the scope of fluorinated pyridines **11** (Scheme 2). Good product yields were obtained with 4-cinnamyl-substituted 1-perfluoroethyl triazoles **6a**–**6e** and 1-tetrafluoroethyl triazoles **6f**–**6h**, while the 5-chloro analogue afforded the product **11i** in a much lower yield. This effect might be due to the decreased electron density of the double bond (chlorine atom in the allylic position) in intermediate **9** for cyclization. Ring-fused products **11j**–**11n** formed in good efficiency from 4-cyclohexenyl triazoles with various fluoroalkyl substitutions on the nitrogen atom. Starting from *N*-trifluoromethyl triazole **6o**, ring-fused *o*,*o*-difluoropyridine **11o** was obtained. Finally, *N*-difluoromethyl-4-cinnamyl-5-iodotriazole **6p** afforded after denitrogenation, cyclization, and HF elimination 2-fluoro-3-iodo-5-substituted pyridine **11p** in a high yield. On the other hand, certain scope limitations were found. For example, the 4-(2-methylcinnamyl) triazole **6q**, although some signals of denitrogenated intermediates were found, did not afford any traces of pyridine **11q**. Here, the methyl group on the double bond prevented the cyclization of planar intermediate **9**. All fluoropyridines **11** were liquids except for **11m**, whose X-ray structure was determined. This unique synthetic methodology leads to 2-fluoro-6-fluoroalkylpyridines, which are unprecedented in literature.

**Scheme 2:**
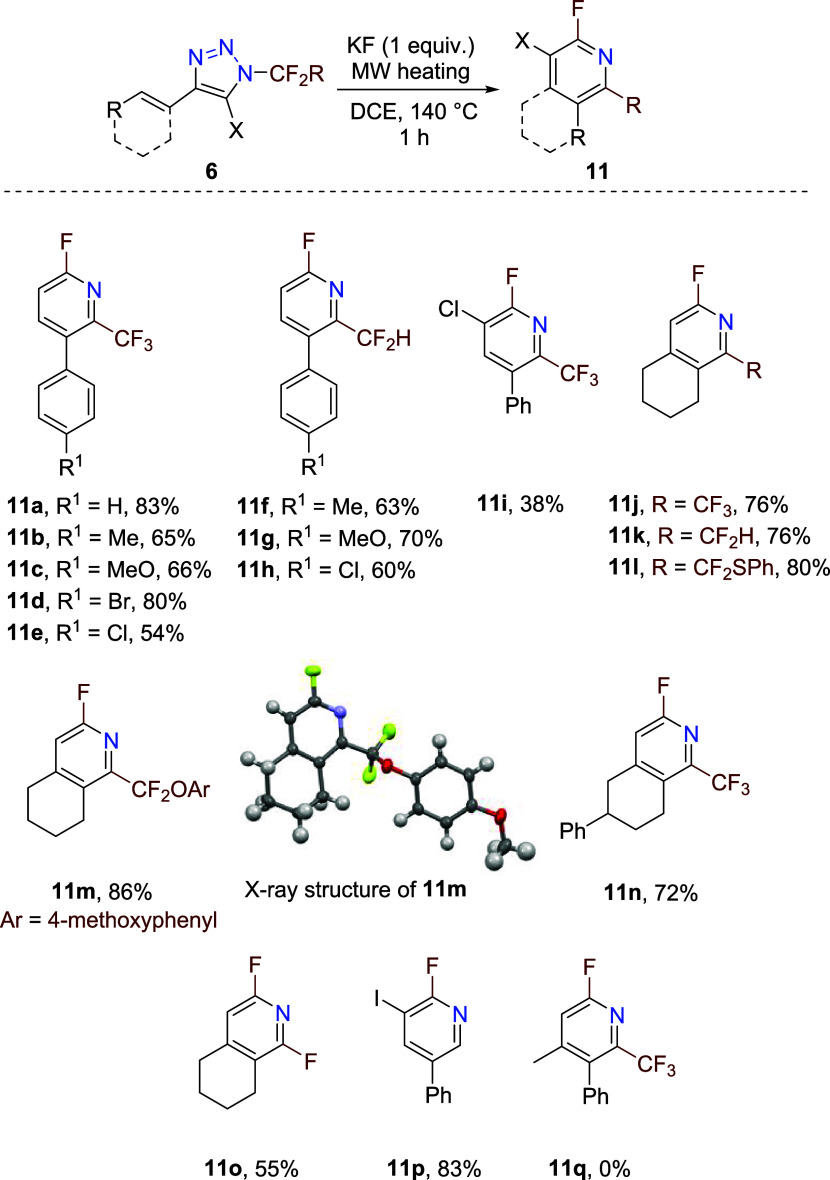
Synthesis of 2-Fluoro-6-fluoroalkylpyridines **11** from Triazoles **6**

Next, optimization of the formation of 2-fluoroalkylpyridines **18** from triazoles **12** was performed starting from triazole **12a** as a model substrate (Table 2). It was found that this process required heating to a higher temperature than in the case of pyridines **11** (165 °C was optimal), and solvent-free and additive-free conditions were preferred. The fluoride additive favored the formation of azadiene **14** but disfavored the formation of azatriene **16** (Scheme 1C). The optimized process consisted of heating neat triazole **12a** to 165 °C for 15 min (entry 8).

**Table 2:**
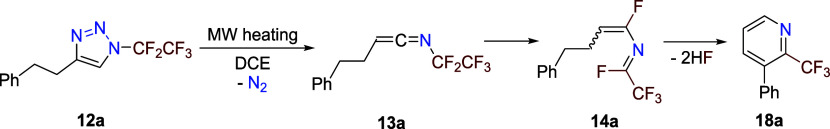
Optimization of the Formation of Fluoropyridine **18a** from Triazole **12a** ^a^

				yield^b^ (%)		
entry	Temp. (°C)	time (min)	additive (1 equiv)	**13a**	**14a**	**18a**
1	140	300		42	0	5
2	160	420		0	0	34
3	180	120		2	36	24
4	160	420	KF	0	0	20
5	180	120	CsF	0	17	10
6	180	120	K_2_CO_3_	7	2	14
7^c^	160	15		2	7	29
8^c^	165	15		3	12	56
9^c^	180	5		2	12	11
10^c^	165	15	KF	0	0	^d^

a Scale 0.25 mmol, DCE (1.5 mL) or neat.

b ^19^F NMR yield using PhCF_3_ as an internal standard.

c No solvent.

d Product formed but decomposed.

The proposed reaction mechanism (Scheme 1C) was partially supported by a series of mechanistic experiments that included the isolation of reactive intermediates. Microwave heating of **12a** in deuterochloroform led to the quantitative formation of ketenimine **13a**. When CsF was added, a fluorine shift occurred at room temperature (rt) to compound **14a**, which formed in a good yield and as a single stereoisomer (Scheme 3). After filtration, solvent removal under reduced pressure, and microwave heating under solvent-free conditions, compound **18a** formed in good yield. These experiments demonstrated that compounds **13a** and **14a** are intermediates on the reaction pathway to pyridine **18a**. We were not able to observe other proposed intermediates **16** and **17** by NMR spectroscopy.

**Scheme 3:**
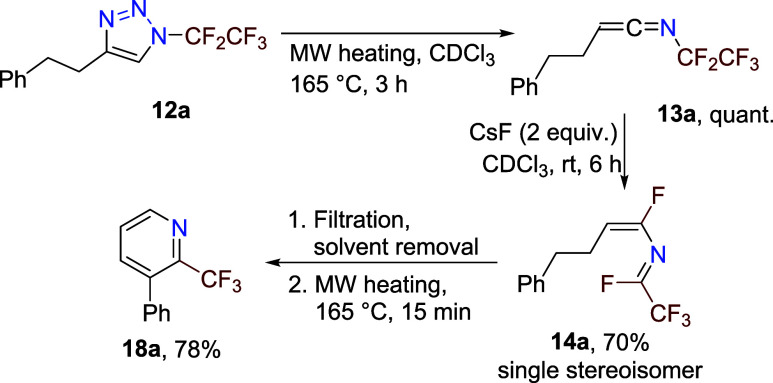
Stepwise Synthesis of 2-Trifluoromethyl-3-phenylpyridine **18a** from Triazole **12a**

The product scope of the synthesis of 2-fluoroalkylpyridines **18** from triazoles **12** was investigated (Scheme 4). Isolated yields of pyridines **18** were lower than in the pyridines **11** series, partly due to their volatility. 4-Alkyl-, 4-substituted alkyl-, and 4-cycloalkyl-triazoles **12** were subjected to microwave heating, and products **18** were obtained in low to good yields. The crystal structure of **18f** was determined. The reaction of 4-cycloalkyl-5-chlorotriazole **12g** afforded ring-fused 5-chloropyridine derivative **18g** in moderate yield. Iodoethylpyridine **18h** was detected in the crude reaction mixture after heating of triazole **12h**, but its isolation in pure form was unsuccessful.

**Scheme 4:**
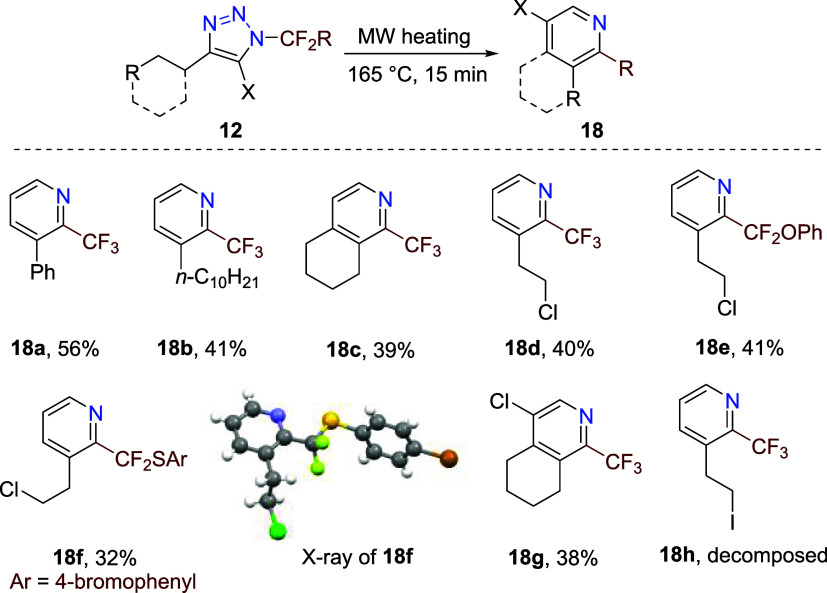
Synthesis of 2-Fluoroalkylpyridines **18** from Triazoles **12**

In order to demonstrate the synthetic utility of primary products, pyridines **11**, the S_N_Ar fluorine substitution reaction was investigated. Reactions with various oxygen-, nitrogen-, and sulfur-nucleophiles afforded substitution products **19**, generally in good to high yields (Scheme 5). Thus, hydroxypyridines **19a** and **19b** were prepared by microwave heating of fluoropyridines **11** with aqueous sodium hydroxide, ethoxy-substituted pyridines **19c** and **19d** were formed by room temperature reaction with EtONa, and phenoxy-substituted product **19e** was prepared by microwave heating with PhONa. Amination of fluoropyridines **11** was performed by microwave heating with primary or secondary amines, and the introduction of the methylthio group (products **19j**–**19p**) was achieved by room temperature reaction with MeSNa. 5-Chloropyridine derivative **18g** afforded under similar conditions the substitution product **19p** in a good yield. This work establishes new synthetic access to unprecedented functionalized 2-fluoroalkylpyridines **19**.

**Scheme 5:**
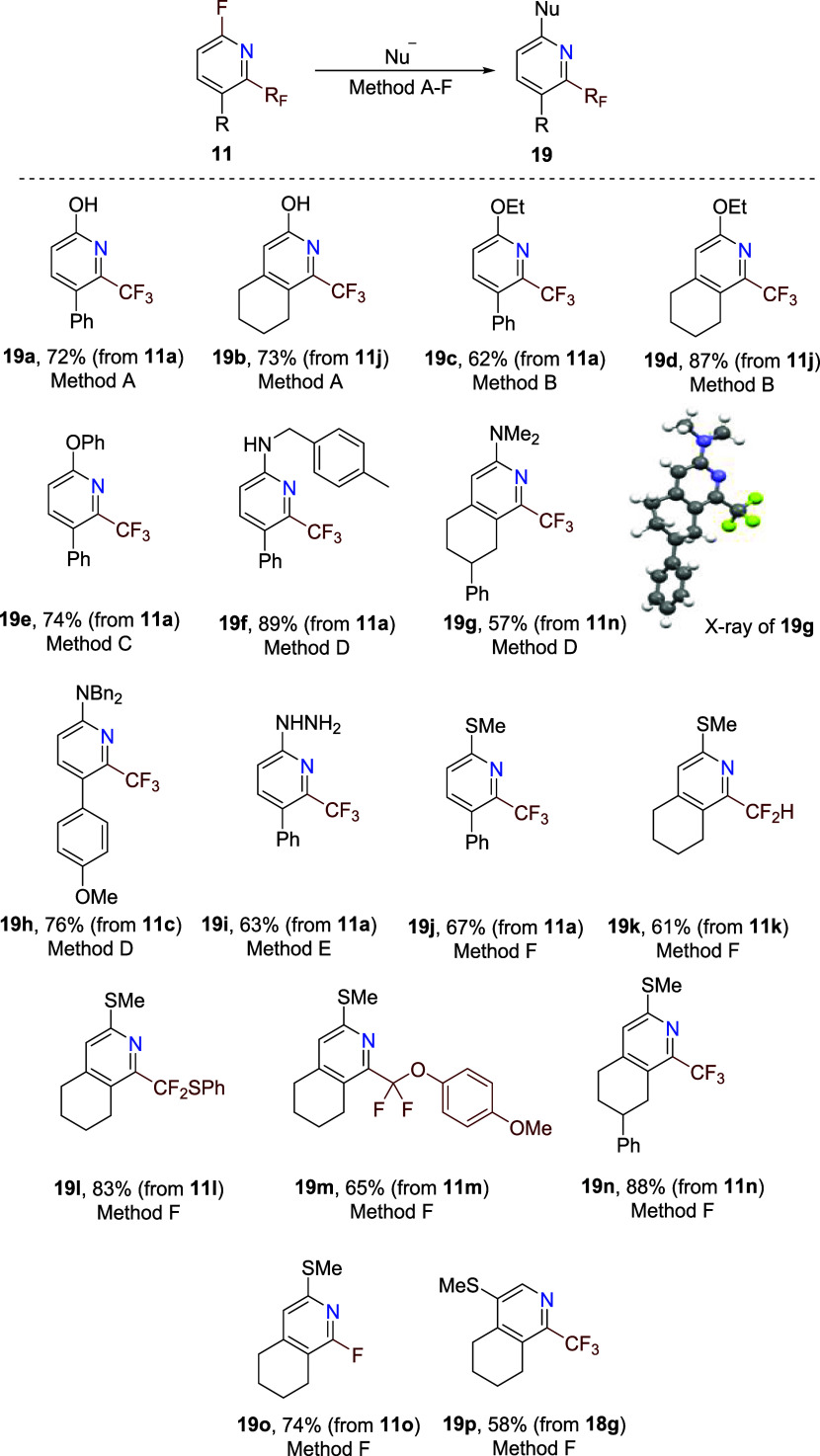
Functionalization of 2-Fluoro-6-fluoroalkylpyridines **11** by S_N_Ar^a^

a Reaction conditions: Method A: NaOH (4 equiv), H_2_O, MW heating 140 °C, 1 h; Method B: EtONa (2 equiv), EtOH, rt, 16 h; Method C: PhONa (3 equiv), *N*,*N*-dimethylacetamide (DMA), MW heating 140 °C, 1 h; Method D: Bn_2_NH or Me_2_NH (3 equiv), DMA, MW heating 140 °C, 1 h; Method E: N_2_H_4_·H_2_O (20 equiv), *i-*PrOH, MW heating 100 °C, 1 h; Method F: MeSNa (4 equiv), DMA, rt, 1 h.

## Conclusion

In summary, a novel methodology for the synthesis of 2-fluoro-6-fluoroalkylpyridines and 2-fluoroalkylpyridines, including ring-fused products, is presented based on thermal decomposition of *N*-fluoroalkyl-4-alkenyl-1,2,3-triazoles available by CuAAC click reaction. The synthetic pathway proceeds via ketenimines, followed by a series of HF elimination, fluorine shift, and cyclization steps, leading to new pyridines of an uncommon substitution pattern. This mechanistic scenario was corroborated by control mechanistic experiments. Follow-up transformations of 2-fluoro-6-fluoroalkylpyridines via nucleophilic aromatic substitution provide a series of substituted 2-fluoroalkylpyridines. This synthetic strategy offers a metal-free, step-economical route to fluorinated pyridine scaffolds that are widely relevant in pharmaceuticals and agrochemicals.

## Supplementary Material



## Data Availability

The data underlying this study are available in the published article, in its Supporting Information, and openly available in Zenodo at https://zenodo.org/records/18171358.

## Notes

Experimental part, NMR data and copies of NMR spectra, and crystal structures of **11m** (CCDC 2499113), **18f** (CCDC 2499114), and **19g** (CCDC 2499115) (PDF)
