# Supplementary material for: Synthesis of 2‑Fluoroalkylated Pyridines and Fluoropyridines by Thermal Denitrogenation of N‑Fluoroalkyl-1,2,3-triazoles and Cyclization of Ketenimines
Source: J Org Chem. 2026 Jan 22;91(5):2232–8. doi: 10.1021/acs.joc.5c03046 (PMC12887997; doi:10.1021/acs.joc.5c03046)

Supporting Information

Synthesis of 2-Fluoroalkylated Pyridines and Fluoropyridines by Thermal Denitrogenation of *N*-Fluoroalkyl-1,2,3-triazoles and Cyclization of Ketenimines

Svatava Voltrová, Blanka Klepetářová, Petr Beier*

Institute of Organic Chemistry and Biochemistry, Czech Academy of Sciences, Flemingovo nám. 2, 160 00 Prague, Czechia

beier@uochb.cas.cz

Contents

1 General information S2

2 Synthesis of starting triazoles **6** and **12** S3

3 Preparation of 2-fluoro-6-fluoroalkylpyridines **11** S7

4 Preparation of compound **14a** S11

5 Preparation of 2-fluoroalkylpyridines **18** S11

6 Functionalization of pyridines **11** by S_NAr. Preparation of compounds **19** S13

7 Crystallographic data S17

8 References S17

9 Copies of NMR spectra S19

10 X-ray crystallography plots S196

1 General information

All commercially available chemicals were used as received unless stated otherwise, column chromatography was performed using silica gel 60 (0.040–0.063 mm). Automated flash column chromatography was performed on Teledyne ISCO CombiFlash Rf+ Lumen Automated Flash Chromatography System with UV/Vis detection. ^{1}H, ^{13}C, and ^{19}F NMR spectra were measured at ambient temperature using 5 mm diameter NMR tubes. ^{13}C NMR spectra were proton decoupled. The chemical shift values (σ) are reported in ppm relative to internal Me_4Si (0 ppm for ^{1}H and ^{13}C NMR) or residual solvents and internal $CFCl_3$ (0 ppm for ^{19}F NMR). Coupling constants (*J*) are reported in Hertz. Structural elucidation was aided by additional acquisition of ^{13}C APT, 1D ^{1}H NOESY and/or various 2D spectra (^{1}H-^{1}H COSY, ^{1}H-^{13}C HSQC, ^{1}H-^{13}C HMBC, ^{13}C-^{19}F HMBC, ^{1}H-^{1}H ROESY). High resolution mass spectra (HRMS) were recorded on a Waters Micromass AutoSpec Ultima or Agilent 7890A GC coupled with Waters GCT Premier orthogonal acceleration time-of-flight detector using electron impact (EI) ionization, on an LTQ Orbitrap XL using electrospray ionization (ESI), and on a Bruker solariX 94 ESI/MALDI-FT-ICR using dual ESI/MALDI ionization. Microwave experiments were done on CEM Focused Microwave™ Synthesis System, Model Discover. Unless stated otherwise, the method was set-up to 300 W, temperature 140–160 °C. All microwave experiments were performed in closed vessels. Both the solution and the neat experiments were tested for 1 mmol substrate scale with similar yields as the 0.5 mmol scale used in most experiments. LRMS spectra were recorded on Agilent 7890A GC (column HP-5MS, 30 m × 0.25 mm × 0.25 μm, 5% phenyl methylpolysiloxane) coupled with 5975C quadrupole mass selective electron impact (EI) detector (70 eV). The melting points were measured on Stuart SMP 30 instrument and are uncorrected. *Safety note:* The release of nitrogen gas increases the internal pressure of the vial in the microwave. Corrosion of the glass reaction vessel by the formed hydrogen fluoride gas was observed.

2 Synthesis of starting triazoles **6** and **12**

*Procedure A: Triazoles **6a-6h**, **6l**, **6m**, **6q**, **6r**, **12a**, **12d-12f**, **12h** (according to ref.[1])*

(*E*)-1-(Perfluoroethyl)-4-styryl-1*H*-1,2,3-triazole (6a) Yield: 78%, white solid; ^{1}H NMR (401 MHz, CDCl$_3$) δ 7.93 (s, 1H), 7.55–7.48 (m, 3H), 7.42–7.36 (m, 2H), 7.35–7.31 (m, 1H), 7.07 (d, *J* = 16.5 Hz, 1H); ^{13}C{^{1}H} NMR (101 MHz, CDCl$_3$) δ 147.1, 136.1, 133.8, 129.0, 128.8, 126.9, 118.4, 117.1 (qt, *J* = 287.6, 41.3 Hz), 114.5, 110.3 (tq, *J* = 270.7, 43.2 Hz); ^{19}F NMR (376 MHz, CDCl$_3$) δ –84.4 (s, 3F), –99.2 (s, 2F); HRMS (ESI$^+$) *m/z* calcd for $C_{12}H_9N_3F_5$: 290.0711, found 290.0712.

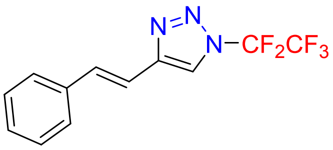

(*E*)-4-(4-Methylstyryl)-1-(perfluoroethyl)-1*H*-1,2,3-triazole (6b) Yield: 76%, white solid; ^{1}H NMR (400 MHz, CDCl$_3$) δ 7.90 (s, 1H), 7.48 (d, *J* = 16.4 Hz, 1H), 7.42 (d, *J* = 8.2 Hz, 2H), 7.19 (d, *J* = 7.9 Hz, 2H), 7.02 (d, *J* = 16.4 Hz, 1H), 2.38 (s, 3H); ^{13}C{^{1}H} NMR (101 MHz, CDCl$_3$) δ 147.3, 138.9, 133.7, 133.3, 129.7, 126.9, 118.2, 117.1 (qt, *J* = 287.2, 41.4 Hz), 113.5, 110.3 (tq, *J* = 270.7, 43.2 Hz), 21.4; ^{19}F NMR (376 MHz, CDCl$_3$) δ –84.4 (s, 3F), –99.2 (s, 2F); HRMS (ESI$^+$) *m/z* calcd for $C_{13}H_{11}N_3F_5$: 304.0867, found 304.0867.

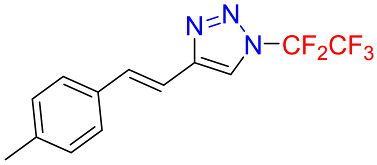

(*E*)-4-(4-Methoxystyryl)-1-(perfluoroethyl)-1*H*-1,2,3-triazole (6c) Yield: 62%, white solid; ^{1}H NMR (400 MHz, CDCl$_3$) δ 7.88 (s, 1H), 7.49–7.40 (m, 3H), 6.97–6.88 (m, 3H), 3.83 (s, 3H); ^{13}C{^{1}H} NMR (101 MHz, CDCl$_3$) δ 160.2, 147.5, 133.3, 128.9, 128.3, 117.9, 117.1 (qt, *J* = 287.6, 41.4 Hz), 114.4, 112.3, 110.3 (tq, *J* = 270.3, 42.9 Hz), 55.5; ^{19}F NMR (376 MHz, CDCl$_3$) δ –84.4 (s, 3F), –99.3 (s, 2F); HRMS (ESI$^+$) *m/z* calcd for $C_{13}H_{11}N_3F_5O$: 320.0817, found 320.0816.

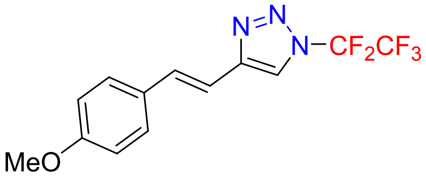

(*E*)-4-(4-Bromostyryl)-1-(perfluoroethyl)-1*H*-1,2,3-triazole (6d) Yield: 60%, off-white solid; ^{1}H NMR (400 MHz, CDCl$_3$) δ 7.92 (s, 1H), 7.53–7.49 (m, 2H), 7.45 (d, *J* = 16.4 Hz, 1H), 7.40–7.36 (m, 2H), 7.04 (d, *J* = 16.3 Hz, 1H); ^{13}C{^{1}H} NMR (101 MHz, CDCl$_3$) δ 146.7, 134.9, 132.4, 132.0, 128.3, 122.6, 118.5, 117.0 (qt, *J* = 287.8, 41.4 Hz), 115.0, 110.1 (tq, *J* = 270.9, 42.9 Hz); ^{19}F NMR (376 MHz, CDCl$_3$) δ –84.4 (s, 3F), –99.3 (s, 2F); HRMS (ESI$^+$) *m/z* calcd for $C_{12}H_8N_3BrF_5$: 367.9816, found 367.9818.

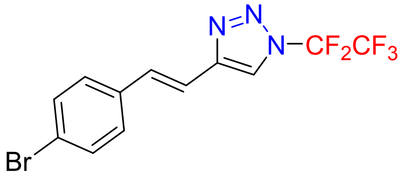

(*E*)-4-(4-Chlorostyryl)-1-(perfluoroethyl)-1*H*-1,2,3-triazole (6e) Yield: 88%, off-white solid; ^{1}H NMR (400 MHz, CDCl$_3$) δ 7.92 (s, 1H), 7.51–7.41 (m, 3H), 7.37–7.32 (m, 2H), 7.02 (d, *J* = 16.4 Hz, 1H); ^{13}C{^{1}H} NMR (101 MHz, CDCl$_3$) δ 146.7, 134.5, 134.4, 132.3, 129.1, 128.0, 118.5, 117.0 (qt, *J* = 287.6, 41.3 Hz), 114.9, 110.1 (tq, *J* = 270.9, 43.1 Hz); ^{19}F NMR (376 MHz, CDCl$_3$) δ –84.4 (s, 3F), –99.3 (s, 2F); HRMS (ESI$^+$) *m/z* calcd for $C_{12}H_8N_3ClF_5$: 324.0321, found 324.0321.

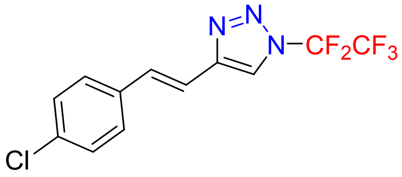

(*E*)-4-(4-Methylstyryl-1-(1,1,2,2-tetrafluoroethyl)-1*H*-1,2,3-triazole (6f) Yield: 83%, white solid; ^{1}H NMR (400 MHz, $CDCl_3$) δ 7.94 (s, 1H), 7.42 (d, J = 7.7 Hz, 3H), 7.19 (d, J = 7.9 Hz, 2H), 7.02 (d, J = 16.4 Hz, 1H), 6.63 (tt, J = 52.4, 4.6 Hz, 1H), 2.38 (s, 3H); ^{13}C{^{1}H} NMR (101 MHz, $CDCl_3$) δ 147.2, 138.9, 133.5, 133.4, 129.7, 126.9, 117.8, 113.8, 112.2 (t, J = 265.9 Hz), 107.8 (tt, J = 253.8, 35.5 Hz), 21.5; ^{19}F NMR (376 MHz, $CDCl_3$) δ –99.5 (td, J = 7.9, 4.7 Hz, 2F), –137.8 (dt, J = 52.4, 7.8 Hz, 2F); HRMS (ESI$^+$) *m/z* calcd for $C_{13}H_{12}N_3F_4$: 286.0962, found 286.0962.

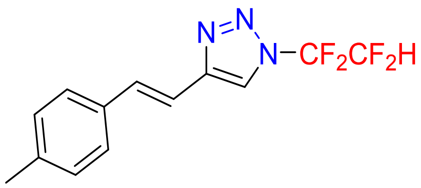

E)-4-(4-Methoxystyryl)-1-(1,1,2,2-tetrafluoroethyl)-1*H*-1,2,3-triazole (6g) Yield: 89%, white solid, ^{1}H NMR (400 MHz, $CDCl_3$) δ 7.92 (s, 1H), 7.45 (dd, J = 8.6, 1.8 Hz, 2H), 7.40 (s, 1H), 6.95–6.89 (m, 3H), 6.63 (tt, J = 52.4, 4.6 Hz, 1H), 3.83 (s, 3H); ^{13}C{^{1}H} NMR (101 MHz, $CDCl_3$) δ 160.2, 147.3, 133.0, 128.9, 128.2, 117.6, 114.4, 112.5, 112.2 (t, J = 265.9 Hz), 107.8 (tt, J = 253.8, 35.6 Hz), 55.4; ^{19}F NMR (376 MHz, $CDCl_3$) δ –99.5 (td, J = 7.8, 4.6 Hz, 2F), –137.8 (dt, J = 52.3, 7.8 Hz, 2F); HRMS (ESI$^+$) *m/z* calcd for $C_{13}H_{12}N_3F_4O$: 302.0911, found 302.0910.

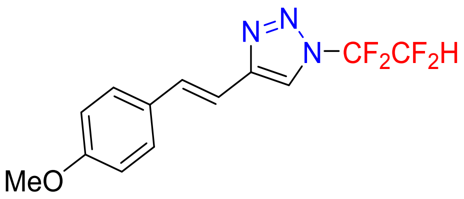

(*E*)-4-(4-Chlorostyryl)-1-(1,1,2,2-tetrafluoroethyl)-1*H*-1,2,3-triazole (6h) Yield: 84%, white solid; ^{1}H NMR (400 MHz, $CDCl_3$) δ 7.96 (s, 1H), 7.49–7.41 (m, 3H), 7.37–7.33 (m, 2H), 7.03 (d, J = 16.4 Hz, 1H), 6.63 (tt, J = 52.4, 4.5 Hz, 1H); ^{13}C{^{1}H} NMR (101 MHz, $CDCl_3$) δ 146.7, 134.6, 134.4, 132.1, 129.2, 128.1, 118.3, 115.3, 112.2 (t, J = 29.3 Hz), 107.8 (tt, J = 254.2, 35.6 Hz); ^{19}F NMR (376 MHz, $CDCl_3$) δ –99.5 (td, J = 7.8, 4.6 Hz, 2F), –137.8 (dt, J = 52.3, 7.7 Hz, 2F); HRMS (ESI$^+$) *m/z* calcd for $C_{12}H_9N_3ClF_4$: 306.0416, found 306.0416.

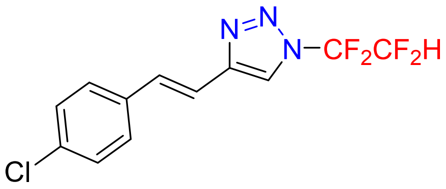

4-(Cyclohex-1-en-1-yl)-1-(1,1,2,2-tetrafluoro-2-(phenylthio)ethyl)-1*H*-1,2,3-triazole (6l) Yield: 65%, yellow oil; ^{1}H NMR (400 MHz, $CDCl_3$) δ 7.65–7.58 (m, 3H), 7.53–7.44 (m, 1H), 7.44–7.35 (m, 2H), 6.69 (tt, J = 3.9, 1.8 Hz, 1H), 2.40–2.30 (m, 2H), 2.28–2.18 (m, 2H), 1.84–1.71 (m, 2H), 1.74–1.63 (m, 2H); ^{13}C{^{1}H} NMR (101 MHz, $CDCl_3$) δ 150.0, 137.3, 131.1, 129.5, 127.6, 126.0, 122.9 (t, J = 2.9 Hz), 122.2 (tt, J = 291.6, 39.6 Hz), 116.8, 113.6 (tt, J = 272.9, 35.8 Hz), 26.3, 25.4, 22.4, 22.1; ^{19}F NMR (376 MHz, $CDCl_3$) δ –89.6 (t, J = 6.4 Hz, 2F), –95.6 (t, J = 6.4 Hz, 2F); HRMS (ESI$^+$) *m/z* calcd for $C_{16}H_{16}N_3F_4S$: 358.0996, found 358.0997.

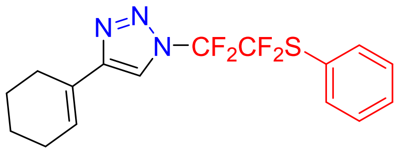

4-(Cyclohex-1-en-1-yl)-1-(1,1,2,2-tetrafluoro-2-(4-methoxyphenoxy)ethyl)-1*H*-1,2,3-triazole (6m) Yield: 81%, white solid; ^{1}H NMR (400 MHz, $CDCl_3$) δ 7.76 (s, 1H), 7.10–7.01 (m, 2H), 6.89–6.81 (m, 2H), 6.72 (tt, J = 4.0, 1.8 Hz, 1H), 3.79 (s, 3H), 2.43–2.34 (m, 2H), 2.28–2.18 (m, 2H), 1.84–1.73 (m, 2H), 1.75–1.63 (m, 2H); ^{13}C{^{1}H} NMR (101 MHz, $CDCl_3$) δ 158.3, 149.9, 141.8 (t, J = 1.9 Hz), 127.6, 126.0, 123.1, 116.8, 115.9 (tt, J = 275.5, 37.8 Hz), 114.8, 111.6 (tt, J = 270.6, 41.9 Hz), 55.7, 26.4, 25.4, 22.4, 22.2; ^{19}F NMR (376 MHz, $CDCl_3$) δ –86.6 (t, J = 3.8 Hz, 2F), –99.2 (t, J = 3.7 Hz, 2F); HRMS (ESI$^+$) *m/z* calcd for $C_{17}H_{18}N_3F_4O_2$: 372.1330, found 372.1331.

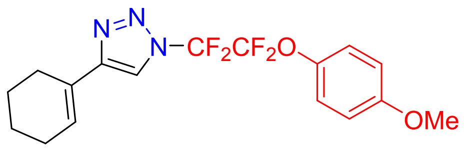

(*E*)-1-(Perfluoroethyl)-4-(1-phenylprop-1-en-2-yl)-1*H*-1,2,3-triazole (6q) Yield: 86%, white solid; ^{1}H NMR (400 MHz, $CDCl_3$) δ 7.89 (s, 1H), 7.59 (d, J = 1.5 Hz, 1H), 7.40 (d, J = 4.4 Hz, 4H), 7.35–7.24 (m, 1H), 2.30 (d, J = 1.5 Hz, 3H); ^{13}C{^{1}H} NMR (101 MHz, $CDCl_3$) δ 151.4, 136.7, 129.8, 129.4, 128.5, 127.5, 124.6, 117.7, 117.2 (qt, J = 287.6, 41.4 Hz), 110.3 (tq, J = 270.5, 43.3 Hz), 16.2; ^{19}F NMR (376 MHz, $CDCl_3$) δ –84.4 (s, 3F), –99.2 (s, 2F); HRMS (EI^+) *m/z* calcd for $C_{13}H_{10}N_3F_5$: 303.079, found 303.078.

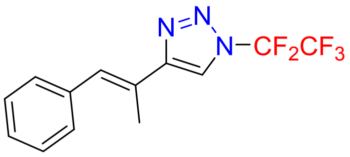

1-(Perfluoroethyl)-4-phenethyl-1*H*-1,2,3-triazole (12a) Yield: 95%, white solid; ^{1}H NMR (400 MHz, $CDCl_3$) δ 7.49 (s, 1H), 7.29 (t, J = 7.4 Hz, 2H), 7.21 (t, J = 7.3 Hz, 1H), 7.16 (d, J = 6.7 Hz, 2H), 3.19–3.10 (m, 2H), 3.10–3.02 (m, 2H); ^{13}C{^{1}H} NMR (101 MHz, $CDCl_3$) δ 148.3, 140.4, 128.6, 128.5, 126.5, 119.8, 117.1 (qt, J = 287.5, 41.6 Hz), 110.2 (tq, J = 270.1, 43.0 Hz), 35.1, 27.2; ^{19}F NMR (376 MHz, $CDCl_3$) δ –84.6 (s, 3F), –99.3 (s, 2F); HRMS (ESI^+) *m/z* calcd for $C_{12}H_{11}N_3F_5$: 292.0868, found 292.0867.

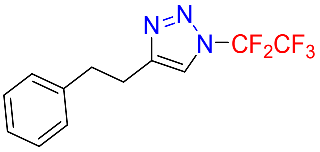

4-(4-Chlorobutyl)-1-(perfluoroethyl)-1*H*-1,2,3-triazole (12d) Yield: 96%, colorless liquid; ^{1}H NMR (400 MHz, $CDCl_3$) δ 7.71 (s, 1H), 3.57 (t, J = 6.1 Hz, 2H), 2.83 (t, J = 6.9 Hz, 2H), 1.95–1.82 (m, 4H); ^{13}C{^{1}H} NMR (101 MHz, $CDCl_3$) δ 148.7, 119.4, 116.9 (qt, J = 287.2, 41.4 Hz), 110.0 (tq, J = 270.0, 43.1 Hz), 44.4, 31.7, 26.1, 24.4; ^{19}F NMR (376 MHz, $CDCl_3$) δ –84.5 (s, 3F), –99.1 (s, 2F); HRMS (ESI^+) *m/z* calcd for $C_8H_{10}N_3ClF_5$: 278.0478, found 278.0477.

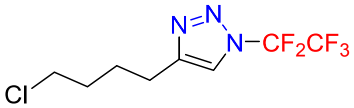

4-(4-Chlorobutyl)-1-(1,1,2,2-tetrafluoro-2-phenoxyethyl)-1*H*-1,2,3-triazole (12e) Yield: 82%, colorless liquid; ^{1}H NMR (400 MHz, $CDCl_3$) δ 7.76 (s, 1H), 7.39–7.33 (m, 2H), 7.31–7.25 (m, 1H), 7.18–7.10 (m, 2H), 3.57 (t, J = 6.1 Hz, 2H), 2.88–2.79 (m, 2H), 1.96–1.81 (m, 4H); ^{13}C{^{1}H} NMR (101 MHz, $CDCl_3$) δ 148.5 (t, J = 1.8 Hz), 148.3, 129.9, 127.1, 121.7, 119.9, 115.9 (tt, J = 276.6, 38.0 Hz), 111.5 (tt, J = 270.7, 41.6 Hz), 44.6, 32.0, 26.4, 24.7; ^{19}F NMR (376 MHz, $CDCl_3$) δ –86.3 (t, J = 3.6 Hz, 2F), –99.3 (t, J = 3.6 Hz, 2F); HRMS (ESI^+) *m/z* calcd for $C_{14}H_{15}N_3ClF_4O$: 352.0834, found 352.0832.

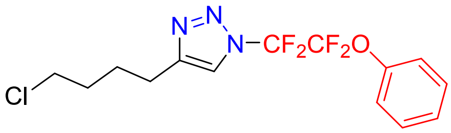

1-(2-((4-Bromophenyl)thio)-1,1,2,2-tetrafluoroethyl)-4-(4-chlorobutyl-1*H*-1,2,3-triazole (12f) Yield: 77%, white solid; ^{1}H NMR (400 MHz, $CDCl_3$) δ 7.61 (s, 1H), 7.56–7.51 (m, 2H), 7.50–7.45 (m, 2H), 3.57 (t, J = 6.0 Hz, 2H), 2.81 (dt, J = 9.5, 4.6 Hz, 2H), 1.84–1.88 (m, 4H); ^{13}C{^{1}H} NMR (101 MHz, $CDCl_3$) δ 148.3, 138.5, 132.7, 126.3, 121.8 (t, J = 2.9 Hz), 121.7 (tt, J = 292.0, 39.3 Hz), 119.7, 113.3 (tt, J = 271.8, 35.4 Hz), 44.5, 31.8, 26.2, 24.6; ^{19}F NMR (376 MHz, $CDCl_3$) δ –89.7 (t, J = 6.3 Hz, 2F), –95.5 (t, J = 6.3 Hz, 2F); HRMS (ESI^+) *m/z* calcd for $C_{14}H_{14}N_3BrClF_4S$: 445.9711, found 445.9717.

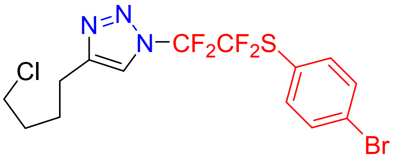

4-(4-Iodobutyl)-1-(perfluoroethyl)-1*H*-1,2,3-triazole (12h) Yield: 69%, colorless liquid; ^{1}H NMR (400 MHz, $CDCl_3$) δ 7 7.71 (s, 1H), 3.22 (t, J = 6.5 Hz, 2H), 2.82 (t, J = 7.2 Hz, 2H), 1.99–1.76 (m, 4H); ^{13}C{^{1}H} NMR (101 MHz, $CDCl_3$) δ 148.7, 119.4, 117.1 (qt, J = 287.2, 41.4 Hz), 110.2 (tq, J = 270.2, 42.9 Hz), 32.7, 29.8, 24.3, 6.1; ^{19}F NMR (376 MHz, $CDCl_3$) δ –84.4 (s, 3F), –99.1 (s, 2F); HRMS (EI^+) *m/z* calcd for $C_8H_9N_3F_5I$: 368.976, found 368.975.

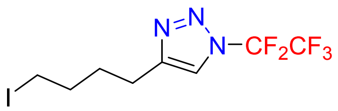

*Procedure **B**: 5-Chloro- and 5-Iodotriazoles **6i**, **6p**, **6s**, **12g** (according to ref.[2])*

(*E*)-5-Chloro-1-(perfluoroethyl)-4-styryl-1*H*-1,2,3-triazole (6i) Yield: 59%, pale yellow solid; ^{1}H NMR (400 MHz, CDCl$_3$) δ 7.70 (d, *J* = 16.4 Hz, 1H), 7.60–7.52 (m, 2H), 7.40 (t, *J* = 7.2 Hz, 2H), 7.37–7.31 (m, 1H), 6.91 (d, *J* = 16.4 Hz, 1H); ^{13}C{^{1}H} NMR (101 MHz, CDCl$_3$) δ 143.2, 135.9, 134.7, 129.1, 129.0, 127.1, 122.3, 117.1 (qt, *J* = 287.9, 39.6 Hz), 111.9, 110.8 (tq, *J* = 272.5, 43.6 Hz); ^{19}F NMR (376 MHz, CDCl$_3$) δ –82.8 (s, 3F), –97.7 (s, 2F); HRMS (EI$^+$) *m/z* calcd for $C_{12}H_7N_3ClF_5$: 323.0243, found 323.0233.

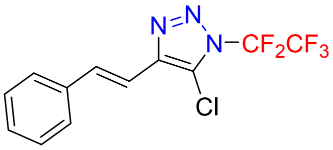

(*E*)-1-(Difluoromethyl)-5-iodo-4-styryl-1*H*-1,2,3-triazole (6p) Yield: 61%, pale yellow solid; ^{1}H NMR (400 MHz, CDCl$_3$) δ 7.73 (d, *J* = 16.3 Hz, 1H), 7.60 (t, *J* = 57.5 Hz, 1H), 7.59–7.54 (m, 2H), 7.43–7.37 (m, 2H), 7.36–7.30 (m, 1H), 6.90 (d, *J* = 16.2 Hz, 1H); ^{13}C{^{1}H} NMR (101 MHz, CDCl$_3$) δ 151.2, 136.2, 134.3, 128.9, 128.9, 127.1, 113.8, 111.0 (t, *J* = 256.4 Hz), 71.6 (t, *J* = 2.2 Hz); ^{19}F NMR (376 MHz, CDCl$_3$) δ –96.2 (d, *J* = 57.5 Hz); HRMS (ESI$^+$) *m/z* calcd for $C_{11}H_9N_3F_2I$: 347.9804, found 347.9803.

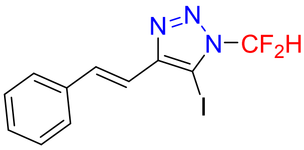

5-Chloro-4-cyclohexyl-1-(perfluoroethyl)-1*H*-1,2,3-triazole (12g) Yield: 70%, colorless liquid; ^{1}H NMR (400 MHz, CDCl$_3$) δ 2.74 (tt, *J* = 12.0, 3.4 Hz, 1H), 1.96–1.81 (m, 4H), 1.78–1.62 (m, 3H), 1.46–1.30 (m, 3H); ^{13}C{^{1}H} NMR (101 MHz, CDCl$_3$) δ 149.9, 122.3, 117.1 (qt, *J* = 287.9, 39.6 Hz), 110.8 (tq, *J* = 271.4, 43.3 Hz), 34.4, 31.3, 26.3, 25.8; ^{19}F NMR (376 MHz, CDCl$_3$) δ –82.7 (s, 3F), –97.4 (s, 2F); HRMS (APCI$^+$) *m/z* calcd for $C_{10}H_{12}N_3ClF_5$: 304.0634, found 304.0634.

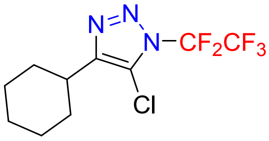

3 Preparation of 2-fluoro-6-fluoroalkylpyridines **11**

A 10 ml MW tube was charged with triazole **6** (0.5 mmol) and under inert atmosphere DCE (3 ml) and KF (1 equiv., 29 mg, 0.5 mmol) were added. The reaction mixture was heated under MW irradiation to 140 °C for 1 h. After completion of the reaction the crude reaction mixture was purified by column chromatography.

6-Fluoro-3-phenyl-2-(trifluoromethyl)pyridine (11a) Yield: 83%, purified by column chromatography (cyclohexane/15% EtOAc); colorless liquid; ^{1}H NMR (401 MHz, $CDCl_3$) δ 7.84 (t, *J* = 7.8 Hz, 1H), 7.47–7.42 (m, 3H), 7.35–7.28 (m, 2H), 7.19 (dd, *J* = 8.4, 3.4 Hz, 1H); ^{13}C{^{1}H} NMR (126 MHz, $CDCl_3$) δ 161.5 (d, *J* = 243.8 Hz), 146.0 (d, *J* = 7.5 Hz), 142.9 (qd, *J* = 34.2, 13.0 Hz), 136.0, 135.3 (d, *J* = 4.5 Hz), 128.9 (d, *J* = 1.8 Hz), 128.7, 128.4, 121.1 (q, *J* = 275.9 Hz), 112.7 (d, *J* = 36.5 Hz); ^{19}F NMR (377 MHz, $CDCl_3$) δ –62.1 (s, 3F), –68.3 (dd, *J* = 7.3, 3.4 Hz, 1F); HRMS (EI$^+$) *m/z* calcd for $C_{12}H_7NF_4$: 241.0509, found 241.0509. *Scale-up experiment:* Triazole **6a** (289 mg, 1 mmol) was dissolved in DCE (3 mL), KF (58 mg, 1 mmol) was added and the reaction mixture was heated in a MW reactor at 140 °C for 1 h. The reaction mixture was filtered and the resulting dark liquid was was purified by column chromatography (cyclohexane/15% EtOAc) to obtain **11a** as colorless liquid (180 mg, 75%).

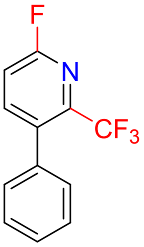

6-Fluoro-3-(*p*-tolyl)-2-(trifluoromethyl)pyridine (11b) Yield: 65%, purified by column chromatography (cyclohexane/15% EtOAc); colorless liquid; ^{1}H NMR (401 MHz, $CDCl_3$) δ 7.84–7.79 (m, 1H), 7.26–7.24 (m, 2H), 7.20 (d, *J* = 8.1 Hz, 2H), 7.17 (ddd, *J* = 8.3, 3.5, 0.6 Hz, 1H), 2.42 (s, 3H); ^{13}C{^{1}H} NMR (126 MHz, $CDCl_3$) δ 161.3 (d, *J* = 243.5 Hz), 145.9 (d, *J* = 7.5 Hz), 142.8 (qd, *J* = 34.1, 13.0 Hz), 138.5, 135.2 (d, *J* = 4.6 Hz), 132.9, 129.0, 128.7 (d, *J* = 1.7 Hz), 121.0 (q, *J* = 275.9 Hz), 112.5 (d, *J* = 36.6 Hz), 21.3; ^{19}F NMR (377 MHz, $CDCl_3$) δ –62.2 (s, 3F), –68.6 (dd, *J* = 7.4, 3.4 Hz, 1F); HRMS (EI$^+$) *m/z* calcd for $C_{13}H_9NF_4$: 255.0666, found 255.0663.

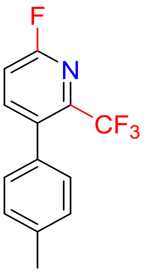

6-Fluoro-3-(4-methoxyphenyl)-2-(trifluoromethyl)pyridine (11c) Yield: 66%, purified by column chromatography (cyclohexane/15% EtOAc); colorless liquid; ^{1}H NMR (401 MHz, $CDCl_3$) δ 7.85–7.77 (m, 1H), 7.25–7.22 (m, 2H), 7.18–7.14 (m, 1H), 7.01–6.93 (m, 2H), 3.86 (s, 3H); ^{13}C{^{1}H} NMR (126 MHz, $CDCl_3$) δ 161.2 (d, *J* = 243.8 Hz), 159.8, 146.0 (d, *J* = 7.5 Hz), 142.8 (qd, *J* = 33.8, 12.9 Hz), 135.0 (d, *J* = 4.7 Hz), 130.0 (t, *J* = 1.8 Hz), 128.0, 121.0 (q, *J* = 275.8 Hz), 113.8, 112.5 (d, *J* = 36.6 Hz), 55.3; ^{19}F NMR (377 MHz, $CDCl_3$) δ –62.2 (s, 3F), –68.8 (dd, *J* = 7.3, 3.5 Hz, 1F); HRMS (EI$^+$) *m/z* calcd for $C_{13}H_9NF_4O$: 271.0615, found 271.0610.

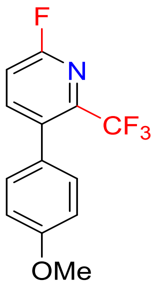

6-Fluoro-3-(4-bromophenyl)-2-(trifluoromethyl)pyridine (11d) Yield: 80%, purified by column chromatography (cyclohexane/15% EtOAc); colorless liquid; ^{1}H NMR (401 MHz, $CDCl_3$) δ 7.83–7.79 (m, 1H), 7.61–7.56 (m, 2H), 7.23–7.15 (m, 3H); ^{13}C{^{1}H} NMR (126 MHz, $CDCl_3$) δ 161.7 (d, *J* = 244.7 Hz), 145.7 (d, *J* = 7.5 Hz), 143.1 (qd, *J* = 34.3, 13.1 Hz), 134.8, 134.0 (d, *J* = 4.7 Hz), 131.7, 130.6 (d, *J* = 1.8 Hz), 123.3, 121.0 (q, *J* = 275.8 Hz), 112.9 (d, *J* = 36.7 Hz); ^{19}F NMR (377 MHz, $CDCl_3$) δ –62.1 (s, 3F), –67.7 (dd, *J* = 7.4, 3.4 Hz, 1F); HRMS (EI$^+$) *m/z* calcd for $C_{12}H_6NBrF_4$: 318.9614, found 318.9616.

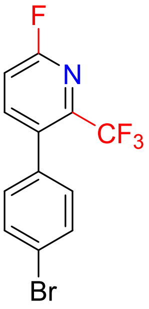

6-Fluoro-3-(4-chlorophenyl)-2-(trifluoromethyl)pyridine (11e) Yield: 54%, purified by column chromatography (cyclohexane/15% EtOAc); colorless liquid; ^{1}H NMR (401 MHz, $CDCl_3$) δ 7.84–7.79 (m, 1H), 7.45–7.41 (m, 2H), 7.27–7.23 (m, 2H), 7.20 (dd, *J* = 8.4, 3.4 Hz, 1H); ^{13}C{^{1}H} NMR (126 MHz, $CDCl_3$) δ 161.7 (d, *J* = 244.6 Hz), 145.8 (d, *J* = 7.5 Hz), 143.1 (qd, *J* = 34.3, 12.8 Hz), 135.1, 134.3, 134.1 (d, *J* = 4.6 Hz), 130.3 (q, *J* = 1.8 Hz), 128.8, 121.0 (q, *J* = 275.5 Hz), 112.8 (d, *J* = 36.5 Hz); ^{19}F NMR (377 MHz, $CDCl_3$) δ –62.2 (s, 3F), –67.6 (dd, *J* = 7.3, 3.4 Hz, 1F); HRMS (EI^+) *m/z* calcd for $C_{12}H_6NClF_4$: 275.0119, found 275.0119.

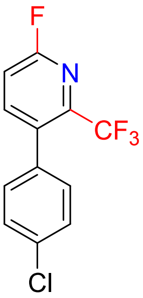

2-(Difluoromethyl)-6-fluoro-3-(p-tolyl)pyridine (11f) Yield: 63%, purified by column chromatography (cyclohexane/15% EtOAc); colorless liquid; ^{1}H NMR (401 MHz, $CDCl_3$) δ 7.86–7.77 (m, 1H), 7.33–7.26 (m, 2H), 7.28–7.20 (m, 2H), 7.11 (ddt, *J* = 8.4, 3.3, 1.1 Hz, 1H), 6.53 (t, *J* = 53.8 Hz, 1H), 2.43 (s, 3H); ^{13}C{^{1}H} NMR (126 MHz, $CDCl_3$) δ 162.4 (d, *J* = 243.0 Hz), 146.7 (td, *J* = 22.1, 12.3 Hz)), 144.4 (d, *J* = 7.6 Hz), 138.9, 135.3 (dd, *J* = 8.7, 4.3 Hz), 132.3, 129.6, 129.3, 112.0 (d, *J* = 37.1 Hz), 111.0 (t, *J* = 239.9 Hz), 21.3; ^{19}F NMR (377 MHz, $CDCl_3$) δ –68.3 (d, *J* = 7.5 Hz, 1F), –113.6 (d, *J* = 53.7 Hz, 2F); HRMS (EI^+) *m/z* calcd for $C_{13}H_{10}NF_3$: 237.0760, found 237.0753.

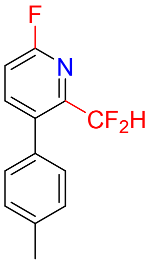

2-(Difluoromethyl)-6-fluoro-3-(4-methoxyphenyl)pyridine (11g) Yield: 70%, purified by column chromatography (cyclohexane/15% EtOAc); colorless liquid; ^{1}H NMR (401 MHz, $CDCl_3$) δ 7.81 (ddt, *J* = 8.4, 7.3, 1.0 Hz, 1H), 7.27 (d, *J* = 8.7 Hz, 2H), 7.10 (ddt, *J* = 8.4, 3.3, 1.1 Hz, 1H), 7.00 (d, *J* = 8.7 Hz, 2H), 6.52 (t, *J* = 53.8 Hz, 1H), 3.87 (s, 3H); ^{13}C{^{1}H} NMR (126 MHz, $CDCl_3$) δ 162.3 (d, *J* = 242.9 Hz), 160.2, 146.5 (td, *J* = 22.3, 12.8 Hz), 144.4 (d, *J* = 7.7 Hz), 135.0 (q, *J* = 4.3 Hz), 130.7, 127.4, 114.4, 111.9 (dt, *J* = 36.9, 1.7 Hz), 111.1 (t, *J* = 240.1 Hz), 55.5; ^{19}F NMR (377 MHz, $CDCl_3$) δ –68.5 (d, *J* = 7.5 Hz, 1F), –113.6 (d, *J* = 53.8 Hz, 2F); HRMS (EI^+) *m/z* calcd for $C_{13}H_{10}NF_3O$: 253.0709, found 253.0708.

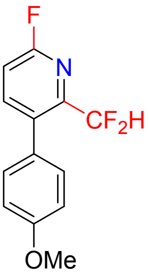

2-(Difluoromethyl)-6-fluoro-3-(4-chlorophenyl)pyridine (11h) Yield: 60%, purified by column chromatography (cyclohexane/15% EtOAc); colorless liquid; ^{1}H NMR (401 MHz, $CDCl_3$) δ 7.80 (ddt, *J* = 8.3, 7.3, 0.9 Hz, 1H), 7.50–7.43 (m, 2H), 7.3–7.28 (m, 2H), 7.14 (ddt, *J* = 8.4, 3.3, 1.1 Hz, 1H), 6.49 (t, *J* = 53.8 Hz, 1H); ^{13}C{^{1}H} NMR (126 MHz, $CDCl_3$) δ 162.5 (d, *J* = 243.9 Hz), 146.9 (td, *J* = 23.0, 12.9 Hz), 144.4 (d, *J* = 7.7 Hz), 135.3, 134.1 (q, *J* = 3.7 Hz), 133.8, 130.7, 129.1, 112.1 (d, *J* = 37.1 Hz), 111.6 (t, *J* = 240.8 Hz); ^{19}F NMR (377 MHz, $CDCl_3$) δ –67.7 (d, *J* = 7.5 Hz, 1F), –112.9 (d, *J* = 53.7 Hz, 2F); HRMS (EI^+) *m/z* calcd for $C_{12}H_7NClF_3$: 257.0214, found 257.0210.

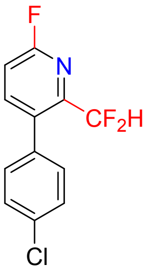

3-Chloro-2-fluoro-5-phenyl-6-(trifluoromethyl)pyridine (11i) Yield: 38%, purified by column chromatography (cyclohexane/15% EtOAc); colorless liquid; ^{1}H NMR (401 MHz, $CDCl_3$) δ 7.88 (d, *J* = 8.1 Hz, 1H), 7.49–7.41 (m, 3H), 7.35–7.28 (m, 2H); ^{13}C{^{1}H} NMR (126 MHz, $CDCl_3$) δ 156.8 (d, *J* = 243.1 Hz), 144.9, 140.6 (qd, *J* = 35.0, 11.4 Hz), 136.7 (d, *J* = 5.2 Hz), 134.7, 129.1, 128.7, 128.5, 120.8 (q, *J* = 275.5 Hz), 120.4 (d, *J* = 33.9 Hz); ^{19}F NMR (377 MHz, $CDCl_3$) δ –61.9 (s, 3F), –71.9 (d, *J* = 8.2 Hz, 1F); HRMS (EI$^+$) *m/z* calcd for $C_{12}H_6NClF_4$: 275.0119, found 275.0118.

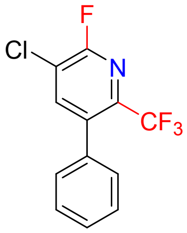

3-Fluoro-1-(trifluoromethyl)-5,6,7,8-tetrahydroisoquinoline (11j) Yield: 76%, purified by column chromatography (cyclohexane/15% EtOAc); colorless liquid; ^{1}H NMR (401 MHz, $CDCl_3$) δ 6.84 (d, *J* = 2.6 Hz, 1H), 2.95–2.84 (m, 4H), 1.88–1.77 (m, 4H); ^{13}C{^{1}H} NMR (126 MHz, $CDCl_3$) δ 160.3 (d, *J* = 240.0 Hz), 155.4 (d, *J* = 7.0 Hz), 143.5 (dd, *J* = 33.7, 13.2 Hz), 130.2 (d, *J* = 4.4 Hz), 121.7 (q, *J* = 275.9 Hz), 112.4 (d, *J* = 35.2 Hz), 30.1 (d, *J* = 2.5 Hz), 24.1 (q, *J* = 2.7 Hz), 22.2, 21.4; ^{19}F NMR (377 MHz, $CDCl_3$) δ –65.6 (s, 3F), –73.5 (s, 1F); HRMS (EI$^+$) *m/z* calcd for $C_{10}H_9NF_4$: 219.0666, found 219.0665.

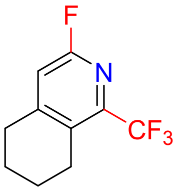

1-(Difluoromethyl)-3-fluoro-5,6,7,8-tetrahydroisoquinoline (11k) Yield: 76%, colorless liquid; unstable (decomposed during isolation); crude product used for the analyses (HRMS, NMR – small impurities in the aliphatic region) and immediately for the next step – preparation of the 4-methylthio derivative **19k**, which was found to be stable and could be fully characterized (see section 6); ^{1}H NMR (401 MHz, $CDCl_3$) δ 6.75–6.73 (m, 1H), 6.55 (t, *J* = 54.4 Hz, 1H), 2.98–2.90 (m, 2H), 2.88–2.80 (m, 2H), 1.86–1.77 (m, 4H); ^{13}C{^{1}H} NMR (126 MHz, $CDCl_3$) δ 160.8 (d, *J* = 238.6 Hz), 154.7 (d, *J* = 7.0 Hz), 147.4 (td, *J* = 25.3, 12.7 Hz), 130.1 (d, *J* = 4.3 Hz), 115.7 (t, *J* = 241.4 Hz), 111.1 (d, *J* = 35.2 Hz), 30.0 (d, *J* = 2.6 Hz), 23.5, 22.2, 21.6; ^{19}F NMR (377 MHz, $CDCl_3$) δ –74.9 (s, 1F), –115.9 (d, *J* = 54.3 Hz), 2F); HRMS (EI$^+$) *m/z* calcd for $C_{10}H_{10}NF_3$: 201.0760, found 201.0758.

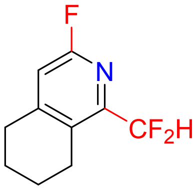

1-(Difluoro(phenylthio)methyl)-3-fluoro-5,6,7,8-tetrahydroisoquinoline (11l) Yield: 80%, purified by column chromatography (cyclohexane/15% EtOAc); colorless liquid; ^{1}H NMR (401 MHz, $CDCl_3$) δ 7.72–7.64 (m, 2H), 7.50–7.41 (m, 1H), 7.45–7.35 (m, 2H), 6.76 (d, *J* = 2.7 Hz, 1H), 3.02–2.94 (m, 2H), 2.88–2.84 (m, 2H), 1.86–1.73 (m, 4H); ^{13}C{^{1}H} NMR (126 MHz, $CDCl_3$) δ 160.0 (d, *J* = 239.4 Hz), 155.1 (d, *J* = 6.9 Hz), 147.9 (td, *J* = 27.3, 12.5 Hz), 137.0, 130.1, 129.8 (d, *J* = 4.3 Hz), 129.1, 127.8 (t, *J* = 278.9 Hz), 126.9, 111.4 (d, *J* = 35.2 Hz), 30.3 (d, *J* = 2.5 Hz), 24.7 (t, *J* = 4.3 Hz), 22.4, 21.5; ^{19}F NMR (377 MHz, $CDCl_3$) δ –71.1 (s, 2F), –74.1 (s, 1F); HRMS (ESI$^+$) *m/z* calcd for $C_{16}H_{15}NF_3S$: 310.0872, found 310.0872.

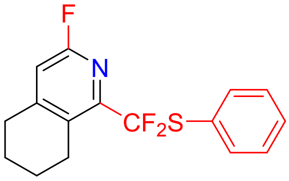

1-(Difluoro(4-methoxyphenoxy)methyl)-3-fluoro-5,6,7,8-tetrahydroisoquinoline (11m) Yield: 86%, purified by column chromatography (cyclohexane/15% EtOAc); white crystals, m. p. 89–91 °C (hexane); ^{1}H NMR (401 MHz, $CDCl_3$) δ 7.25–7.16 (m, 2H), 6.92–6.83 (m, 2H), 6.79 (d, *J* = 2.7 Hz, 1H), 3.80 (s, 3H), 3.06 (t, *J* = 6.0 Hz, 2H), 2.88 (t, *J* = 6.2 Hz, 2H), 1.89–1.79 (m, 4H); ^{13}C{^{1}H} NMR (126 MHz, $CDCl_3$) δ 160.2 (d, *J* = 238.5 Hz), 157.5, 155.0 (d, *J* = 6.9 Hz), 146.4 (td, *J* = 32.8, 12.9 Hz), 143.6 (t, *J* = 2.0 Hz), 130.0 (d, *J* = 4.3 Hz), 123.4, 120.0 (t, *J* = 264.6 Hz), 114.5, 111.4 (d, *J* = 35.3 Hz), 55.7, 30.2 (d, *J* = 2.5 Hz), 24.5 (t, *J* = 3.1 Hz), 22.5, 21.6; ^{19}F NMR (377 MHz, $CDCl_3$) δ –70.0 (s, 2F), –74.0 (s, 1F); HRMS (ESI$^+$) *m/z* calcd for $C_{17}H_{17}NF_3O_2$: 324.1206, found 324.1208. For

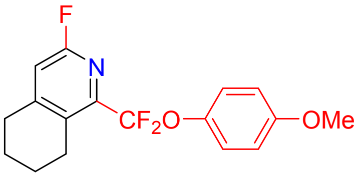

X-ray analysis, the compound was crystallized from hexane by slow cooling of the saturated solution (40 °C to 20 °C during 3 days).

3-Fluoro-6-phenyl-1-(trifluoromethyl)-5,6,7,8-tetrahydroisoquinoline (11n) Yield: 72%, purified by column chromatography (cyclohexane/15% EtOAc); colorless liquid; ^{1}H NMR (401 MHz, $CDCl_3$) δ 7.41–7.33 (m, 2H), 7.31–7.23 (m, 3H), 6.92 (d, J = 2.9 Hz, 1H), 3.32 (dd, J = 16.4, 3.8 Hz, 1H), 3.10–3.03 (m, 2H), 3.01–2.87 (m, 2H), 2.22–2.15 (m, 1H), 2.04–1.90 (m, 1H); ^{13}C{^{1}H} NMR (126 MHz, $CDCl_3$) δ 160.5 (d, J = 240.4 Hz), 154.6 (d, J = 7.1 Hz), 144.8, 143.6 (qd, J = 33.9, 13.2 Hz), 129.8 (d, J = 4.4 Hz), 128.9, 127.0, 126.9, 121.6 (q, J = 275.9 Hz), 112.3 (d, J = 35.3 Hz), 39.8, 32.1 (d, J = 2.8 Hz), 30.3 (d, J = 2.5 Hz), 28.6; ^{19}F NMR (377 MHz, $CDCl_3$) δ –65.6 (s, 3F), –73.0 (s, 1F); HRMS (EI$^+$) *m/z* calcd for $C_{16}H_{13}NF_4$: 295.0979, found 295.0983.

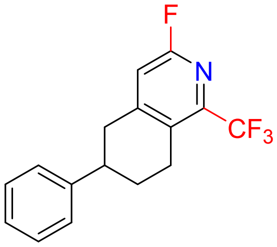

1,3-Difluoro-5,6,7,8-tetrahydroisoquinoline (11o) Yield: 55%, purified by column chromatography (cyclohexane/15% EtOAc); colorless liquid; ^{1}H NMR (401 MHz, $CDCl_3$) δ 6.52 (s, 1H), 2.82–2.74 (m, 2H), 2.67–2.62 (m, 2H), 1.86–1.75 (m, 4H); ^{13}C{^{1}H} NMR (126 MHz, $CDCl_3$) δ 160.5 (dd, J = 15.2, 13.4 Hz), 158.0 (dd, J = 15.2, 11.5 Hz), 156.5 (t, J = 6.8 Hz), 115.5 (dd, J = 30.1, 5.4 Hz), 105.3 (dd, J = 33.3, 5.3 Hz), 29.4 (t, J = 3.1 Hz), 21.9, 21.8, 21.4; ^{19}F NMR (377 MHz, $CDCl_3$) δ –74.7 (d, J = 12.5 Hz, 1F), –76.3 (d, J = 12.5 Hz, 1F); HRMS (ESI$^+$) *m/z* calcd for $C_9H_{10}NF_2$: 170.0776, found 170.0777.

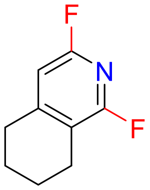

2-Fluoro-3-iodo-5-phenylpyridine (11p) Yield: 83%, purified by column chromatography (cyclohexane/15% EtOAc); colorless liquid; ^{1}H NMR (401 MHz, $CDCl_3$) δ 8.37–8.31 (m, 2H), 7.54–7.39 (m, 5H); ^{13}C{^{1}H} NMR (126 MHz, $CDCl_3$) δ 161.6 (d, J = 234.8 Hz), 148.6 (d, J = 3.1 Hz), 145.4 (d, J = 13.5 Hz), 136.8 (d, J = 5.1 Hz), 135.4, 129.4, 128.7, 127.2, 76.1 (d, J = 44.0 Hz); ^{19}F NMR (377 MHz, $CDCl_3$) δ –59.1 (d, J = 7.5 Hz); HRMS (EI$^+$) *m/z* calcd for $C_{11}H_7NFI$: 298.9602, found 298.9605.

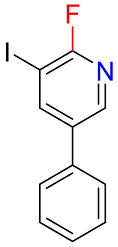

4 Preparation of compound **14a**

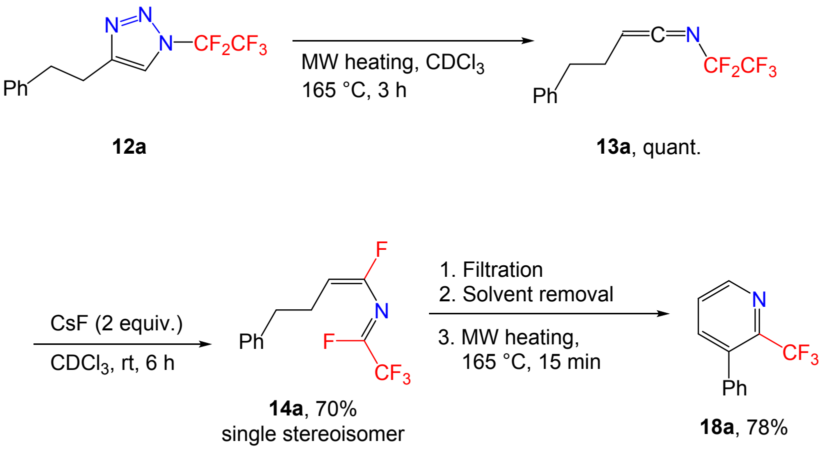

A 10 ml MW tube was charged with triazole **12a** (146 mg, 0.5 mmol) in $CDCl_3$ (3 ml) and the reaction mixture was heated under MW irradiation to 165 °C for 3 h to form the ketenimine **13a**. Quantitative formation was observed by ^{19}F NMR spectroscopy: ^{19}F NMR (377 MHz, $CDCl_3$) δ –85.9 (s, 3F), –97.5 (2 s, 2F), cf. Ref.[3]

To the solution of the formed ketenimine **13a** the overnight-dried (135 °C, vacuo) CsF (152 mg, 1 mmol, 2.0 equiv.) was added and the reaction mixture was left at ambient temperature for 6 h. The suspension was then filtered and the filtrate carefully evaporated under inert gas. Only the single stereoisomer **14a** (70% by ^{19}F NMR yield) was observed in the reaction mixture: ^{19}F NMR (377 MHz, $CDCl_3$) δ –46.5 (dq, J = 20.0, 5.0 Hz, 1F), –73.0 (d, J = 5.3 Hz, 3F), –94.1 (dd, J = 19.9, 9.4 Hz, 1F), cf. Ref.[4]

The oily residue containing product **14a** (92 mg, 0.35 mmol) was heated in a MW reactor at 165 °C for 5 min. After cooling, the crude reaction mixture was purified by column chromatography (cyclohexane/EtOAc) to afford 3-phenyl-2-(trifluoromethyl)pyridine (**18a**) (61 mg, 0.273 mmol, 78%).

5 Preparation of 2-fluoroalkylpyridines **18**

A 10 ml MW tube was charged with triazole **12** (0.5 mmol) and the reaction mixture was heated under MW irradiation to 165 °C for 15 min. After completion of the reaction the crude reaction mixture was purified by column chromatography.

3-Phenyl-2-(trifluoromethyl)pyridine (18a) Yield: 56%, purified by column chromatography (cyclohexane/15% EtOAc); colorless liquid; ^{1}H NMR (401 MHz, $CDCl_3$) δ 8.72 (dd, J = 4.7, 1.7 Hz, 1H), 7.73 (dd, J = 7.8, 1.7 Hz, 1H), 7.53 (dd, J = 7.8, 4.7 Hz, 1H), 7.48–7.41 (m, 3H), 7.36–7.30 (m, 2H); ^{13}C{^{1}H} NMR (126 MHz, $CDCl_3$) δ 148.0, 145.3 (q, J = 32.3 Hz), 140.4, 137.4, 137.2, 128.9 (q, J = 1.7 Hz), 128.5, 128.3, 125.9, 122.0 (q, J = 275.8 Hz); ^{19}F NMR (377 MHz, $CDCl_3$) δ –61.8 (s, 3F); HRMS (EI$^+$) m/z calcd for $C_{12}H_8NF_3$: 223.0603, found 223.0597.

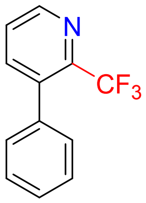

3-Decyl-2-(trifluoromethyl)pyridine (18b) Yield: 41%, purified by column chromatography (cyclohexane/15% EtOAc); colorless liquid; ^{1}H NMR (401 MHz, CDCl$_3$) δ 8.53 (dd, *J* = 4.7, 1.3 Hz, 1H), 7.69 (d, *J* = 7.5 Hz, 1H), 7.41 (dd, *J* = 7.8, 4.6 Hz, 1H), 2.84–2.74 (m, 2H), 1.70–1.55 (m, 2H), 1.47–1.17 (m, 14H), 0.88 (t, *J* = 6.8 Hz, 3H); ^{13}C{^{1}H} NMR (126 MHz, CDCl$_3$) δ 146.4, 145.6 (q, *J* = 32.3 Hz), 139.4, 137.5, 126.2, 122.4 (q, *J* = 275.5 Hz), 31.9, 31.3 (q, *J* = 2.2 Hz), 31.2, 29.6, 29.5, 29.5, 29.3, 29.3, 22.7, 14.1; ^{19}F NMR (377 MHz, CDCl$_3$) δ –64.2 (s); HRMS (EI$^+$) *m/z* calcd for $C_{16}H_{25}NF_3$: 288.1934, found 288.1935.

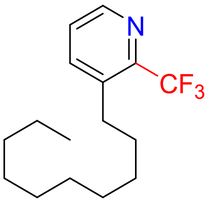

1-Trifluoromethyl-5,6,7,8-tetrahydroisoquinoline (18c) Yield: 39%, purified by column chromatography (cyclohexane/15% EtOAc); colorless liquid; ^{1}H NMR (401 MHz, CDCl$_3$) δ 8.34 (d, *J* = 4.9 Hz, 1H), 7.16 (d, *J* = 4.9 Hz, 1H), 2.97–2.89 (m, 2H), 2.88–2.80 (m, 2H), 1.91–1.78 (m, 4H); ^{13}C{^{1}H} NMR (126 MHz, CDCl$_3$) δ 149.0, 145.9 (q, *J* = 31.9 Hz), 145.1, 132.3, 127.1, 122.5 (q, *J* = 275.8 Hz), 29.5, 24.3 (q, *J* = 2.9 Hz), 22.2, 21.6; ^{19}F NMR (377 MHz, CDCl$_3$) δ –65.7 (s); HRMS (EI$^+$) *m/z* calcd for $C_{10}H_{10}NF_3$: 201.0760, found 201.0758. *Scale up experiment:* Triazole **12c** (300 mg, 1.12 mmol) was heated in a microwave reactor at 165 °C for 15 min. The dark reaction mixture was purified by column chromatography (cyclohexane/15% EtOAc) to obtain **18c** as a pale-yellow liquid (81 mg, 36%).

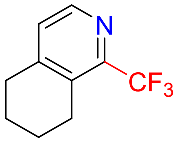

3-(2-Chloroethyl)-2-(trifluoromethyl)pyridine (18d) Yield: 40%, purified by column chromatography (cyclohexane/15% EtOAc); colorless liquid; ^{1}H NMR (401 MHz, CDCl$_3$) δ 8.61 (dd, *J* = 4.7, 1.6 Hz, 1H), 7.80 (ddq, *J* = 7.8, 1.6, 0.6 Hz, 1H), 7.48 (dd, *J* = 7.9, 4.7 Hz, 1H), 3.75 (t, *J* = 6.9 Hz, 2H), 3.30 (tq, *J* = 6.3, 1.4 Hz, 2H); ^{13}C{^{1}H} NMR (126 MHz, CDCl$_3$) δ 147.6, 146.2 (q, *J* = 32.9 Hz), 140.6, 132.4, 126.2, 122.5 (q, *J* = 288.8 Hz), 43.6, 34.5 (q, *J* = 2.3 Hz); ^{19}F NMR (377 MHz, CDCl$_3$) δ –64.0 (s); HRMS (EI$^+$) *m/z* calcd for $C_8H_7NClF_3$: 209.0214, found 209.0212.

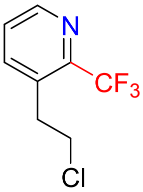

3-(2-Chloroethyl)-2-(difluoro(phenoxy)methyl)pyridine (18e) Yield: 41%, purified by column chromatography (cyclohexane/15% EtOAc); colorless liquid; ^{1}H NMR (401 MHz, CDCl$_3$) δ 8.63 (dd, *J* = 4.7, 1.6 Hz, 1H), 7.78 (dd, *J* = 7.8, 1.6 Hz, 1H), 7.44 (dd, *J* = 7.8, 4.7 Hz, 1H), 7.40–7.36 (m, 2H), 7.35–7.28 (m, 2H), 7.30–7.21 (m, 1H), 3.81 (t, *J* = 7.2 Hz, 2H), 3.42 (t, *J* = 7.2 Hz, 2H); ^{13}C{^{1}H} NMR (126 MHz, CDCl$_3$) δ 150.1, 148.9 (t, *J* = 31.8 Hz), 147.3, 140.5, 132.3, 129.6, 126.0, 125.5, 122.0, 120.5 (t, *J* = 265.0 Hz), 43.9 (t, *J* = 1.7 Hz), 35.0 (t, *J* = 2.3 Hz); ^{19}F NMR (377 MHz, CDCl$_3$) δ –68.2 (s); HRMS (ESI$^+$) *m/z* calcd for $C_{14}H_{13}NClF_2O$: 284.0648, found 284.0649.

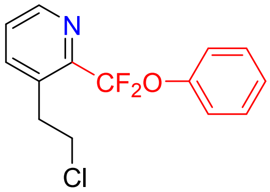

2-(((4-Bromophenyl)thio)difluoromethyl)-3-(2-chloroethyl)pyridine (18f) Yield: 32%, purified by column chromatography (cyclohexane/15% EtOAc); light yellow crystals, m. p. 144–146 °C (Et_2O/hexane); ^{1}H NMR (401 MHz, CDCl$_3$) δ 8.56 (dd, *J* = 4.7, 1.6 Hz, 1H), 7.76 (dd, *J* = 7.8, 1.6 Hz, 1H), 7.57–7.53 (m, 4H), 7.42 (dd, *J* = 7.8, 4.7 Hz, 1H), 3.73 (t, *J* = 7.0 Hz, 2H), 3.30 (tt, *J* = 6.9, 1.6 Hz, 2H); ^{13}C{^{1}H} NMR (126 MHz, CDCl$_3$) δ 150.5 (t, *J* = 27.5 Hz), 147.1, 140.9, 138.5, 132.4, 132.1, 129.3 (t, *J* = 277.1 Hz), 126.2 (t, *J* = 1.7 Hz), 125.7, 125.2, 44.1, 34.7; ^{19}F NMR (377 MHz, CDCl$_3$) δ –67.4 (s); HRMS (EI$^+$) *m/z* calcd for $C_{14}H_{11}NBrClF_2S$: 376.9447, found 376.9446. For X-ray analysis, the compound was crystallized from hexane by slow cooling of the saturated solution (40 °C to 20 °C during 2 days).

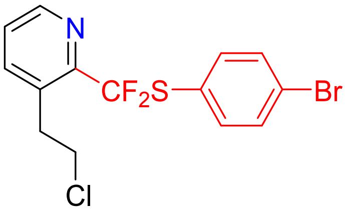

4-Chloro-1-(trifluoromethyl)-5,6,7,8-tetrahydroisoquinoline (18g) Yield: 38%, colorless liquid; unstable (decomposed during isolation); crude product used for the analyses (NMR, HRMS) and immediately for the next step – preparation of the 4-methylthio derivative **19p**, which was found to be stable and could be fully characterized (see the next paragraph); ^{1}H NMR (401 MHz, $CDCl_3$) δ 7.21 (s, 1H), 2.92–2.85 (m, 2H), 2.86–2.79 (m, 2H), 1.87–1.77 (m, 4H); $^{13}C\{^1H\}$ NMR (126 MHz, $CDCl_3$) δ 152.7, 147.2, 145.8 (q, J = 33.4 Hz), 131.6, 127.4, 121.7 (q, J = 276.1 Hz), 29.6, 24.1 (q, J = 2.8 Hz), 22.1, 21.4; ^{19}F NMR (377 MHz, $CDCl_3$) δ –65.6 (s); HRMS (EI$^+$) *m/z* calcd for $C_{10}H_9NClF_3$: 235.0370, found 235.0371.

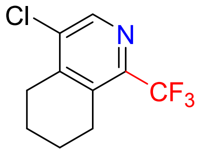

6 Functionalization of pyridines **11** by S_NAr. Preparation of compounds **19**

Method A: A 10 ml MW tube was charged with pyridine **11** (0.3 mmol), then water (2 ml) and NaOH (4 equiv., 48 mg, 1.2 mmol) were added. The reaction mixture was heated under MW irradiation to 140 °C for 1 h. After completion of the reaction the crude product was extracted to DCM and after drying purified by column chromatography.

5-Phenyl-6-(trifluoromethyl)pyridin-2-ol (19a) Made from **11a**. Yield: 72%, purified by column chromatography (cyclohexane); colorless solid; ^{1}H NMR (401 MHz, $CDCl_3$) δ 11.28 (br s, 1H), 7.54 (d, J = 8.9 Hz, 1H), 7.46–7.38 (m, 3H), 7.31–7.29 (m, 2H), 6.94 (d, J = 9.0 Hz, 1H); $^{13}C\{^1H\}$ NMR (126 MHz, $CDCl_3$) δ 162.8, 144.8, 136.0, 135.7 (q, J = 34.1 Hz), 129.1, 128.5, 128.4, 125.8, 120.9 (q, J = 275.9 Hz), 119.6; ^{19}F NMR (377 MHz, $CDCl_3$) δ –61.2 (s); HRMS (EI$^+$) *m/z* calcd for $C_{12}H_8NF_3O$: 239.0552, found 239.0550.

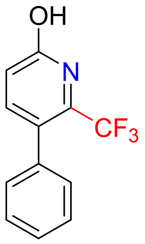

1-(Trifluoromethyl)-5,6,7,8-tetrahydroisoquinolin-3-ol (19b) Made from **11j**. Yield: 73%, purified by column chromatography (cyclohexane); colorless solid; ^{1}H NMR (401 MHz, $CDCl_3$) δ 10.52 (br s, 1H), 6.63 (s, 1H), 2.82–2.74 (m, 4H), 1.80–1.73 (m, 4H); $^{13}C\{^1H\}$ NMR (126 MHz, $CDCl_3$) δ 161.4, 154.0, 137.0 (q, J = 33.0 Hz), 122.2, 121.7 (q, J = 275.6 Hz), 117.1, 30.0, 23.6 (q, J = 2.5 Hz), 22.4, 21.7; ^{19}F NMR (377 MHz, $CDCl_3$) δ –64.5 (s); HRMS (EI$^+$) *m/z* calcd for $C_{10}H_{10}NF_3O$: 217.0709, found 217.0704.

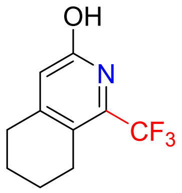

Method B: A 10 ml screw-cap tube was charged with pyridine **11** (0.3 mmol), then EtOH (2 ml) and EtONa (2 equiv., 41 mg, 0.6 mmol) were added. The reaction mixture was stirred at room temp. for 16 h. After completion of the reaction the crude product was concentrated, dissolved in DCM and purified by column chromatography.

6-Ethoxy-3-phenyl-2-(trifluoromethyl)pyridine (19c) Made from **11a**. Yield: 62%, purified by column chromatography (cyclohexane/15% EtOAc); yellow oil; ^{1}H NMR (401 MHz, $CDCl_3$) δ 7.56 (d, J = 8.4 Hz, 1H), 7.44–7.38 (m, 3H), 7.36–7.26 (m, 2H), 6.92 (d, J = 8.4 Hz, 1H), 4.48 (q, J = 7.0 Hz, 2H), 1.44 (t, J = 7.0 Hz, 3H); $^{13}C\{^1H\}$ NMR (126 MHz, $CDCl_3$) δ 162.3, 143.0, 141.9 (q, J = 32.7 Hz), 137.6, 130.0 (d, J = 1.6 Hz), 129.1 (q, J = 1.7 Hz), 128.2, 127.9, 121.9 (q, J = 275.5 Hz), 113.8, 62.6, 14.6; ^{19}F NMR (377 MHz, $CDCl_3$) δ –62.0 (s); HRMS (EI$^+$) *m/z* calcd for $C_{14}H_{12}NF_3O$: 267.0866, found 267.0862.

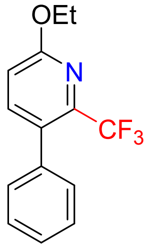

3-Ethoxy-1-(trifluoromethyl)-5,6,7,8-tetrahydroisoquinoline (19d) Made from **11j**. Yield: 87%, purified by column chromatography (cyclohexane/15% EtOAc); colorless oil; ^{1}H NMR (401 MHz, $CDCl_3$) δ 6.59 (s, 1H), 4.34 (q, *J* = 7.1 Hz, 2H), 2.87–2.82 (m, 2H), 2.82–2.71 (m, 2H), 1.84–1.72 (m, 4H), 1.36 (t, *J* = 7.1 Hz, 3H); $^{13}C\{^{1}H\}$ NMR (126 MHz, $CDCl_3$) δ 160.7, 151.7, 142.6 (q, *J* = 32.3 Hz), 125.0, 122.6 (q, *J* = 275.7 Hz), 113.3, 62.0, 29.9, 23.9 (q, *J* = 2.7 Hz), 22.7, 21.9, 14.7; ^{19}F NMR (377 MHz, $CDCl_3$) δ –65.2 (s); HRMS (EI^+) *m/z* calcd for $C_{12}H_{14}NF_3O$: 245.1022, found 245.1011.

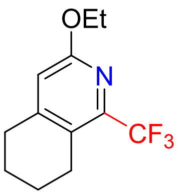

Method C: A 10 ml MW tube was charged with pyridine **11** (0.3 mmol), then DMA (1.5 ml) and PhONa (3 equiv., 105 mg, 0.9 mmol) were added. The reaction mixture was heated under MW irradiation to 140 °C for 1 h. After completion of the reaction the crude product was purified by column chromatography.

6-Phenoxy-3-phenyl-2-(trifluoromethyl)pyridine (19e) Made from **11a**. Yield: 74%, purified by column chromatography (cyclohexane/15% EtOAc); colorless solid; ^{1}H NMR (401 MHz, $CDCl_3$) δ 7.68 (d, *J* = 8.4 Hz, 1H), 7.45–7.41 (m, 5H), 7.35–7.30 (m, 2H), 7.27–7.22 (m, 3H), 7.04 (d, *J* = 8.4 Hz, 1H); $^{13}C\{^{1}H\}$ NMR (126 MHz, $CDCl_3$) δ 161.9, 153.7, 144.0, 142.8 (q, *J* = 33.4 Hz), 137.0, 132.0, 129.9, 129.1 (q, *J* = 1.8 Hz), 128.3, 128.2, 125.3, 121.5 (q, *J* = 275.8 Hz), 121.2, 113.5; ^{19}F NMR (377 MHz, $CDCl_3$) δ –62.0 (s); HRMS (ESI^+) *m/z* calcd for $C_{18}H_{13}NF_3O$: 316.09438, found 316.09433.

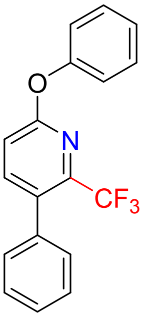

Method D: A 10 ml MW tube was charged with pyridine **11** (0.3 mmol), then DMA (1.5 ml) and the corresponding amine (3 equiv., 0.9 mmol) was added. The reaction mixture was heated under MW irradiation to 140 °C for 1 h. After completion of the reaction the crude product was purified by column chromatography.

***N*-(4-Methylbenzyl-5-phenyl-6-(trifluoromethyl)pyridin-2-amine (19f)** Made from **11a**. Yield: 89%, purified by column chromatography (cyclohexane/15% EtOAc); colorless oil; ^{1}H NMR (401 MHz, $CDCl_3$) δ 7.44–7.37 (m, 4H), 7.32–7.29 (m, 4H), 7.22–7.17 (m, 2H), 6.55 (d, *J* = 8.5 Hz, 1H), 5.18 (t, *J* = 5.8 Hz, 1H), 4.54 (d, *J* = 5.8 Hz, 2H), 2.37 (s, 3H); $^{13}C\{^{1}H\}$ NMR (126 MHz, $CDCl_3$) δ 156.9, 143.0 (q, *J* = 31.9 Hz), 141.9, 138.3, 137.3, 135.7, 129.5, 129.3 (q, *J* = 1.8 Hz), 128.1, 127.7, 127.5, 126.1, 122.2 (q, *J* = 275.8 Hz), 109.1, 46.2, 21.2; ^{19}F NMR (377 MHz, $CDCl_3$) δ –62.0 (s); HRMS (ESI^+) *m/z* calcd for $C_{20}H_{18}N_2F_3$: 343.14166, found 343.14155.

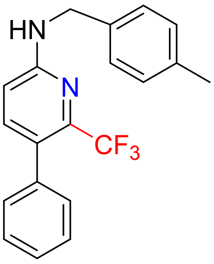

***N*,*N*-Dimethyl-7-phenyl-1-(trifluoromethyl)-5,6,7,8-tetrahydroisoquinolin-3-amine (19g)** Made from **11n**. Yield: 57%, purified by column chromatography (cyclohexane/15% EtOAc); colorless crystals, m. p. (dec.) 123–125 °C (hexane); ^{1}H NMR (401 MHz, $CDCl_3$) δ 7.39–7.30 (m, 2H), 7.33–7.21 (m, 3H), 6.42 (s, 1H), 3.25–3.14 (m, 1H), 3.08 (s, 6H), 3.00–2.88 (m, 3H), 2.86–2.75 (m, 1H), 2.17–2.05 (m, 1H), 1.99–1.84 (m, 1H); $^{13}C\{^{1}H\}$ NMR (126 MHz, $CDCl_3$) δ 156.5, 149.0, 146.1, 143.6 (q, *J* = 31.6 Hz), 128.7, 127.0, 126.6, 122.8 (q, *J* = 276.1 Hz), 118.7, 107.8, 40.6, 37.9, 31.9 (q, *J* = 2.5 Hz), 30.4, 29.3; ^{19}F NMR (377 MHz, $CDCl_3$) δ –65.4 (s); HRMS (EI^+) *m/z* calcd for $C_{18}H_{19}N_2F_3$: 320.1495, found 320.1503. For X-ray analysis, the compound was crystallized from pentane by slow cooling of the saturated solution (30 °C to 20 °C during 2 weeks).

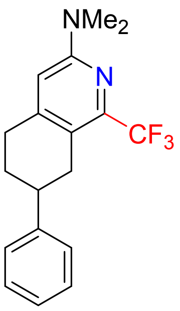

_N,N_-Dibenzyl-5-(4-methoxyphenyl)-6-(trifluoromethyl)pyridin-2-amine (19h) Made from **11c**. Yield: 76%, purified by column chromatography (cyclohexane/15% EtOAc); colorless solid; ^{1}H NMR (401 MHz, $CDCl_3$) δ 7.37–7.28 (m, 11H), 7.27–7.20 (m, 2H), 6.93 (d, J = 8.7 Hz, 2H), 6.59 (d, J = 9.0 Hz, 1H), 4.86 (s, 4H), 3.85 (s, 3H); $^{13}C\{^{1}H\}$ NMR (126 MHz, $CDCl_3$) δ 159.1, 156.5, 142.7 (q, J = 31.9 Hz), 142.1, 138.1, 130.6, 130.4, 128.8, 127.6, 127.4, 124.8, 122.3 (q, J = 276.2 Hz), 113.5, 108.3, 55.4, 51.3; ^{19}F NMR (377 MHz, $CDCl_3$) δ –62.1 (s); HRMS (ESI$^+$) *m/z* calcd for $C_{27}H_{24}N_2F_3O$: 449.1835, found 449.1835.

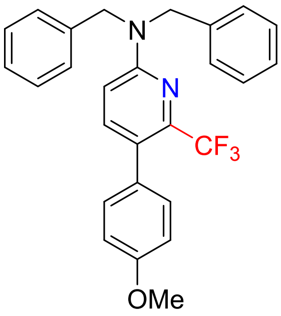

Method E: A 10 ml MW tube was charged with pyridine **11** (0.3 mmol), then *i*-PrOH (2 ml) and hydrazine monohydrate (20 equiv., 0.29 ml, 6 mmol) were added. The reaction mixture was heated under MW irradiation to 100 °C for 1 h. After completion of the reaction the crude product was purified by column chromatography.

6-Hydrazinyl-3-phenyl-2-(trifluoromethyl)pyridine (19i) Made from **11a**. Yield: 63%, purified by column chromatography (cyclohexane/20% EtOAc); colorless solid; ^{1}H NMR (401 MHz, $CDCl_3$) δ 7.49 (d, J = 8.6 Hz, 1H), 7.43–7.35 (m, 3H), 7.33–7.26 (m, 2H), 6.97 (d, J = 8.5 Hz, 1H), 6.33 (s, 1H), 3.94 (s, 2H); $^{13}C\{^{1}H\}$ NMR (126 MHz, $CDCl_3$) δ 159.7, 142.6 (q, J = 32.1 Hz), 142.0, 138.0, 129.2 (q, J = 1.8 Hz), 128.1, 127.7, 127.5, 122.2 (q, J = 275.7 Hz), 109.5; ^{19}F NMR (377 MHz, $CDCl_3$) δ –62.0 (s); HRMS (EI$^+$) *m/z* calcd for $C_{12}H_{10}N_3F_3$: 253.0821, found 253.0823.

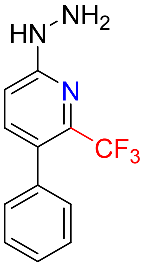

Method F: **A** 10 ml screw-cap tube was charged with pyridine **11** (0.3 mmol), then DMA (1 ml) and MeSNa (4 equiv., 84 mg, 1.2 mmol) were added. The reaction mixture was stirred at room temp. for 1 h. After completion of the reaction the crude product was purified by column chromatography.

6-(Methylthio)-3-phenyl-2-(trifluoromethyl)pyridine (19j) Made from **11a**. Yield: 67%, purified by column chromatography (cyclohexane/15% EtOAc); colorless solid; ^{1}H NMR (401 MHz, $CDCl_3$) δ 7.48 (d, J = 8.2 Hz, 1H), 7.45–7.39 (m, 3H), 7.37 (d, J = 8.3 Hz, 1H), 7.33–7.28 (m, 2H), 2.64 (s, 3H); $^{13}C\{^{1}H\}$ NMR (126 MHz, $CDCl_3$) δ 159.0, 144.6 (q, J = 32.6 Hz), 139.9, 137.2, 132.5, 129.0 (q, J = 1.7 Hz), 128.3, 128.2, 123.8, 122.0 (q, J = 276.0 Hz), 13.3; ^{19}F NMR (377 MHz, $CDCl_3$) δ –62.0 (s); HRMS (EI$^+$) *m/z* calcd for $C_{13}H_{10}NF_3S$: 269.0481, found 269.0484.

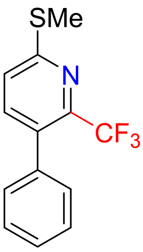

1-(Difluoromethyl)-3-(methylthio)-5,6,7,8-tetrahydroisoquinoline (19k) Made from **11k**. Yield: 61%, purified by column chromatography (cyclohexane/15% EtOAc); colorless oil; ^{1}H NMR (401 MHz, $CDCl_3$) δ 6.97 (s, 1H), 6.62 (t, J = 54.8 Hz, 1H), 2.94–2.87 (m, 2H), 2.77–2.70 (m, 2H), 2.53 (s, 3H), 1.84–1.75 (m, 4H); $^{13}C\{^{1}H\}$ NMR (126 MHz, $CDCl_3$) δ 155.2 (t, J = 1.7 Hz), 149.5 (t, J = 25.0 Hz), 148.6, 128.2, 123.4 (t, J = 1.7 Hz), 117.5 (t, J = 241.4 Hz), 29.5, 23.4, 22.4, 21.9, 13.4; ^{19}F NMR (377 MHz, $CDCl_3$) δ –115.2 (d, J = 54.8 Hz); HRMS (EI$^+$) *m/z* calcd for $C_{11}H_{13}NF_2S$: 229.0731, found 229.0725.

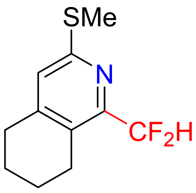

1-(Difluoro(phenylthio)methyl)-3-(methylthio)-5,6,7,8-tetrahydroisoquinoline (19l) Made from **11l**. Yield: 83%, purified by column chromatography (cyclohexane/15% EtOAc); yellowish oil; ^{1}H NMR (401 MHz, $CDCl_3$) δ 7.71–7.67 (m, 2H), 7.49–7.37 (m, 3H), 7.01 (s, 1H), 2.93–2.86 (m, 2H), 2.78–2.71 (m, 2H), 2.60 (s, 3H), 1.79–1.73 (m, 4H); ^{13}C{^{1}H} NMR (126 MHz, $CDCl_3$) δ 154.5, 149.5 (t, J = 27.8 Hz), 149.0, 137.1, 130.6 (t, J = 278.8 Hz), 129.7, 128.9, 128.2, 127.5, 124.0, 29.7, 24.0 (t, J = 4.3 Hz), 22.5, 21.9, 13.4; ^{19}F NMR (377 MHz, $CDCl_3$) δ –68.8 (s); HRMS (EI$^+$) *m/z* calcd for $C_{17}H_{17}NF_2S_2$: 337.0765, found 337.0766.

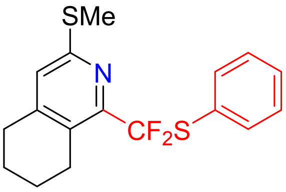

1-(Difluoro(4-methoxyphenoxy)methyl)-3-(methylthio)-5,6,7,8-tetrahydroisoquinoline (19m) Made from **11m**. Yield: 65%, purified by column chromatography (cyclohexane/15% EtOAc); colorless oil; ^{1}H NMR (401 MHz, $CDCl_3$) δ 7.25–7.17 (m, 2H), 7.01 (s, 1H), 6.91–6.82 (m, 2H), 3.80 (s, 3H), 3.06–2.98 (m, 2H), 2.80–2.70 (m, 2H), 2.56 (s, 3H), 1.88–1.73 (m, 4H); ^{13}C{^{1}H} NMR (126 MHz, $CDCl_3$) δ 157.2, 154.8, 148.7, 148.2 (t, J = 31.1 Hz), 143.9 (t, J = 2.2 Hz), 127.9, 123.6, 123.0, 120.7 (t, J = 265.0 Hz), 114.3, 55.6, 29.6, 24.4, 22.6, 21.8, 13.2; ^{19}F NMR (377 MHz, $CDCl_3$) δ –70.0 (s); HRMS (EI$^+$) *m/z* calcd for $C_{18}H_{19}NF_2O_2S$: 351.1099, found 351.1106.

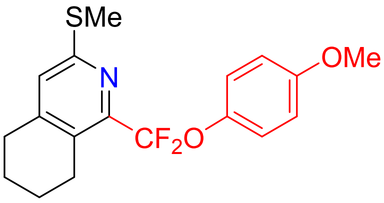

3-(Methylthio)-7-phenyl-1-(trifluoromethyl)-5,6,7,8-tetrahydroisoquinoline (19n) Made from **11n**. Yield: 88%, purified by column chromatography (cyclohexane/15% EtOAc); colorless oil; ^{1}H NMR (401 MHz, $CDCl_3$) δ 7.40–7.31 (m, 2H), 7.3–7.22 (m, 3H), 7.11 (s, 1H), 3.31–3.20 (m, 1H), 3.02–2.91 (m, 4H), 2.58 (s, 3H), 2.21–2.09 (m, 1H), 2.01–1.86 (m, 1H); ^{13}C{^{1}H} NMR (126 MHz, $CDCl_3$) δ 155.8, 148.0, 145.5 (q, J = 32.3 Hz), 145.2, 128.7, 127.3, 126.8, 126.7, 124.1, 122.3 (q, J = 276.0 Hz), 39.9, 31.9 (q, J = 2.8 Hz), 29.7, 28.7, 13.1; ^{19}F NMR (377 MHz, $CDCl_3$) δ –61.8 (s); HRMS (EI$^+$) *m/z* calcd for $C_{17}H_{16}NF_3S$: 323.0950, found 323.0956.

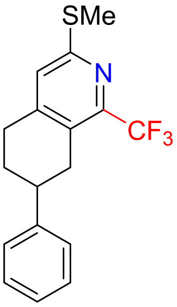

1-Fluoro-3-(methylthio)-5,6,7,8-tetrahydroisoquinoline (19o) Made from **11o**. Yield: 74%, purified by column chromatography (cyclohexane/15% EtOAc); colorless oil; ^{1}H NMR (401 MHz, $CDCl_3$) δ 6.32 (d, J = 2.2 Hz, 1H), 2.75–2.69 (m, 2H), 2.53 (s, 3H), 2.52–2.49 (m, 2H), 1.88–1.73 (m, 4H); ^{13}C{^{1}H} NMR (126 MHz, $CDCl_3$) δ 161.6 (d, J = 236.2 Hz), 157.2 (d, J = 13.9 Hz), 150.7 (d, J = 7.2 Hz), 127.1 (d, J = 4.3 Hz), 103.3 (d, J = 35.7 Hz), 29.8 (d, J = 2.7 Hz), 24.8, 22.7, 22.0, 13.2; ^{19}F NMR (377 MHz, $CDCl_3$) δ –75.7 (s); HRMS (EI$^+$) *m/z* calcd for $C_{10}H_{12}NFS$: 197.0669, found 197.0669.

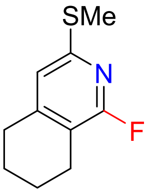

4-(Methylthio)-1-(trifluoromethyl)-5,6,7,8-tetrahydroisoquinoline (19p) Made from **18g**. Yield: 58%, purified by column chromatography (cyclohexane/15% EtOAc); colorless oil; ^{1}H NMR (401 MHz, $CDCl_3$) δ 7.04 (s, 1H), 2.89–2.81 (m, 2H), 2.80–2.72 (m, 2H), 2.55 (s, 3H), 1.87–1.72 (m, 4H); ^{13}C{^{1}H} NMR (126 MHz, $CDCl_3$) δ 155.4, 148.8, 145.4 (q, J = 32.3 Hz), 127.8, 124.3, 122.4 (q, J = 275.8 Hz), 29.5, 23.9 (q, J = 2.7 Hz), 22.3, 21.6, 13.1; ^{19}F NMR (377 MHz, $CDCl_3$) δ –65.5 (s); HRMS (ESI$^+$) *m/z* calcd for $C_{11}H_{13}NF_3S$: 248.0715, found 248.0716.

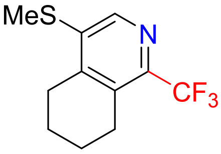

7 Crystallographic data

Single-crystal diffraction data of **11m** were collected at 180 K using Bruker D8 VENTURE system equipped with a Photon 100 CMOS detector and a CuKα Incoatec microfocus sealed tube (λ = 1.54178 Å). The frames were integrated with the with Bruker SAINT[5] software package.

Single-crystal diffraction data of **18f** and **19g** were collected on a Rigaku HF007 diffractometer equipped with a rotating copper anode (Cu Kα radiation, λ = 1.54184 Å) and a HyPix-6000HE hybrid photon counting detector at 100 K. The data were collected and processed using CrysAlisPro[6] software.

The structures were solved by direct methods by charge-flipping methods using Superflip[7] and were refined by full-matrix least-squares on F^2 with CRYSTALS.[8] The positional and anisotropic thermal parameters of all non-hydrogen atoms were refined. All hydrogen atoms were initially located in the difference Fourier map, then they were repositioned geometrically and refined with riding constraints.

Crystal data for 11m (colorless, 0.028 x 0.034 x 0.429 mm): $C_{17}H_{16}F_3NO_2$, monoclinic, space group $P2_1/n$, a = 7.4643(2) Å, b = 8.3072(2) Å, c = 24.3245(6) Å, β = 95.0272(11)°, V = 1502.50(7) Å^3, Z = 4, M = 323.31, 28161 reflections measured, 2739 independent reflections. Final R = 0.0317, wR = 0.0854, GoF = 0.9493 for 2336 reflections with I > 2σ(I) and 209 parameters. CCDC 2499113.

Crystal data for 18f (colorless, 0.060 x 0.113 x 0.521 mm): $C_{14}H_{11}BrClF_2NS$, monoclinic, space group $P2_1/c$, a = 6.9331(2) Å, b = 21.6213(7) Å, c = 9.7200(3) Å, β = 97.977(3)°, V = 1442.95(8) Å^3, Z = 4, M = 378.67, 8394 reflections measured, 2798 independent reflections. Final R = 0.0441, wR = 0.1231, GoF = 0.9664 for 2433 reflections with I > 2σ(I) and 181 parameters. CCDC 2499114.

Crystal data for 19g (colorless, 0.042 x 0.247 x 0.475 mm): $C_{18}H_{19}F_3N_2$, monoclinic, space group $C2/c$, a = 20.8511(17) Å, b = 10.0873(4) Å, c = 17.9515(14) Å, β = 124.166(12)°, V = 3124.1(6) Å^3, Z = 8, M = 320.36, 49205 reflections measured, 3073 independent reflections. Final R = 0.0747, wR = 0.2154, GoF = 1.0172 for 2699 reflections with I > 2σ(I) and 227 parameters. Two carbon atoms of the isoquinoline benzene ring were found to be disordered over two positions with occupancies of 0.817(11):0.183(11). The disorder was refined with appropriate displacement restraints. CCDC 2499115.

8 References

1 Blastik, Z. E.; Voltrová, S.; Matoušek, V.; Jurásek, B.; Manley, D. W.; Klepetářová, B.; Beier, P. *Angew. Chem., Int. Ed.* **2017**, *56*, 346.

2 Jeffery, T. *Tetrahedron.* **1996**, *52*, 10113.

3 Kubíčková, A.; Markos, A.; Voltrová, S.; Marková, A.; Filgas, J.; Klepetářová, B.; Slavíček, P.; Beier, P., *Org. Chem. Front.* **2023**, *10*, 3201.

4 Kubíčková, A.; Voltrová, S.; Kleman, A.; Klepetářová, B.; Beier, P., *Org. Chem. Front.* **2024**, *11*, 4442.

5 SAINT. Bruker AXS Inc., Madison, Wisconsin, USA, 2015.

6 CrysAlisPro, Rigaku Oxford Diffraction, 2024.

7 Palatinus, L.; Chapuis, G. *J. Appl. Cryst.* **2007**, *40*, 786.

8 Betteridge, P. W.; Carruthers, J. R.; Cooper, R. I.; Prout, K., Watkin, D. J. *J. Appl. Cryst.* **2003**, *36*, 1487.

9 Copies of NMR spectra

^{1}H NMR spectrum of **6a** ($CDCl_3$, 401 MHz)

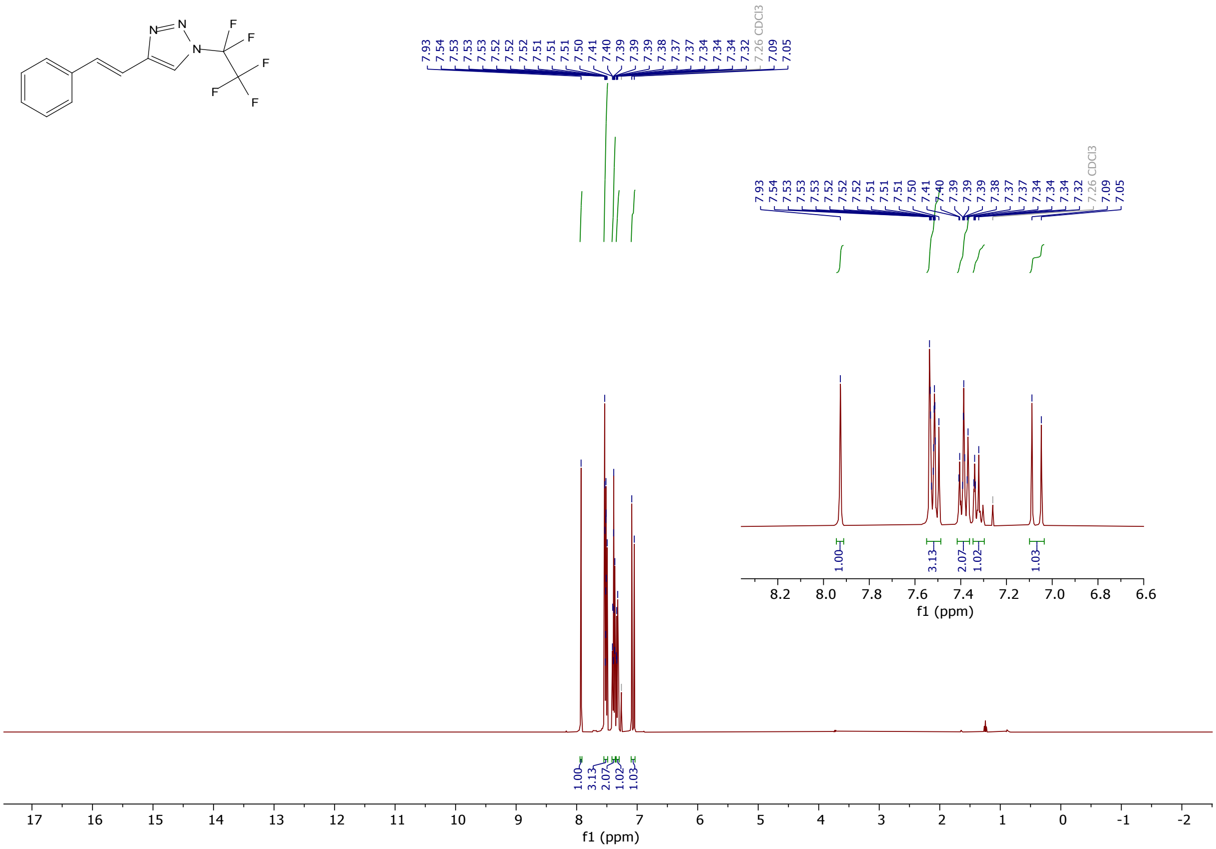

^{13}C NMR spectrum of **6a** ($CDCl_3$, 101 MHz)

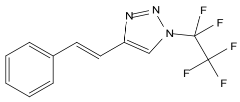

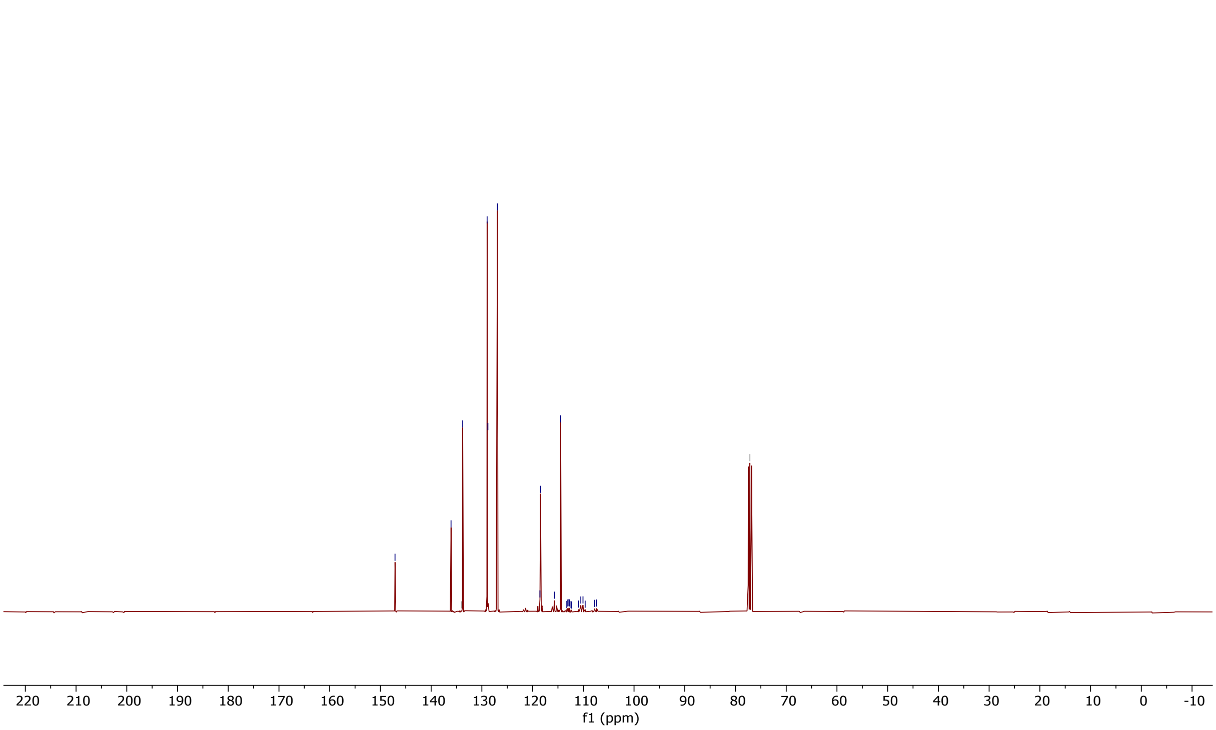

^{19}F NMR spectrum of **6a** ($CDCl_3$, 376 MHz)

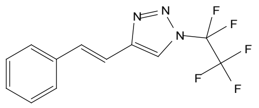

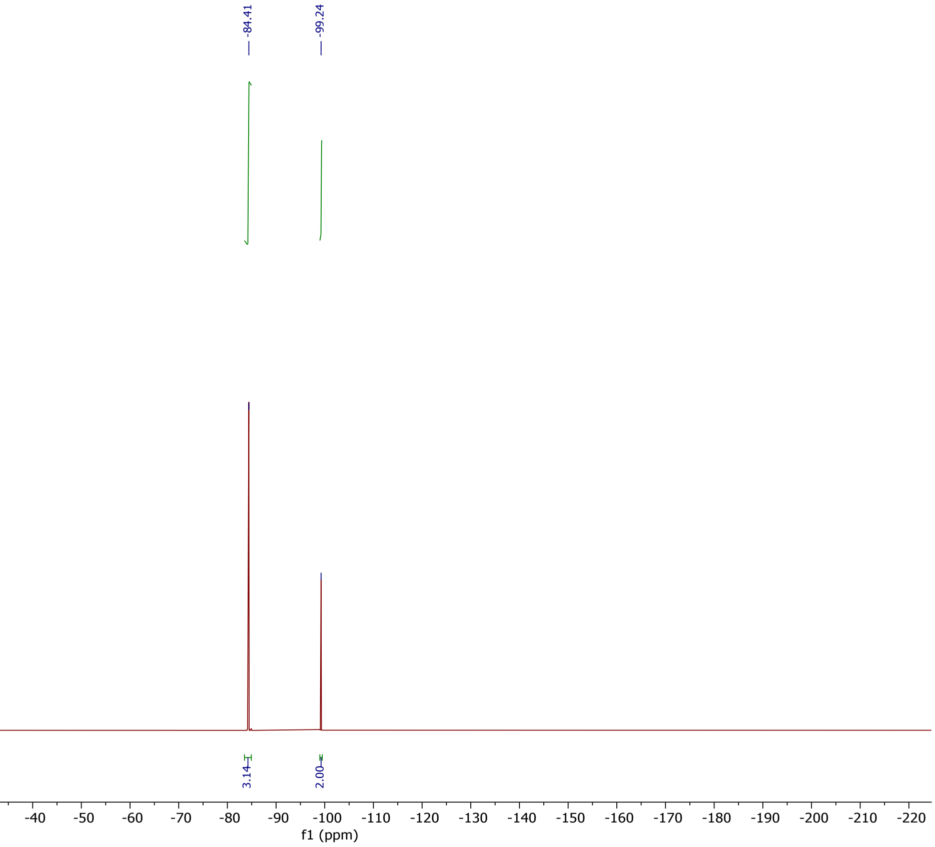

^{1}H NMR spectrum of **6b** ($CDCl_3$, 401 MHz)

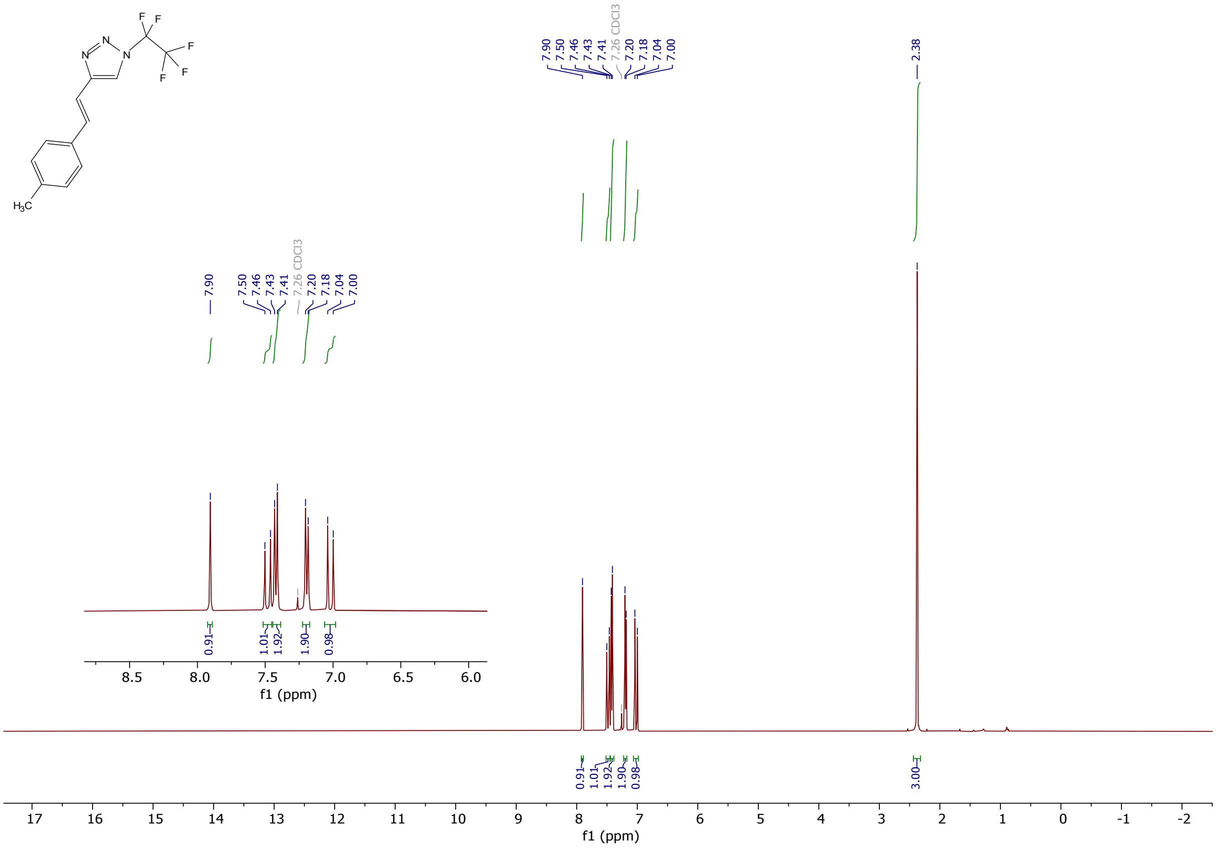

^{13}C NMR spectrum of **6b** ($CDCl_3$, 101 MHz)

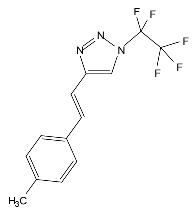

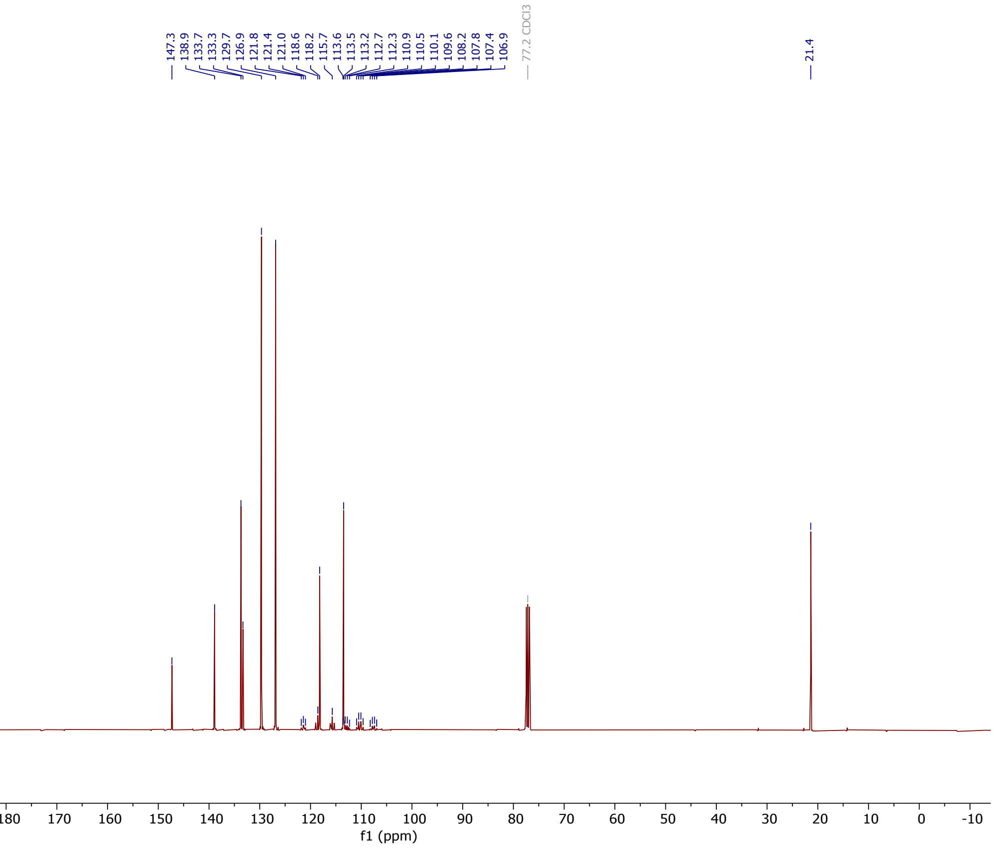

^{19}F NMR spectrum of **6b** ($CDCl_3$, 376 MHz)

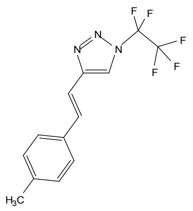

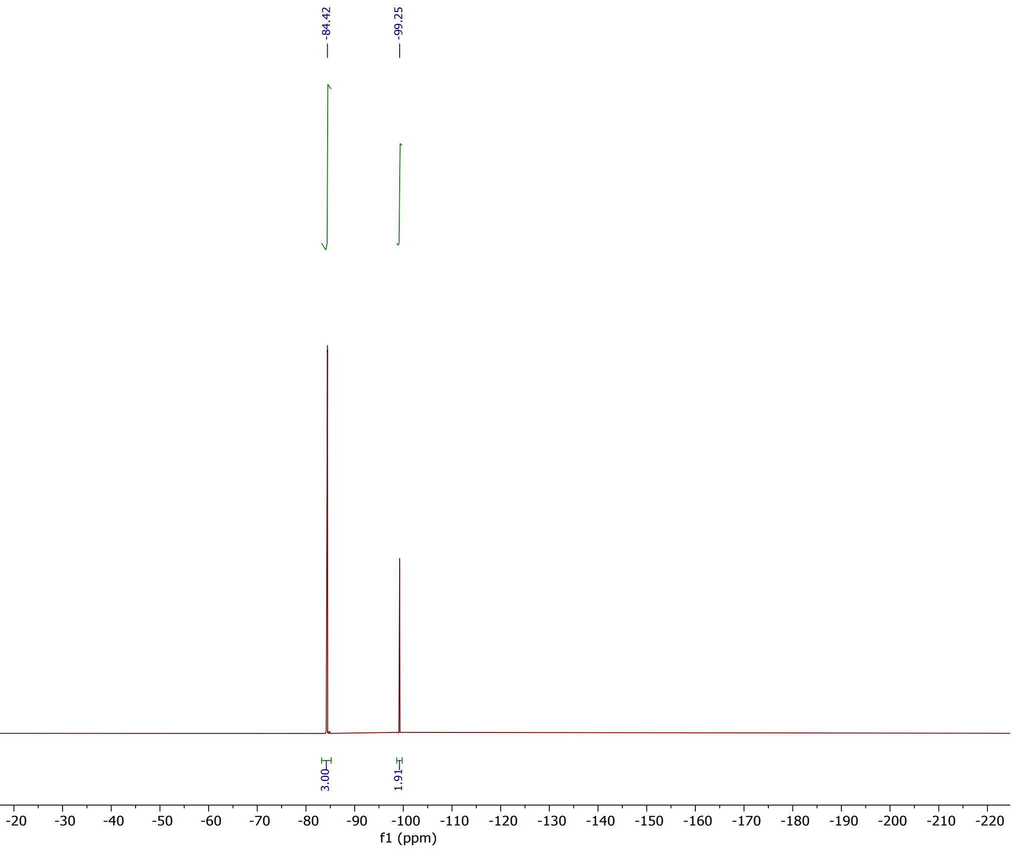

^{1}H NMR spectrum of **6c** ($CDCl_3$, 401 MHz)

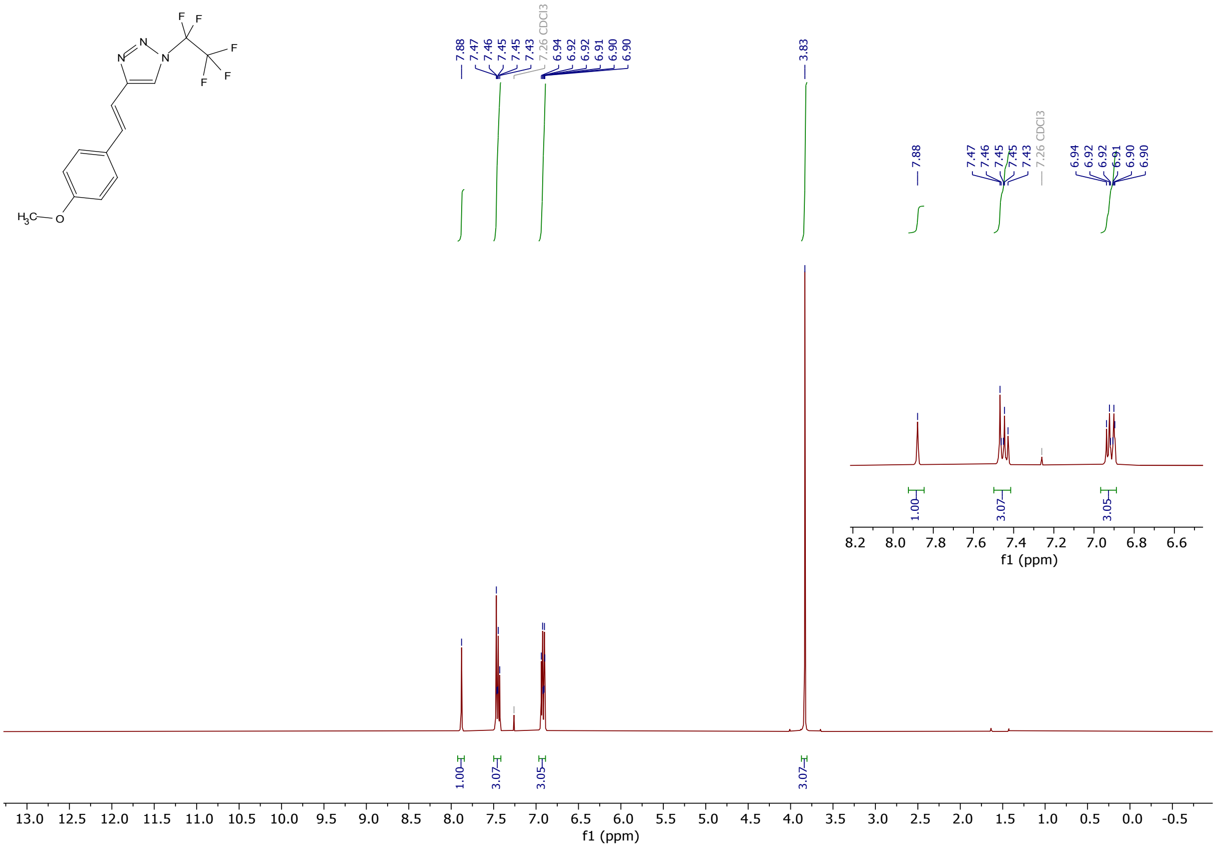

^{13}C NMR spectrum of **6c** ($CDCl_3$, 101 MHz)

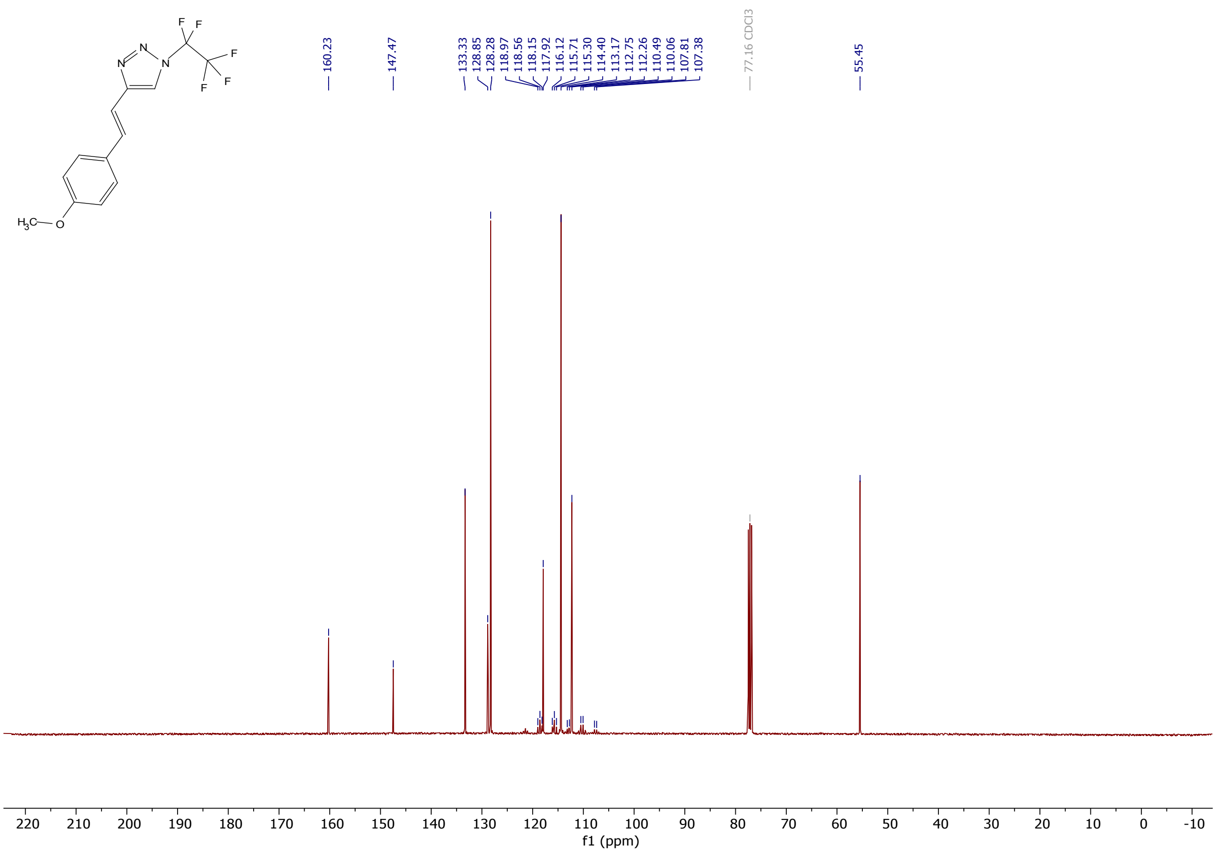

^{19}F NMR spectrum of **6c** ($CDCl_3$, 376 MHz)

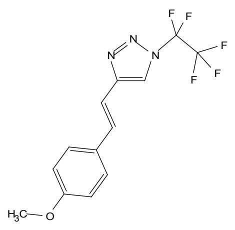

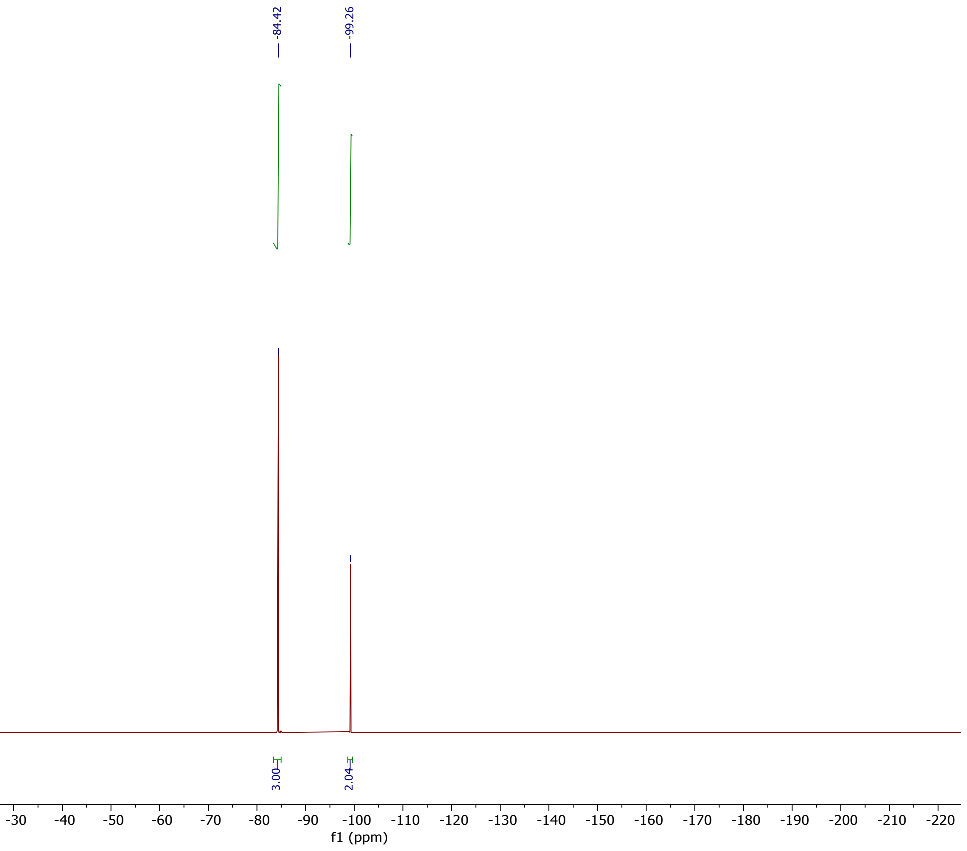

^{1}H NMR spectrum of **6d** ($CDCl_3$, 401 MHz)

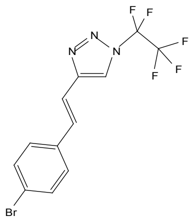

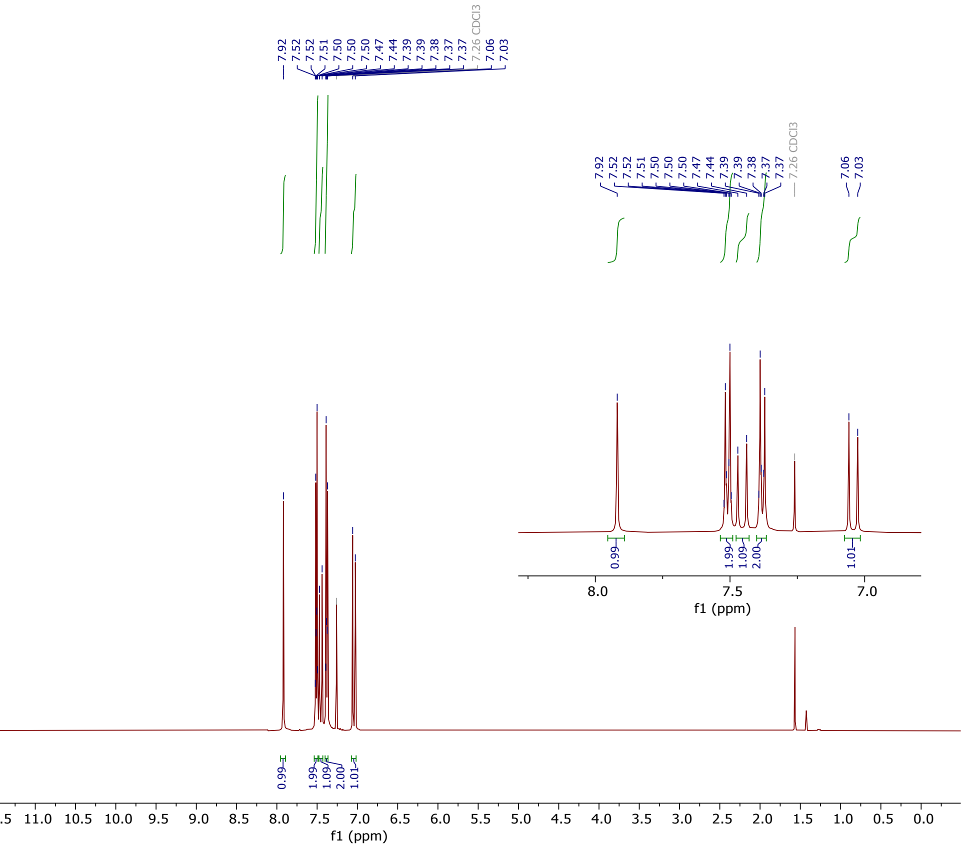

^{13}C NMR spectrum of **6d** ($CDCl_3$, 101 MHz)

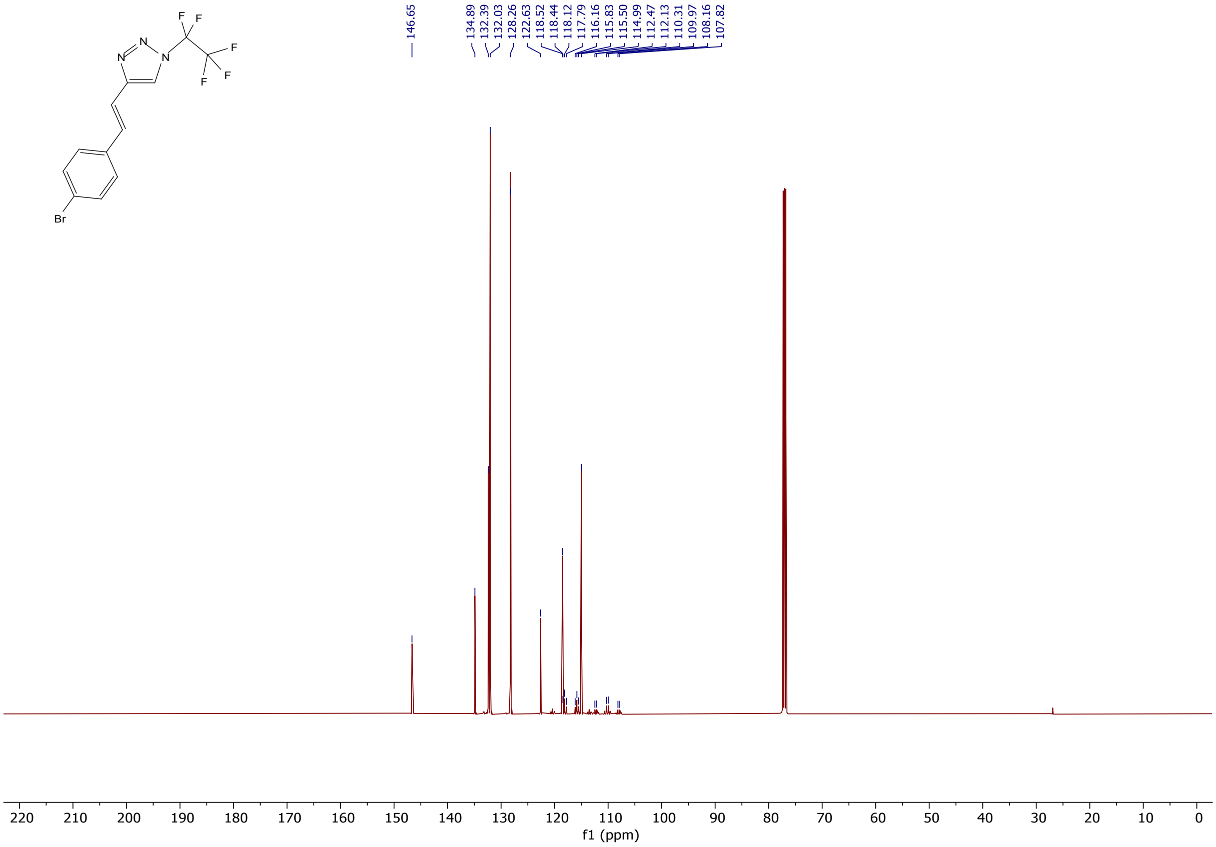

^{19}F NMR spectrum of **6d** ($CDCl_3$, 376 MHz)

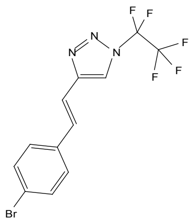

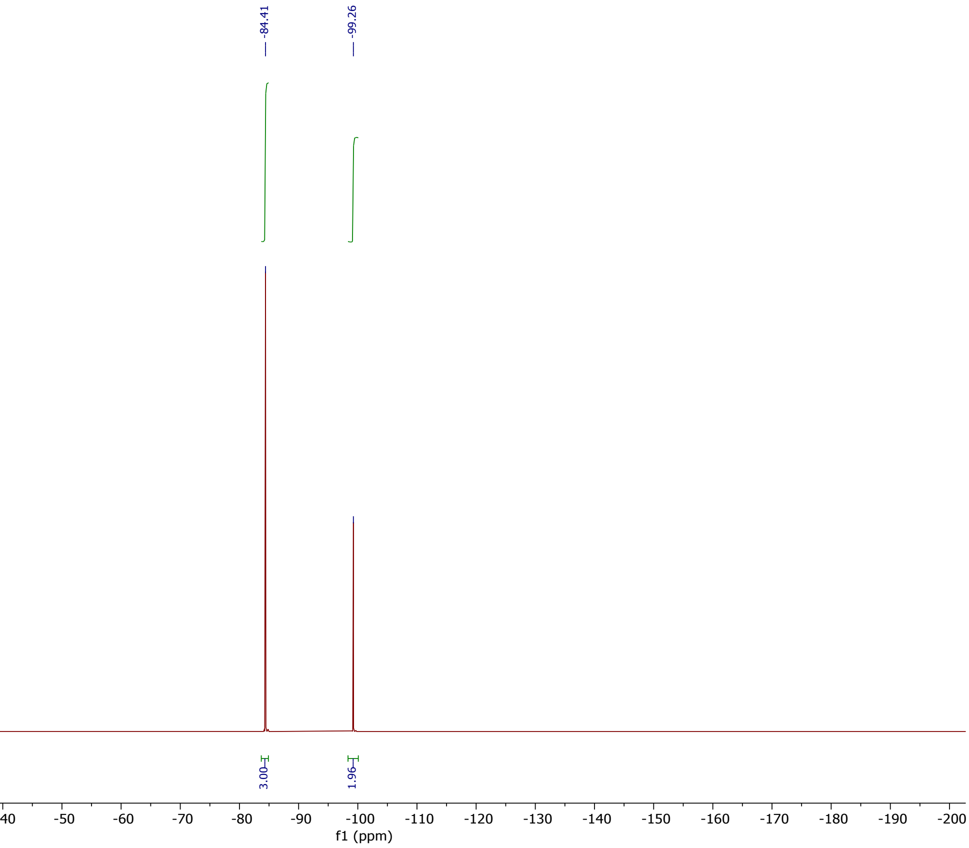

^1H NMR spectrum of **6e** ($CDCl_3$, 401 MHz)

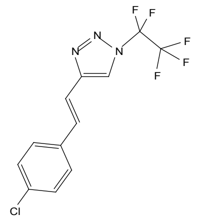

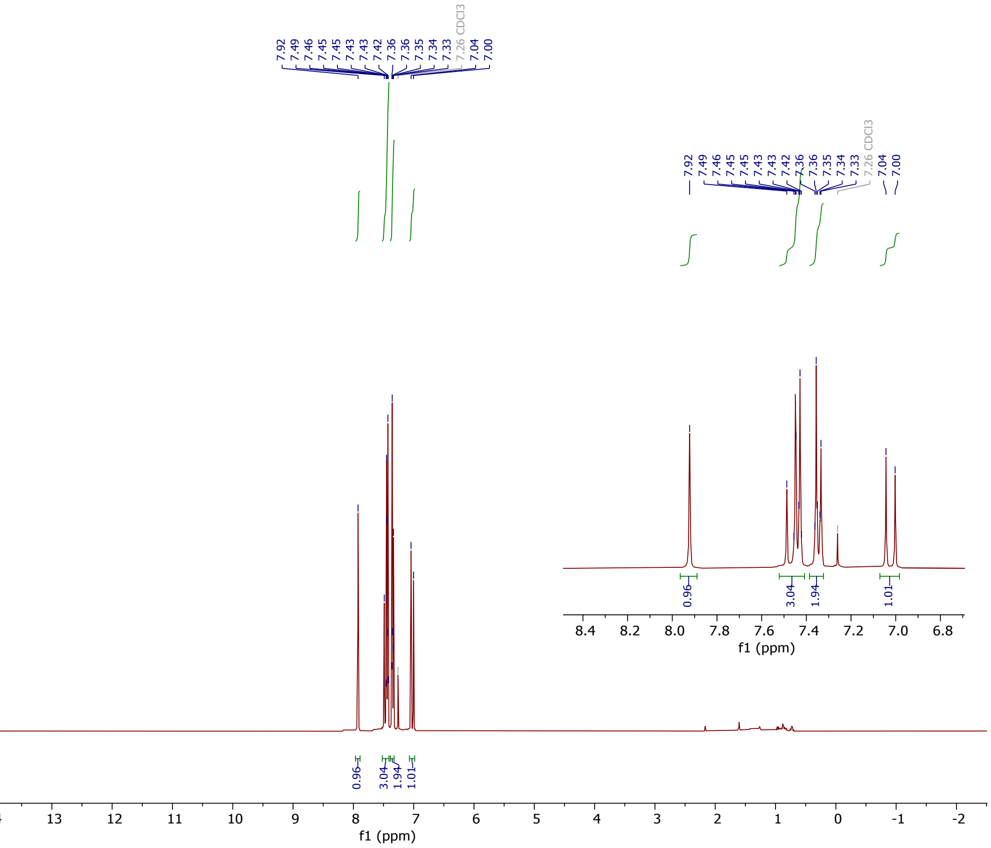

^{13}C NMR spectrum of **6e** ($CDCl_3$, 101 MHz)

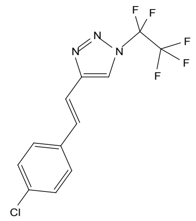

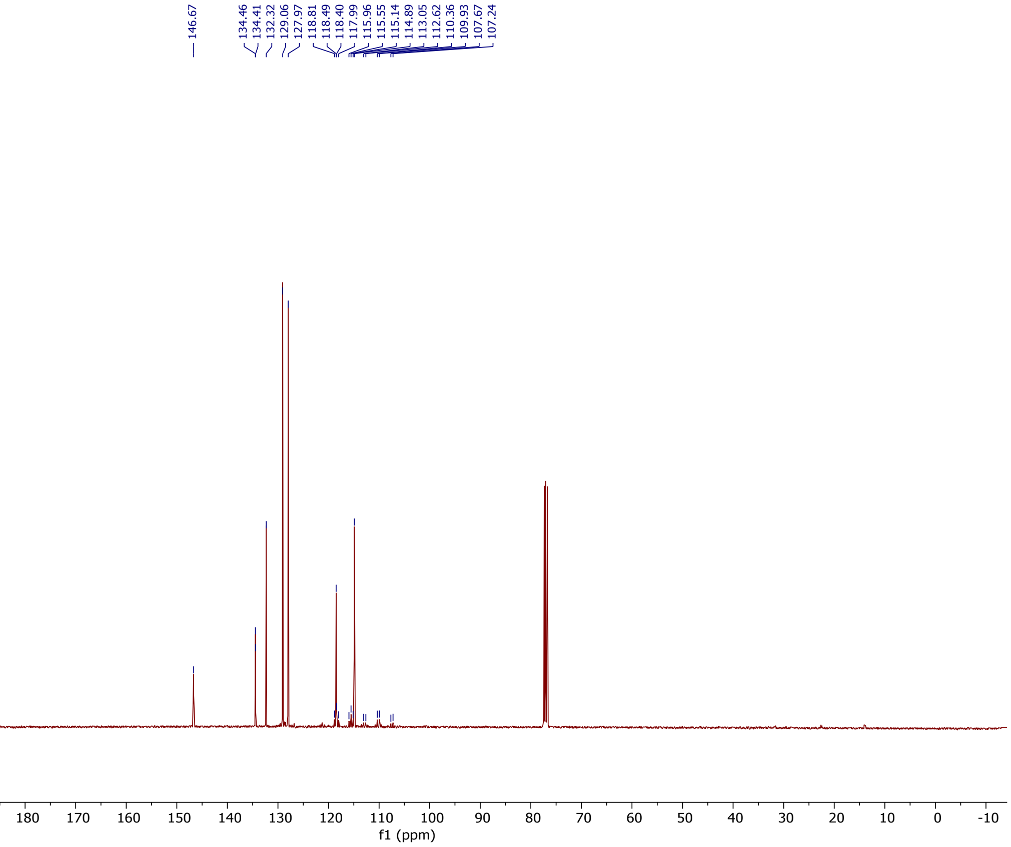

^{19}F NMR spectrum of **6e** ($CDCl_3$, 376 MHz)

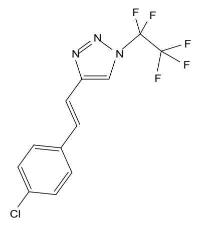

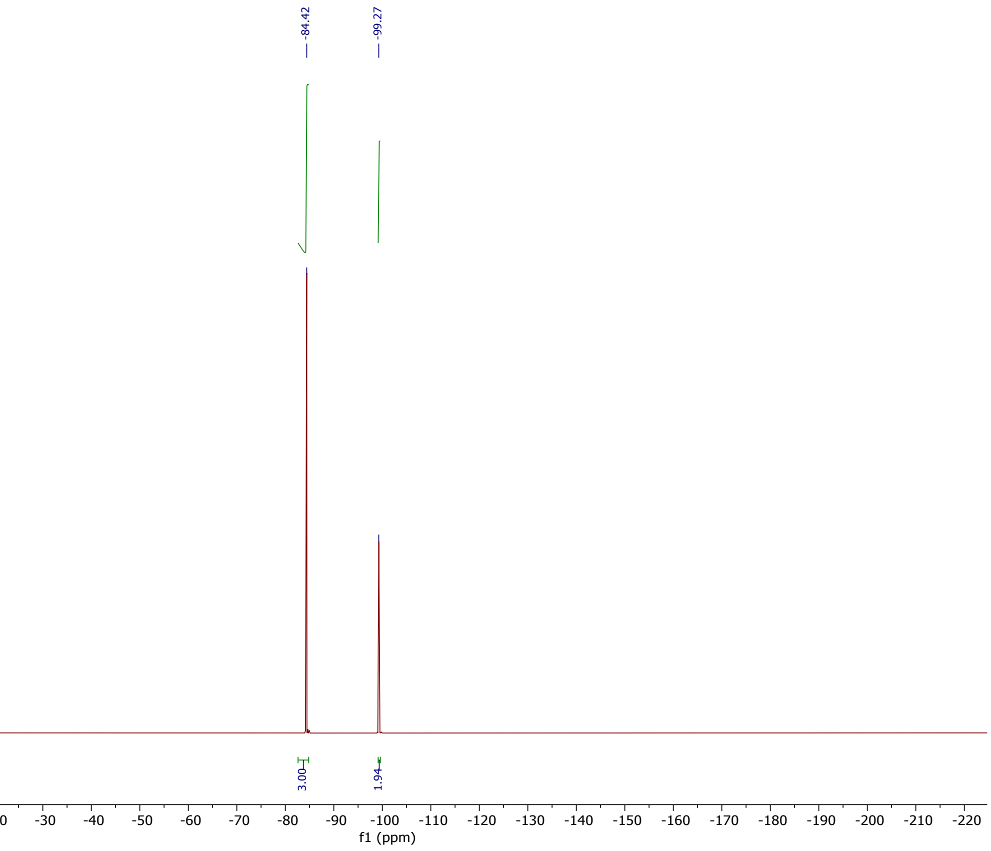

^{1}H NMR spectrum of **6f** ($CDCl_3$, 401 MHz)

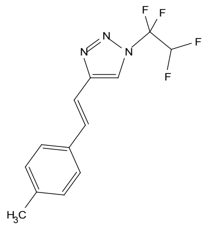

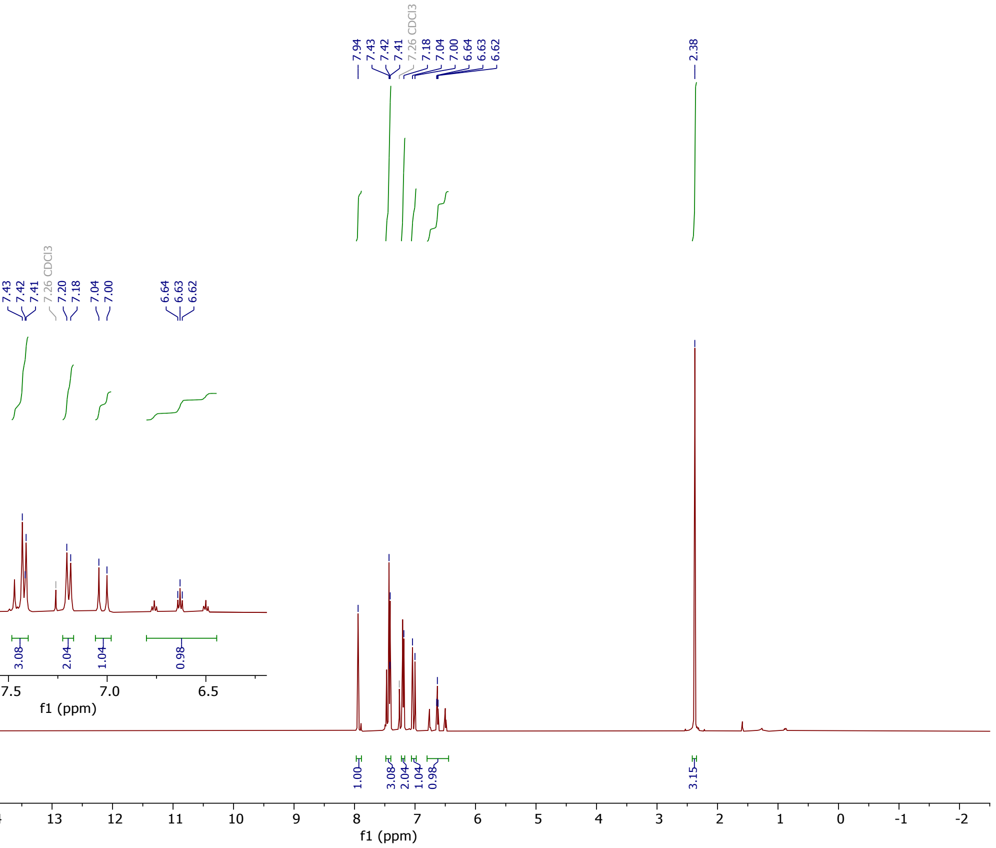

^{13}C NMR spectrum of **6f** ($CDCl_3$, 101 MHz)

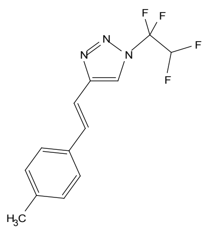

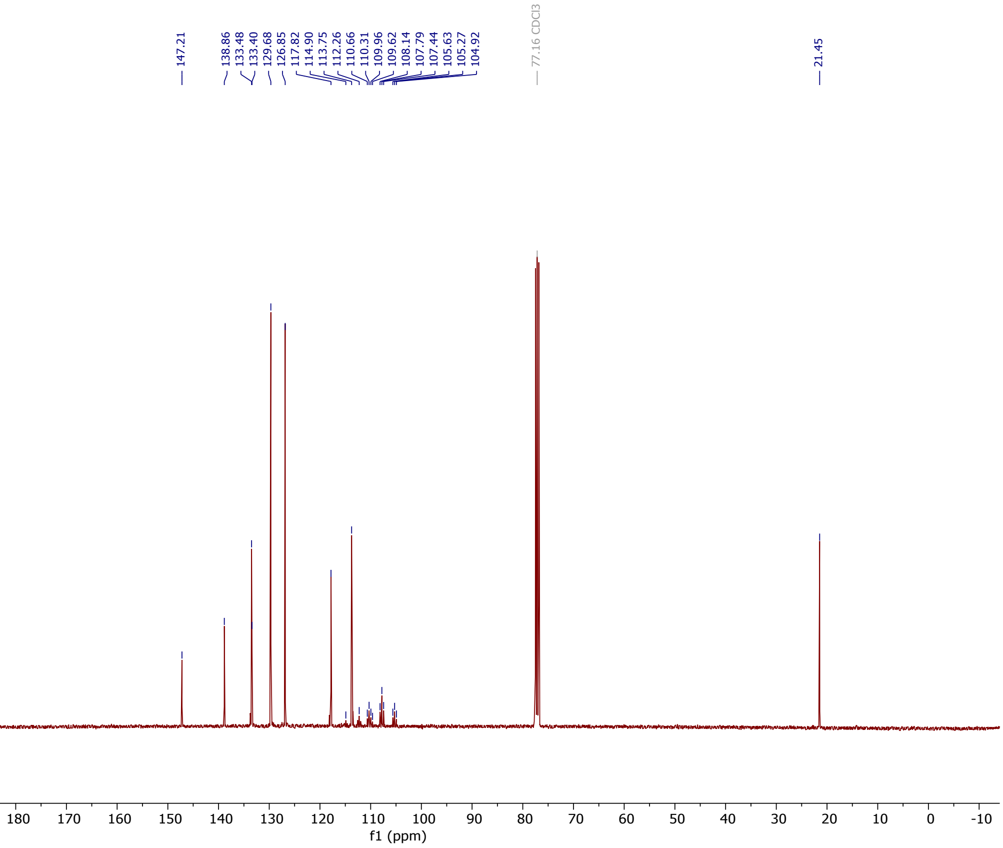

^{19}F NMR spectrum of **6f** (CDCl$_3$, 376 MHz)

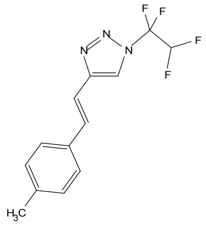

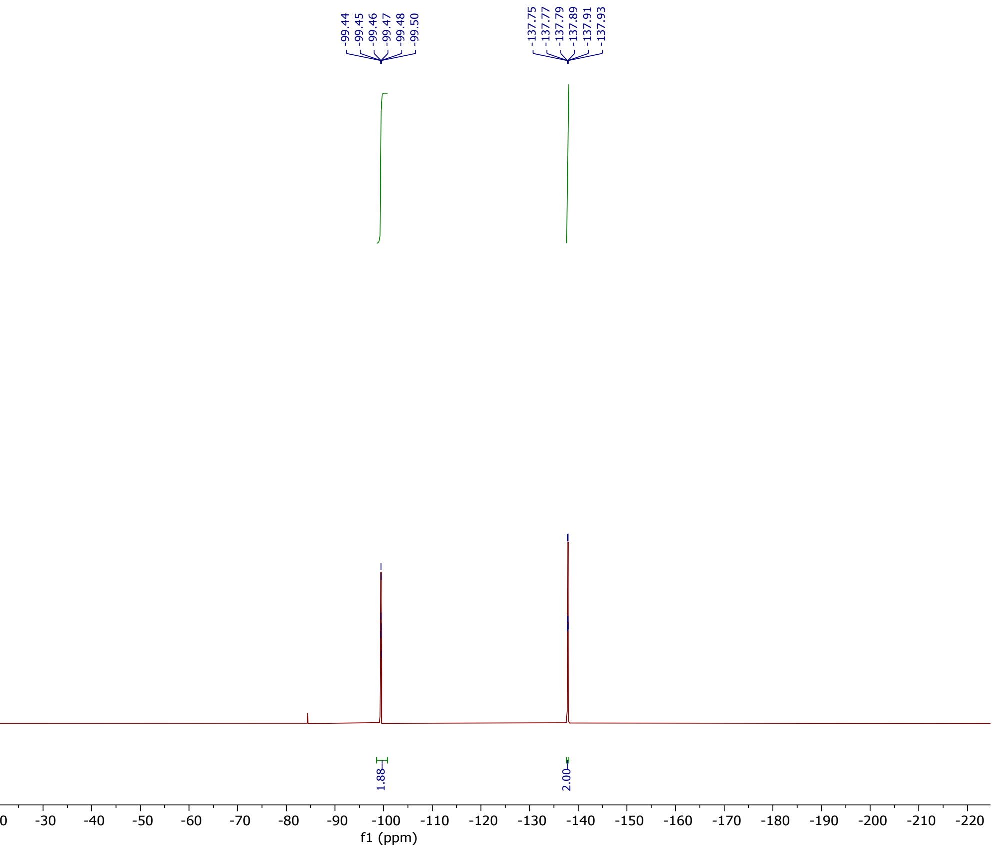

^{1}H NMR spectrum of **6g** ($CDCl_3$, 401 MHz)

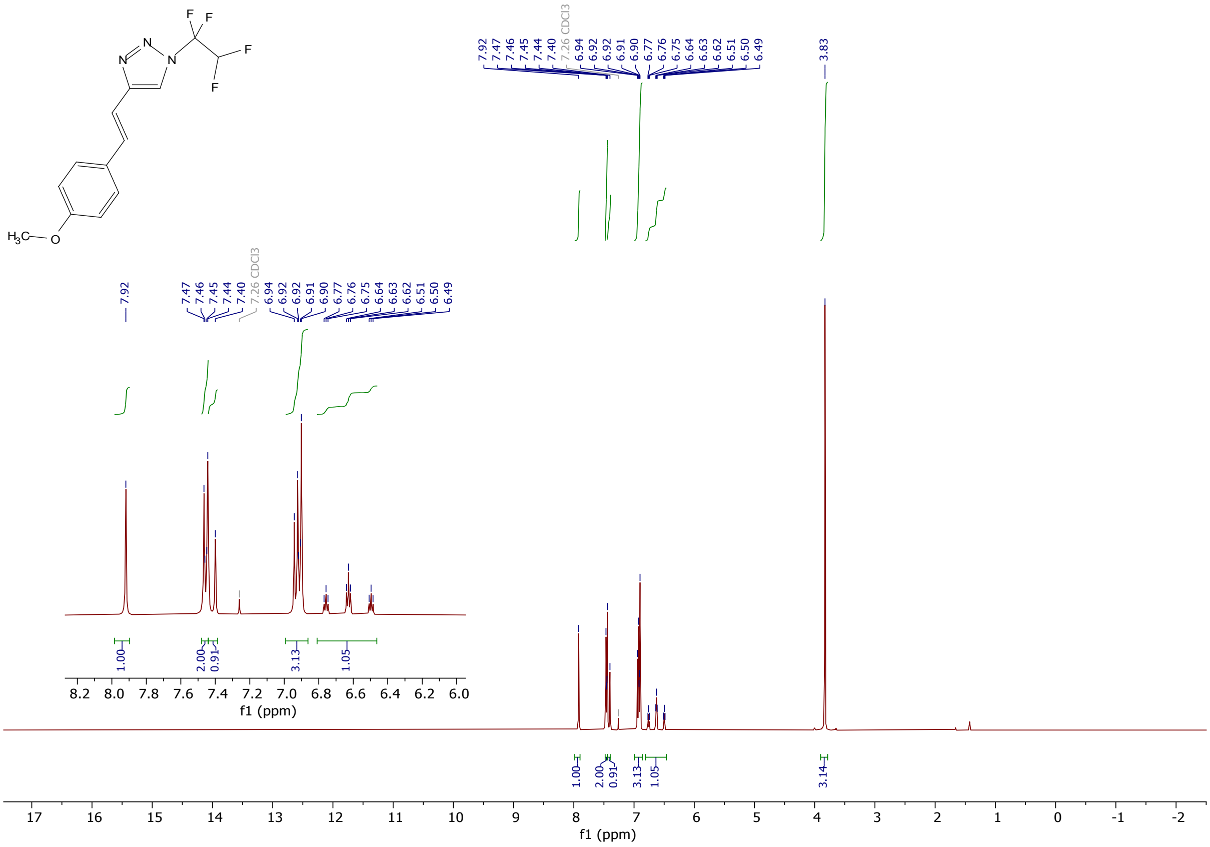

^{13}C NMR spectrum of **6g** ($CDCl_3$, 101 MHz)

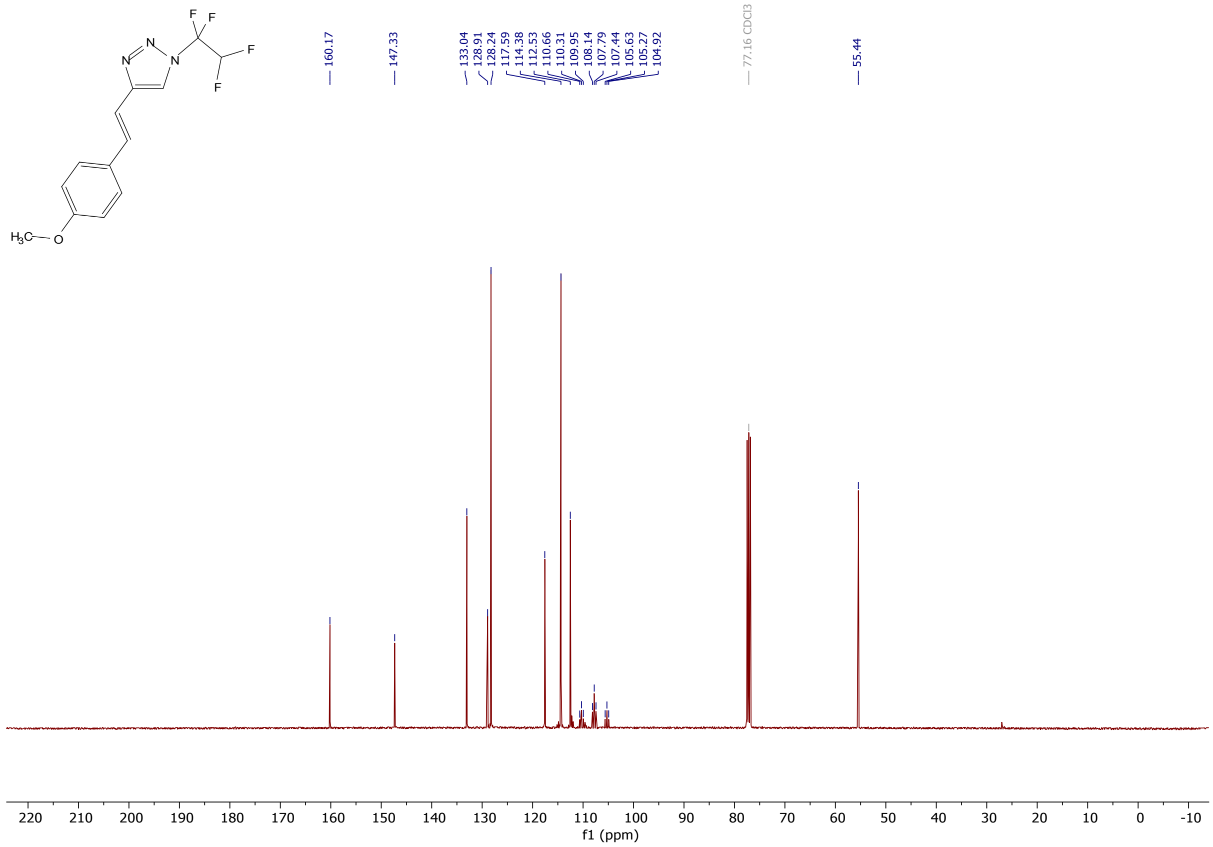

^{19}F NMR spectrum of **6g** ($CDCl_3$, 376 MHz)

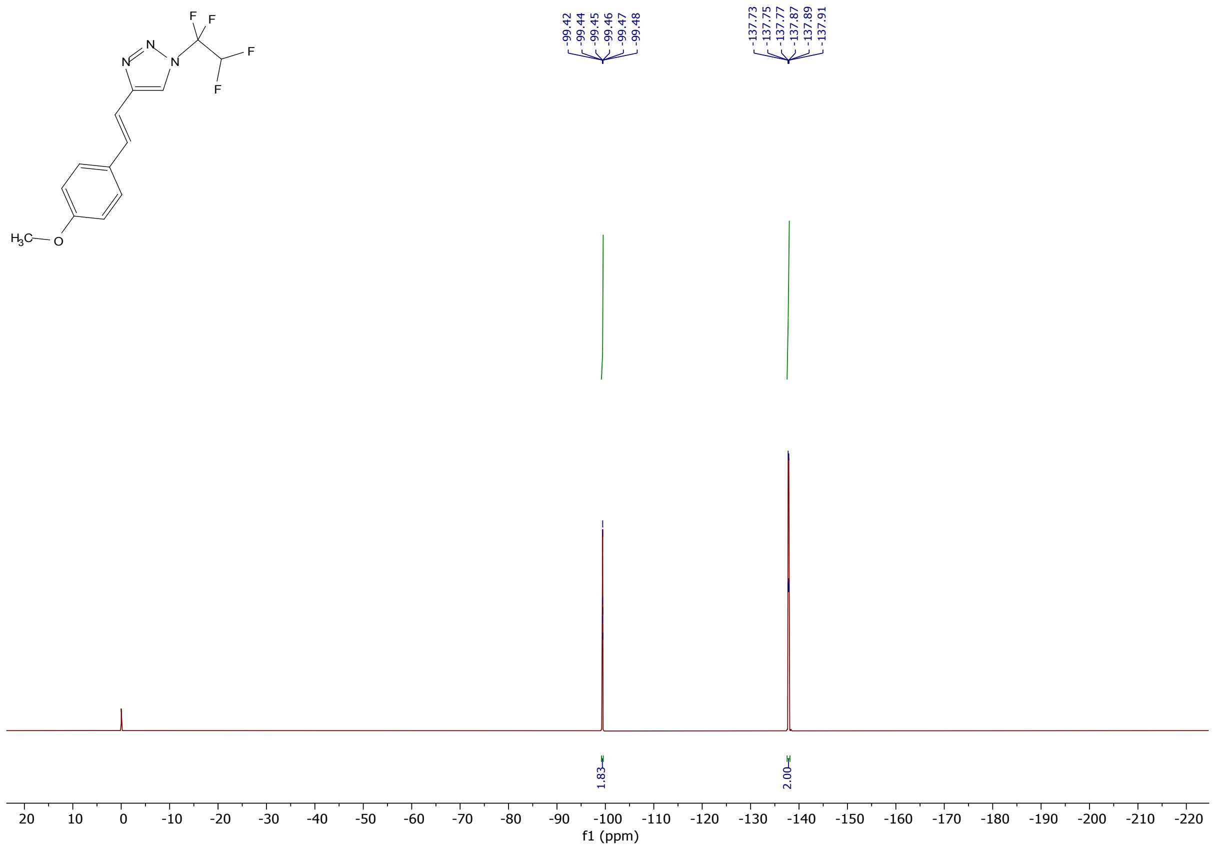

^{1}H NMR spectrum of **6h** ($CDCl_3$, 401 MHz)

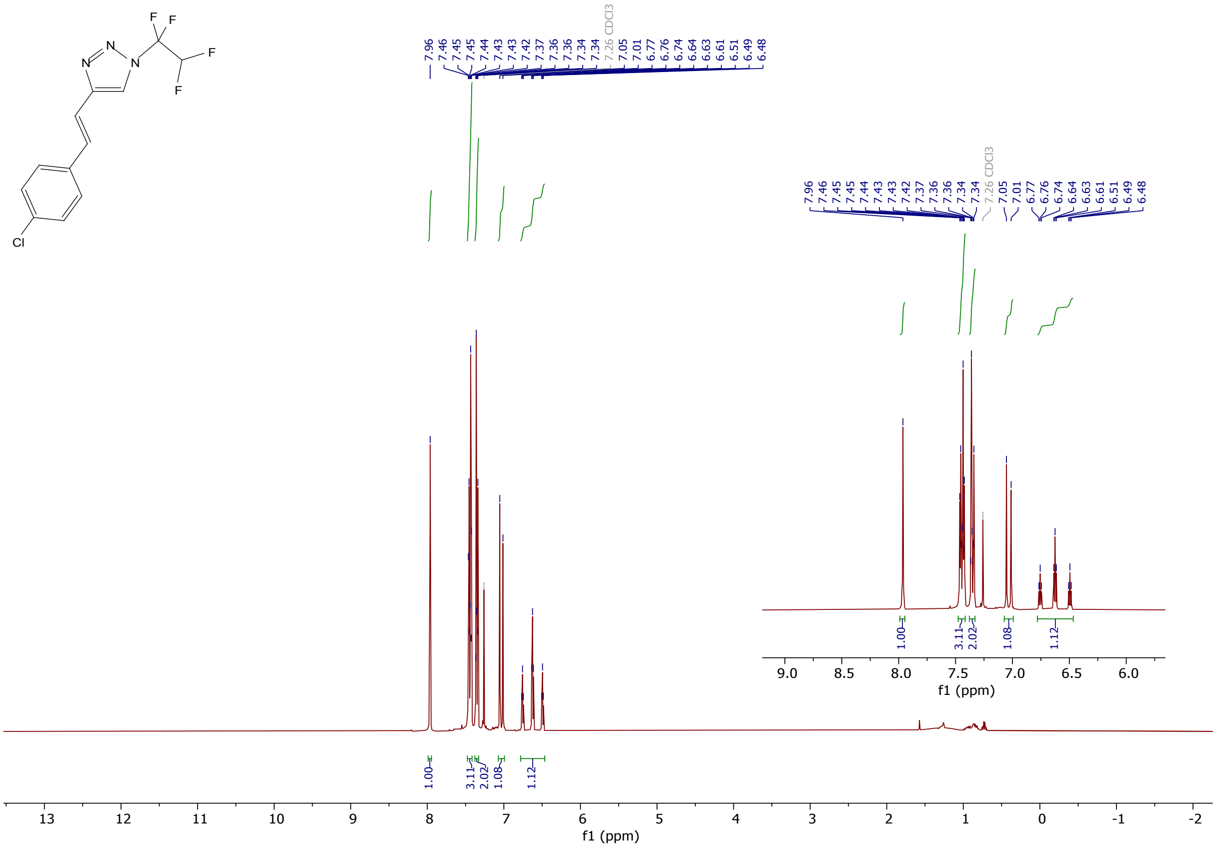

^{13}C NMR spectrum of **6h** ($CDCl_3$, 101 MHz)

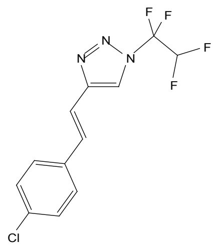

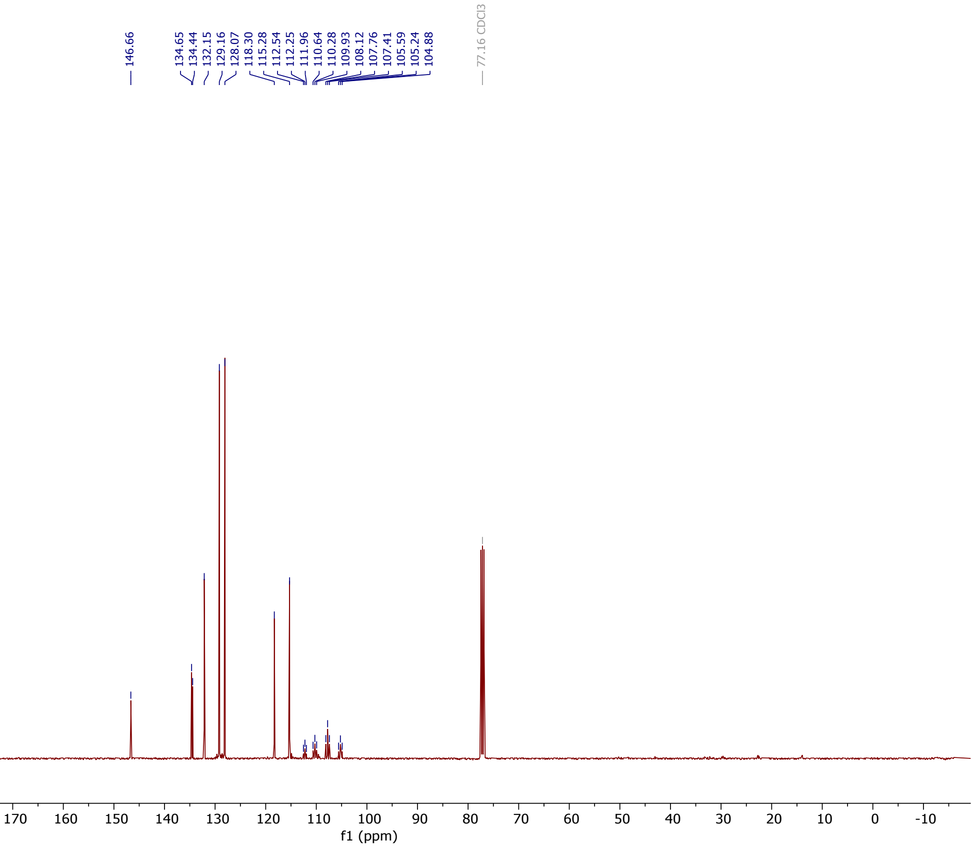

^{19}F NMR spectrum of **6h** ($CDCl_3$, 376 MHz)

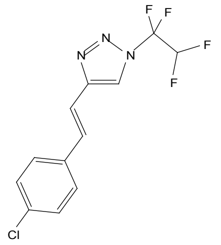

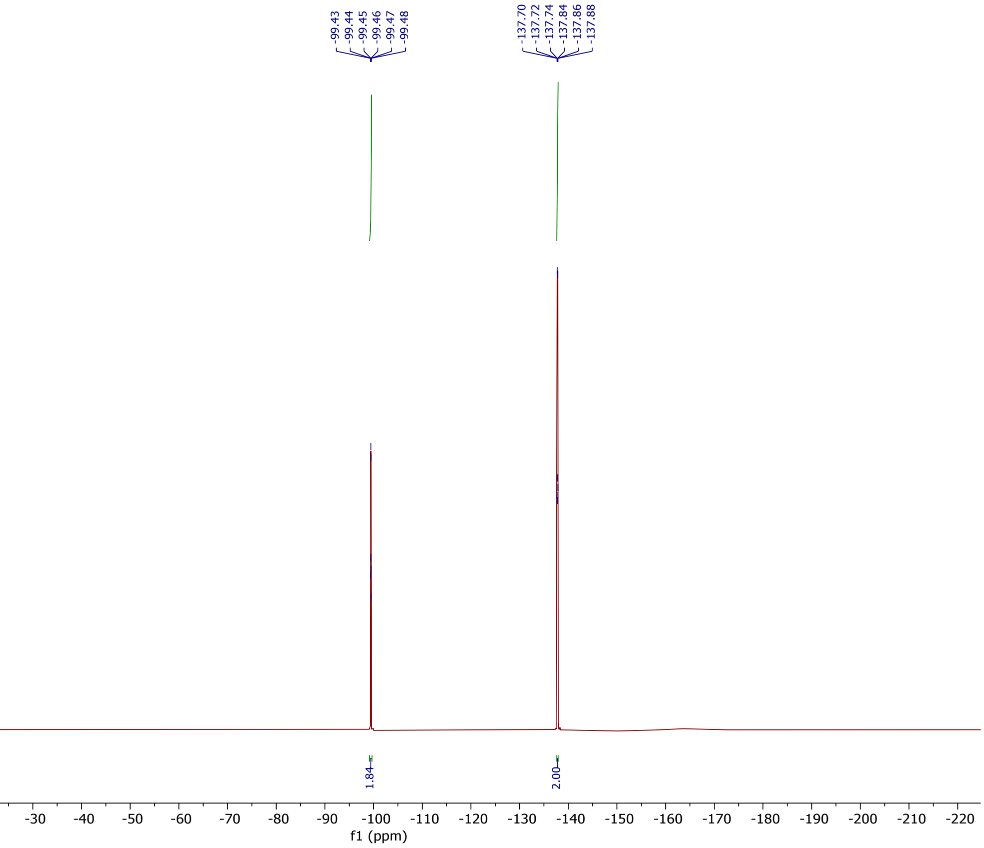

^{1}H NMR spectrum of **6i** ($CDCl_3$, 401 MHz)

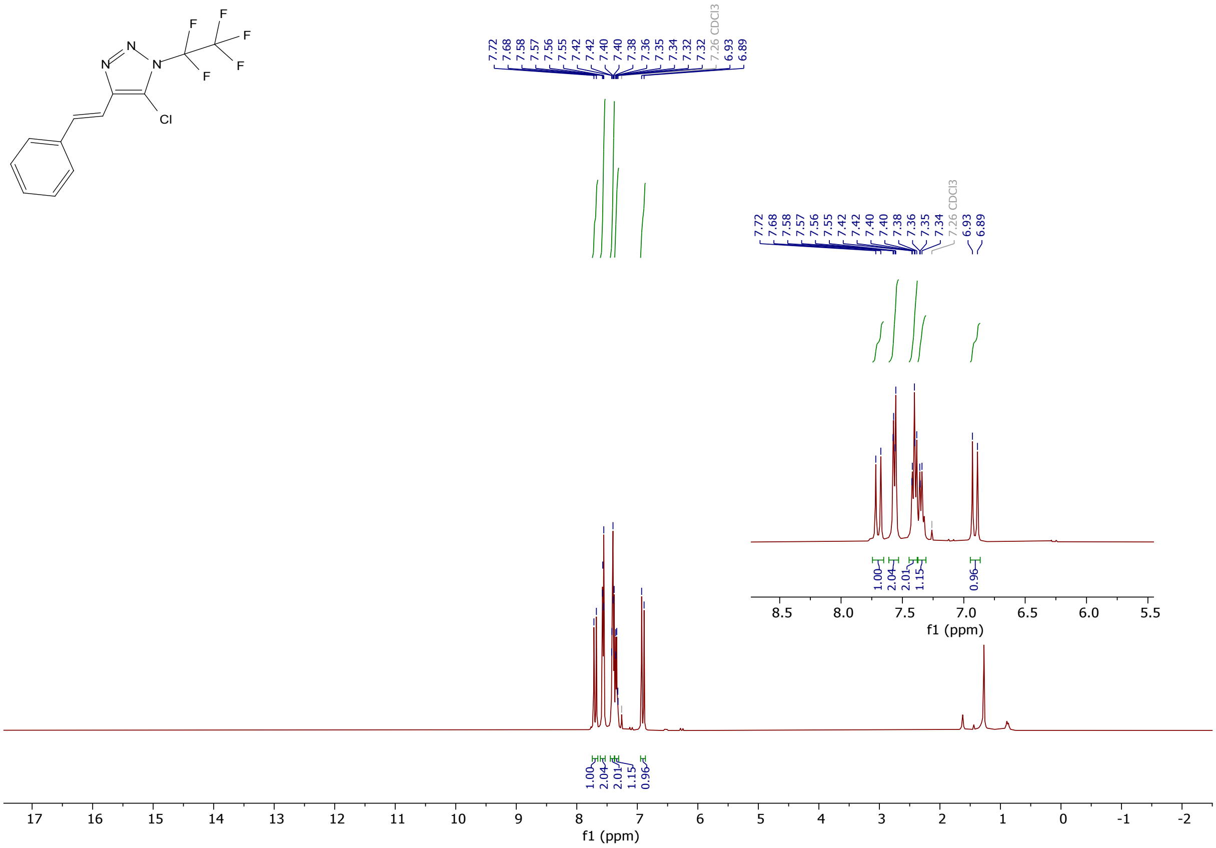

^{13}C NMR spectrum of **6i** ($CDCl_3$, 101 MHz)

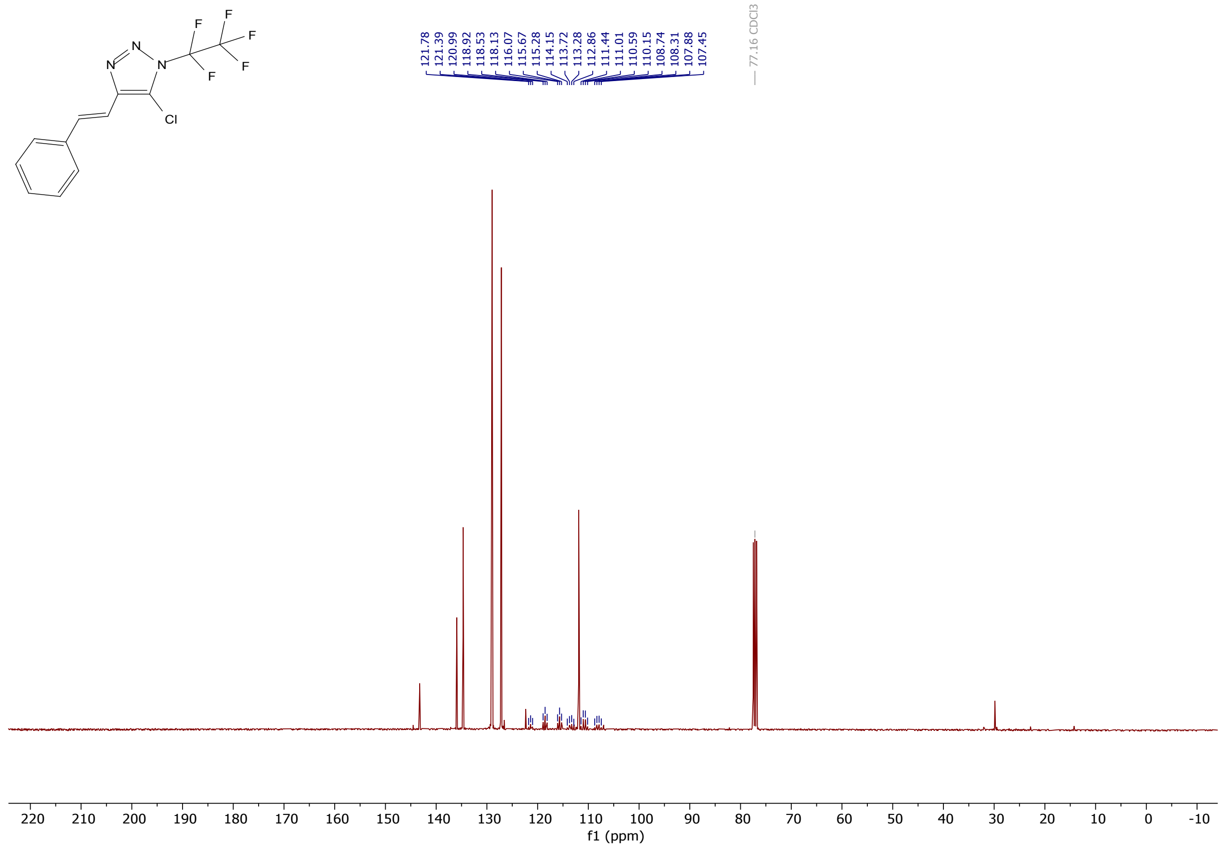

^{19}F NMR spectrum of **6i** ($CDCl_3$, 376 MHz)

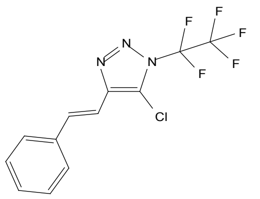

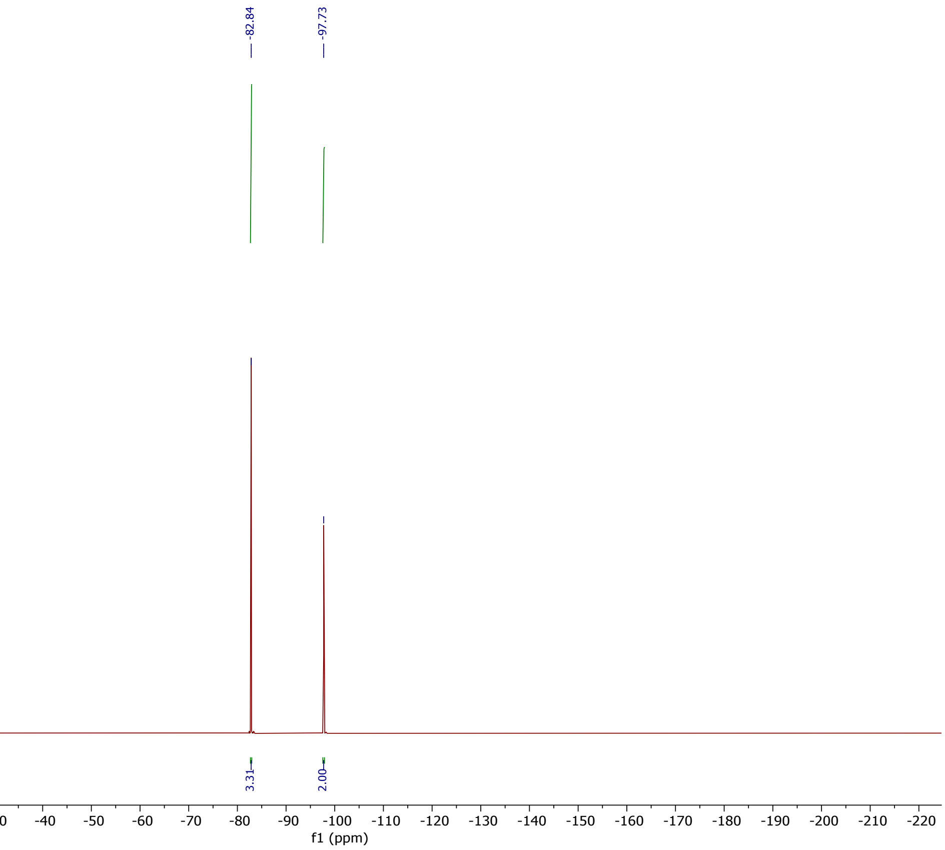

^{1}H NMR spectrum of **6l** ($CDCl_3$, 401 MHz)

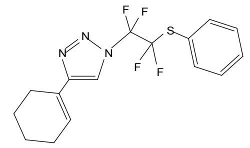

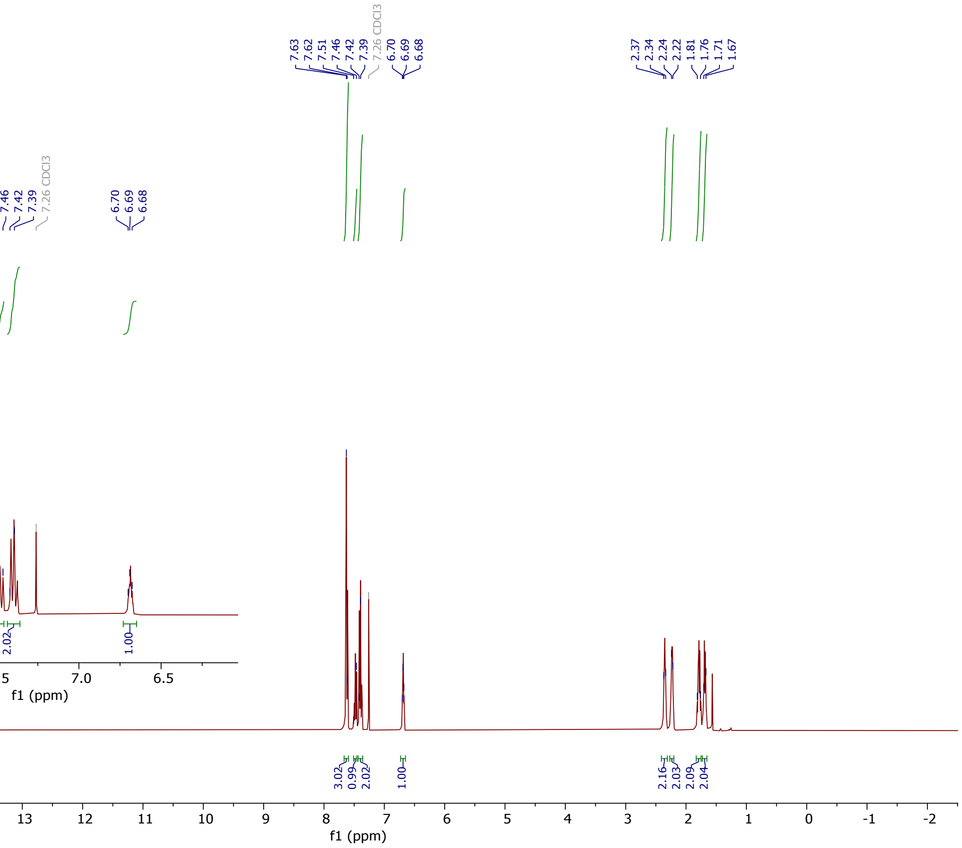

^{13}C NMR spectrum of **6l** ($CDCl_3$, 101 MHz)

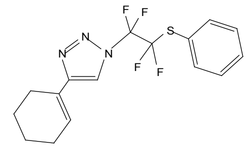

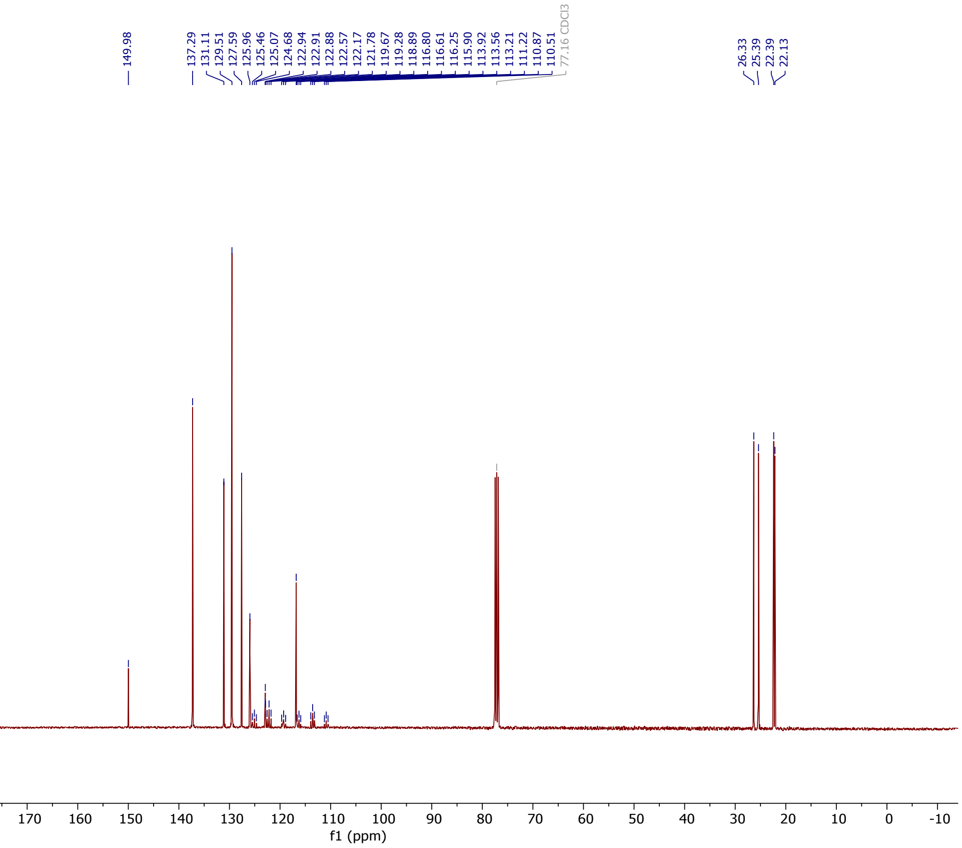

^{19}F NMR spectrum of **6l** ($CDCl_3$, 376 MHz)

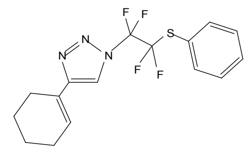

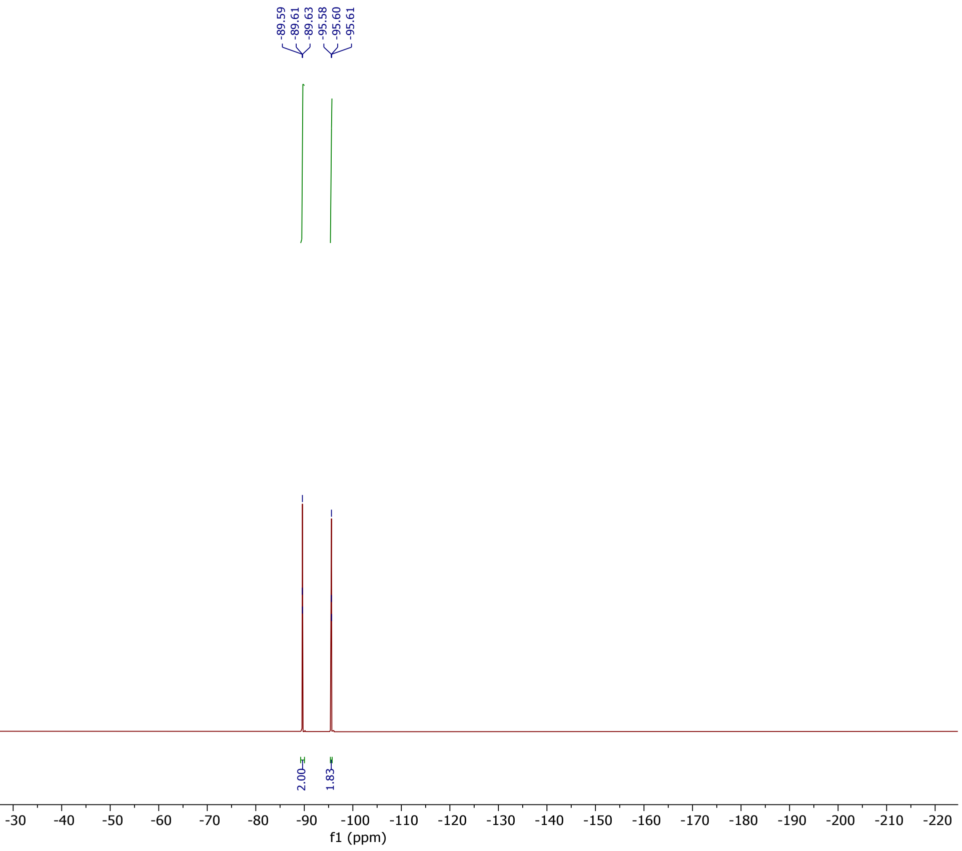

^{1}H NMR spectrum of **6m** ($CDCl_3$, 401 MHz)

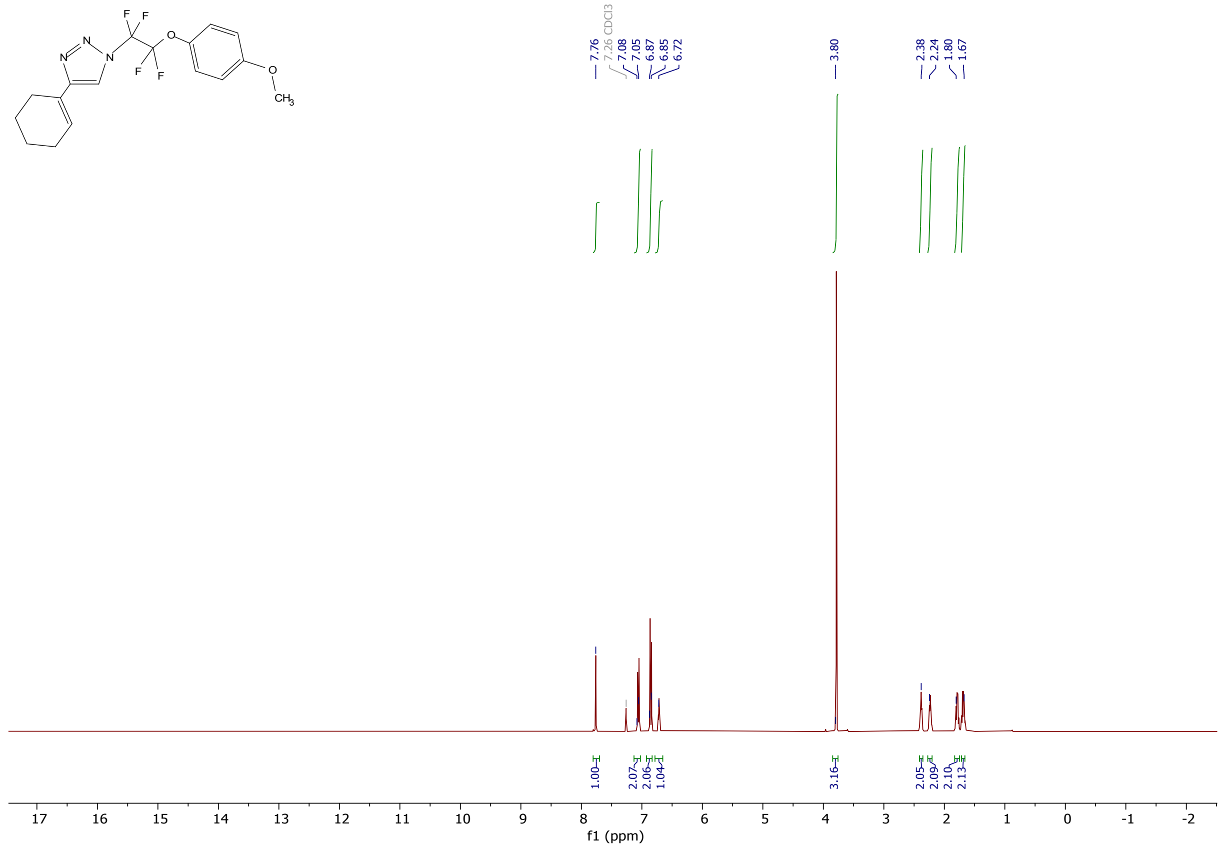

^{13}C NMR spectrum of **6m** ($CDCl_3$, 101 MHz)

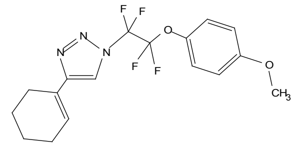

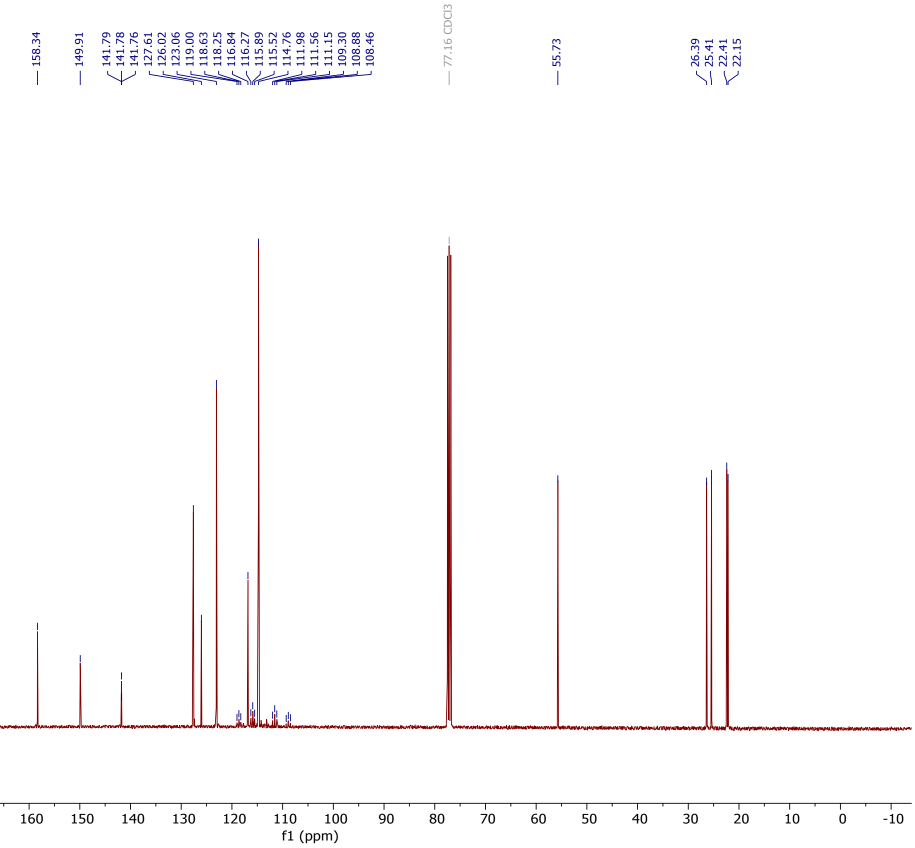

^{19}F NMR spectrum of **6m** ($CDCl_3$, 376 MHz)

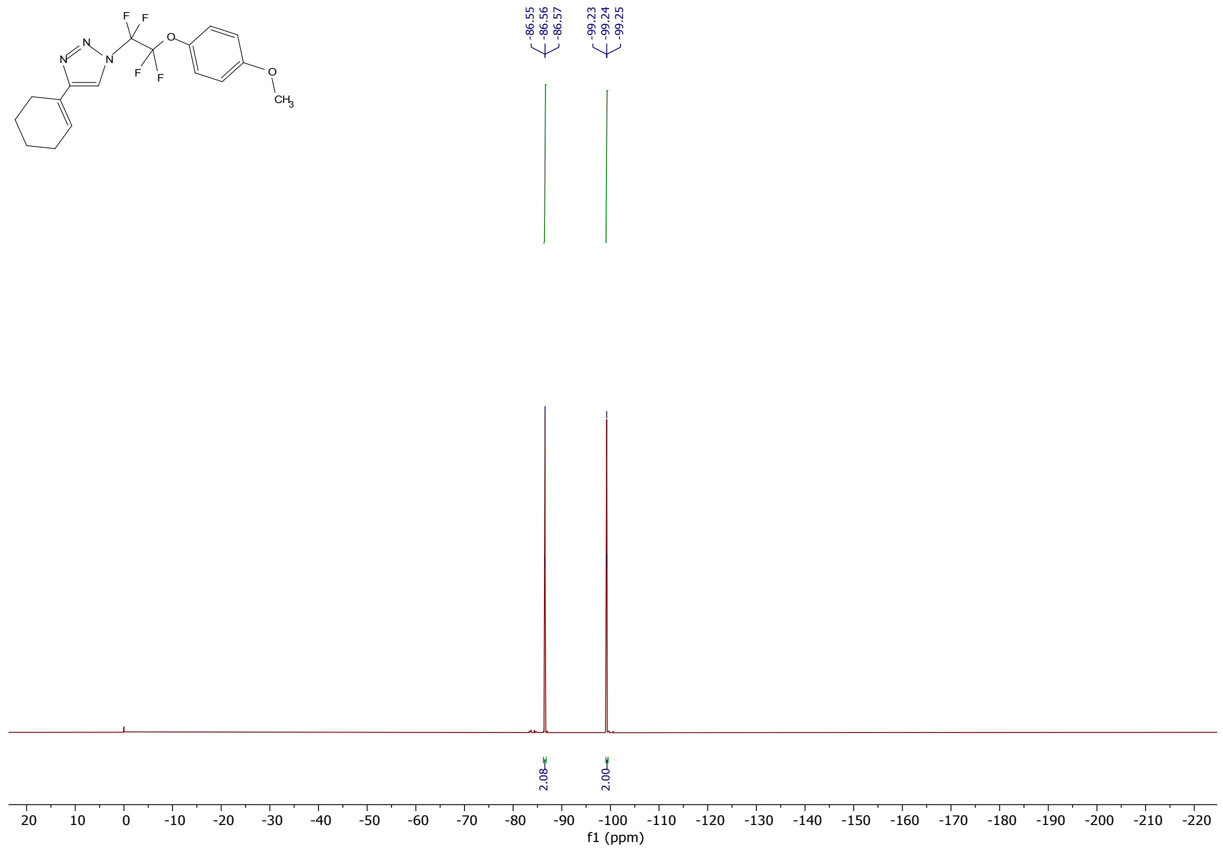

^{1}H NMR spectrum of **6p** ($CDCl_3$, 401 MHz)

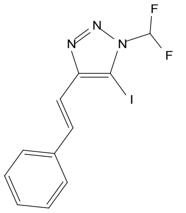

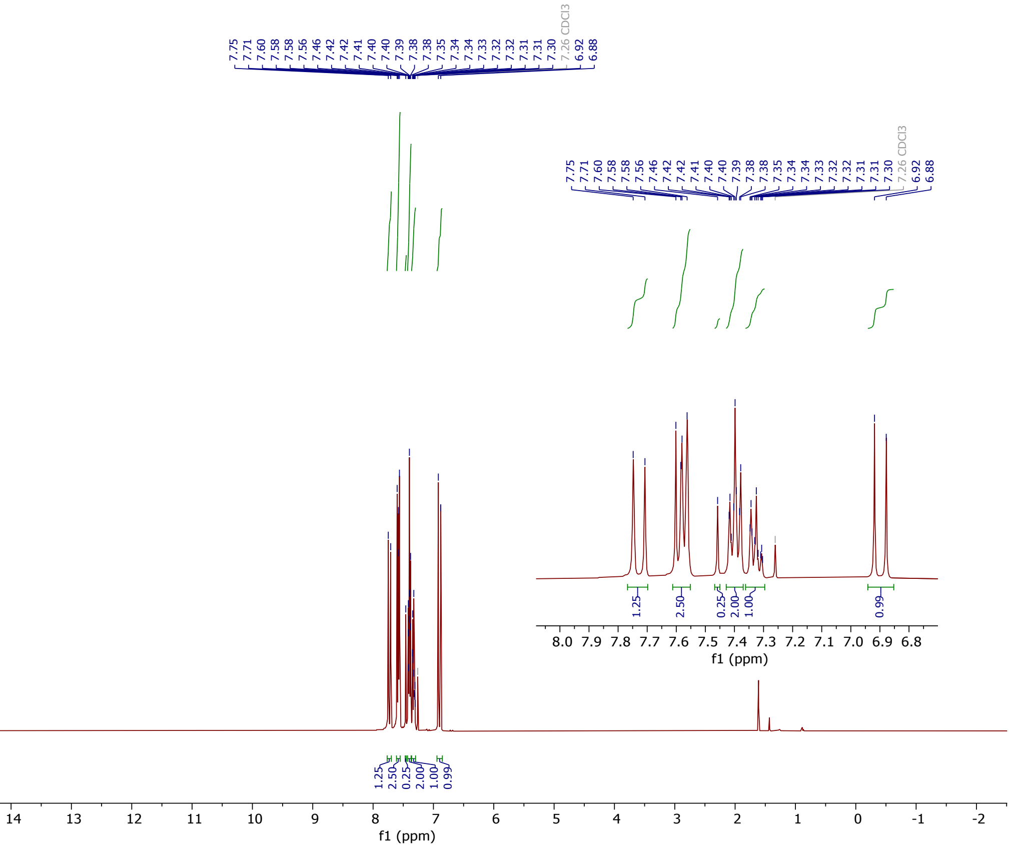

^{13}C NMR spectrum of **6p** ($CDCl_3$, 101 MHz)

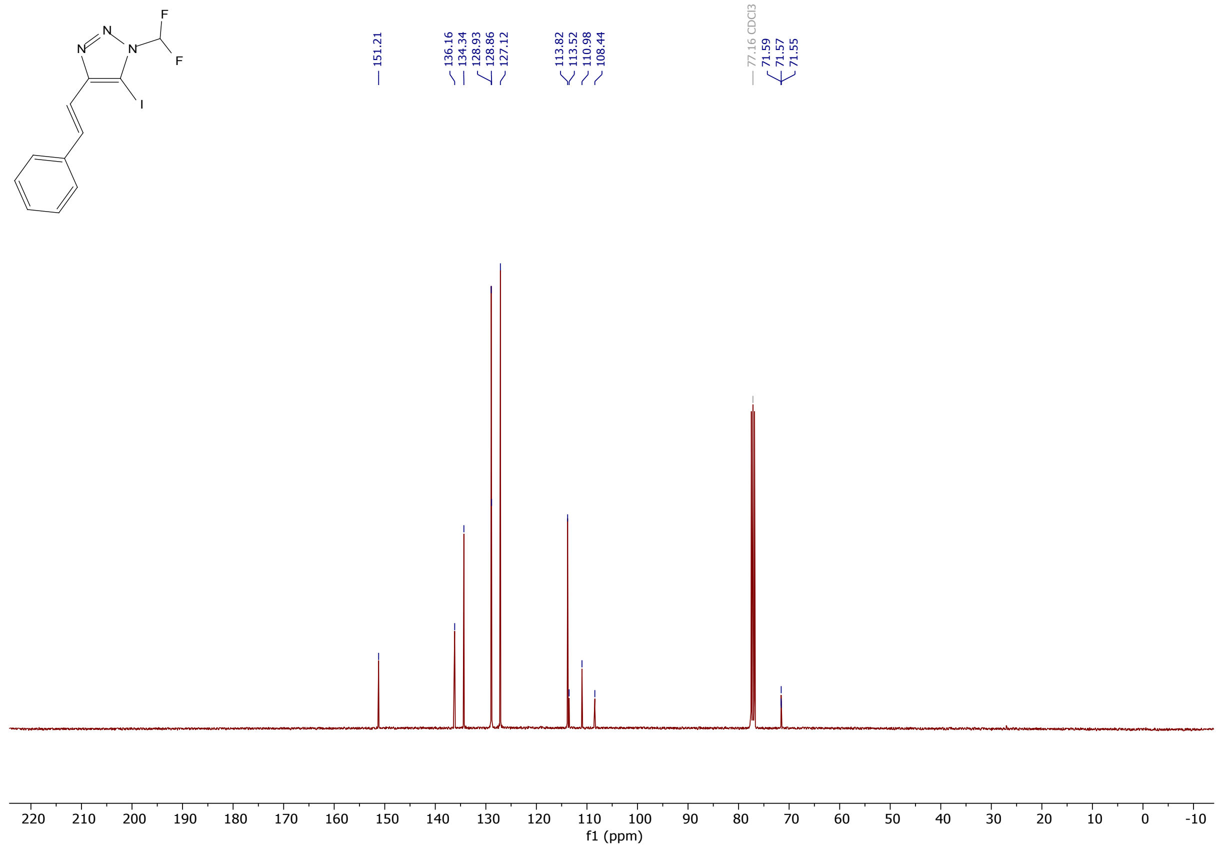

^{19}F NMR spectrum of **6p** ($CDCl_3$, 376 MHz)

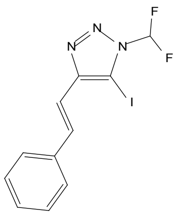

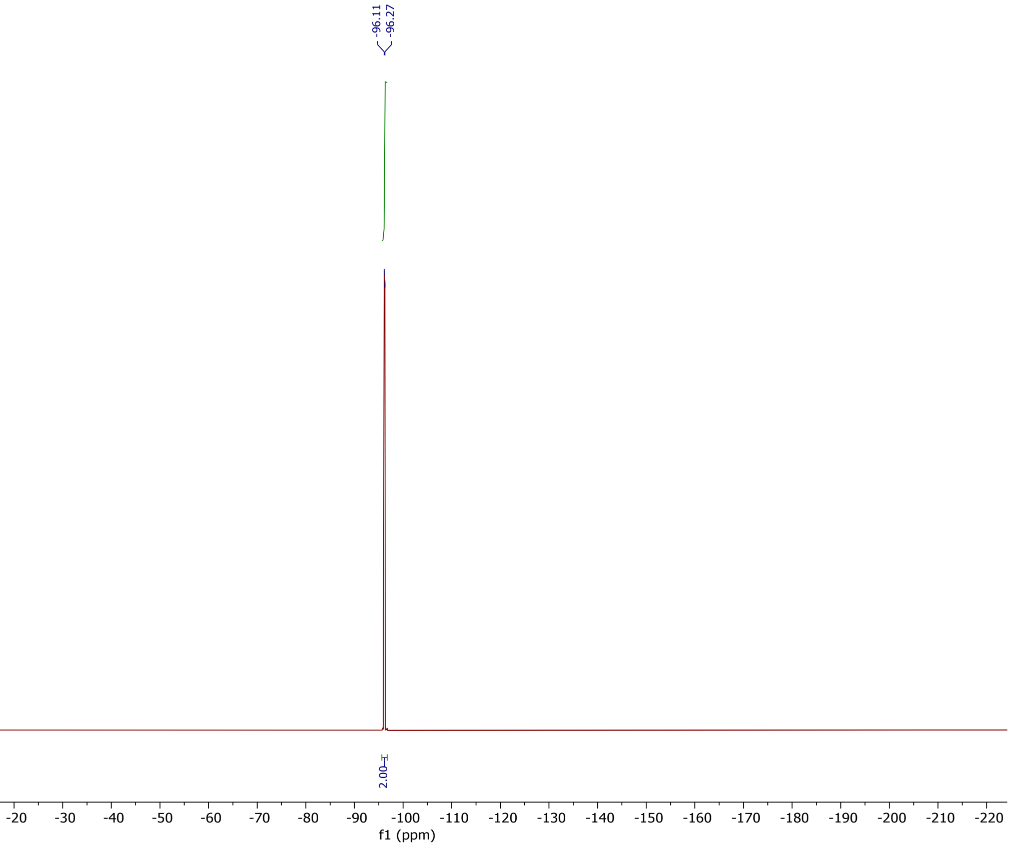

^{1}H NMR spectrum of **6q** ($CDCl_3$, 401 MHz)

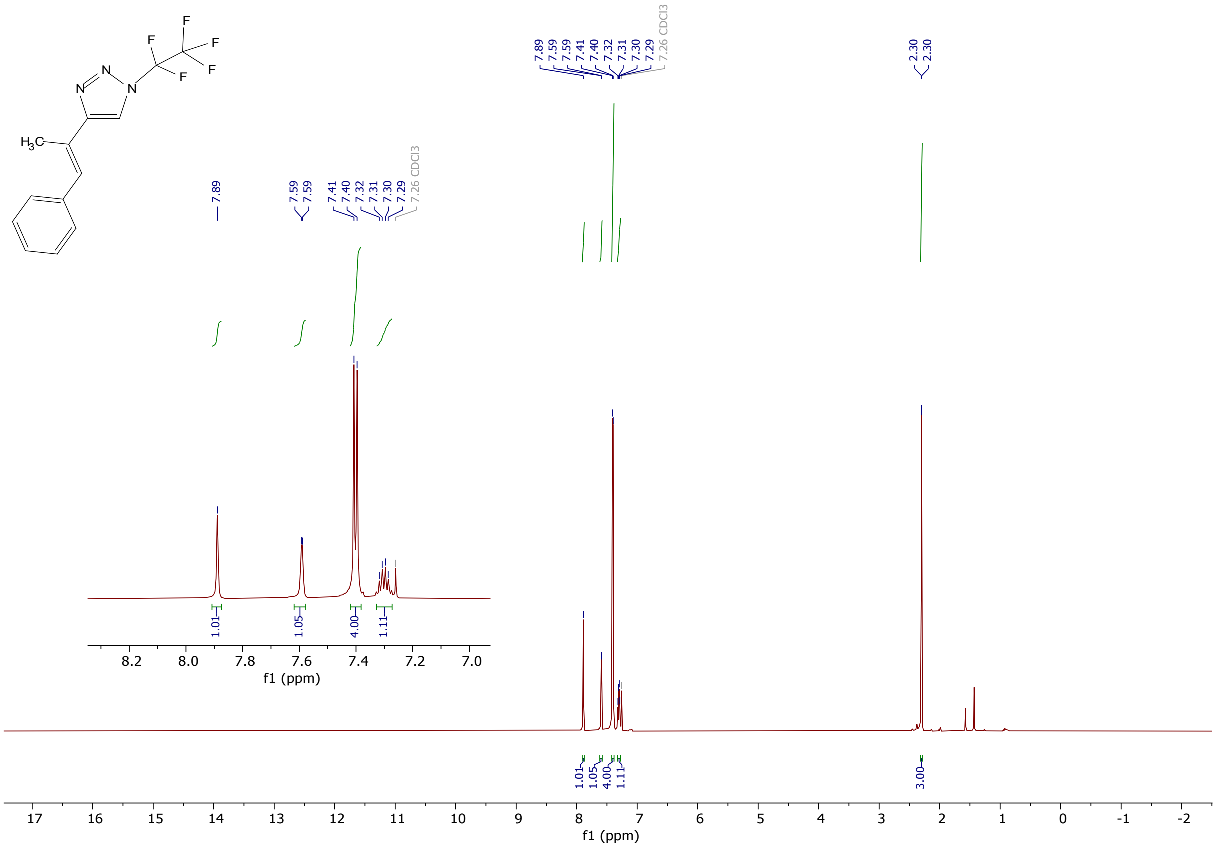

^{13}C NMR spectrum of **6q** ($CDCl_3$, 101 MHz)

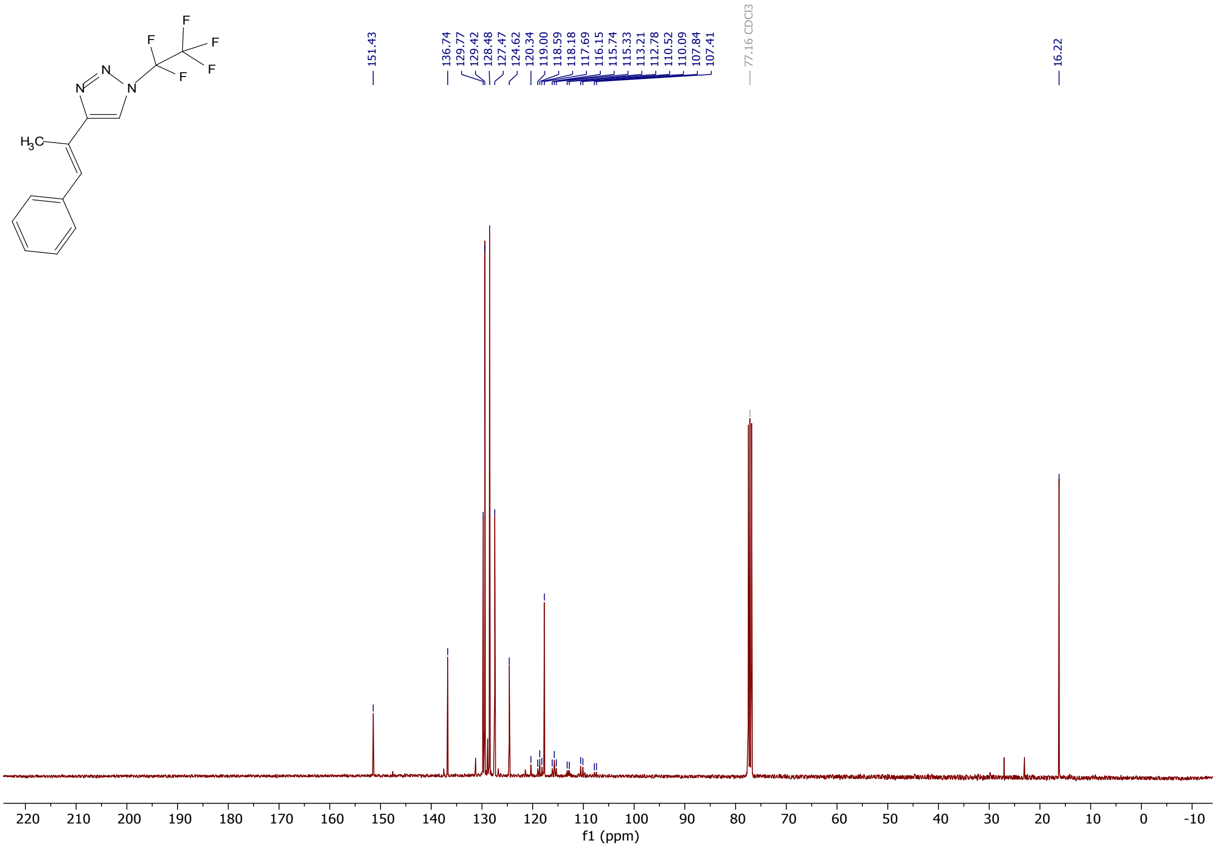

^{19}F NMR spectrum of **6q** ($CDCl_3$, 376 MHz)

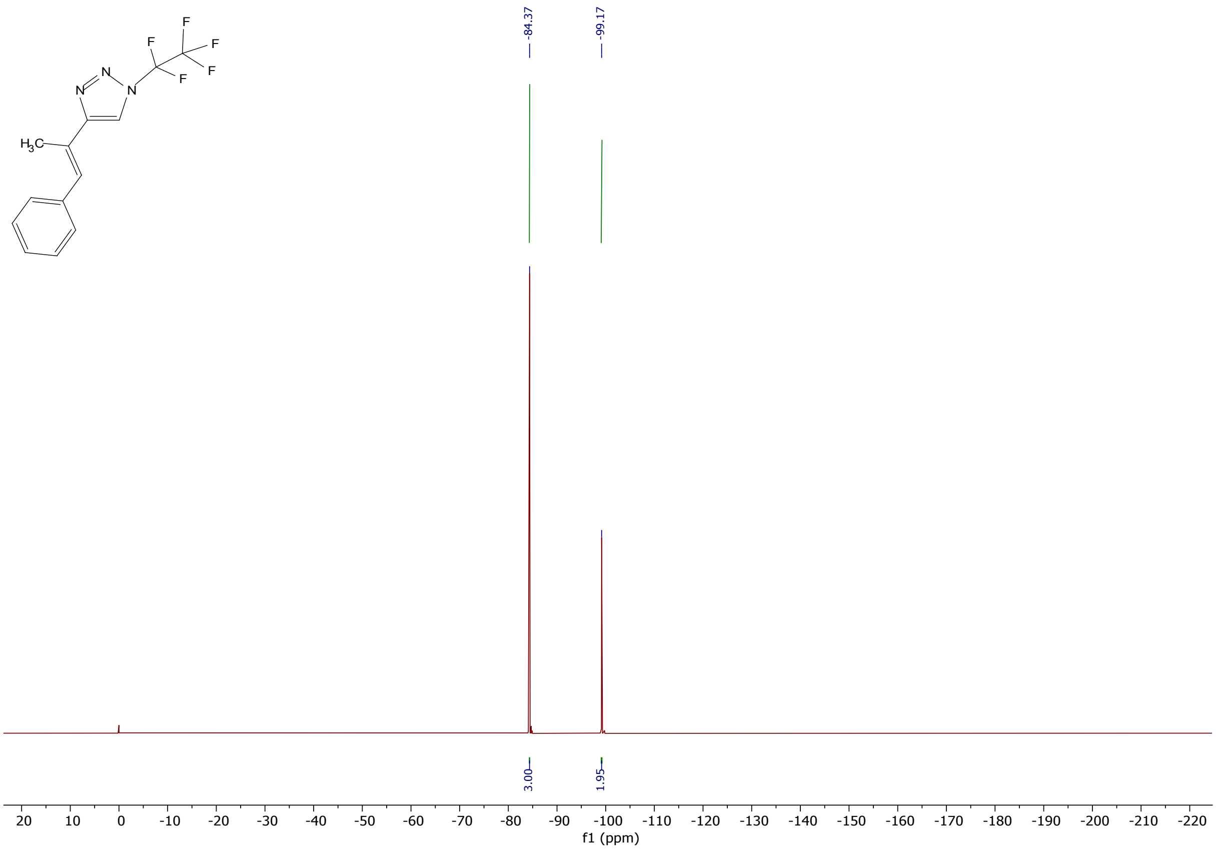

^{1}H NMR spectrum of **6r** ($CDCl_3$, 401 MHz)

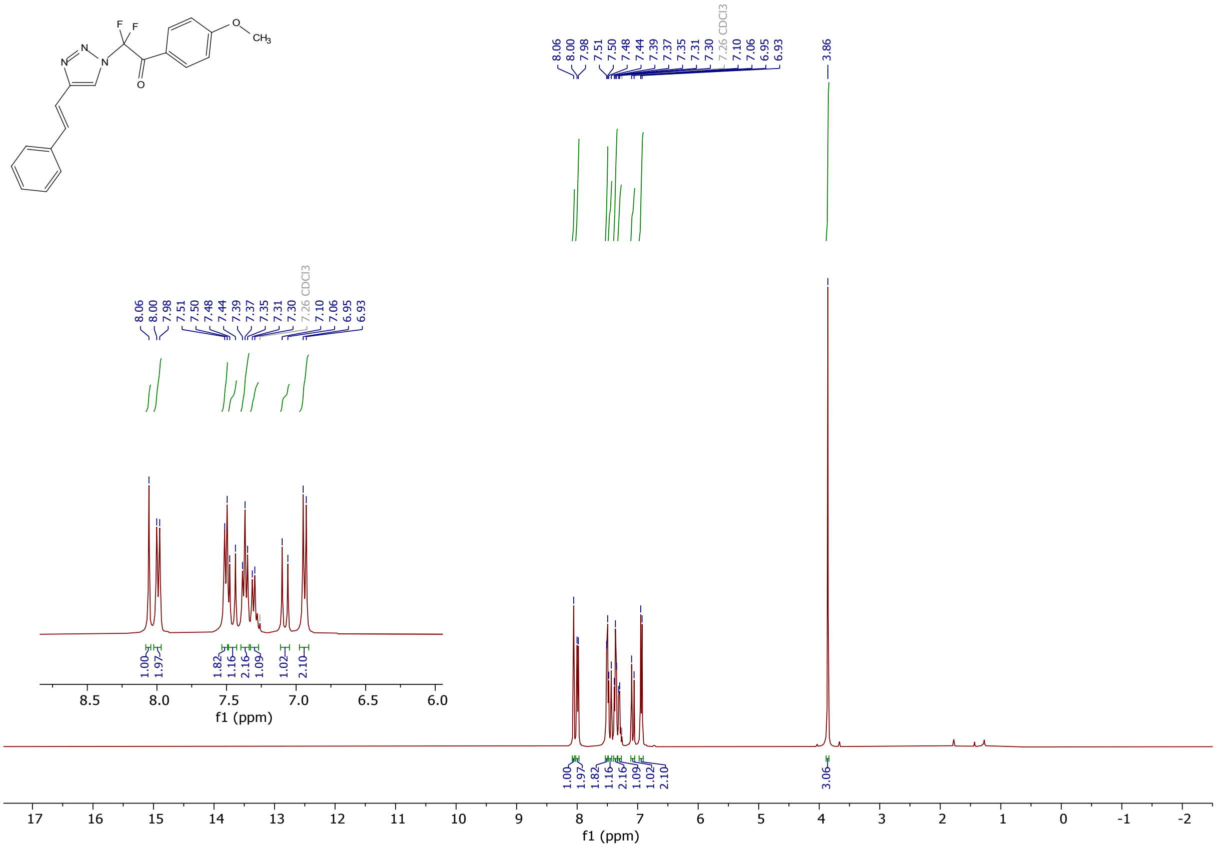

^{13}C NMR spectrum of **6r** ($CDCl_3$, 101 MHz)

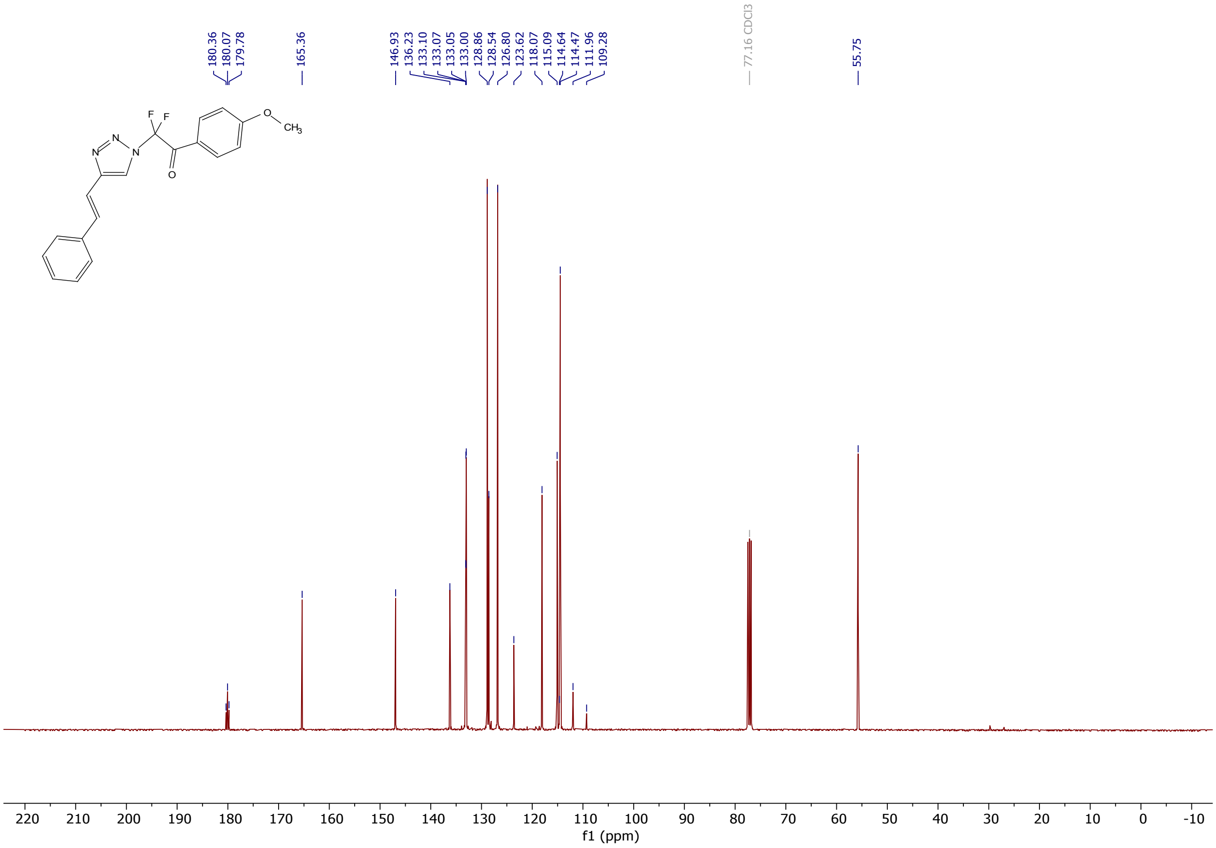

^{19}F NMR spectrum of **6r** ($CDCl_3$, 376 MHz)

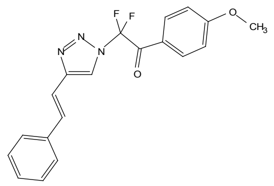

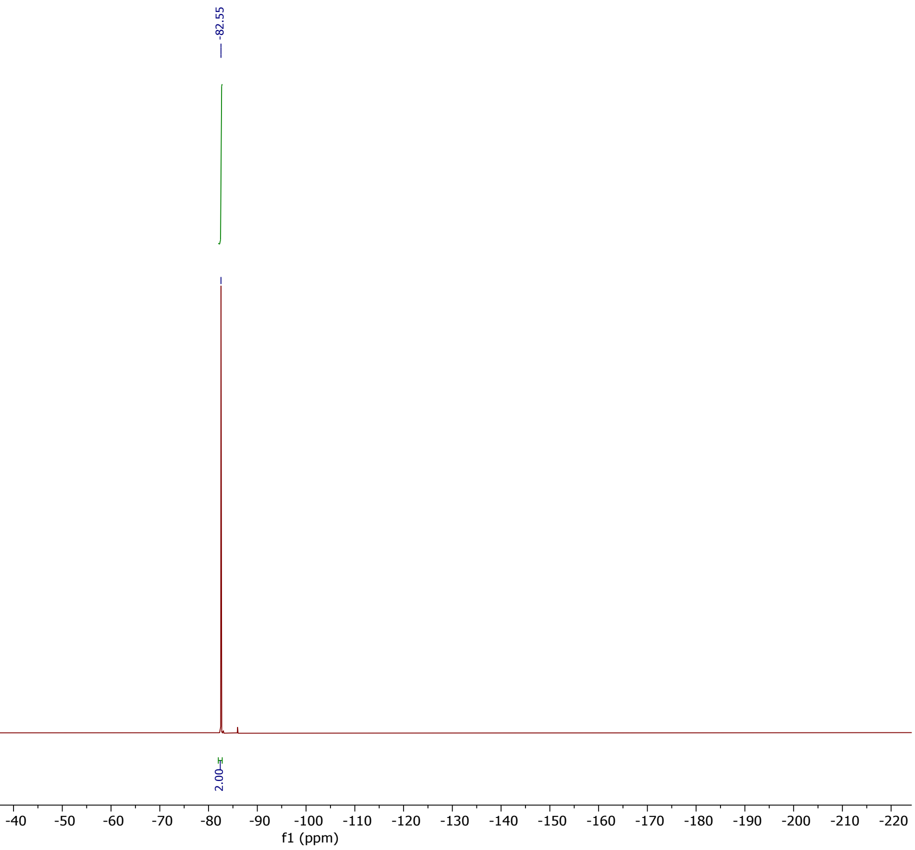

^{1}H NMR spectrum of **11a** ($CDCl_3$, 401 MHz)

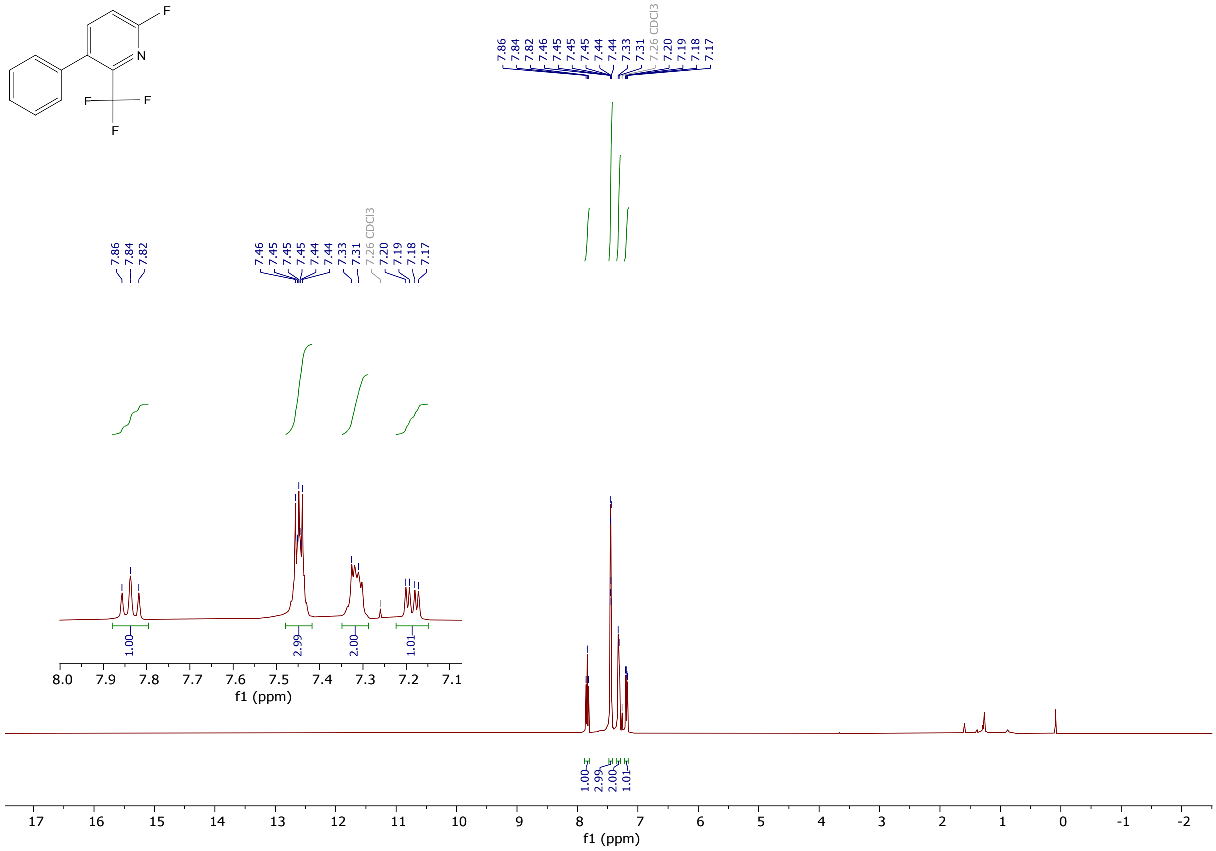

^{13}C NMR spectrum of **11a** ($CDCl_3$, 101 MHz)

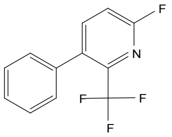

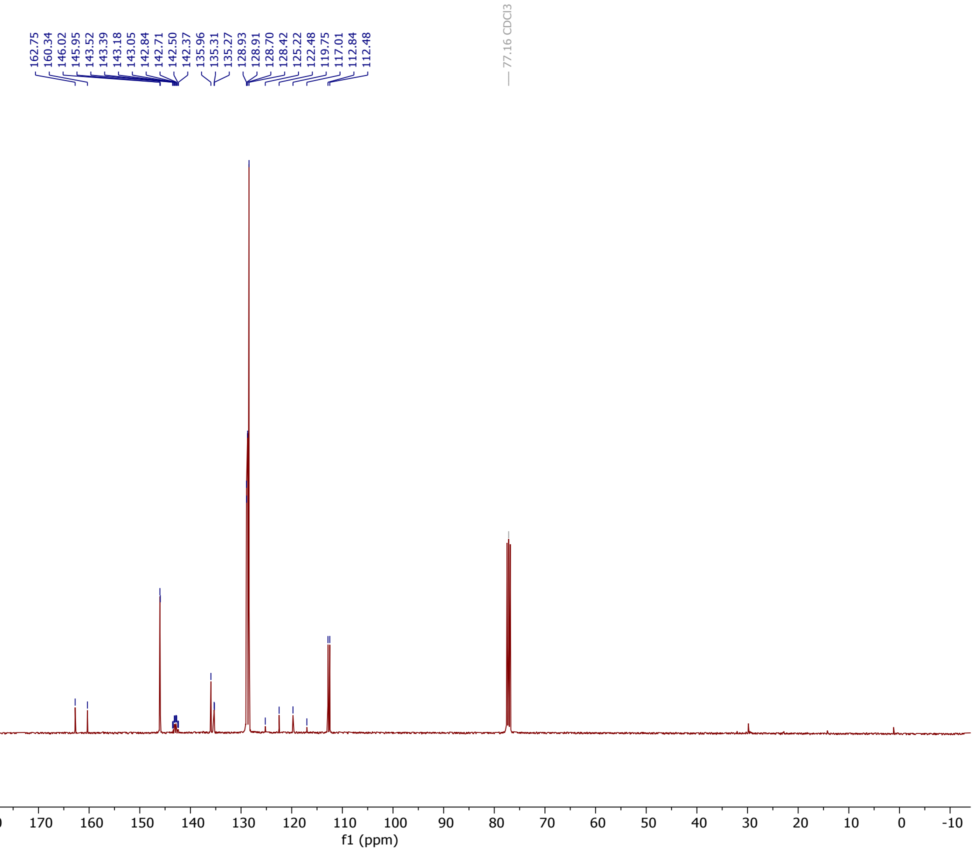

^{19}F NMR spectrum of **11a** ($CDCl_3$, 376 MHz)

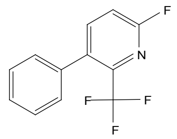

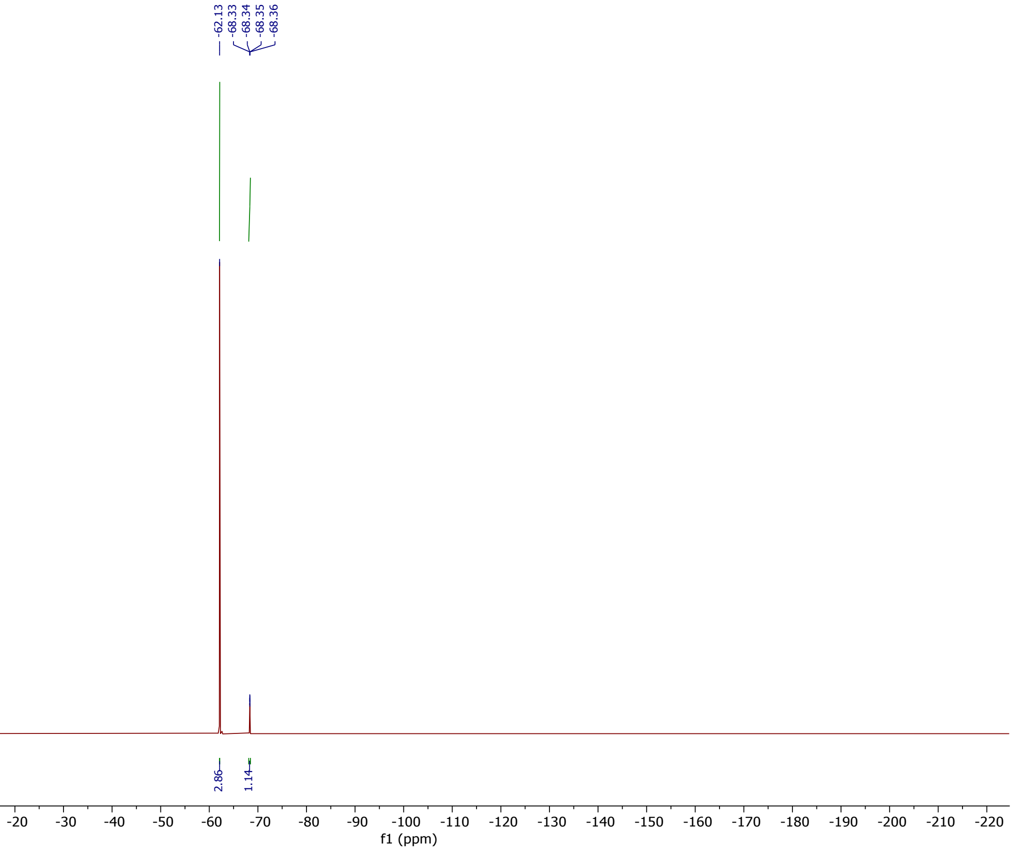

^{1}H NMR spectrum of **11b** ($CDCl_3$, 401 MHz)

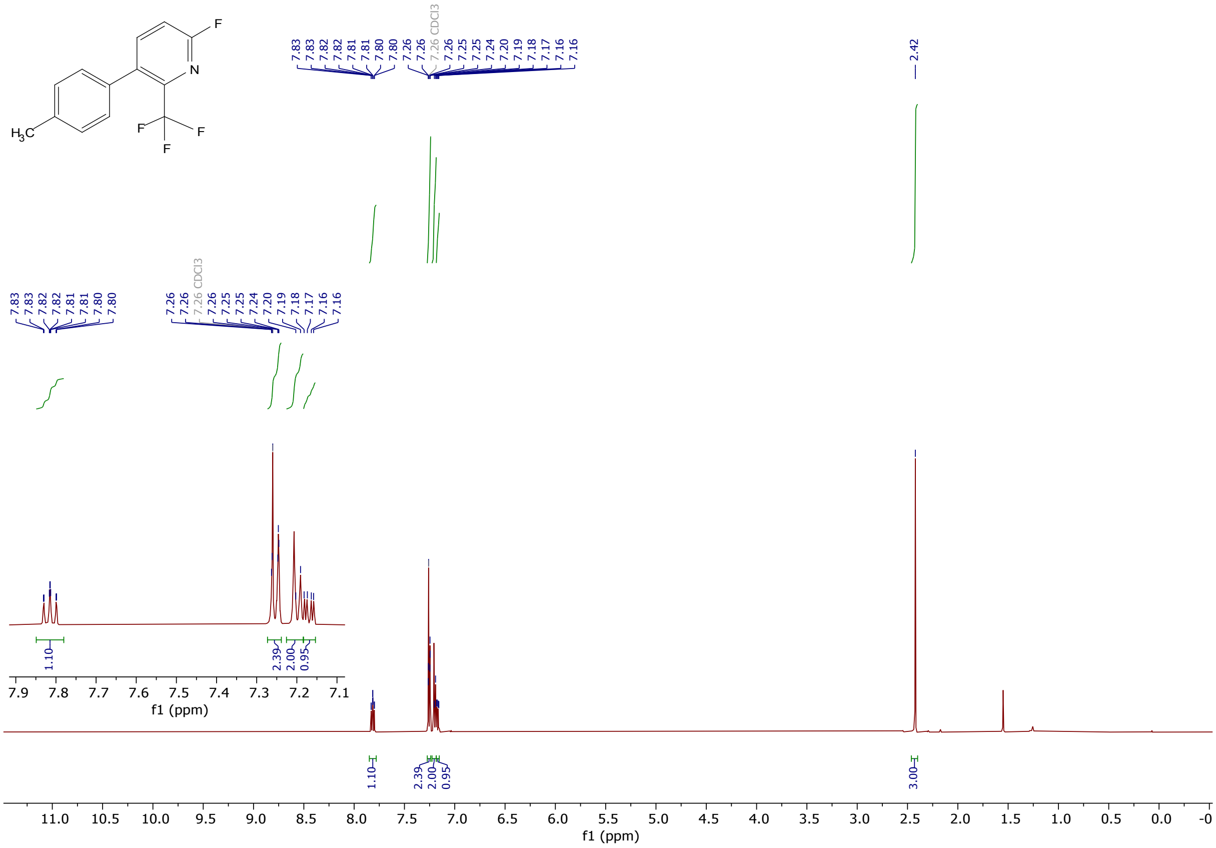

^{13}C NMR spectrum of **11b** ($CDCl_3$, 101 MHz)

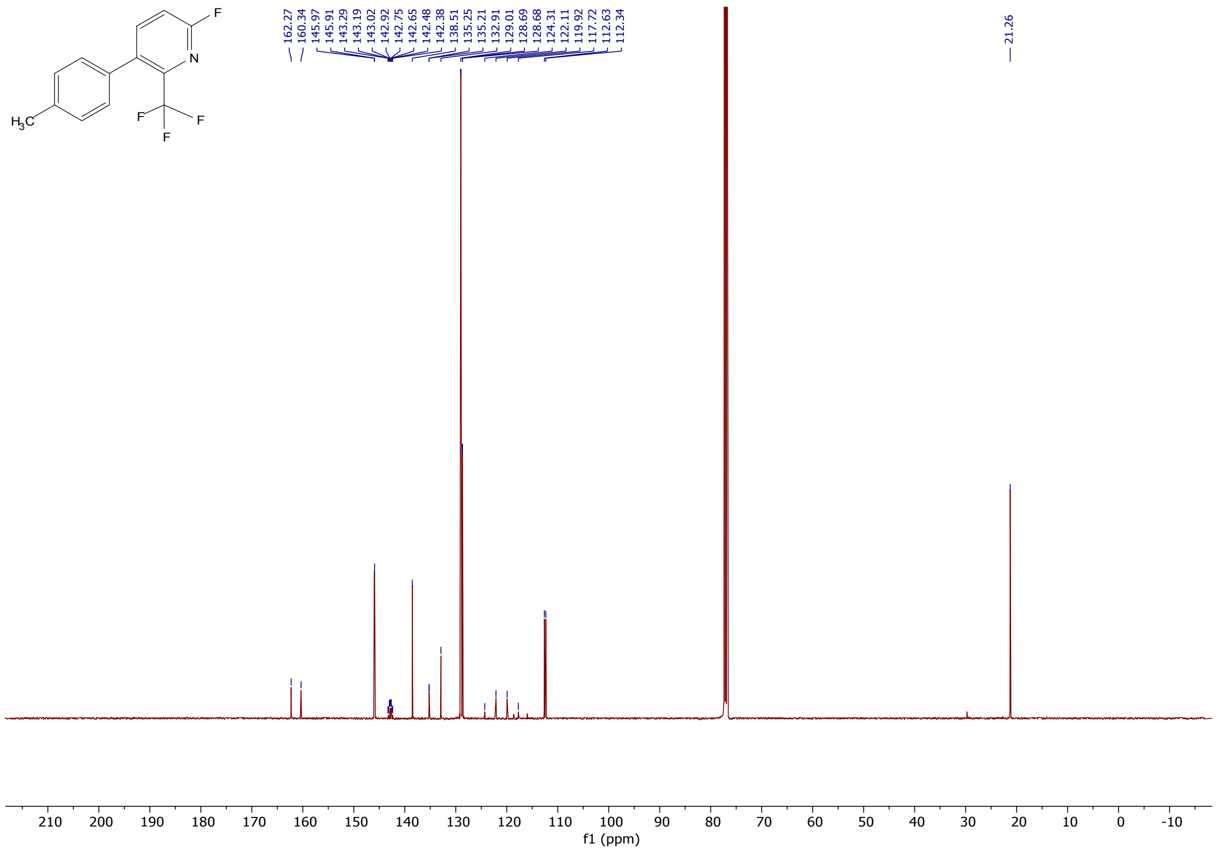

^{19}F NMR spectrum of **11b** ($CDCl_3$, 376 MHz)

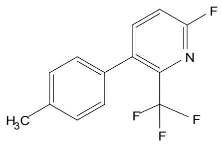

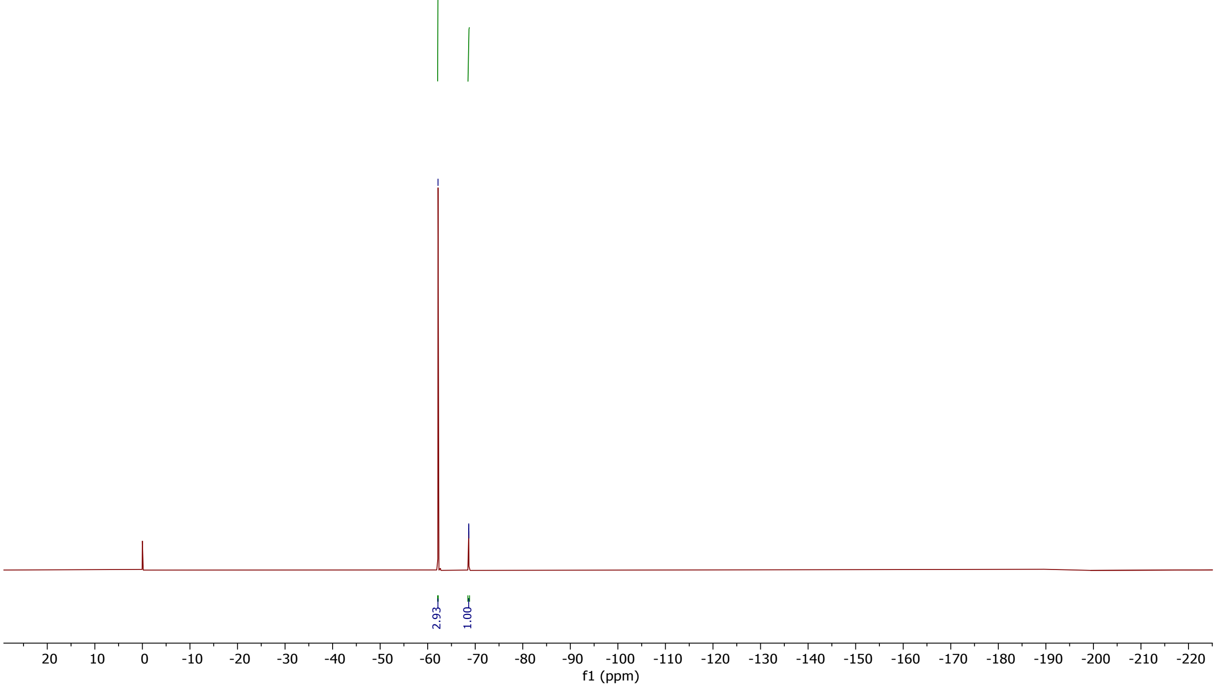

¹H NMR spectrum of **11c** ($CDCl_3$, 401 MHz)

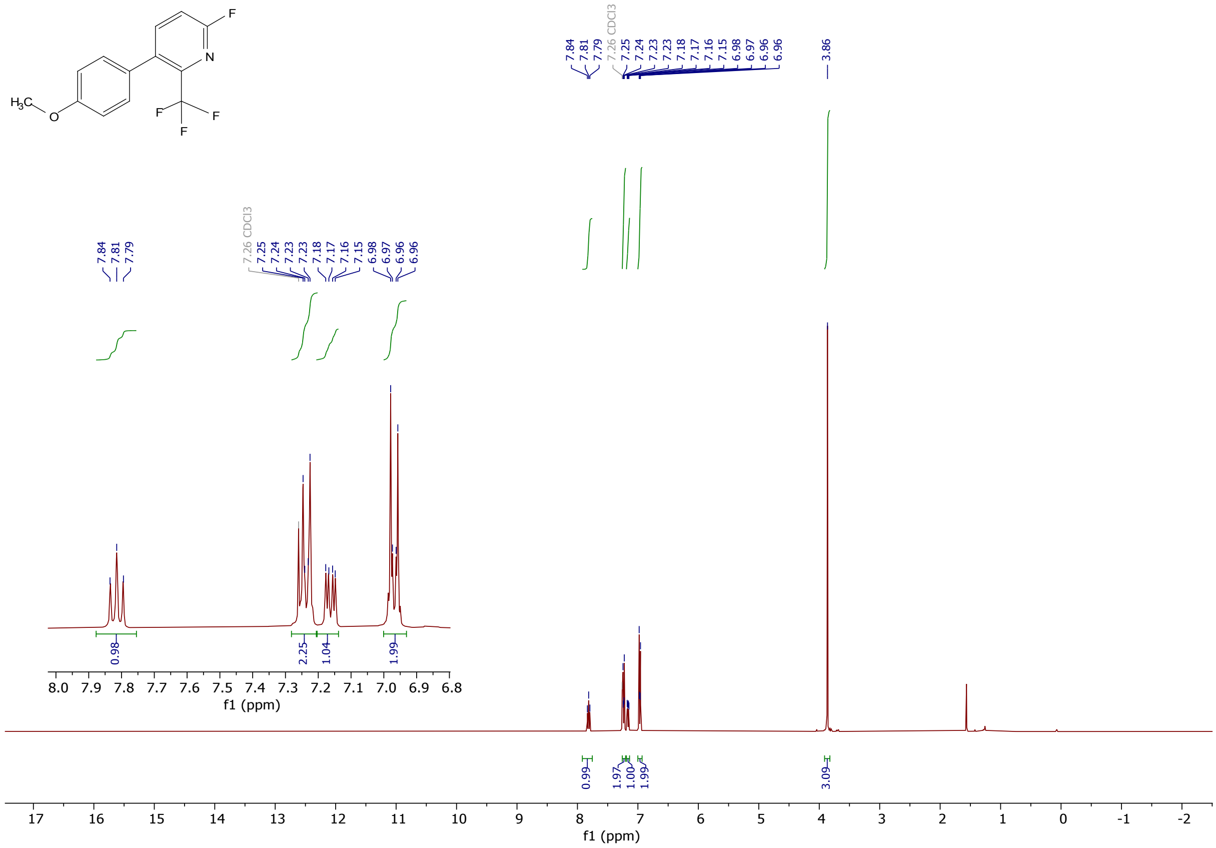

^{13}C NMR spectrum of **11c** ($CDCl_3$, 101 MHz)

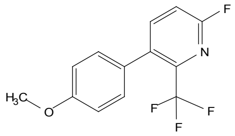

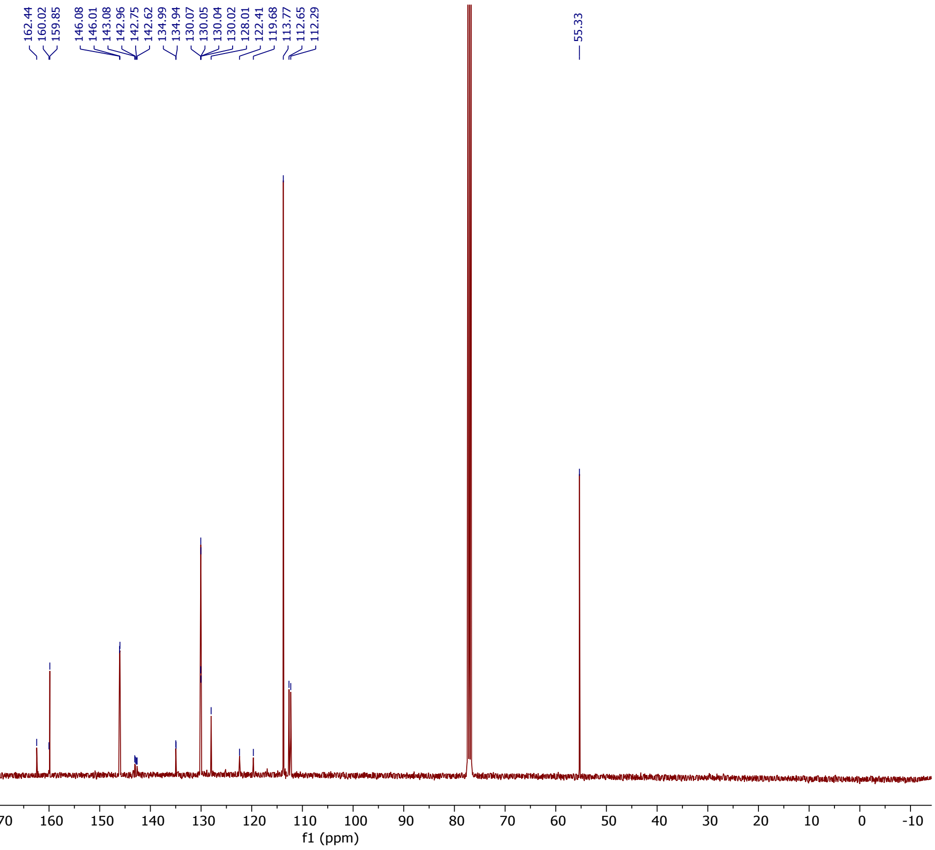

^{19}F NMR spectrum of **11c** ($CDCl_3$, 376 MHz)

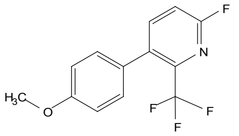

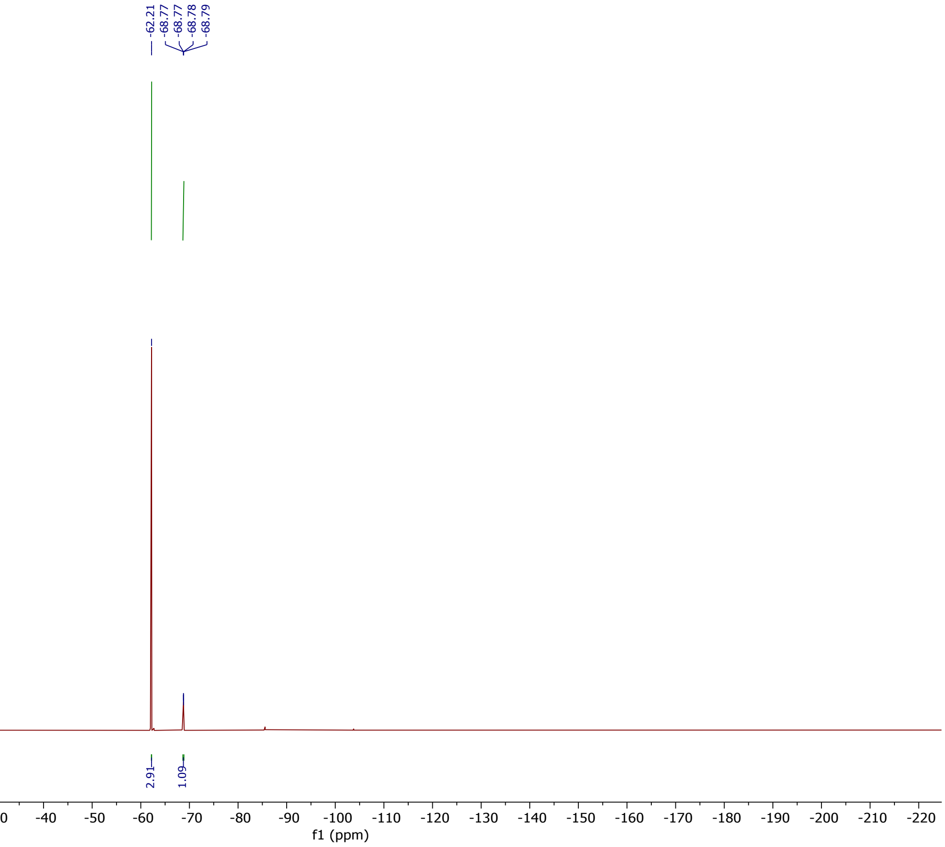

^{1}H NMR spectrum of **11d** ($CDCl_3$, 401 MHz)

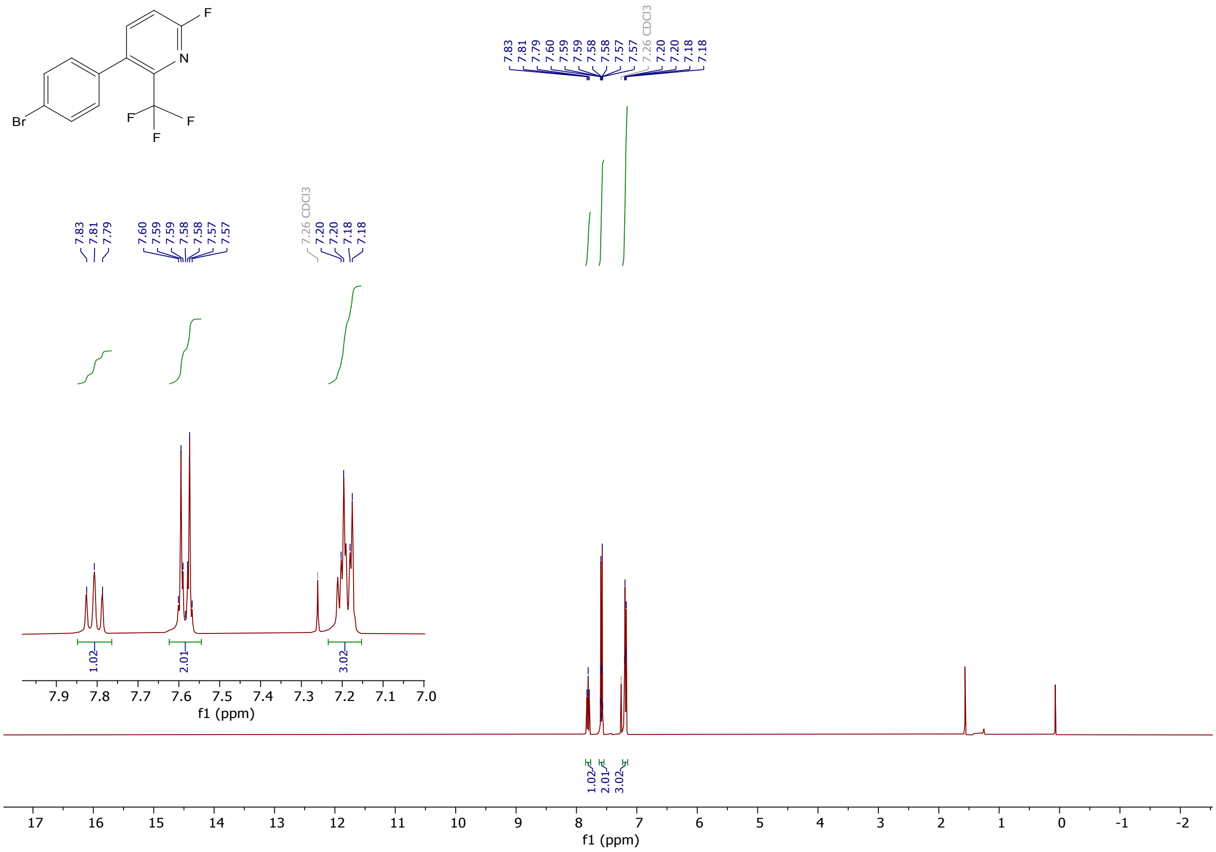

^{13}C NMR spectrum of **11d** ($CDCl_3$, 101 MHz)

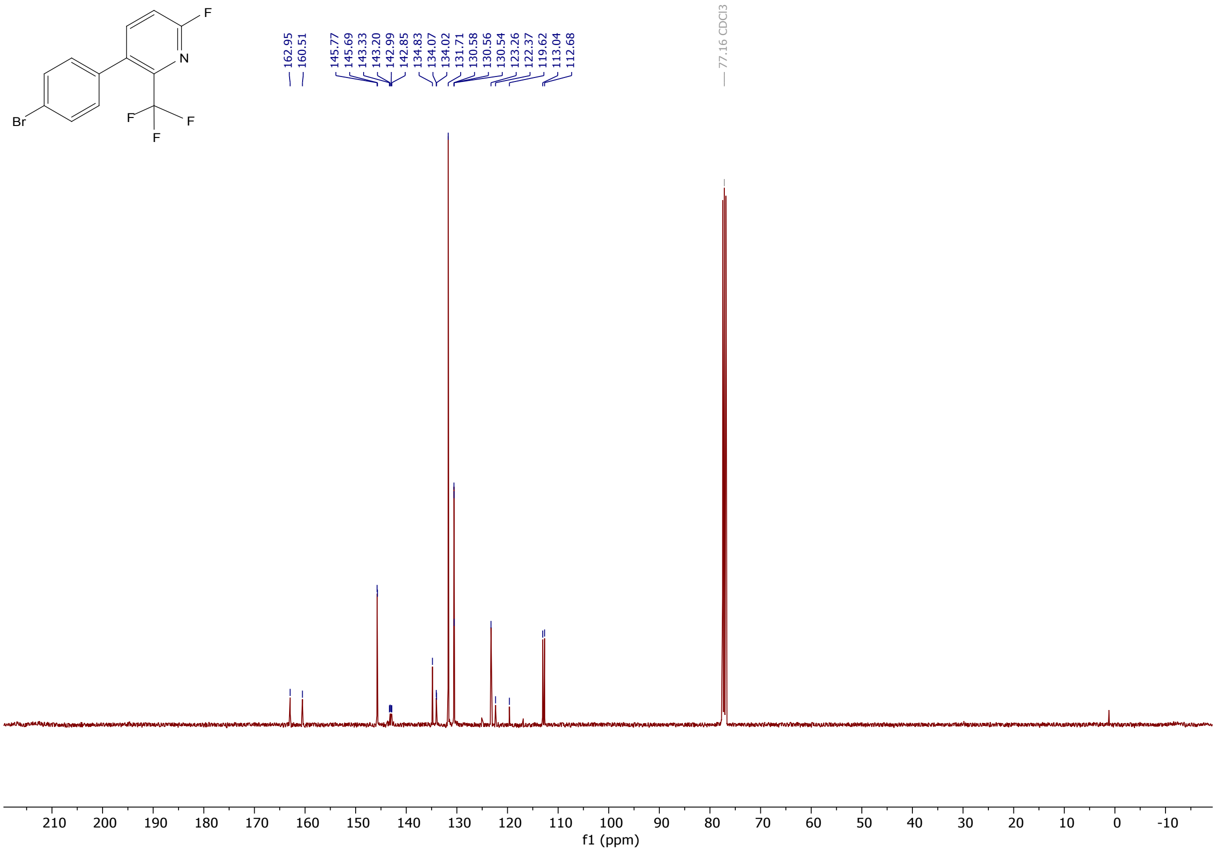

^{19}F NMR spectrum of **11d** ($CDCl_3$, 376 MHz)

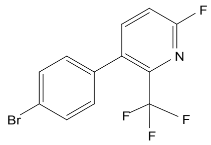

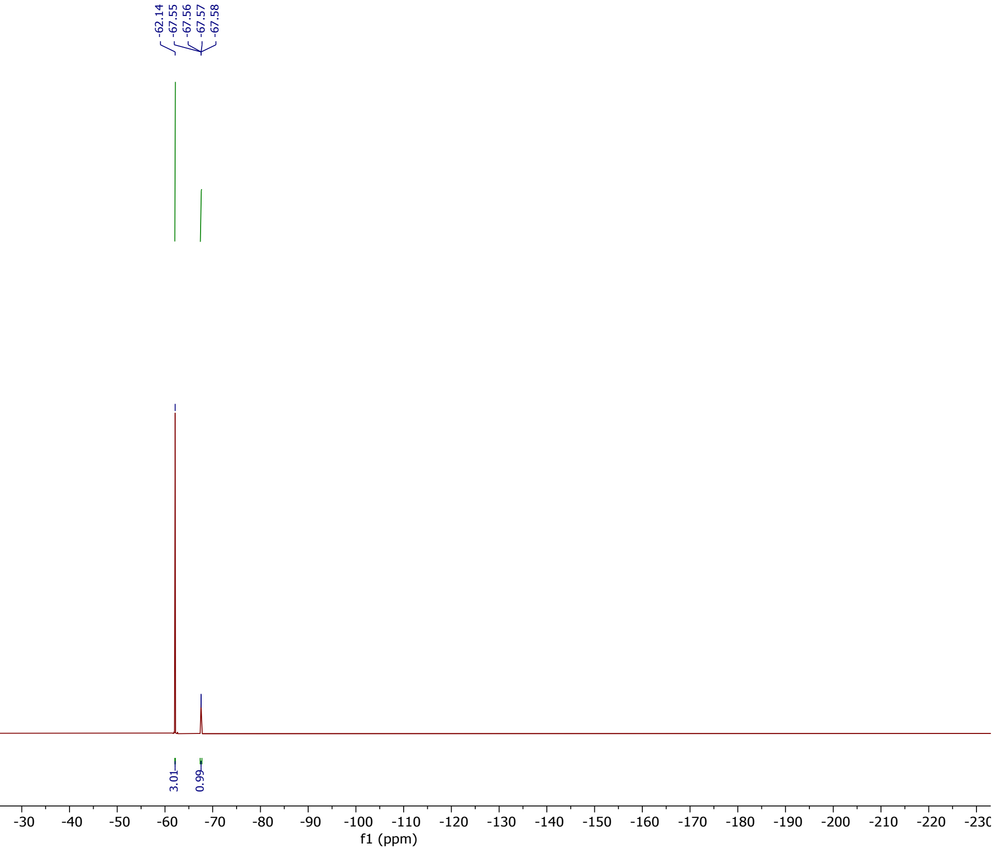

^{1}H NMR spectrum of **11e** ($CDCl_3$, 401 MHz)

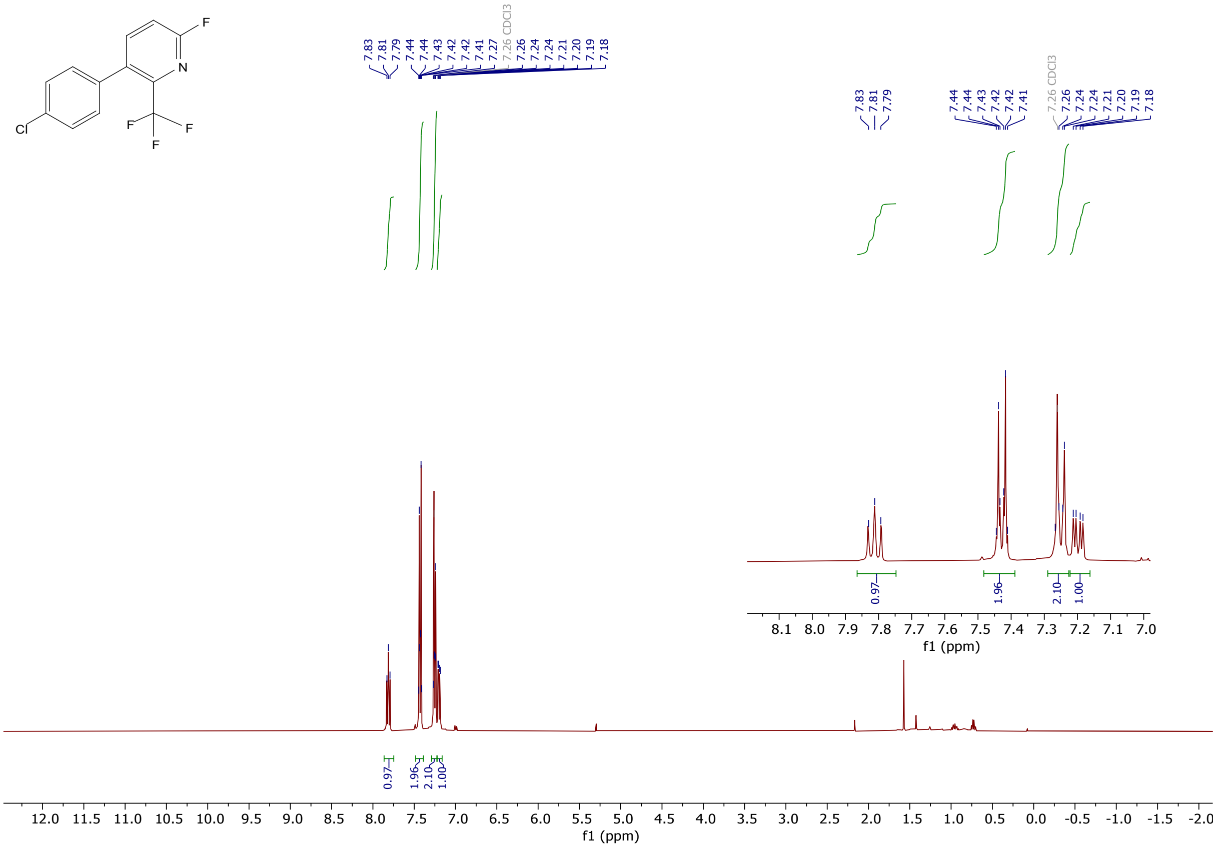

^{13}C NMR spectrum of **11e** ($CDCl_3$, 101 MHz)

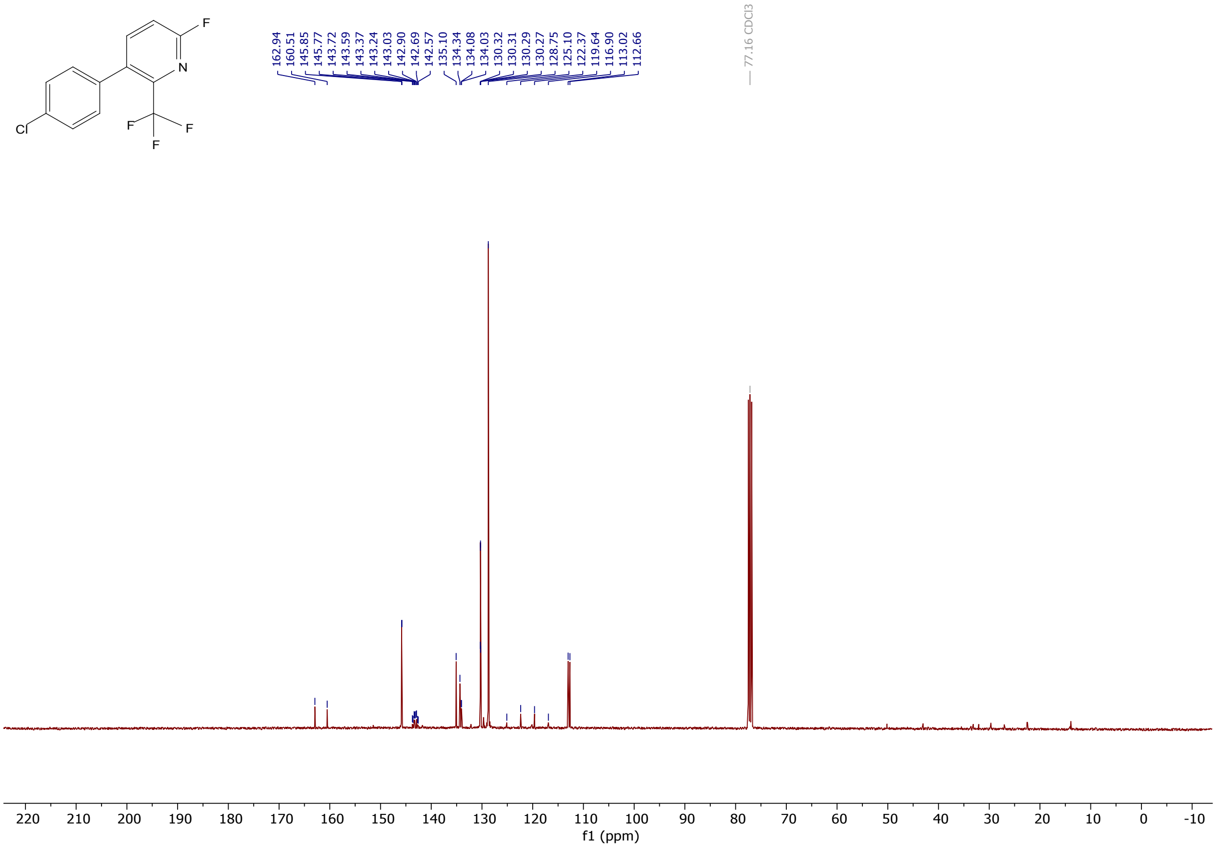

^{19}F NMR spectrum of **11e** ($CDCl_3$, 376 MHz)

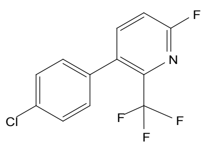

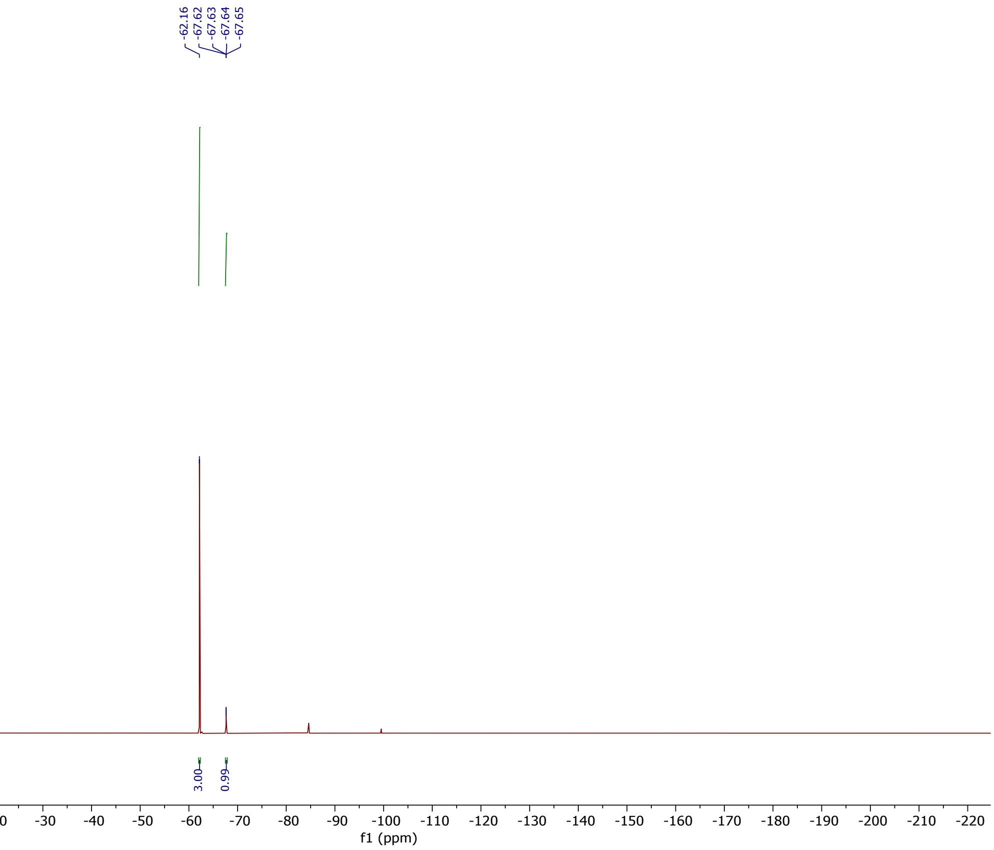

^1H NMR spectrum of **11f** ($CDCl_3$, 401 MHz)

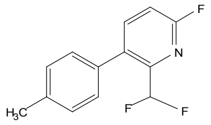

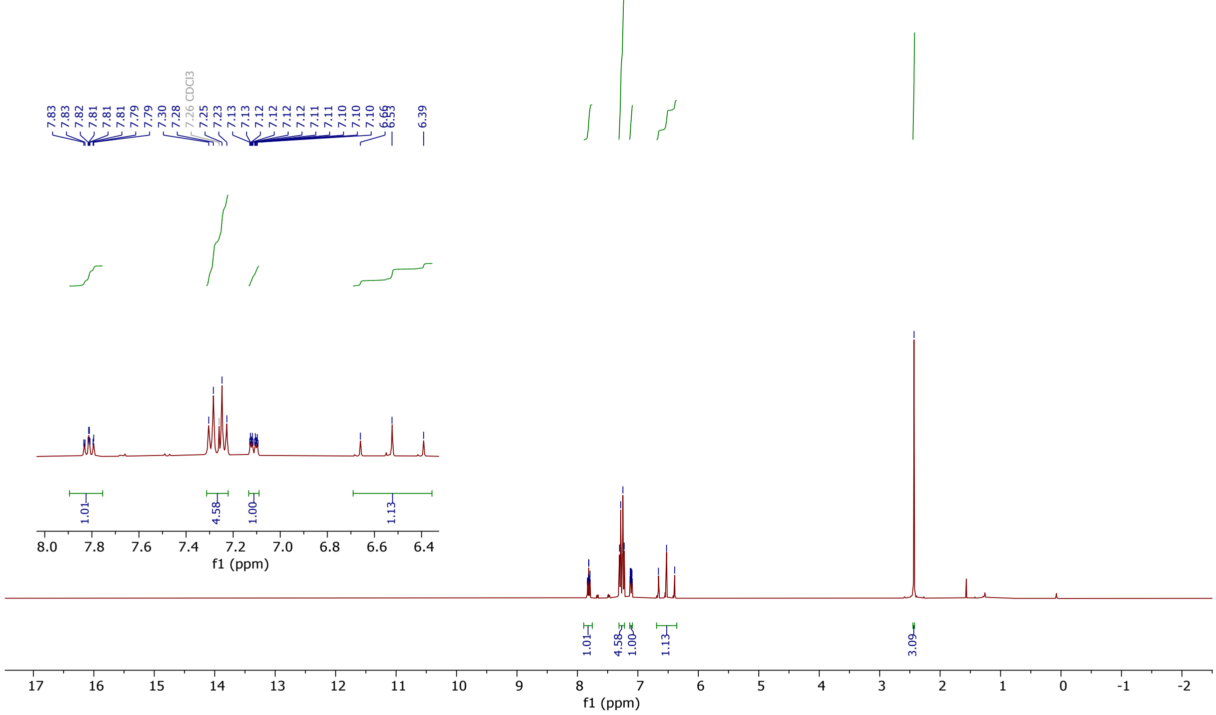

^{13}C NMR spectrum of **11f** ($CDCl_3$, 101 MHz)

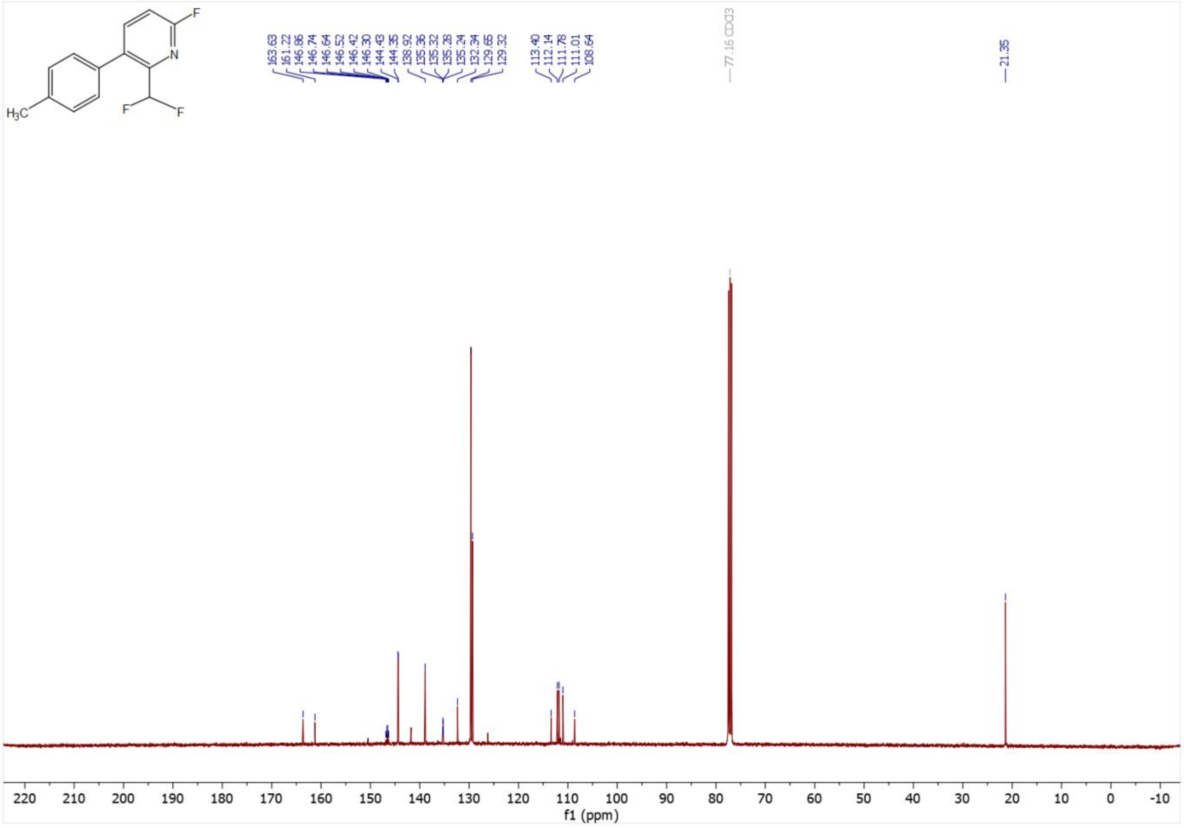

^{19}F NMR spectrum of **11f** ($CDCl_3$, 376 MHz)

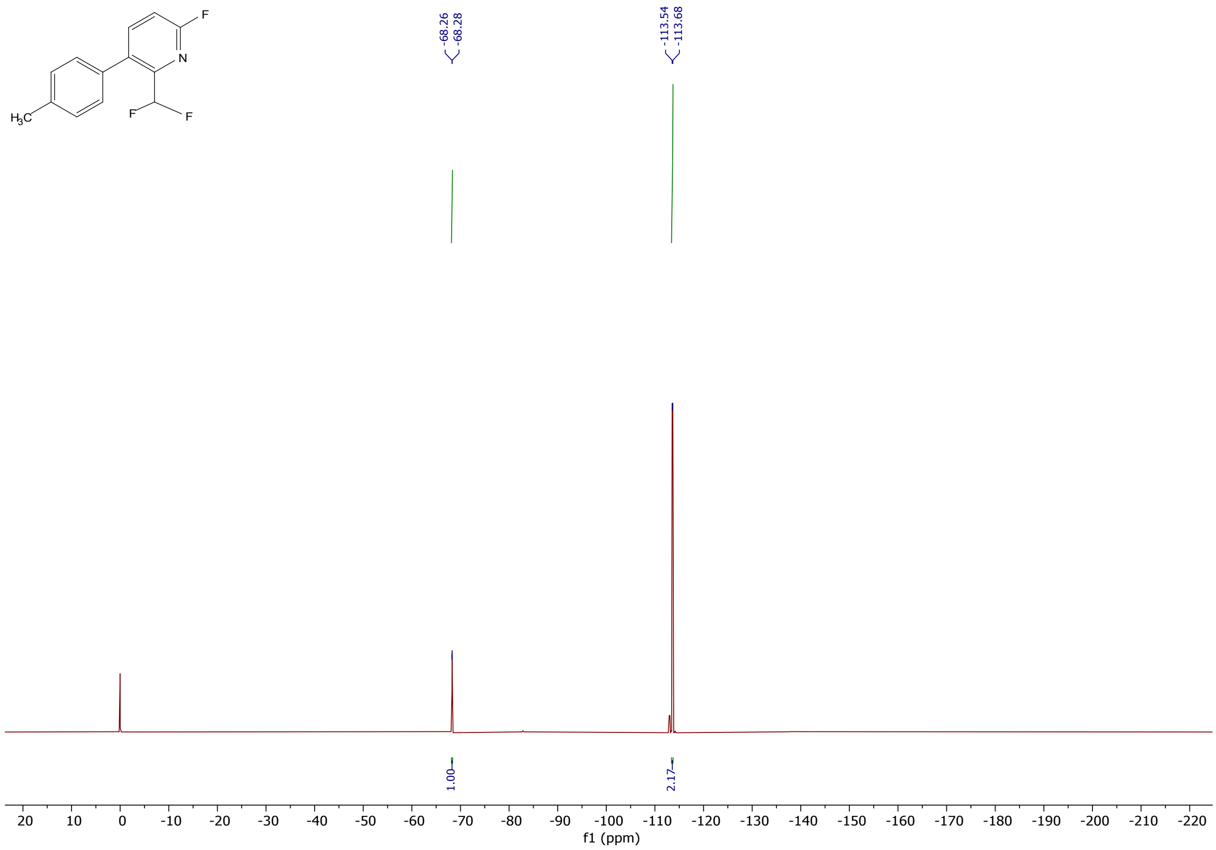

^{1}H NMR spectrum of **11g** ($CDCl_3$, 401 MHz)

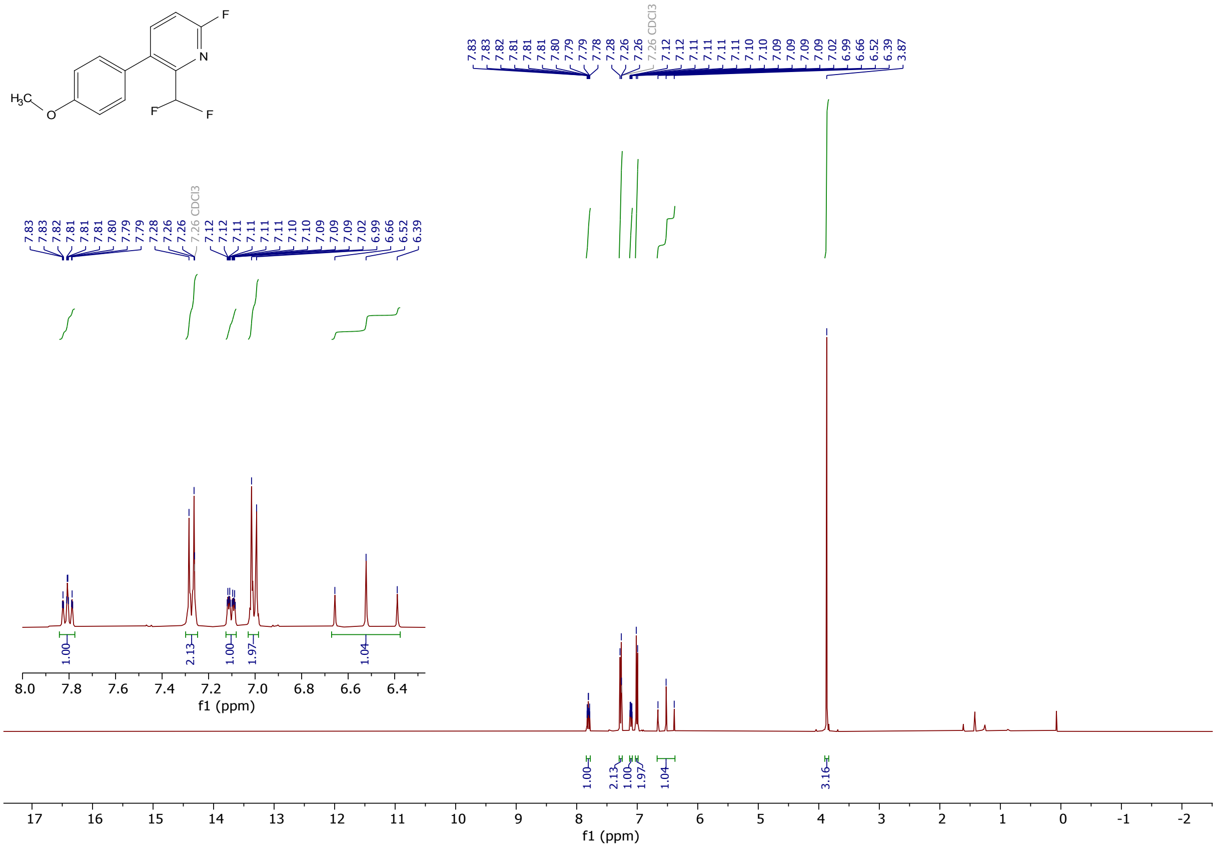

^{13}C NMR spectrum of **11g** ($CDCl_3$, 101 MHz)

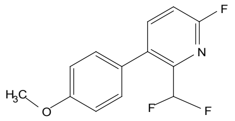

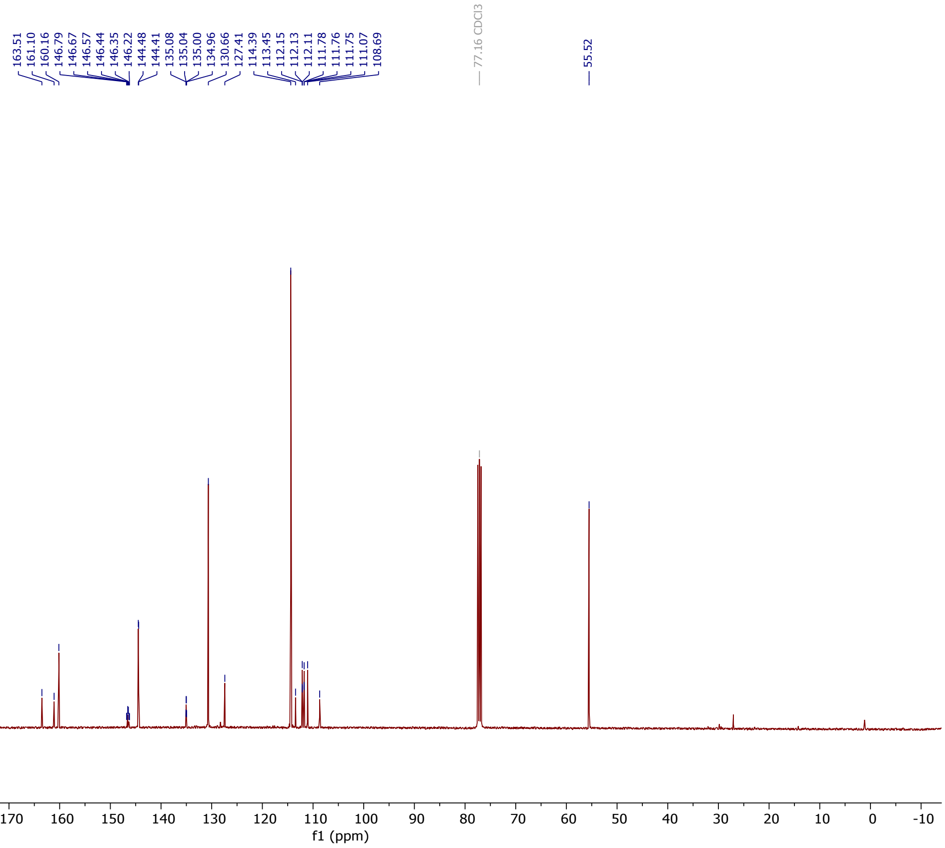

^{19}F NMR spectrum of **11g** ($CDCl_3$, 376 MHz)

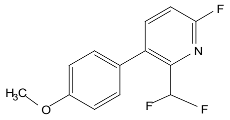

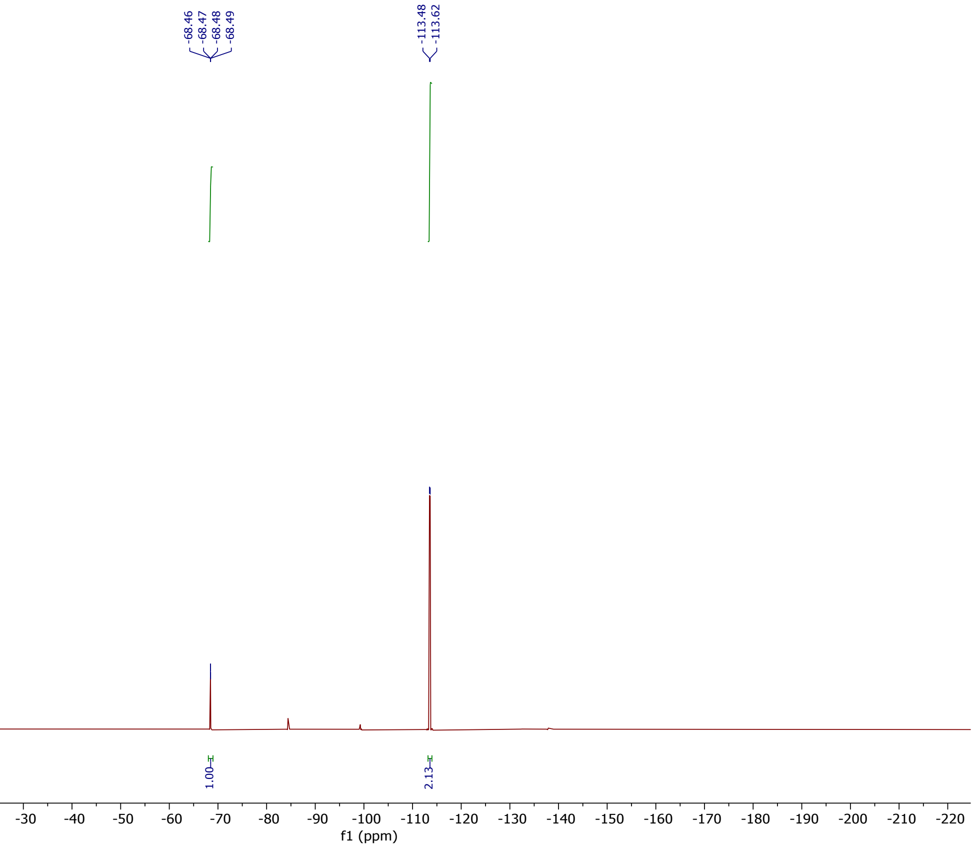

^{1}H NMR spectrum of **11h** ($CDCl_3$, 401 MHz)

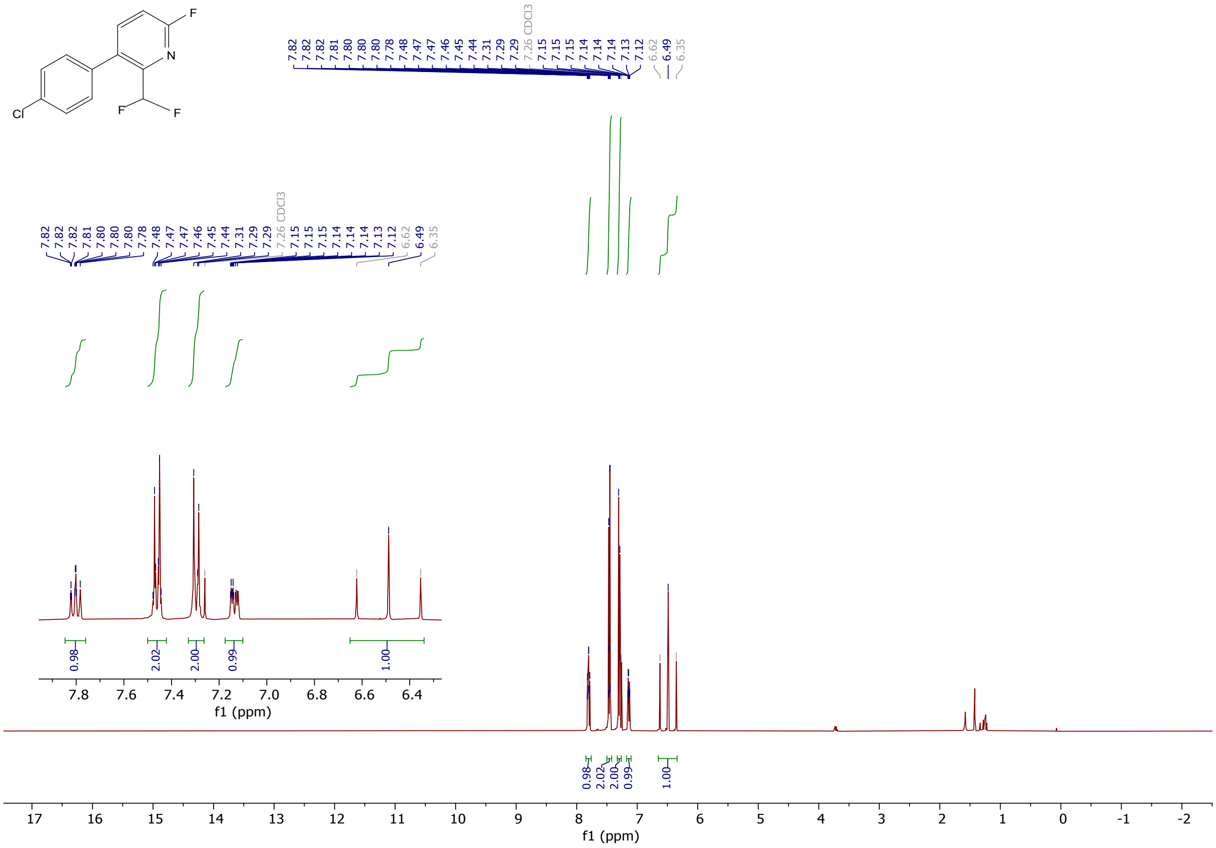

^{13}C NMR spectrum of **11h** ($CDCl_3$, 101 MHz)

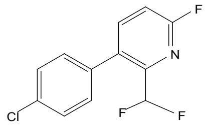

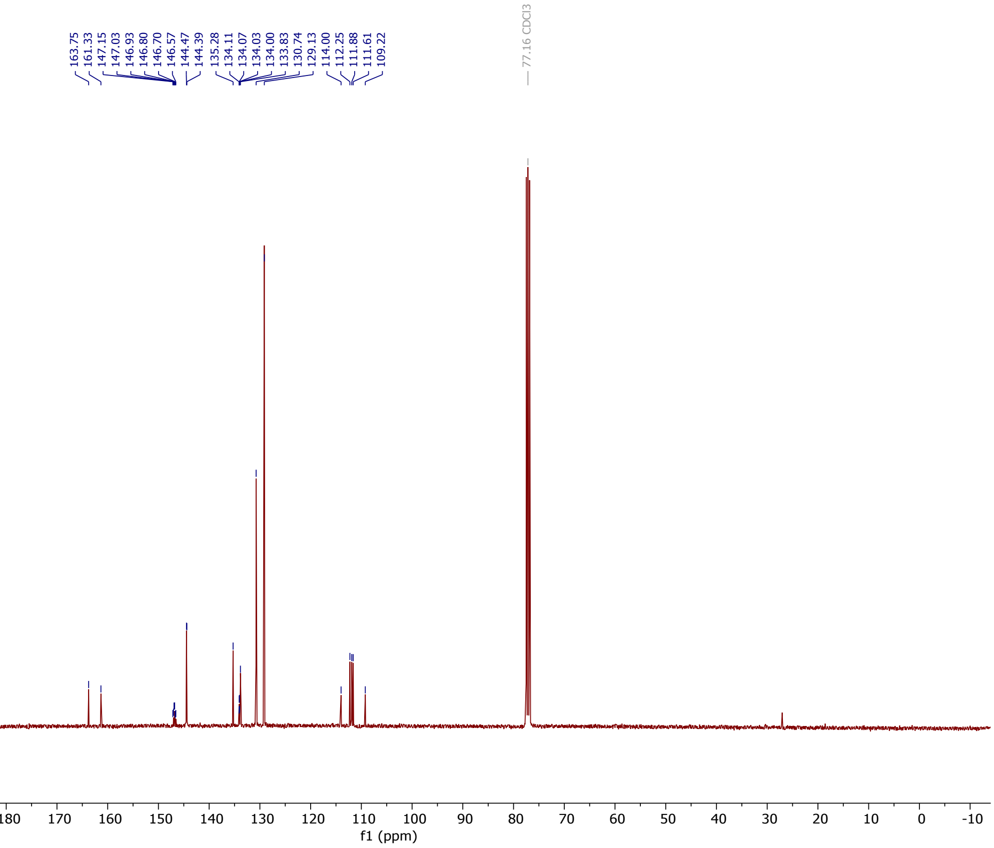

^{19}F NMR spectrum of **11h** ($CDCl_3$, 376 MHz)

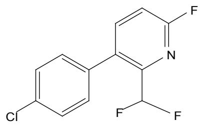

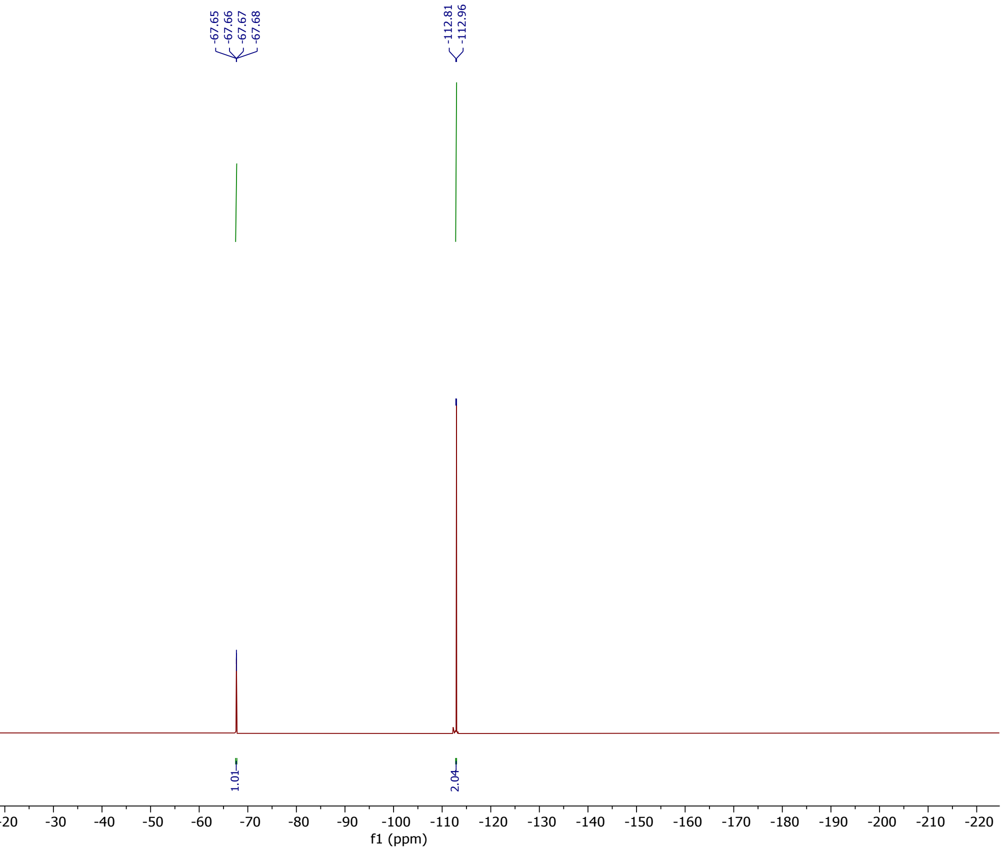

^{1}H NMR spectrum of **11i** ($CDCl_3$, 401 MHz)

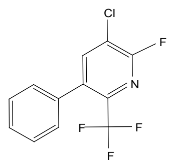

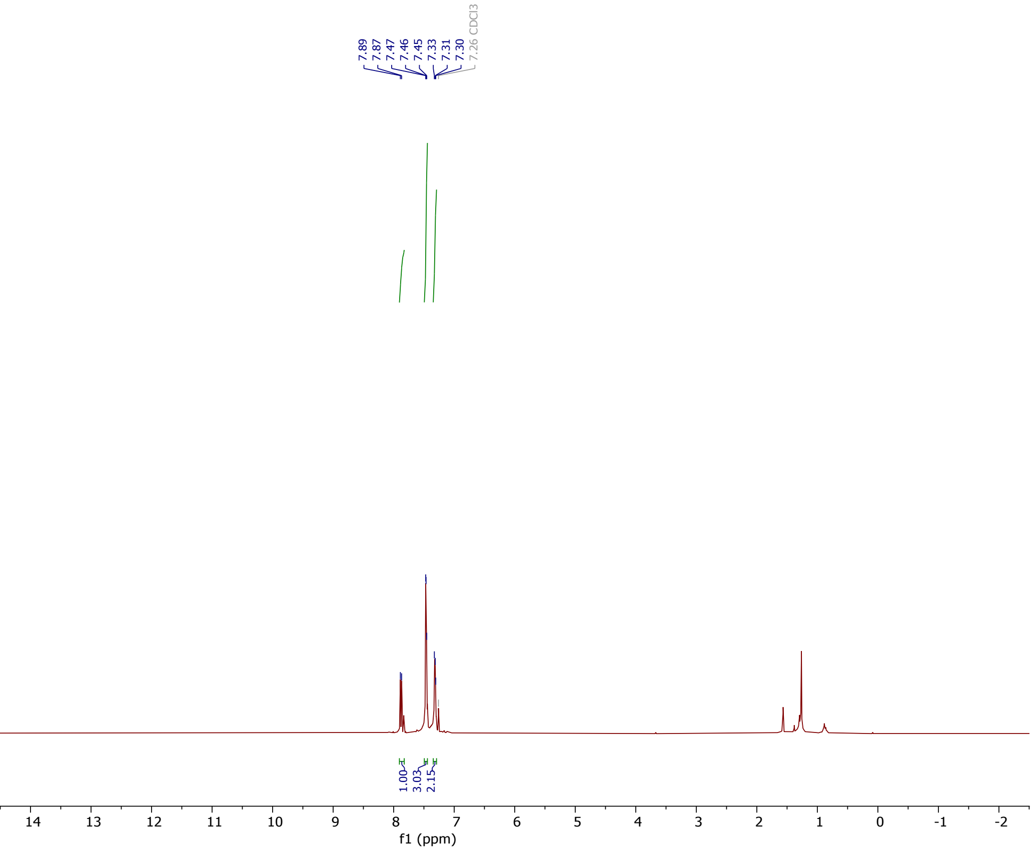

^{13}C NMR spectrum of **11i** ($CDCl_3$, 101 MHz)

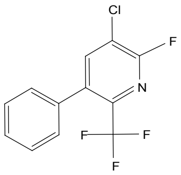

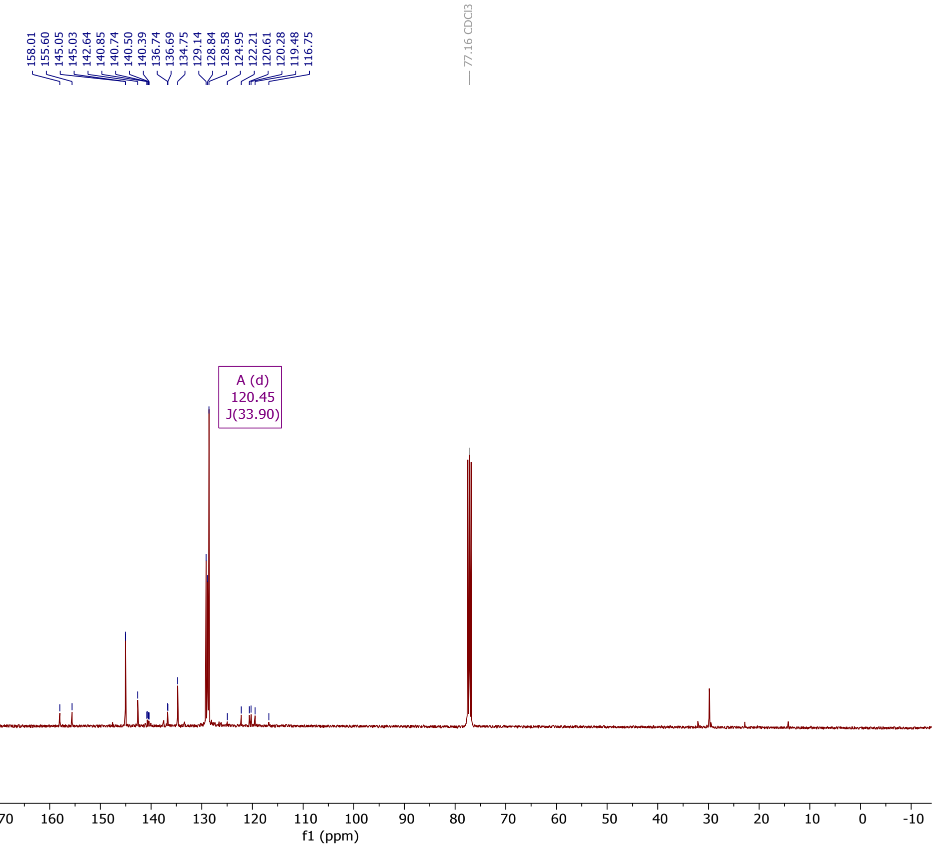

^{19}F NMR spectrum of **11i** ($CDCl_3$, 376 MHz)

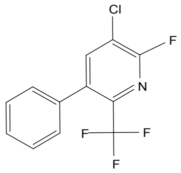

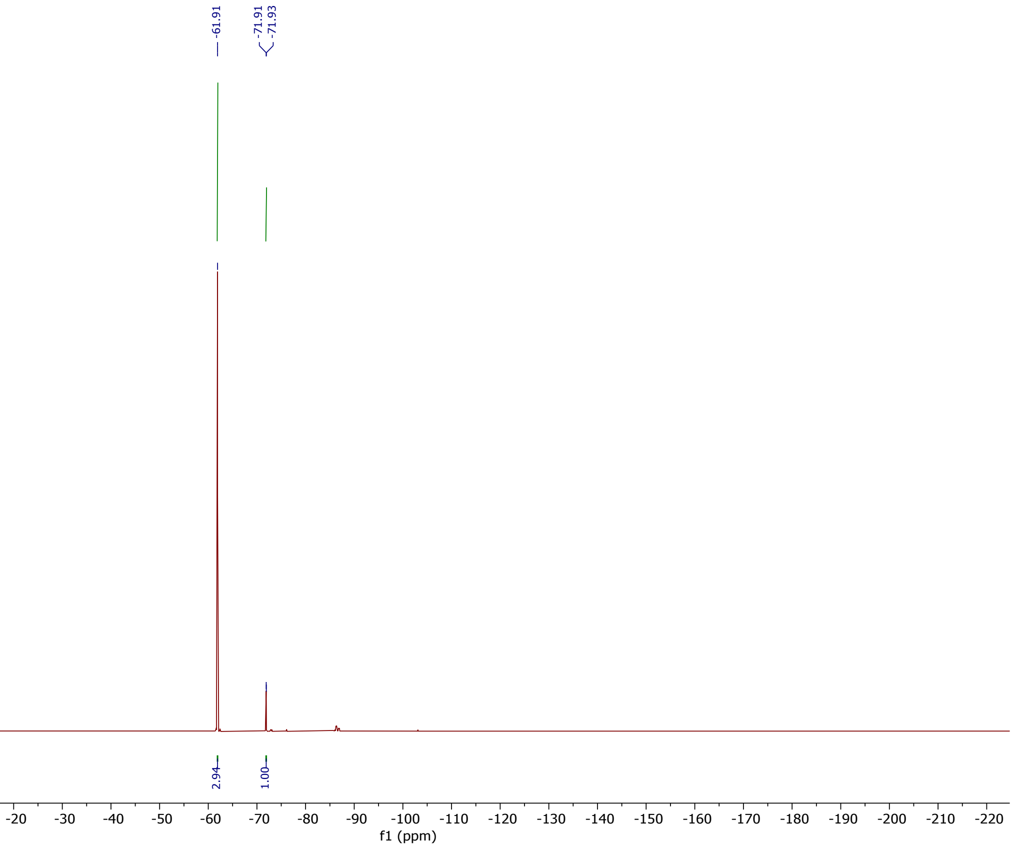

^{1}H NMR spectrum of **11j** ($CDCl_3$, 401 MHz)

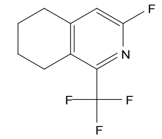

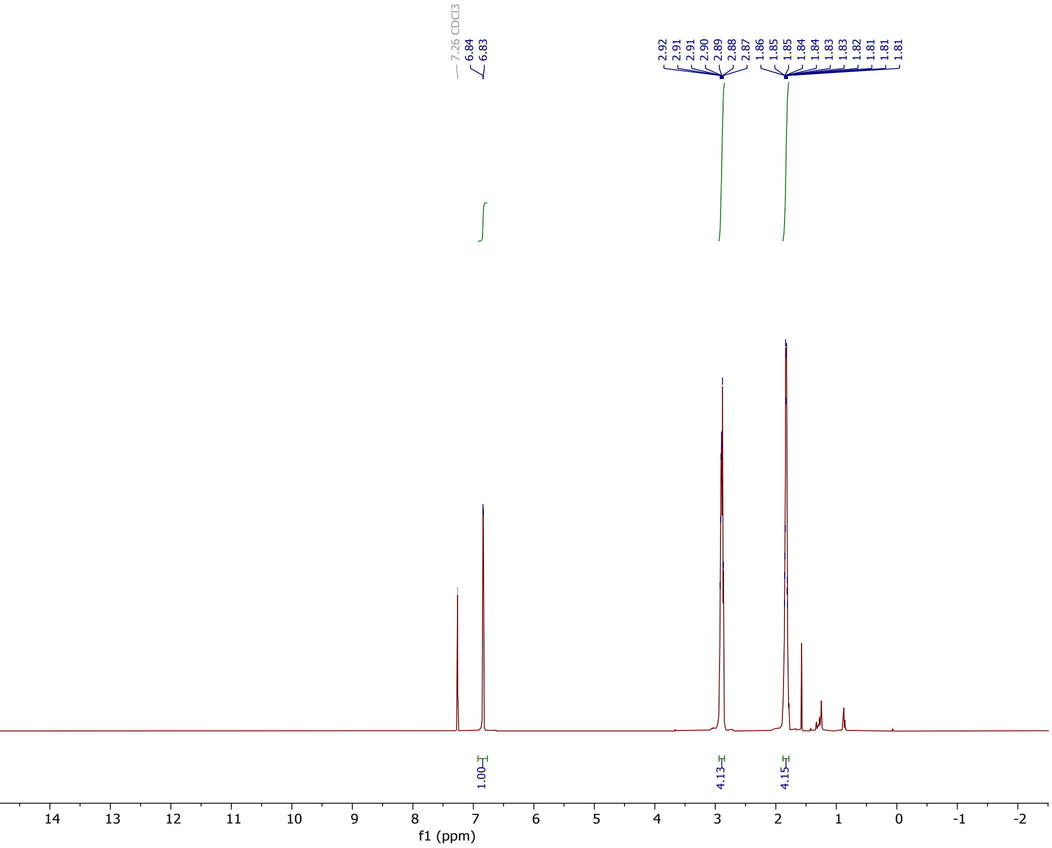

^{13}C NMR spectrum of **11j** ($CDCl_3$, 101 MHz)

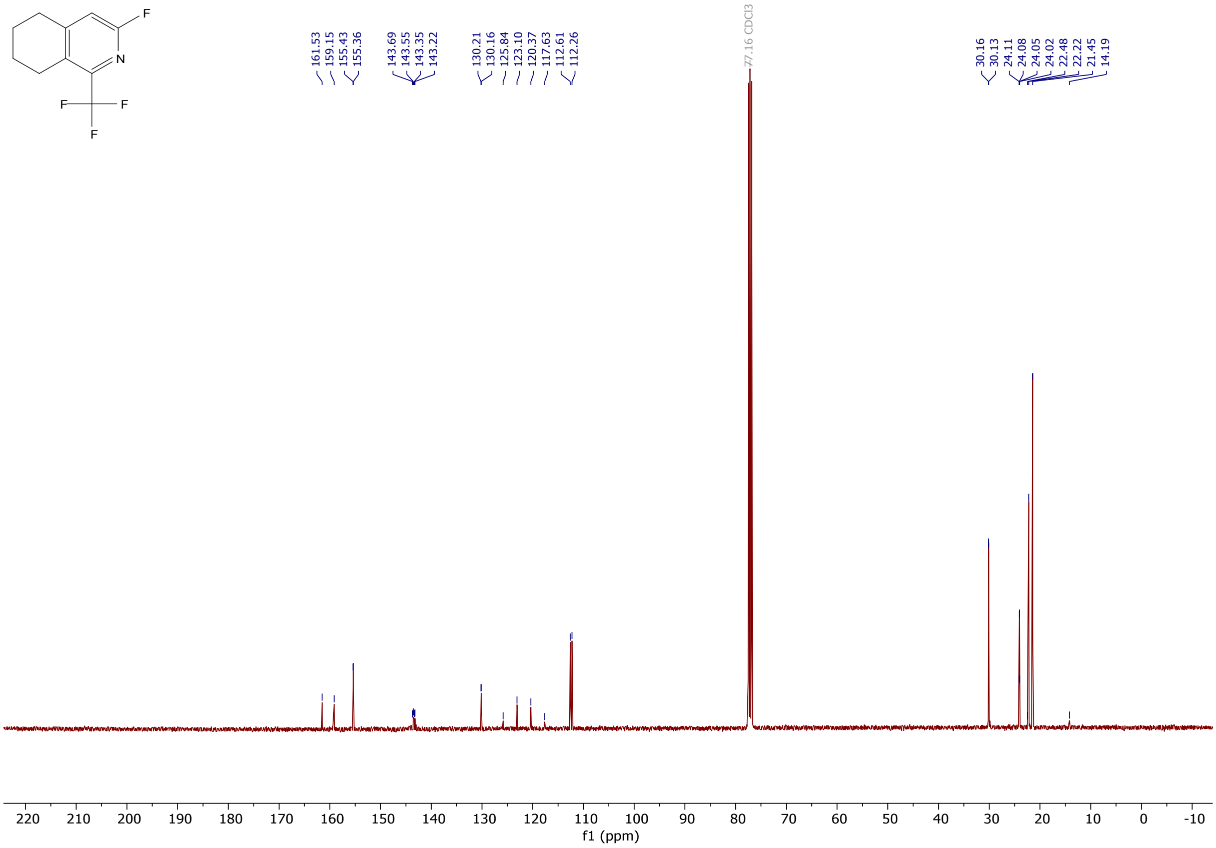

^{19}F NMR spectrum of **11j** ($CDCl_3$, 376 MHz)

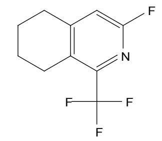

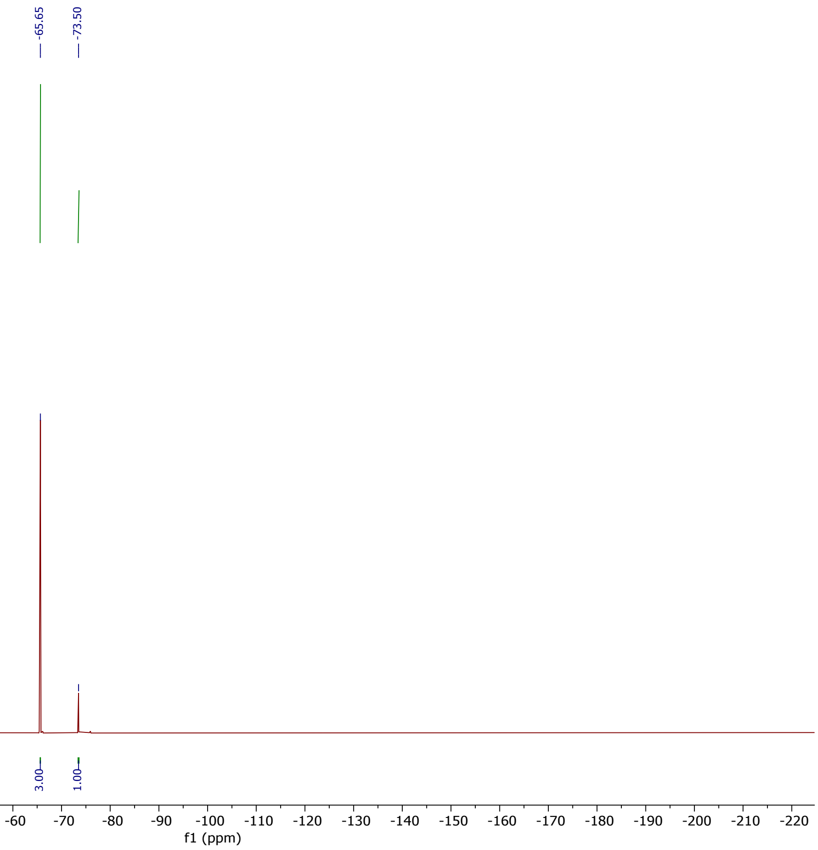

^{1}H NMR spectrum of **11k** ($CDCl_3$, 401 MHz)

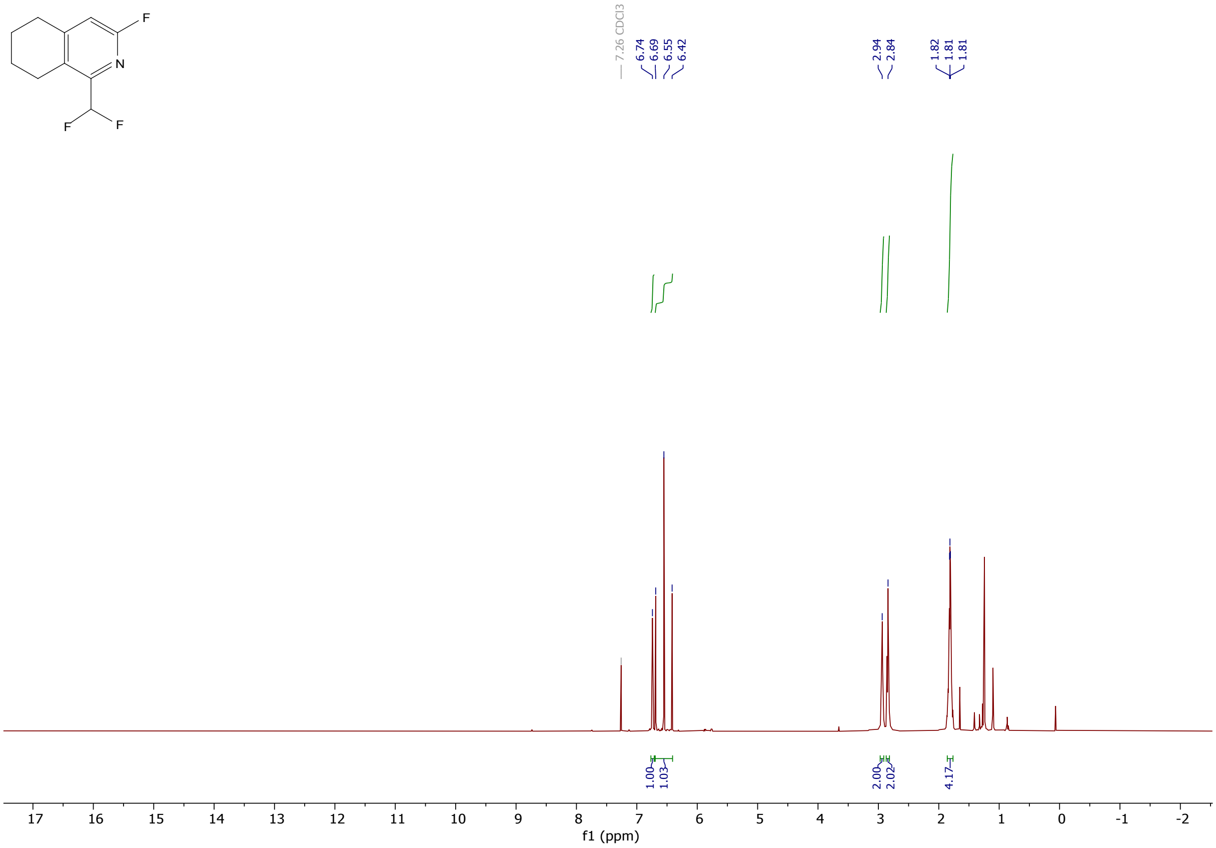

^{13}C NMR spectrum of **11k** ($CDCl_3$, 101 MHz)

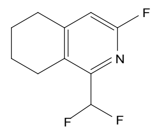

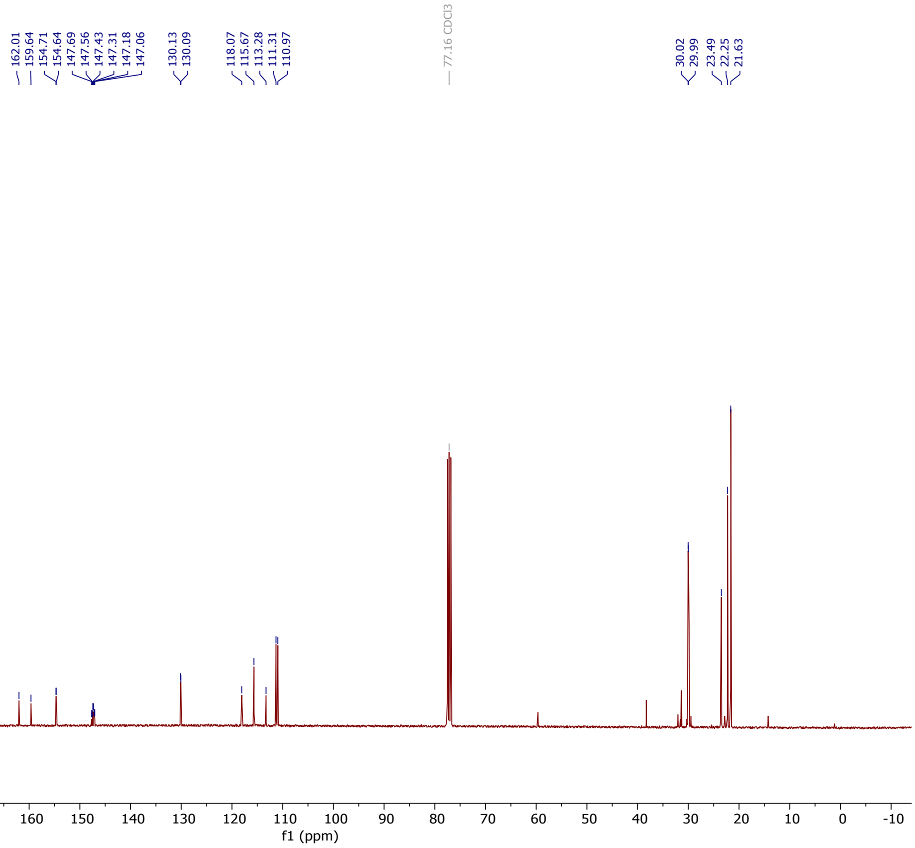

^{19}F NMR spectrum of **11k** ($CDCl_3$, 376 MHz)

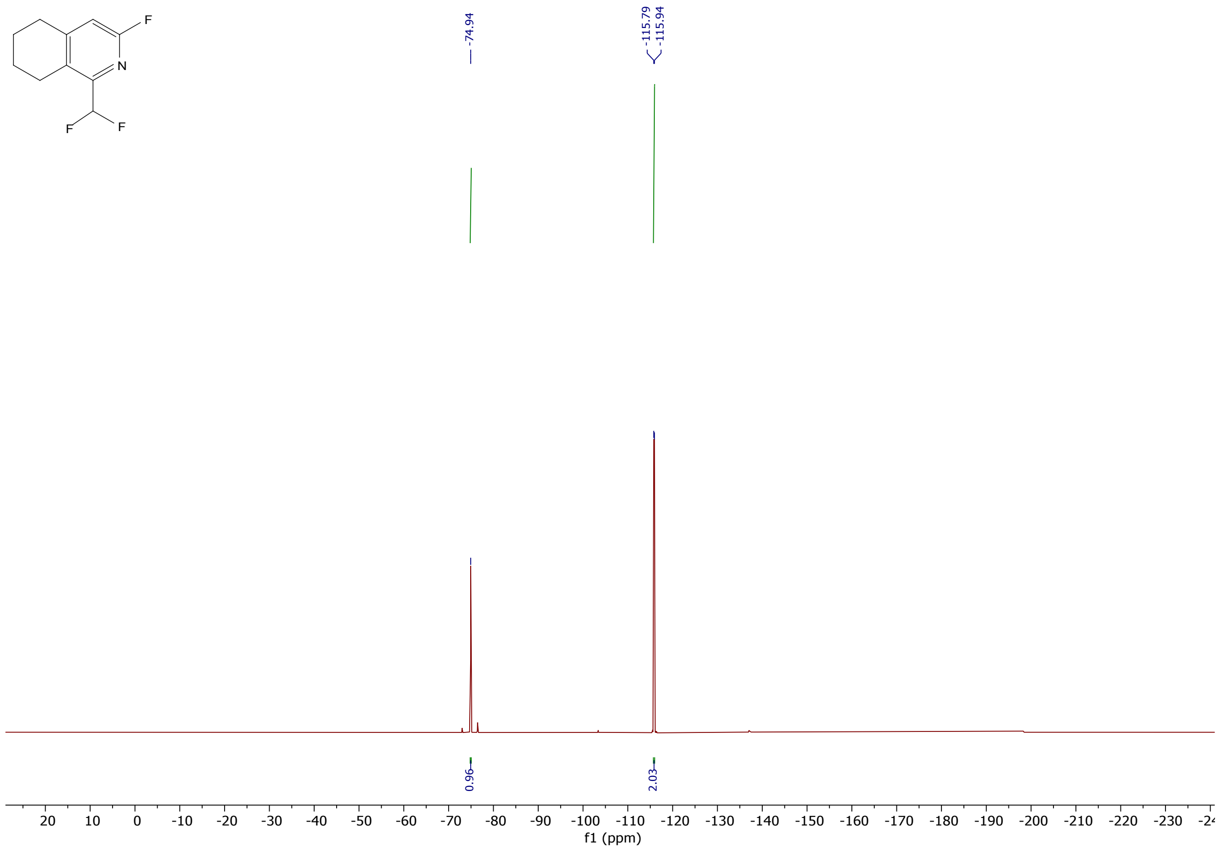

^{1}H NMR spectrum of **11l** ($CDCl_3$, 401 MHz)

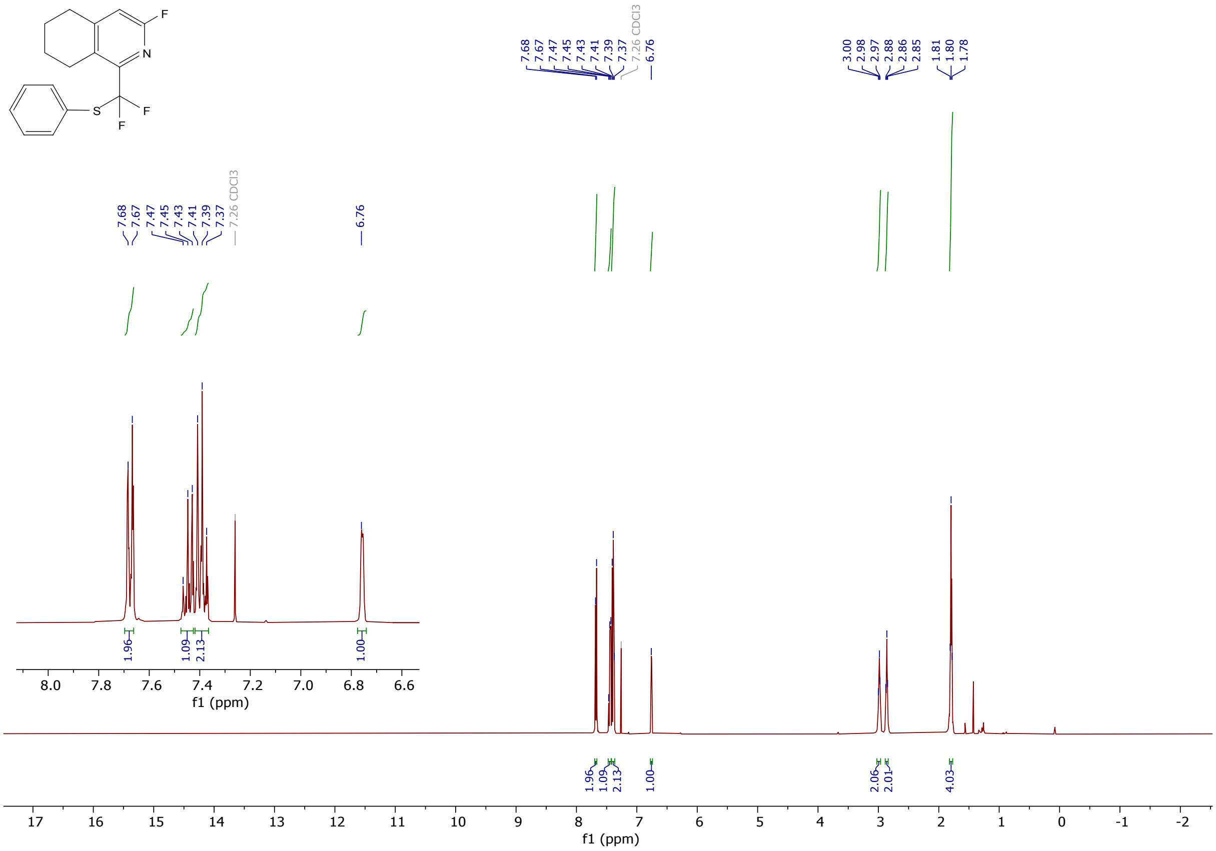

^{13}C NMR spectrum of **11l** ($CDCl_3$, 101 MHz)

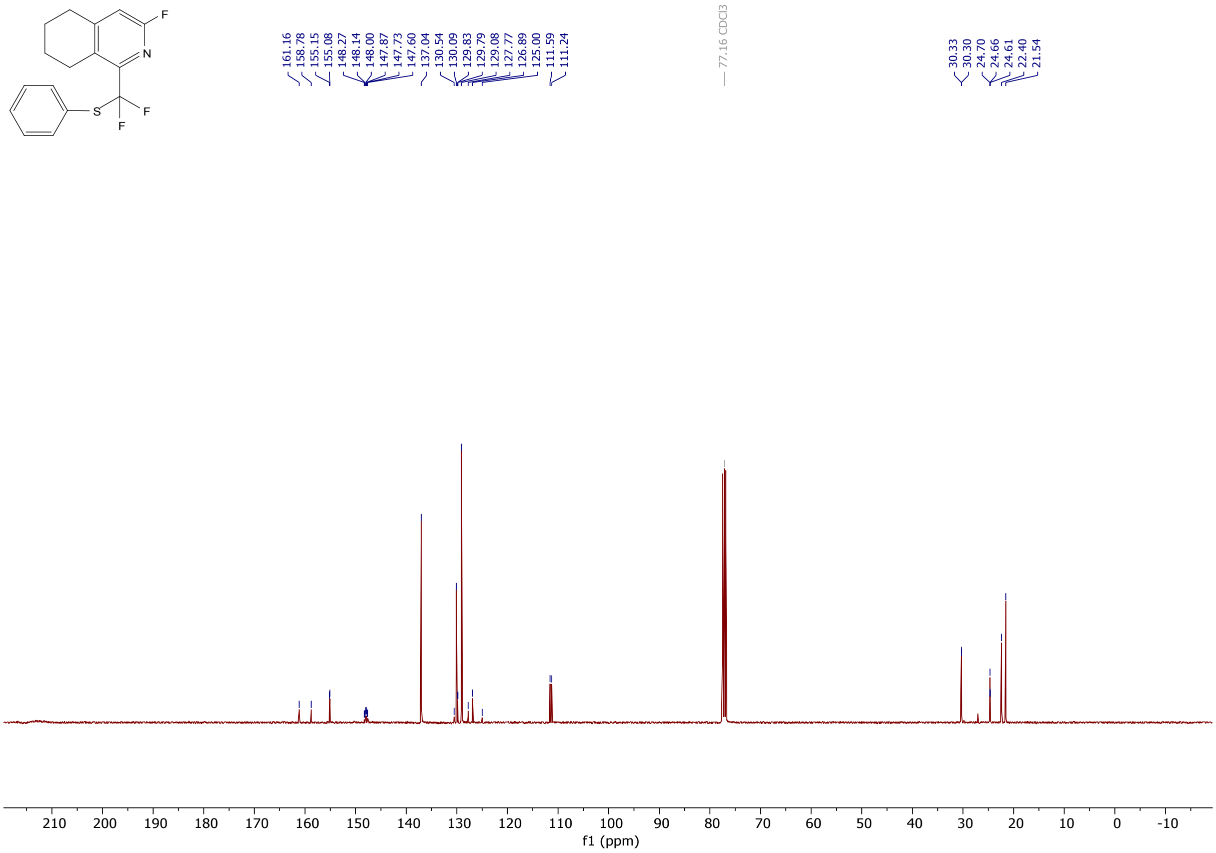

^{19}F NMR spectrum of **11l** ($CDCl_3$, 376 MHz)

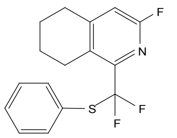

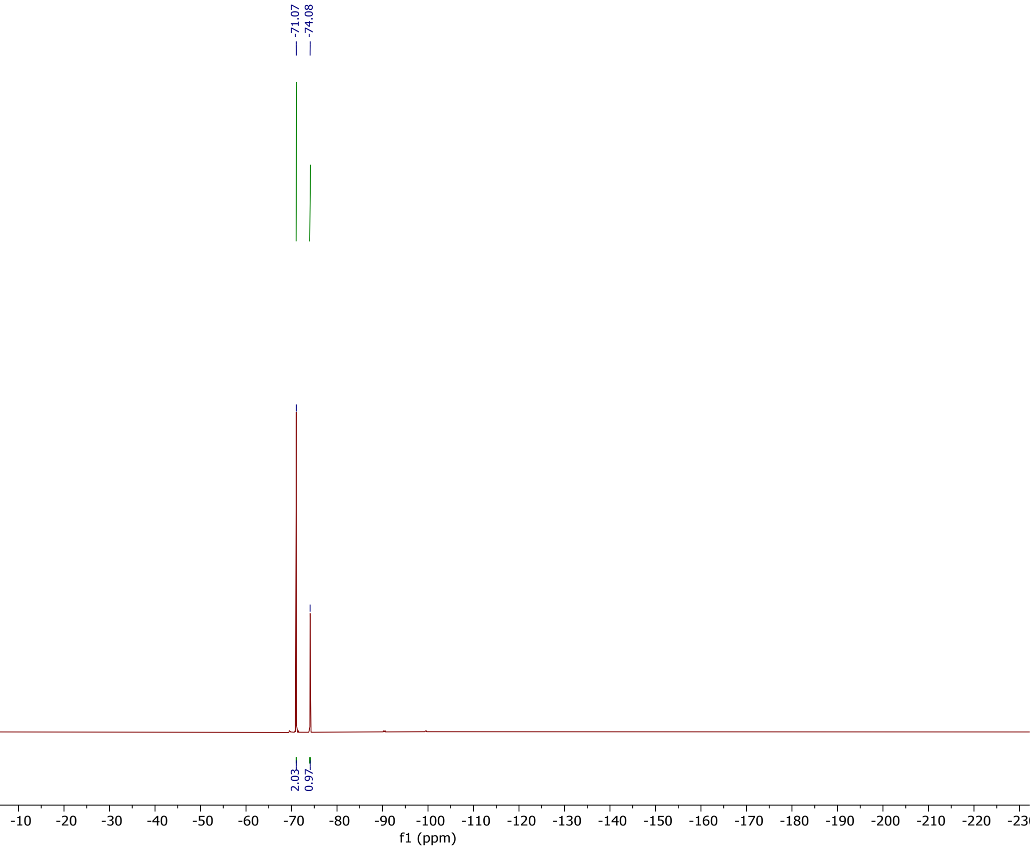

^{1}H NMR spectrum of **11m** ($CDCl_3$, 401 MHz)

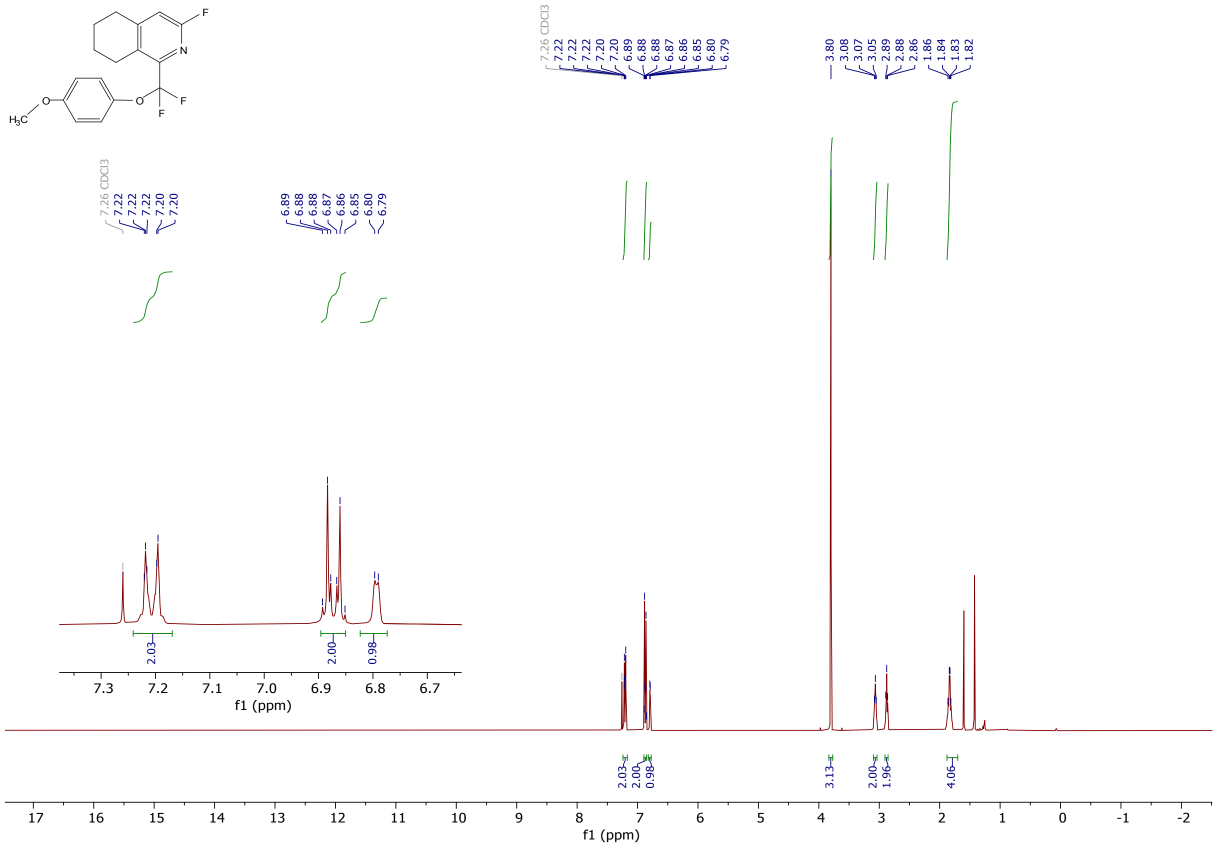

^{13}C NMR spectrum of **11m** ($CDCl_3$, 101 MHz)

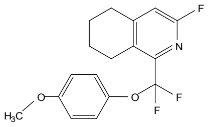

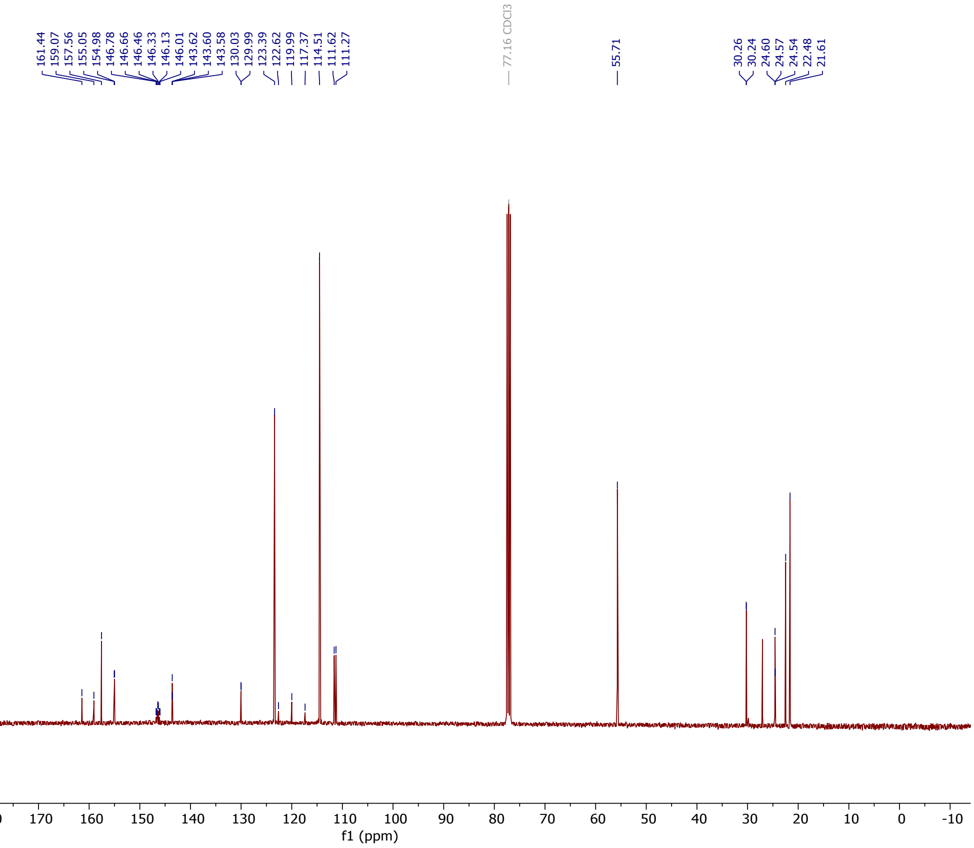

^{19}F NMR spectrum of **11m** ($CDCl_3$, 376 MHz)

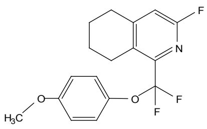

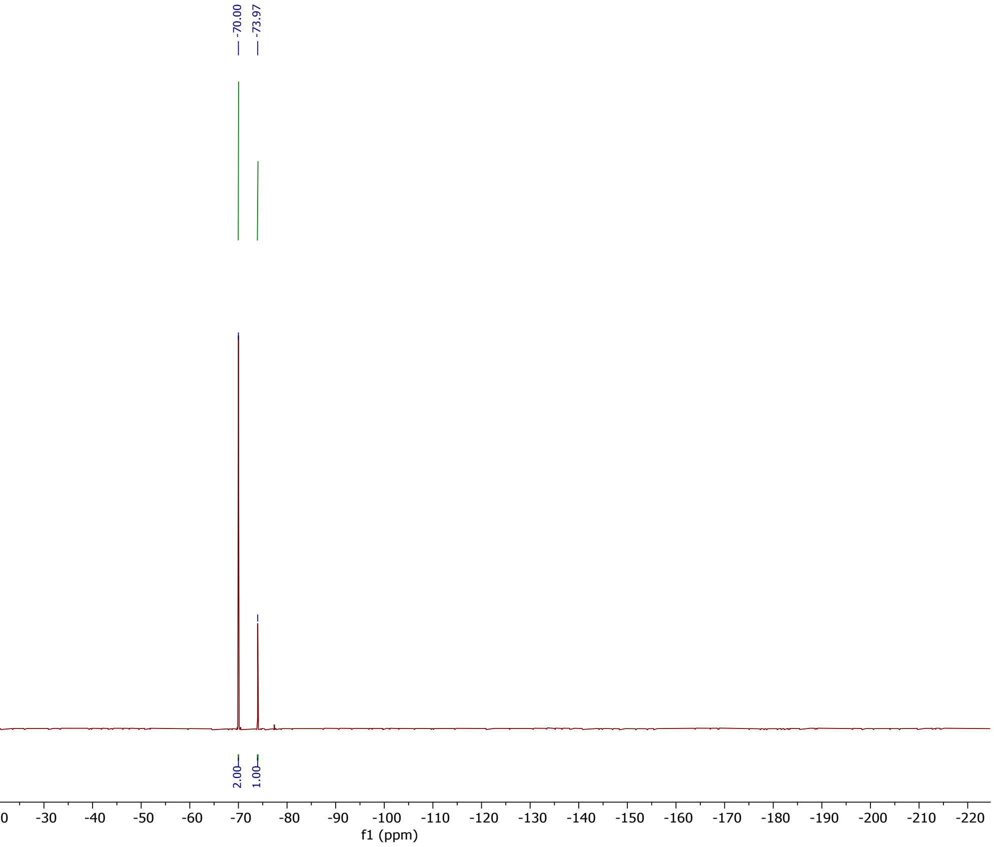

^{1}H NMR spectrum of **11n** ($CDCl_3$, 401 MHz)

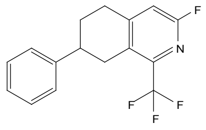

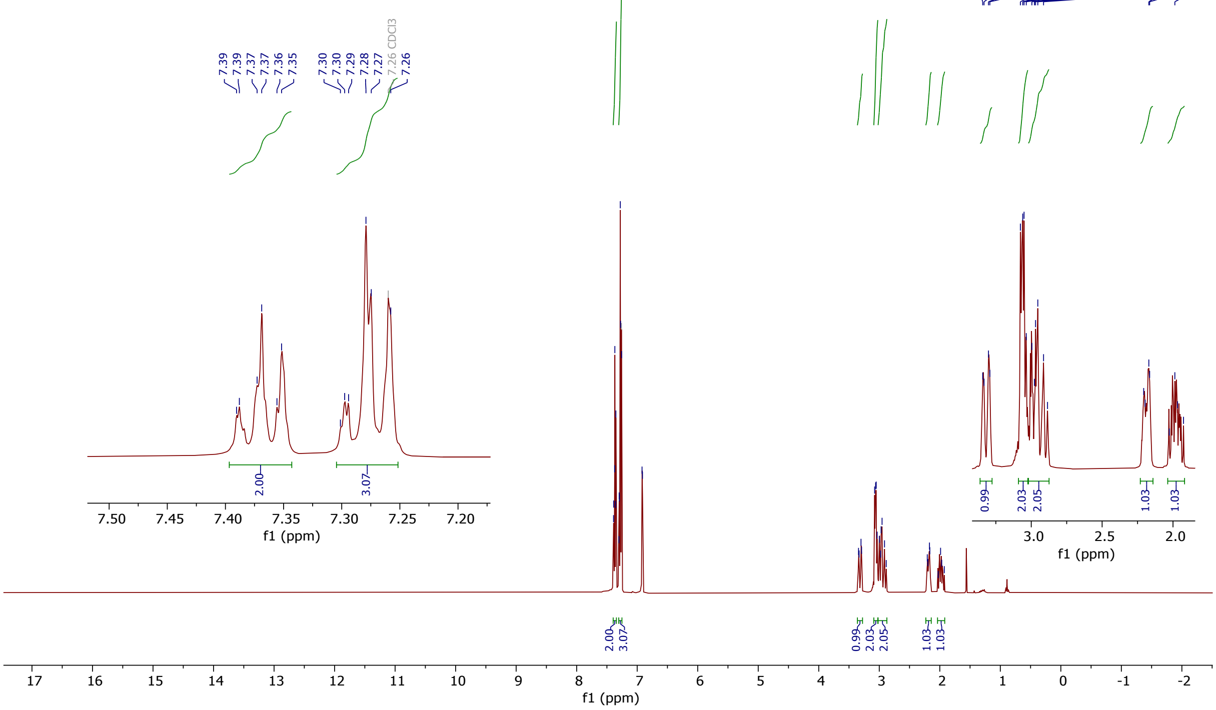

^{13}C NMR spectrum of **11n** ($CDCl_3$, 101 MHz)

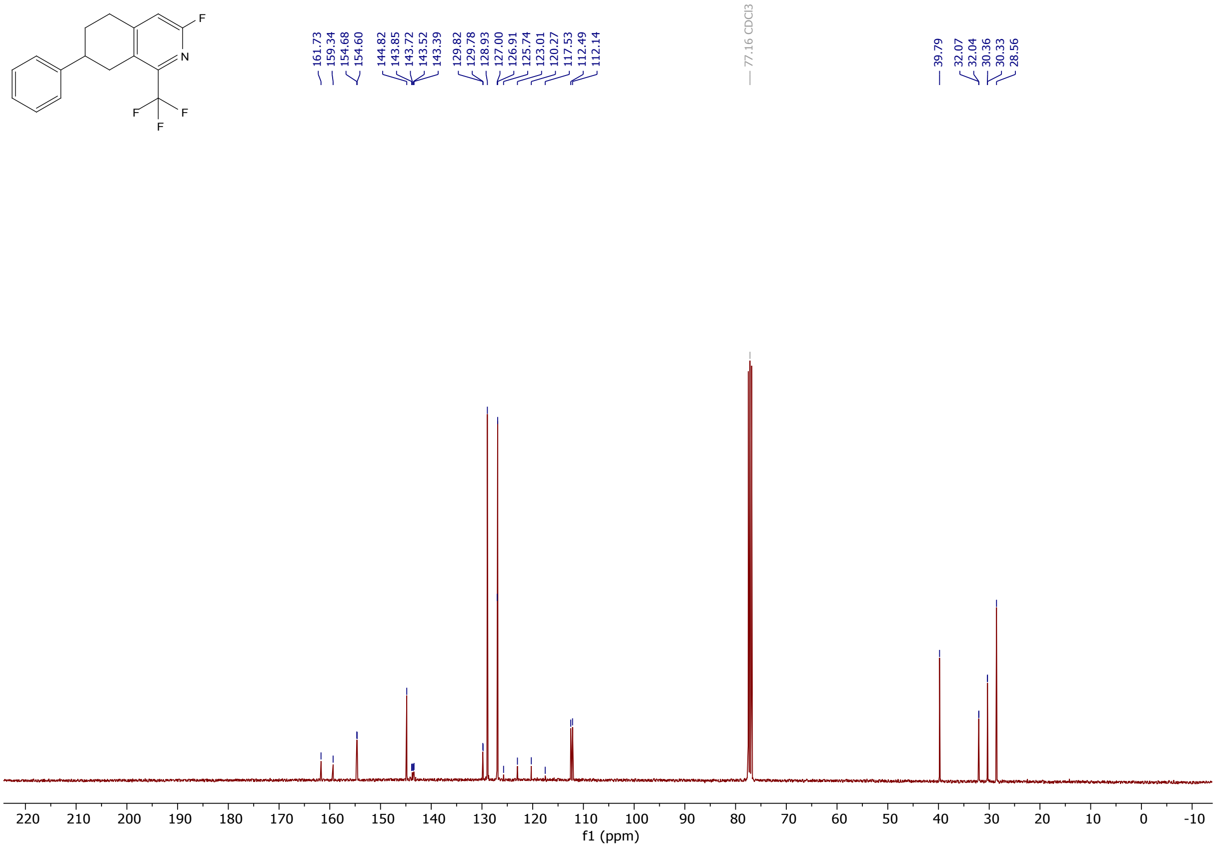

^{19}F NMR spectrum of **11n** ($CDCl_3$, 376 MHz)

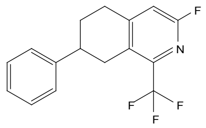

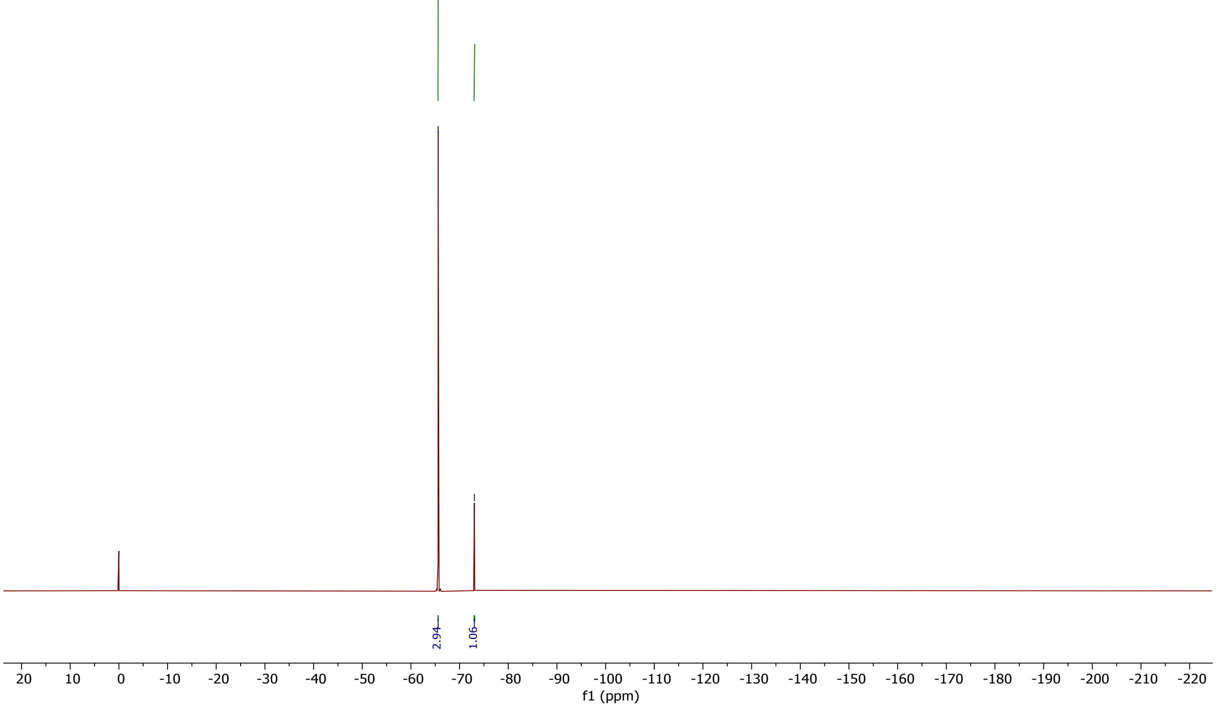

^{1}H NMR spectrum of **11o** ($CDCl_3$, 401 MHz)

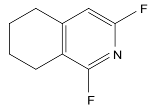

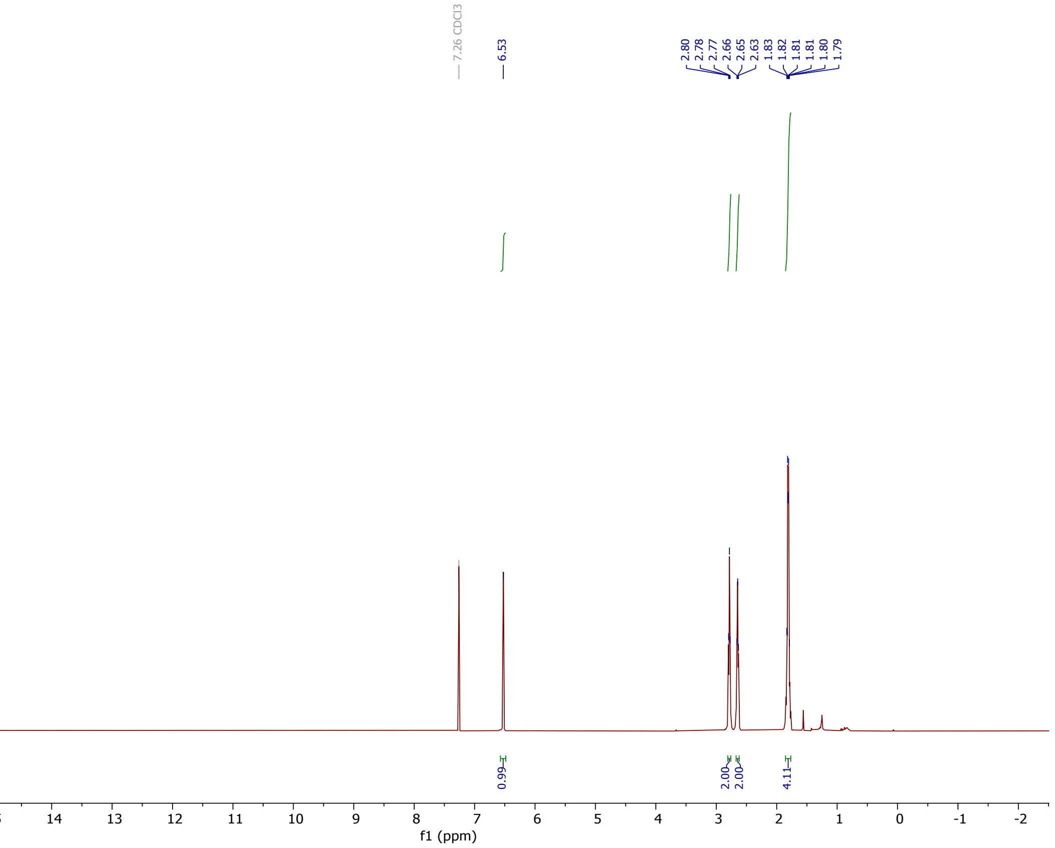

^{13}C NMR spectrum of **11o** ($CDCl_3$, 101 MHz)

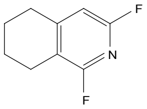

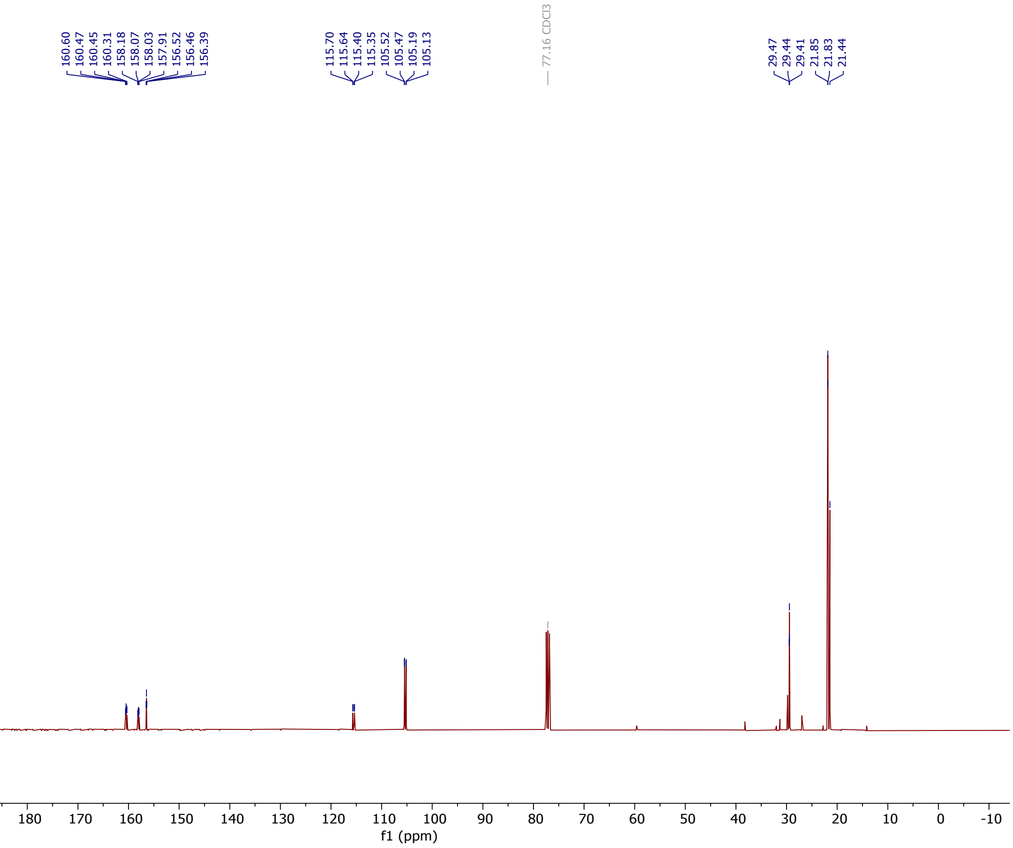

^{19}F NMR spectrum of **11o** ($CDCl_3$, 376 MHz)

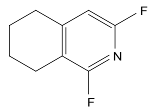

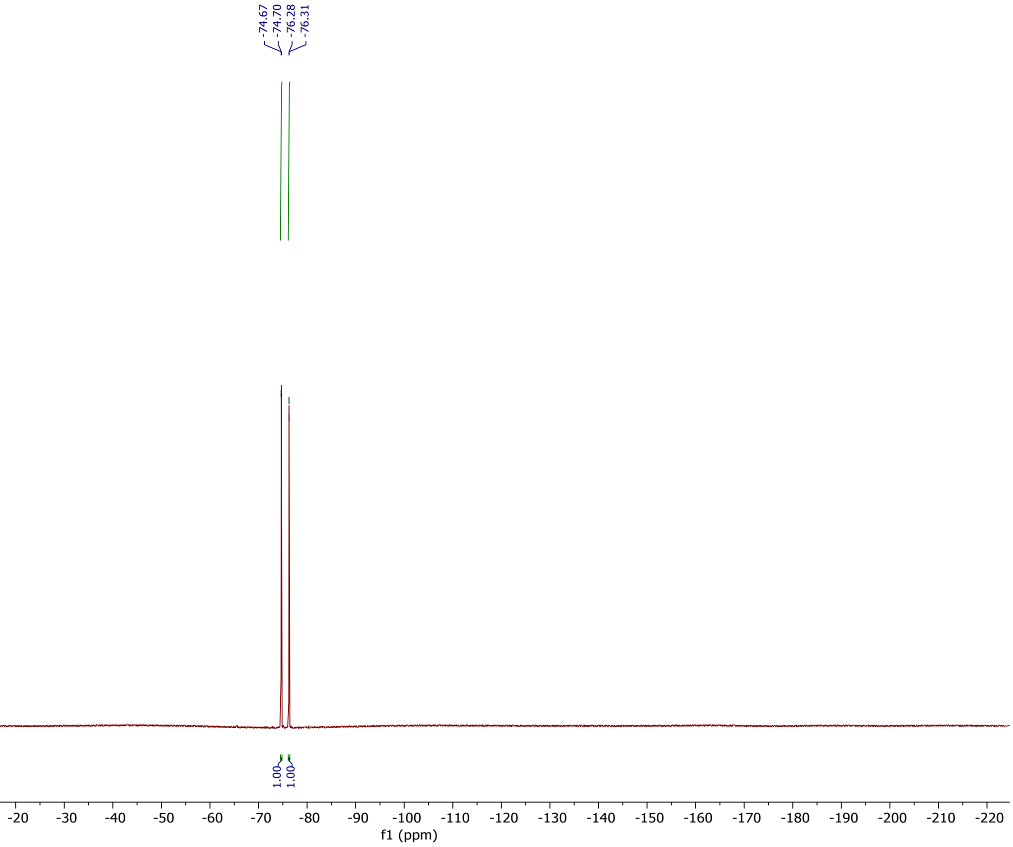

^{1}H NMR spectrum of **11p** ($CDCl_3$, 401 MHz)

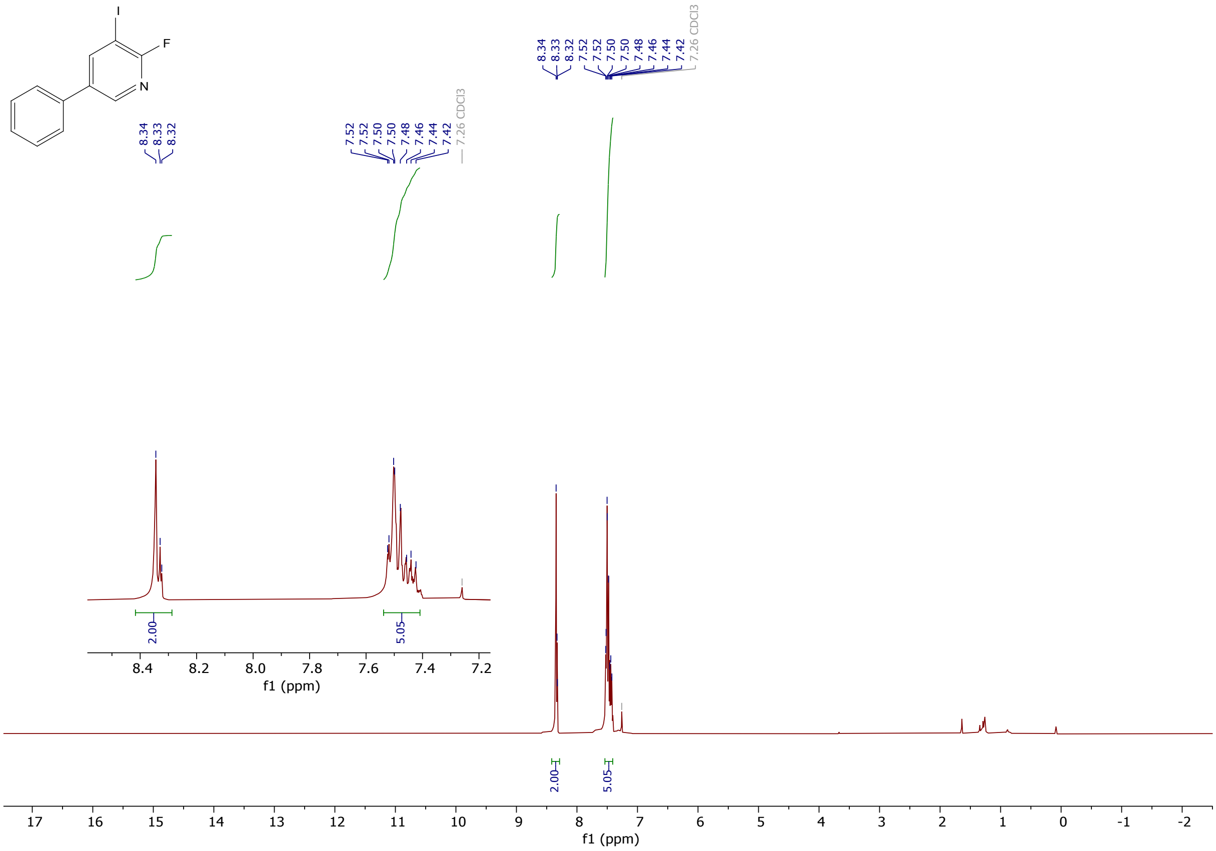

^{13}C NMR spectrum of **11p** (CDCl$_3$, 101 MHz)

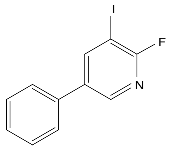

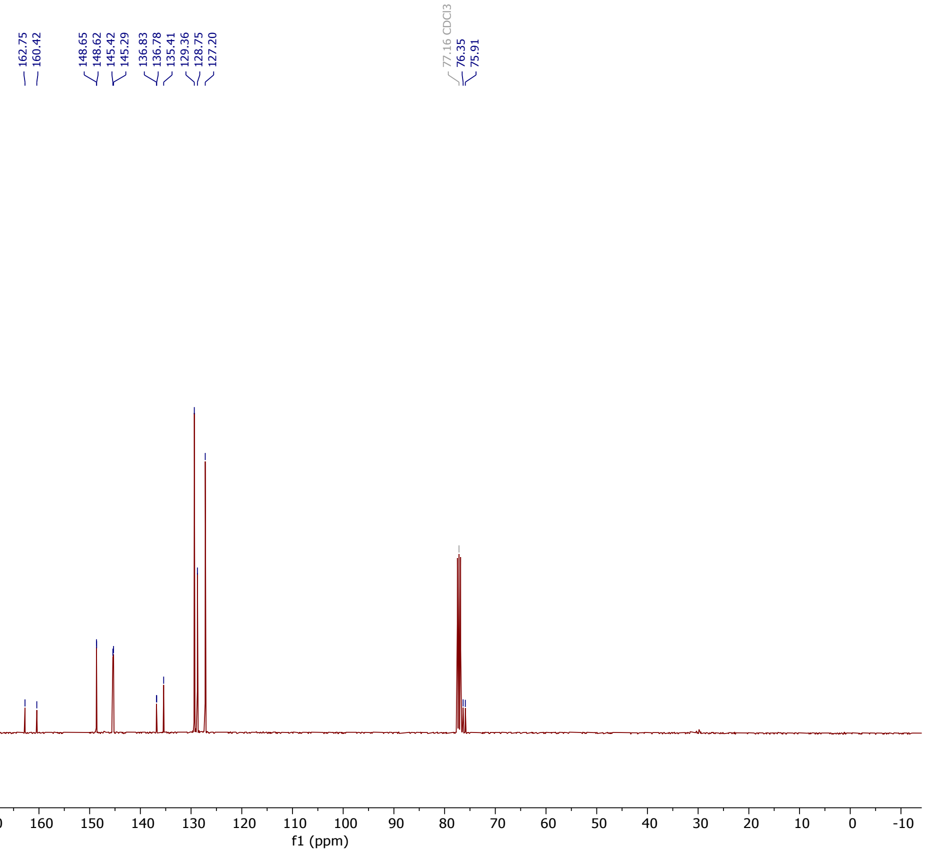

^{19}F NMR spectrum of **11p** ($CDCl_3$, 376 MHz)

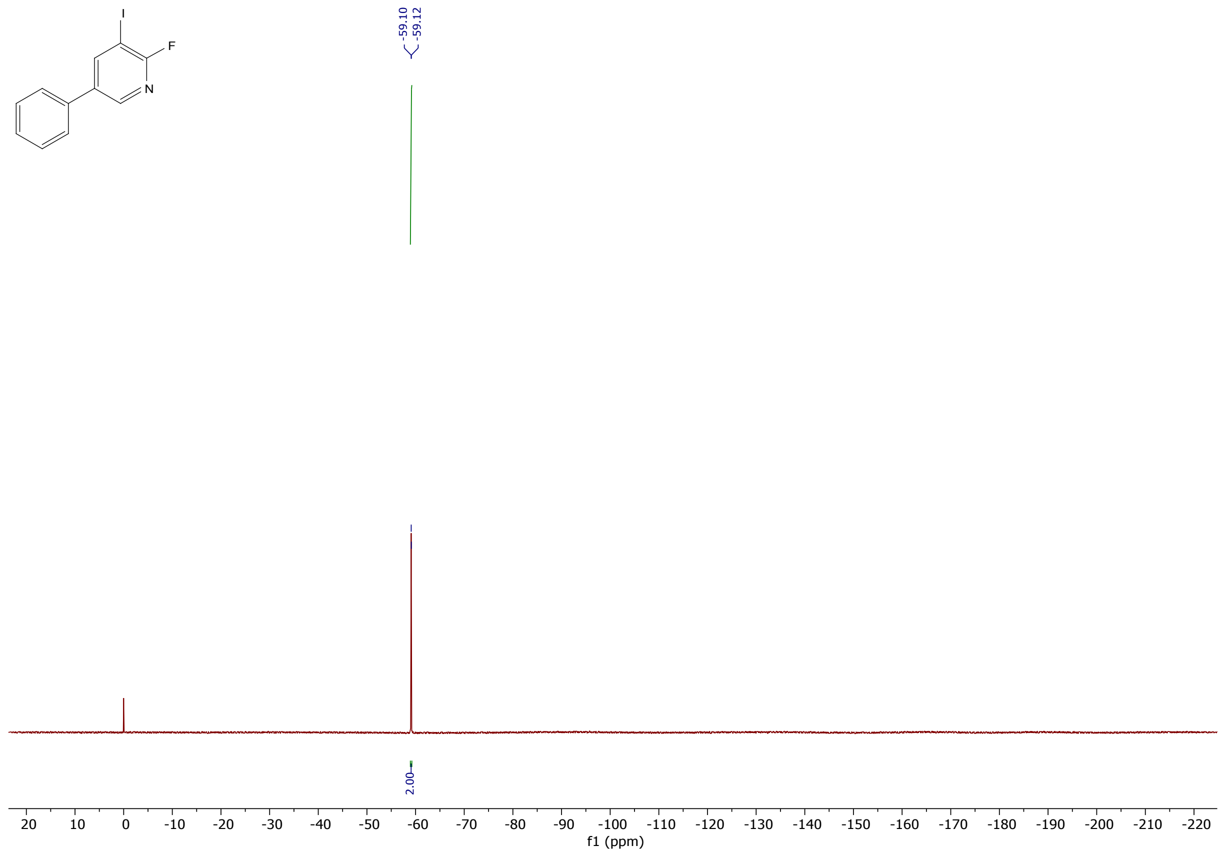

^{1}H NMR spectrum of **12a** ($CDCl_3$, 401 MHz)

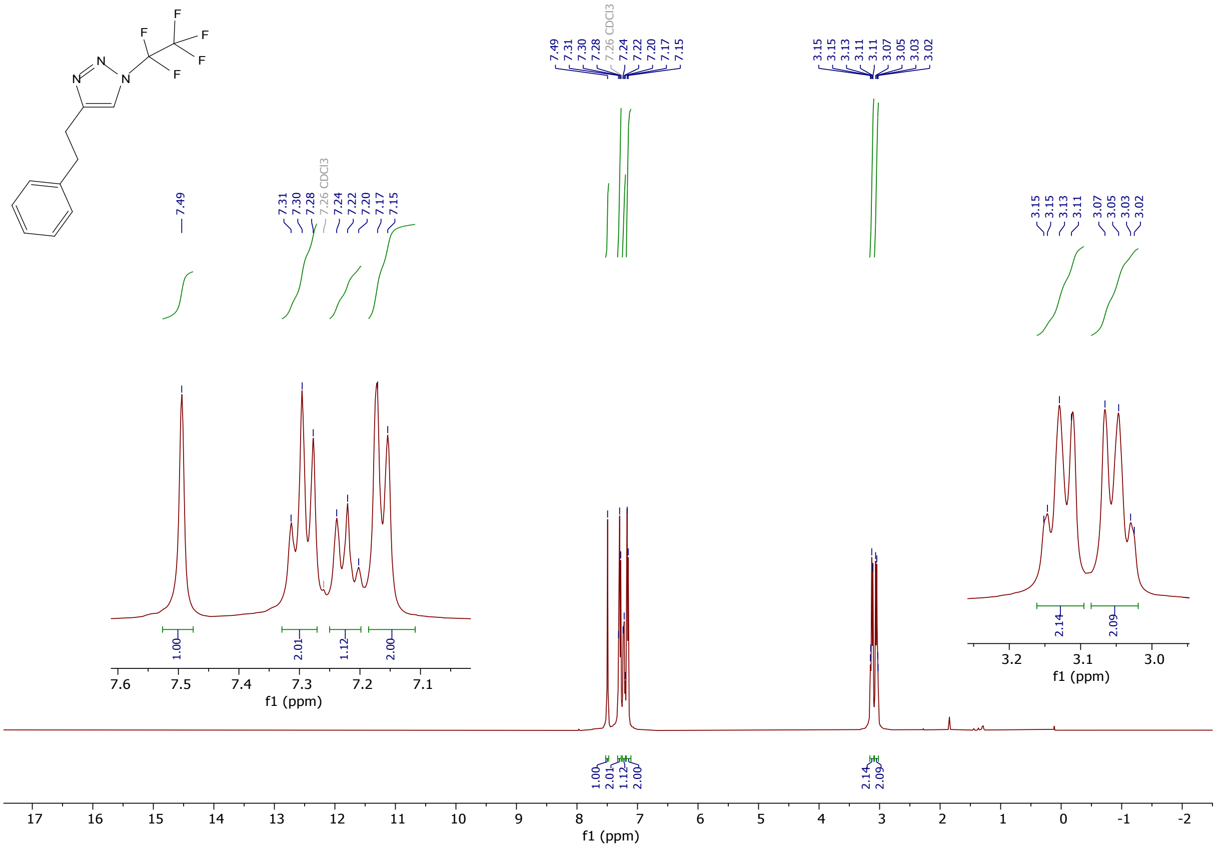

^{13}C NMR spectrum of **12a** ($CDCl_3$, 101 MHz)

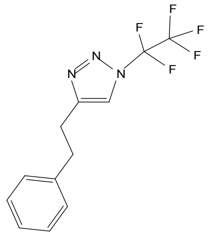

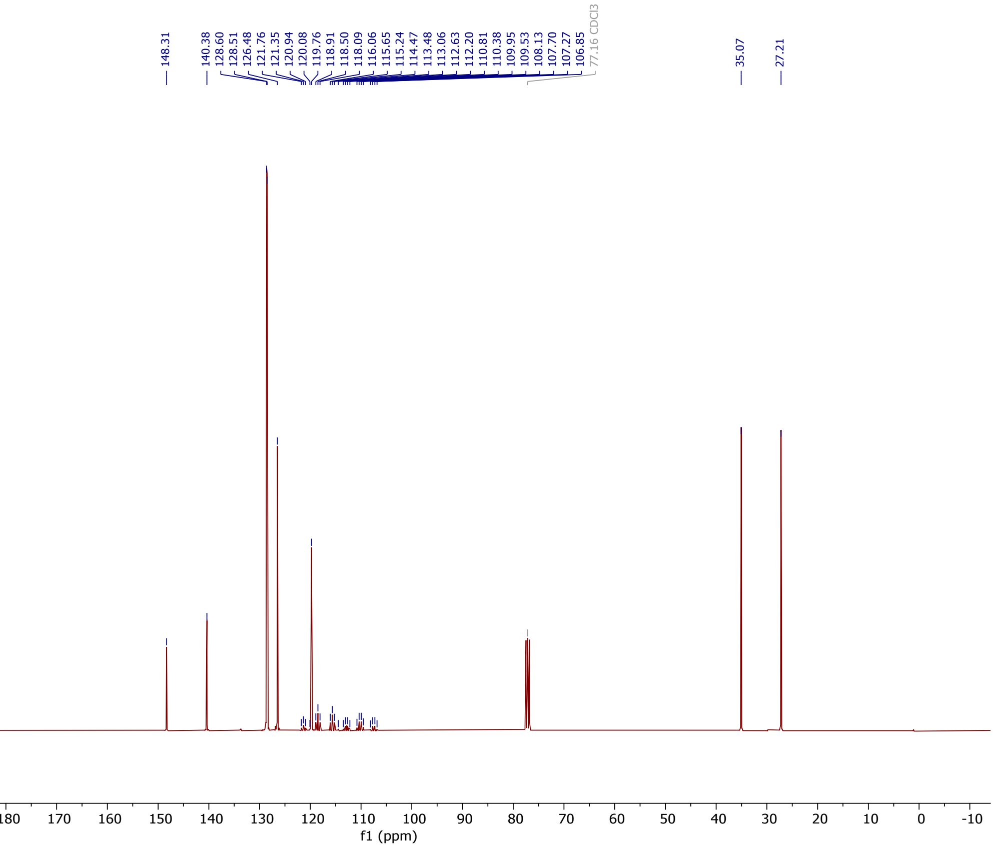

^{19}F NMR spectrum of **12a** ($CDCl_3$, 376 MHz)

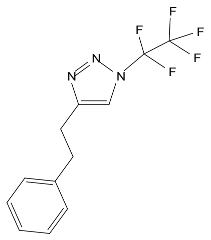

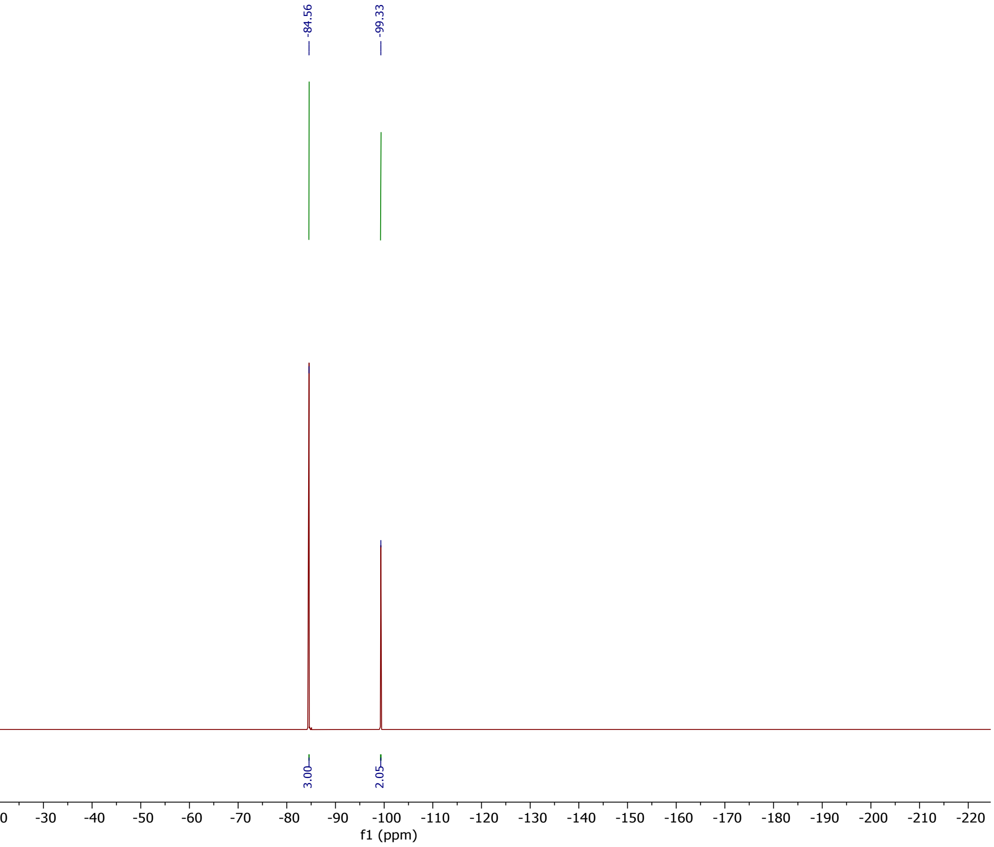

^{1}H NMR spectrum of **12d** ($CDCl_3$, 401 MHz)

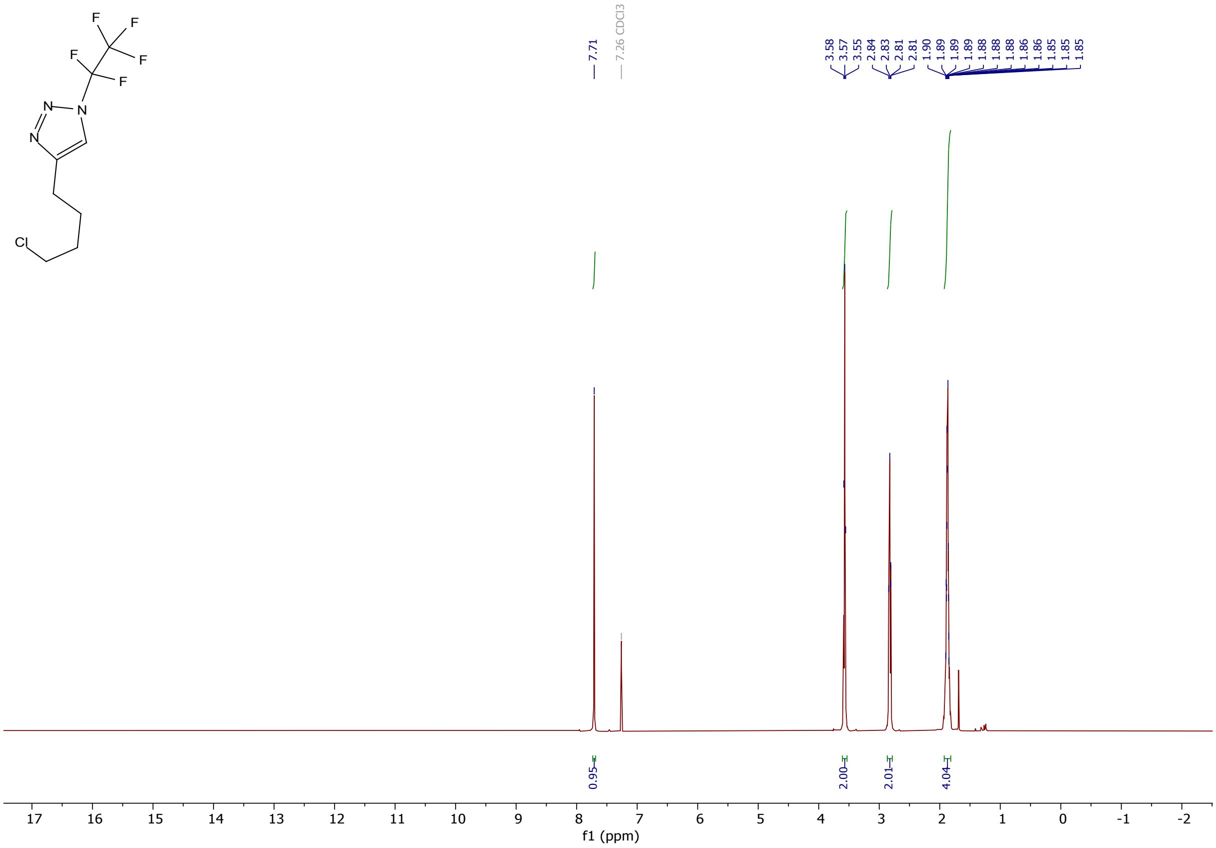

^{13}C NMR spectrum of **12d** ($CDCl_3$, 101 MHz)

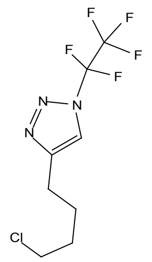

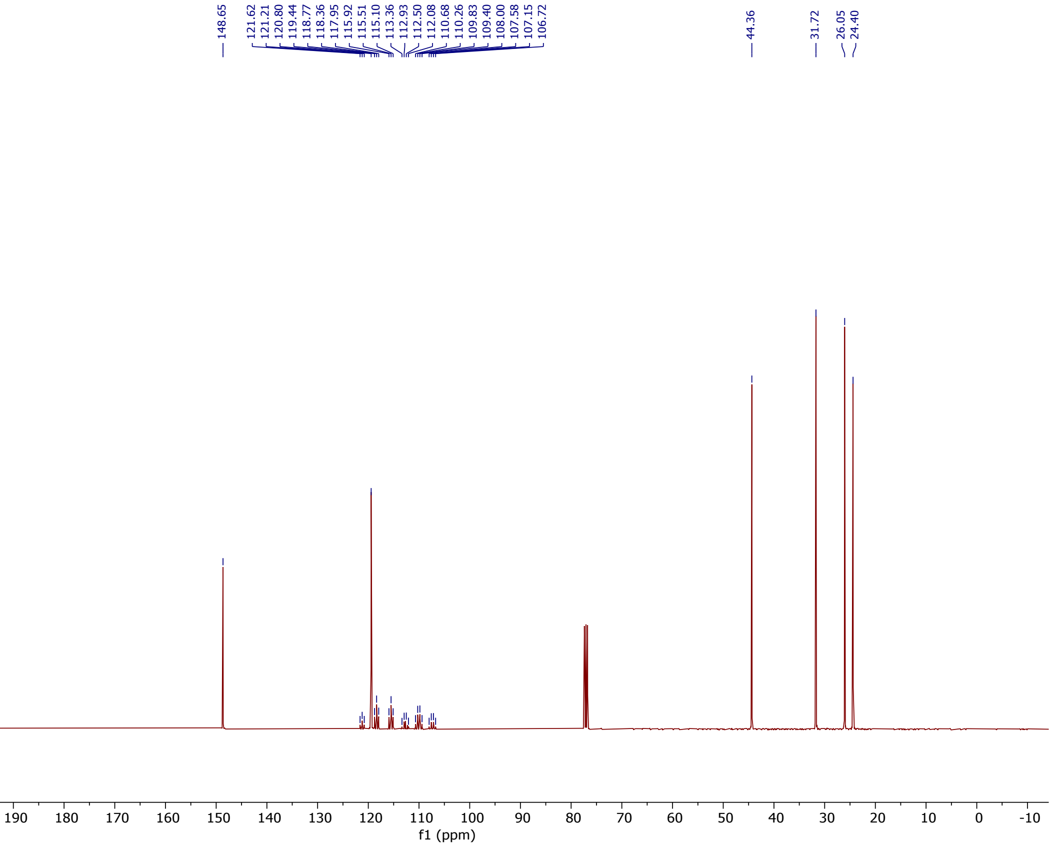

^{19}F NMR spectrum of **12d** ($CDCl_3$, 376 MHz)

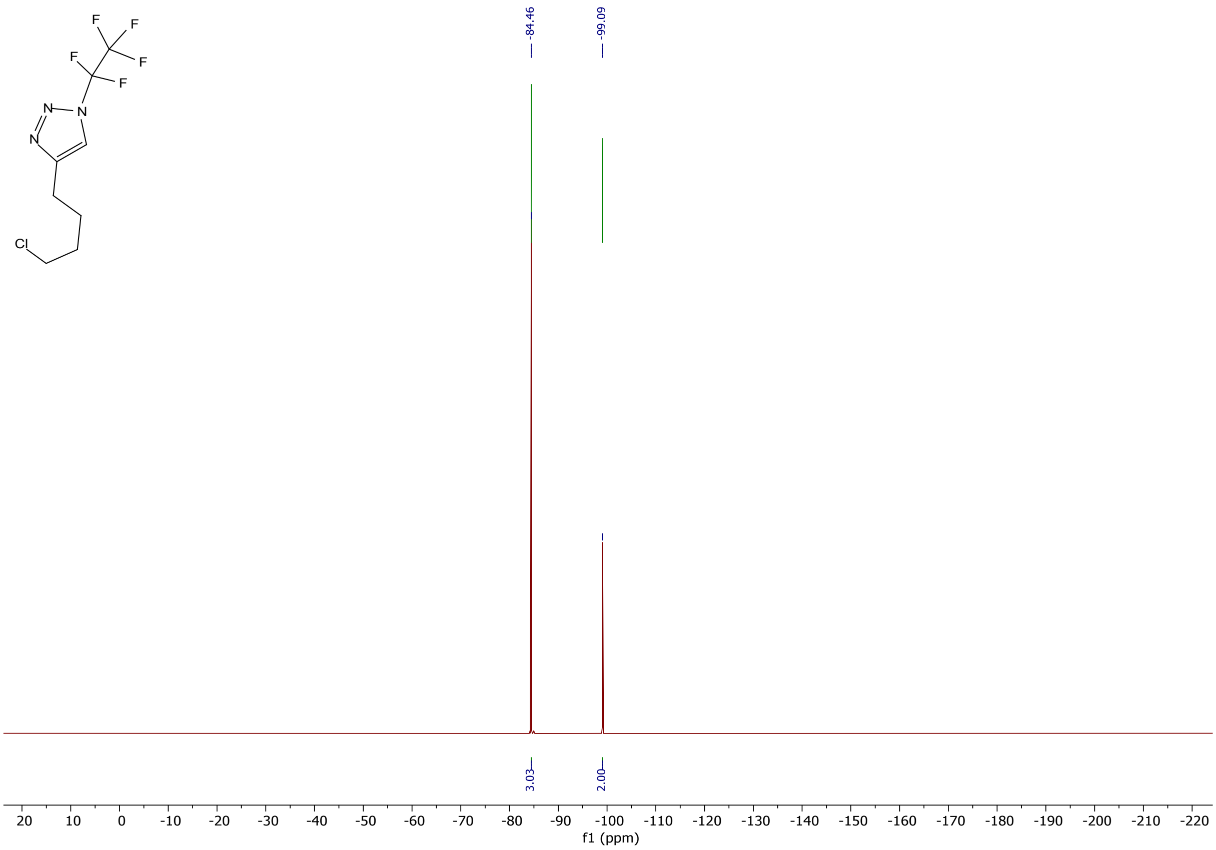

^{1}H NMR spectrum of **12e** ($CDCl_3$, 401 MHz)

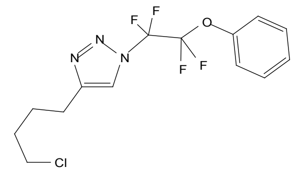

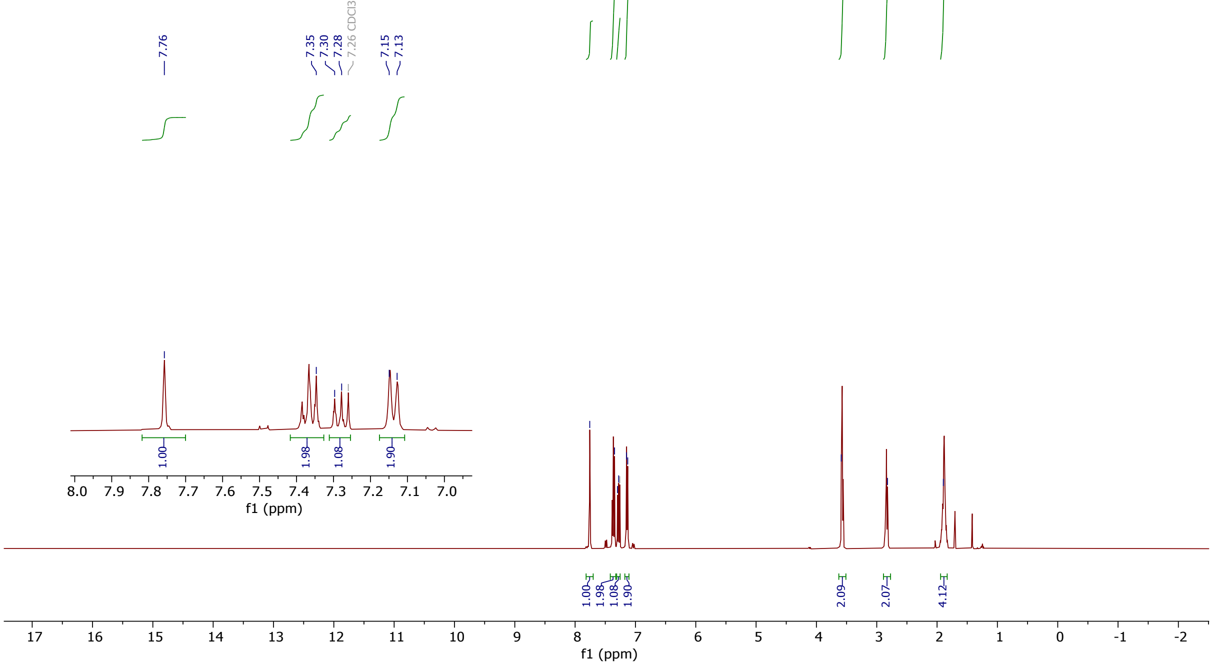

^{13}C NMR spectrum of **12e** ($CDCl_3$, 101 MHz)

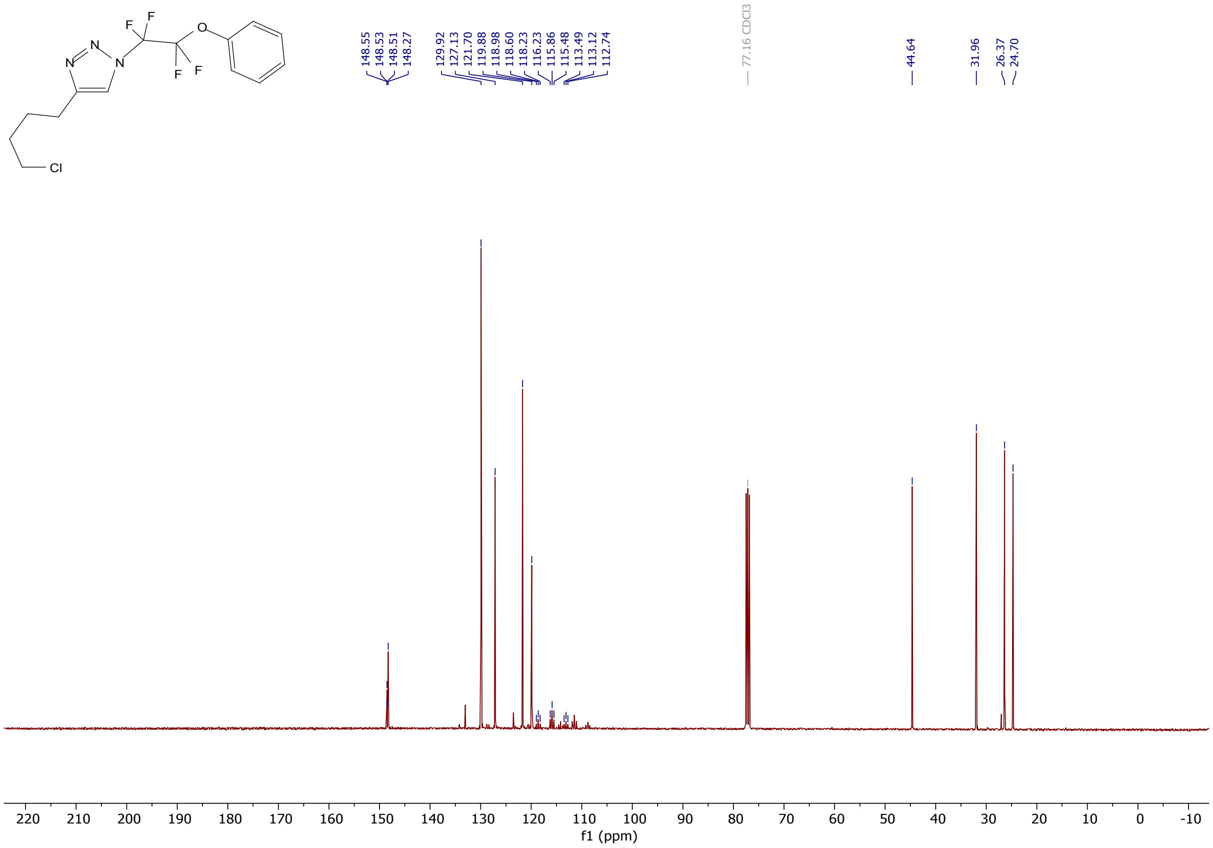

^{19}F NMR spectrum of **12e** ($CDCl_3$, 376 MHz)

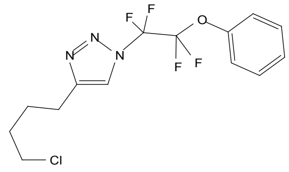

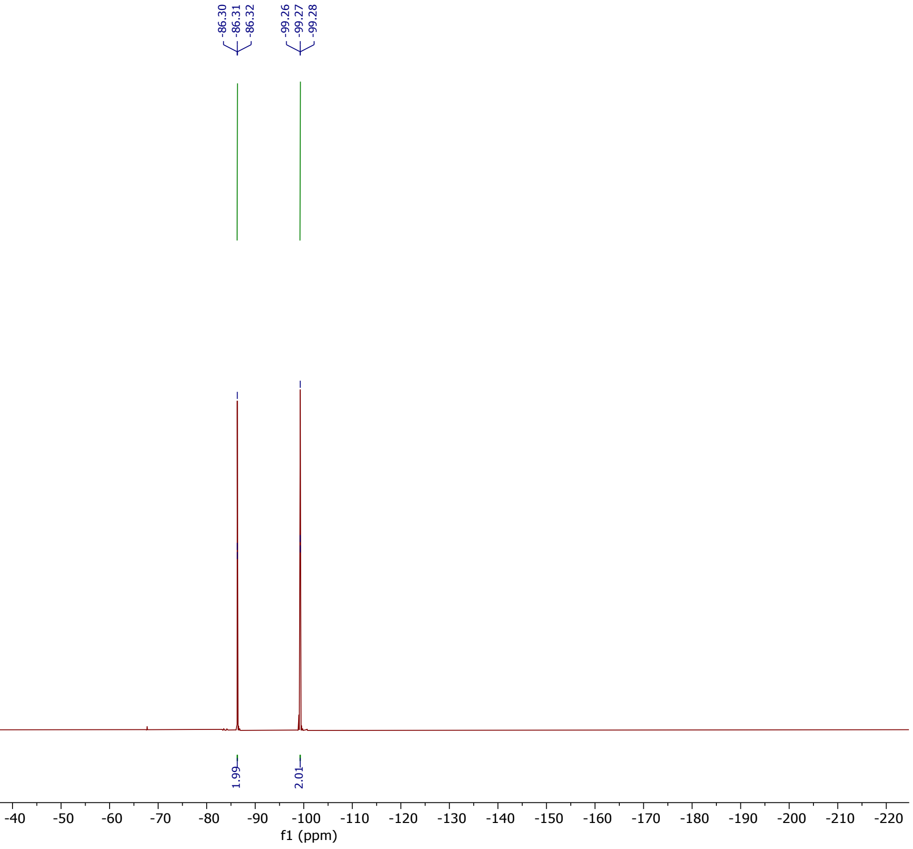

^{1}H NMR spectrum of **12f** ($CDCl_3$, 401 MHz)

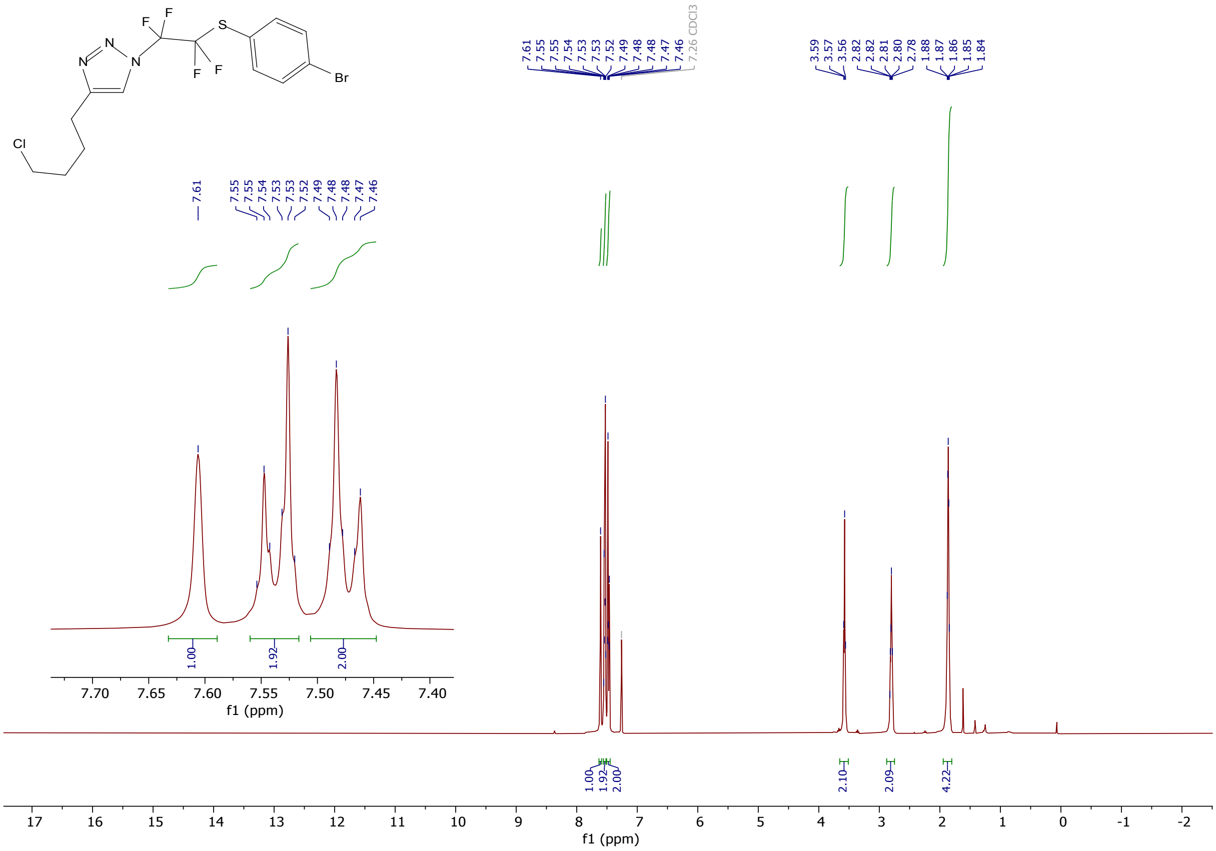

^{13}C NMR spectrum of **12f** ($CDCl_3$, 101 MHz)

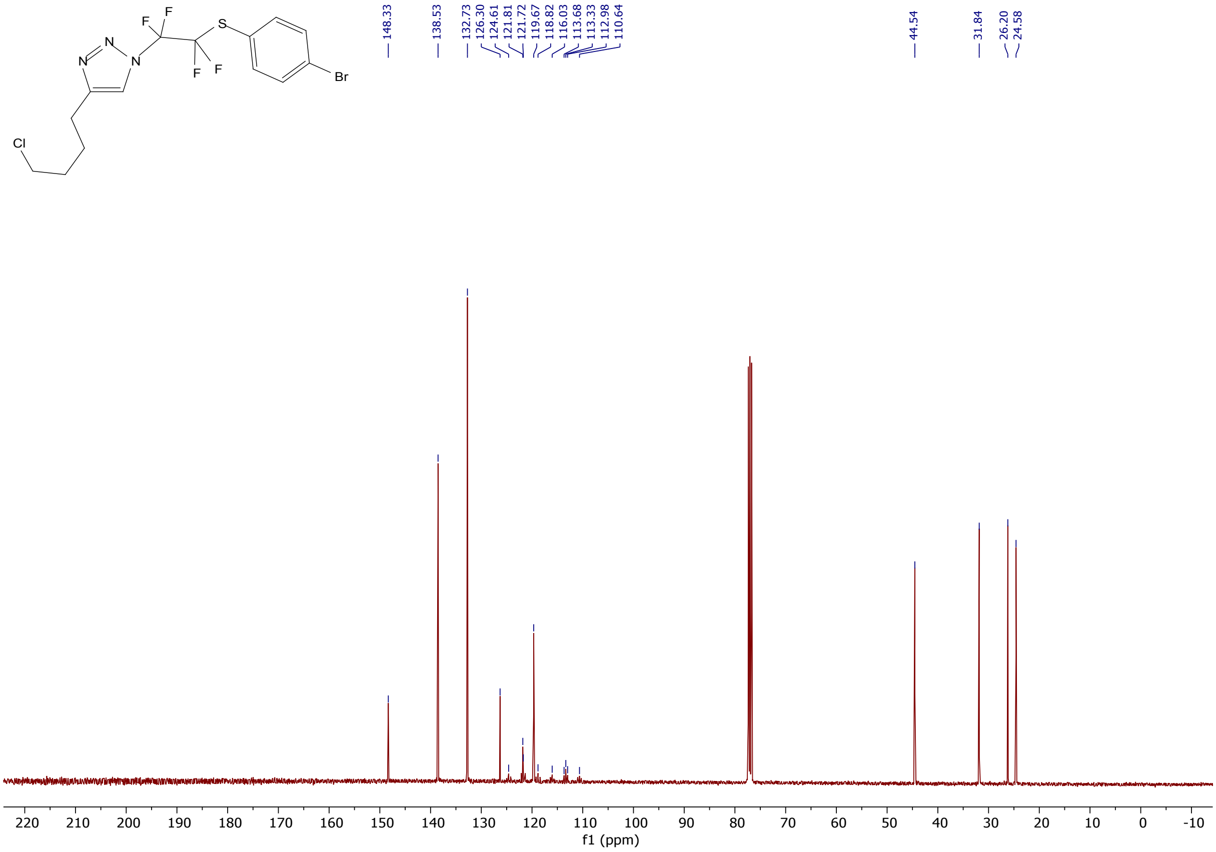

^{19}F NMR spectrum of **12f** ($CDCl_3$, 376 MHz)

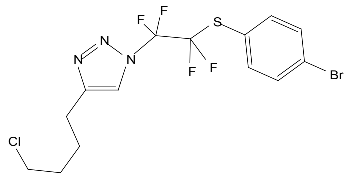

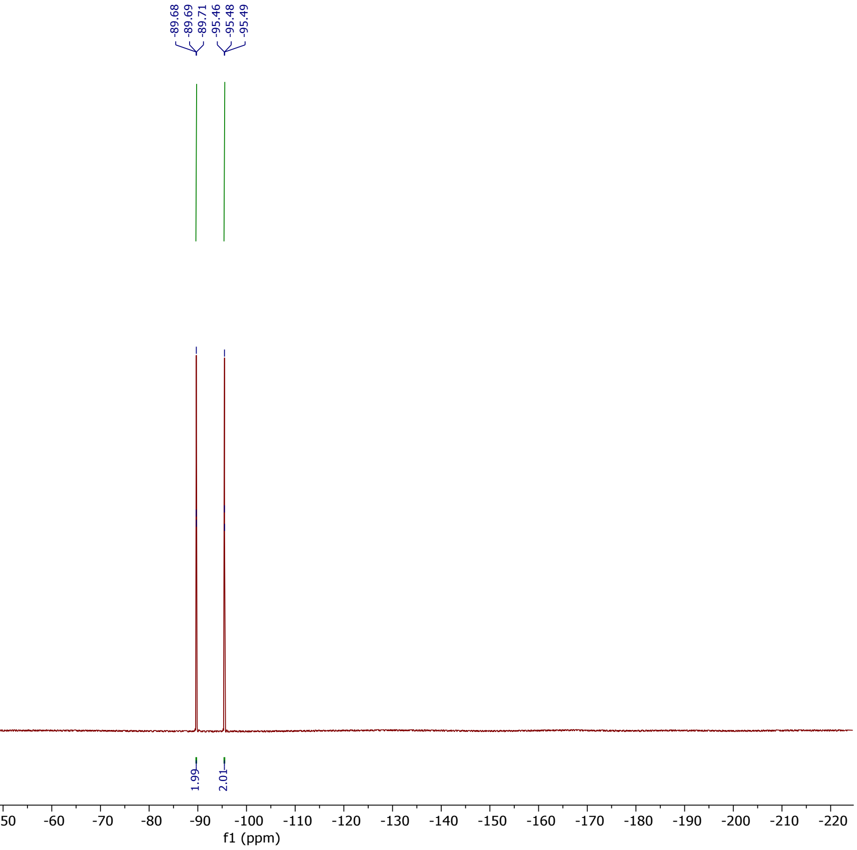

^{1}H NMR spectrum of **12g** ($CDCl_3$, 401 MHz)

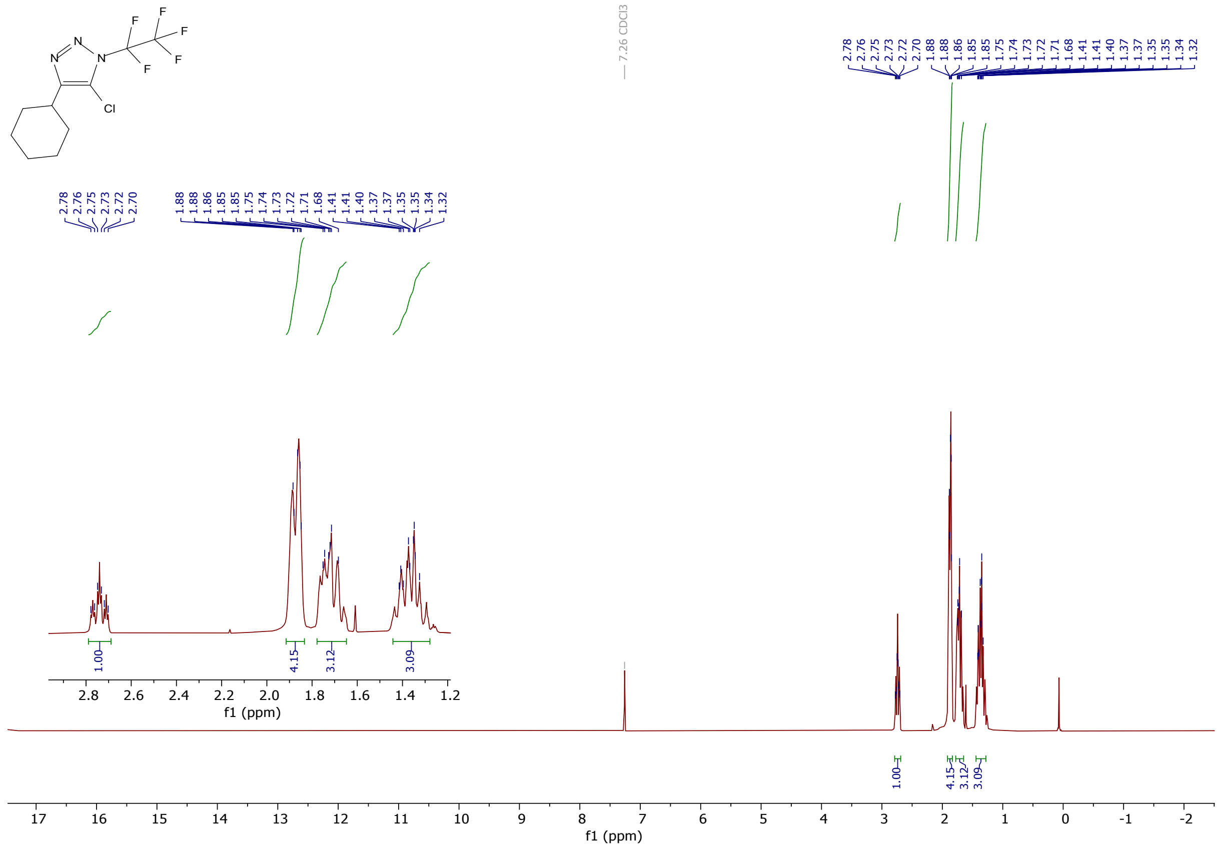

^{13}C NMR spectrum of **12g** ($CDCl_3$, 101 MHz)

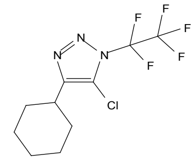

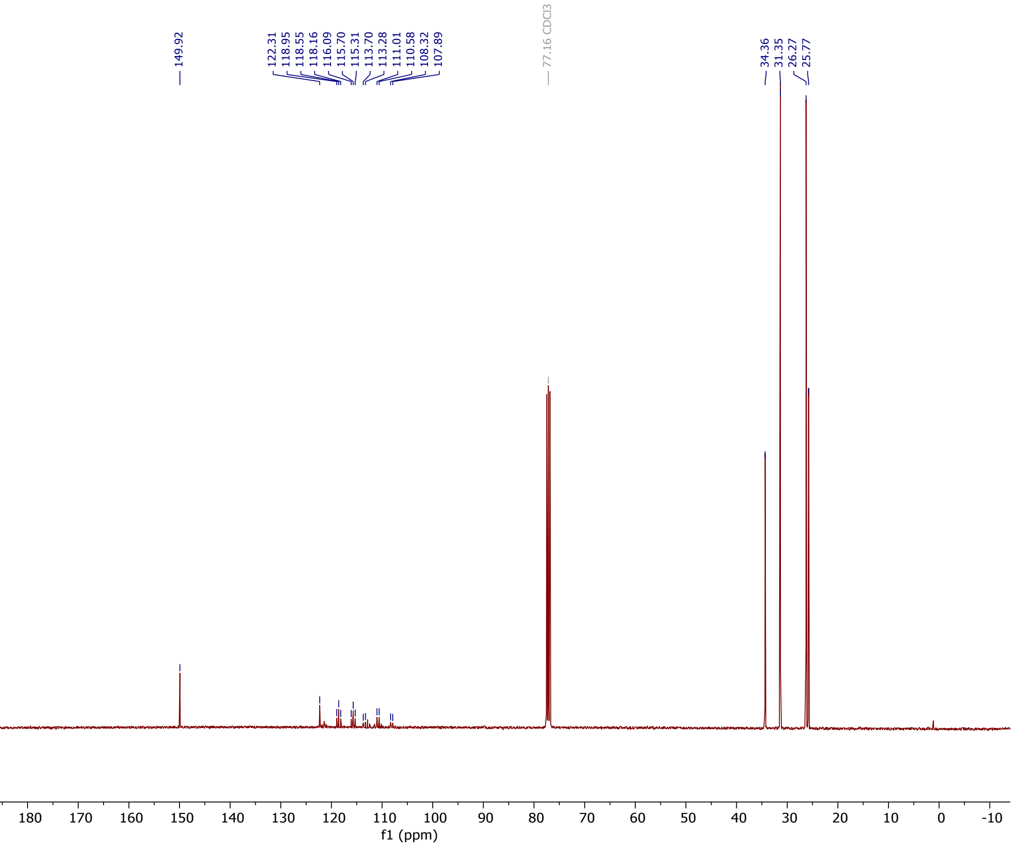

^{19}F NMR spectrum of **12g** ($CDCl_3$, 376 MHz)

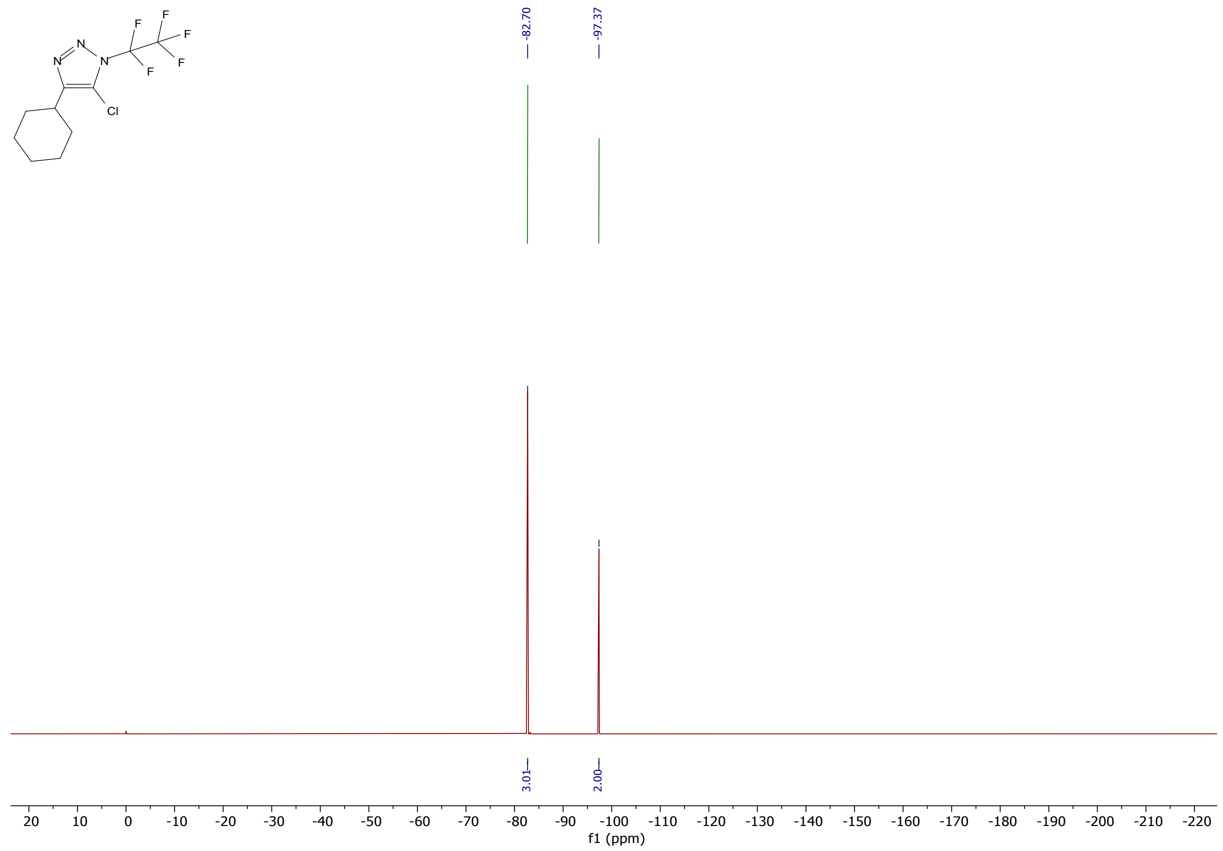

^{1}H NMR spectrum of **12h** ($CDCl_3$, 401 MHz)

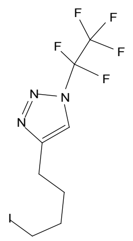

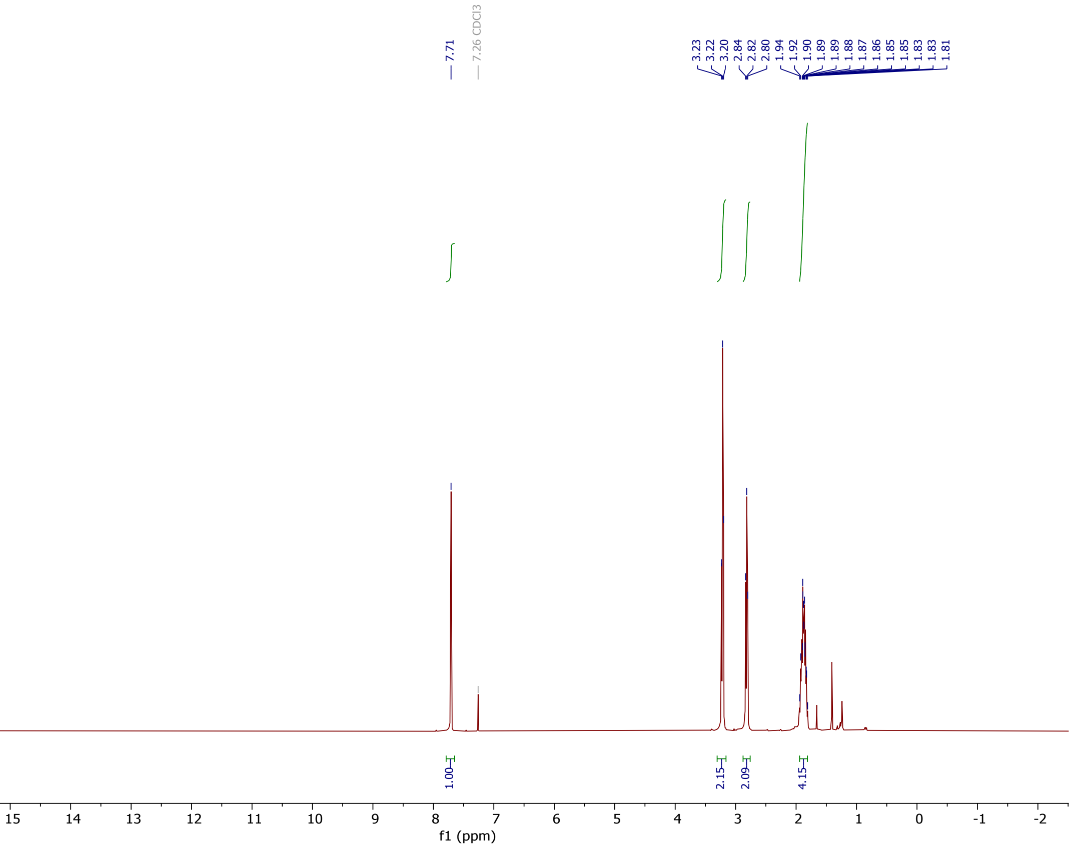

^{13}C NMR spectrum of **12h** ($CDCl_3$, 101 MHz)

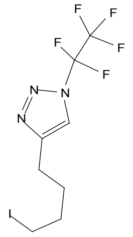

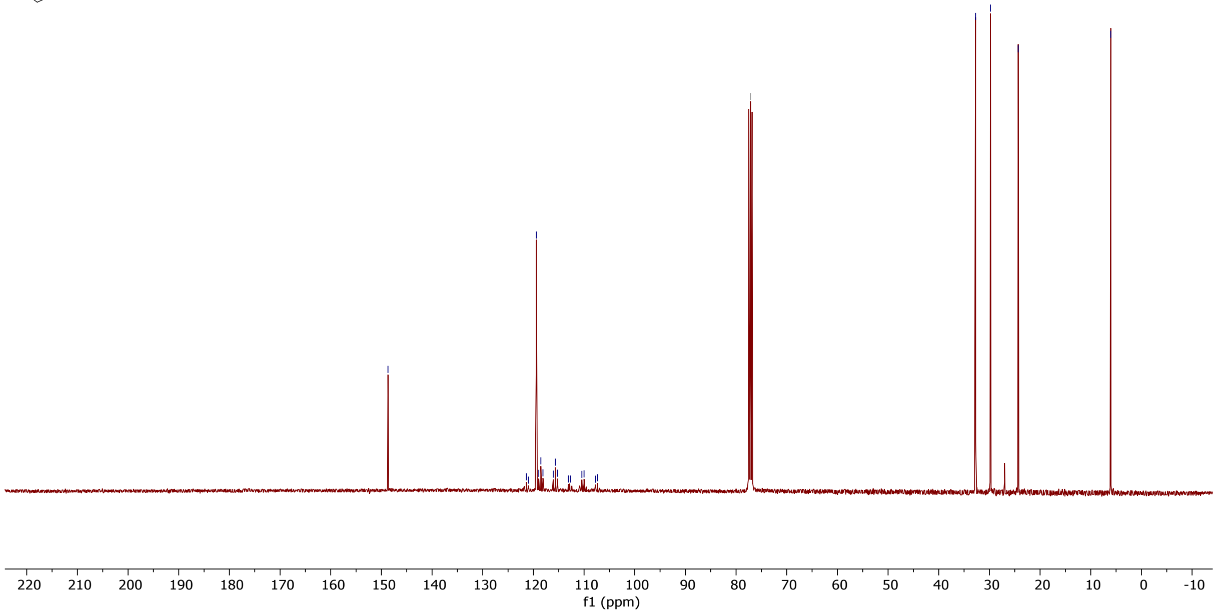

^{19}F NMR spectrum of **12h** ($CDCl_3$, 376 MHz)

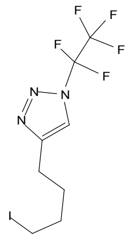

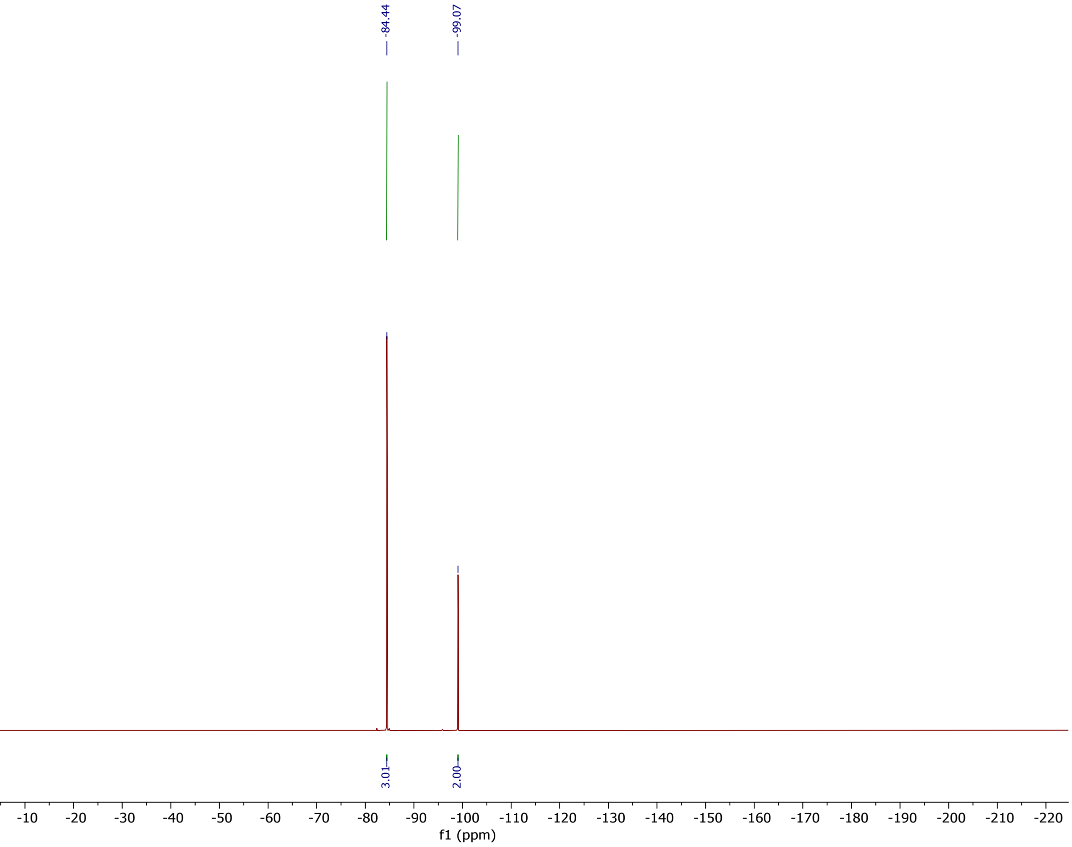

^{1}H NMR spectrum of **18a** ($CDCl_3$, 401 MHz)

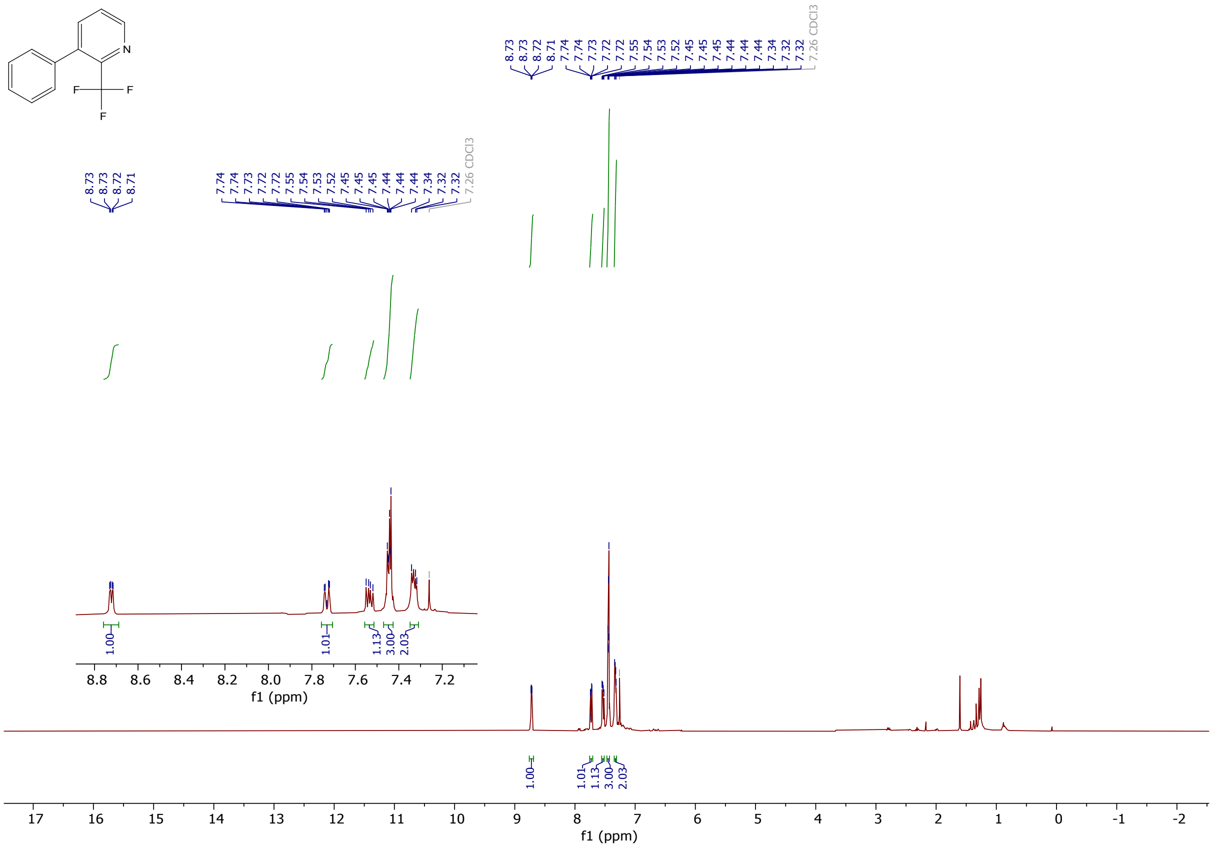

^{13}C NMR spectrum of **18a** ($CDCl_3$, 101 MHz)

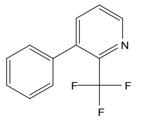

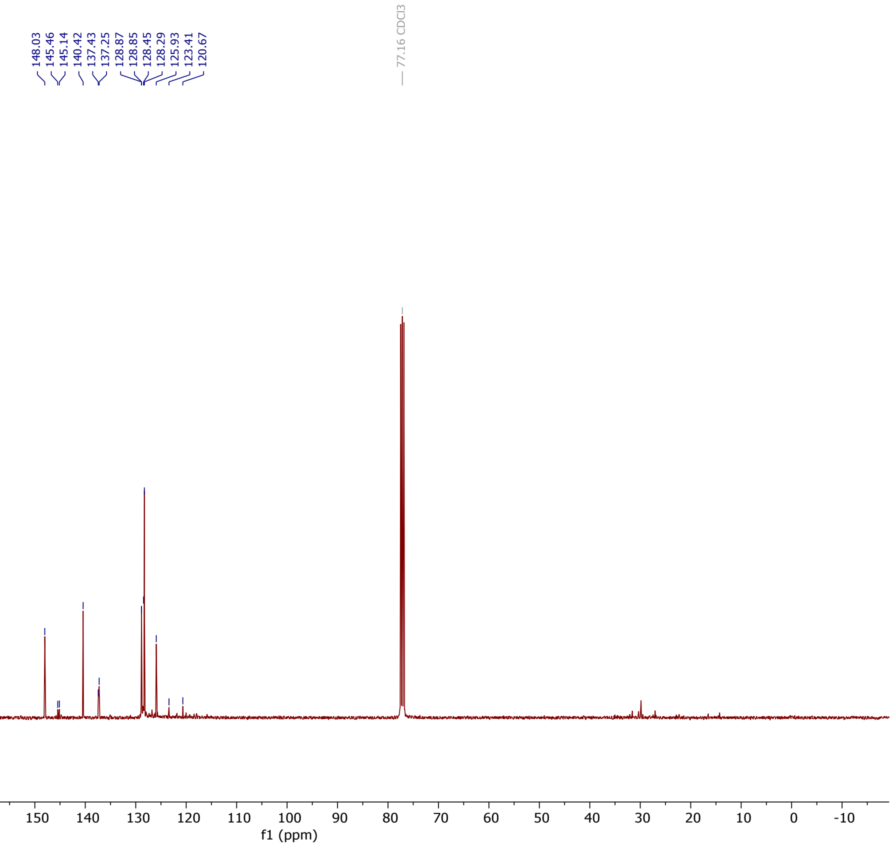

^{19}F NMR spectrum of **18a** ($CDCl_3$, 376 MHz)

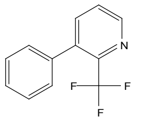

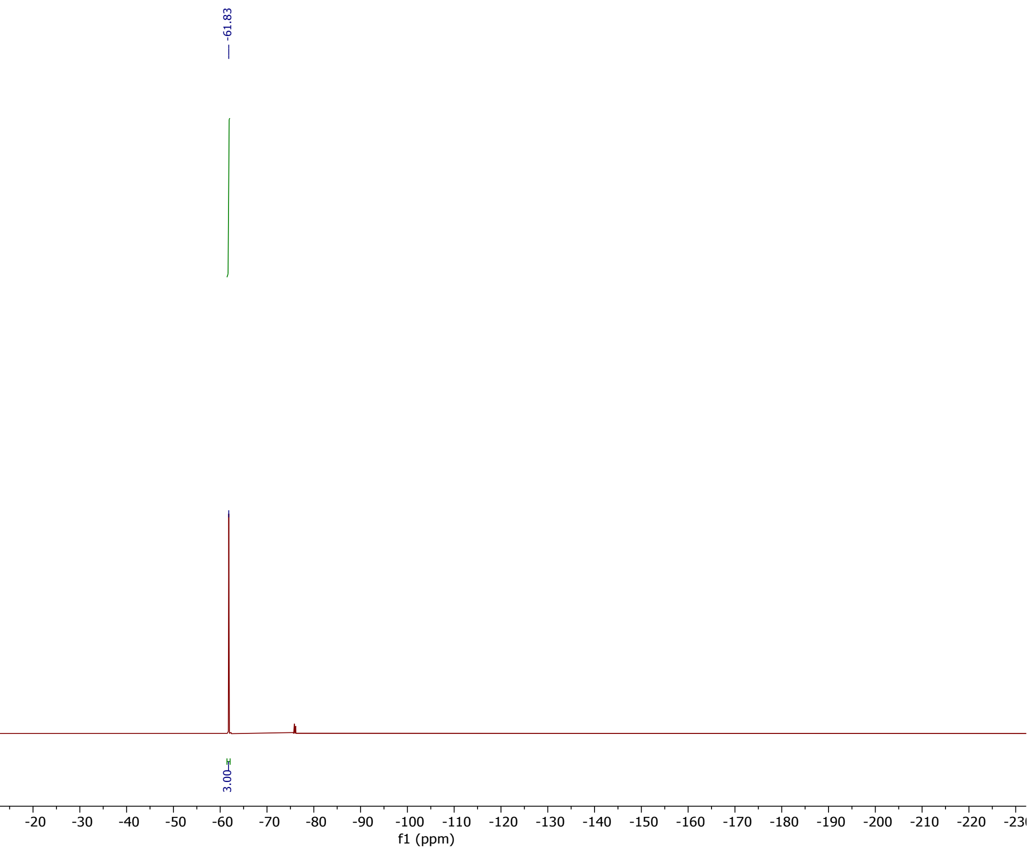

^{1}H NMR spectrum of **18b** ($CDCl_3$, 401 MHz)

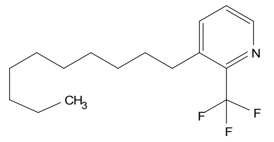

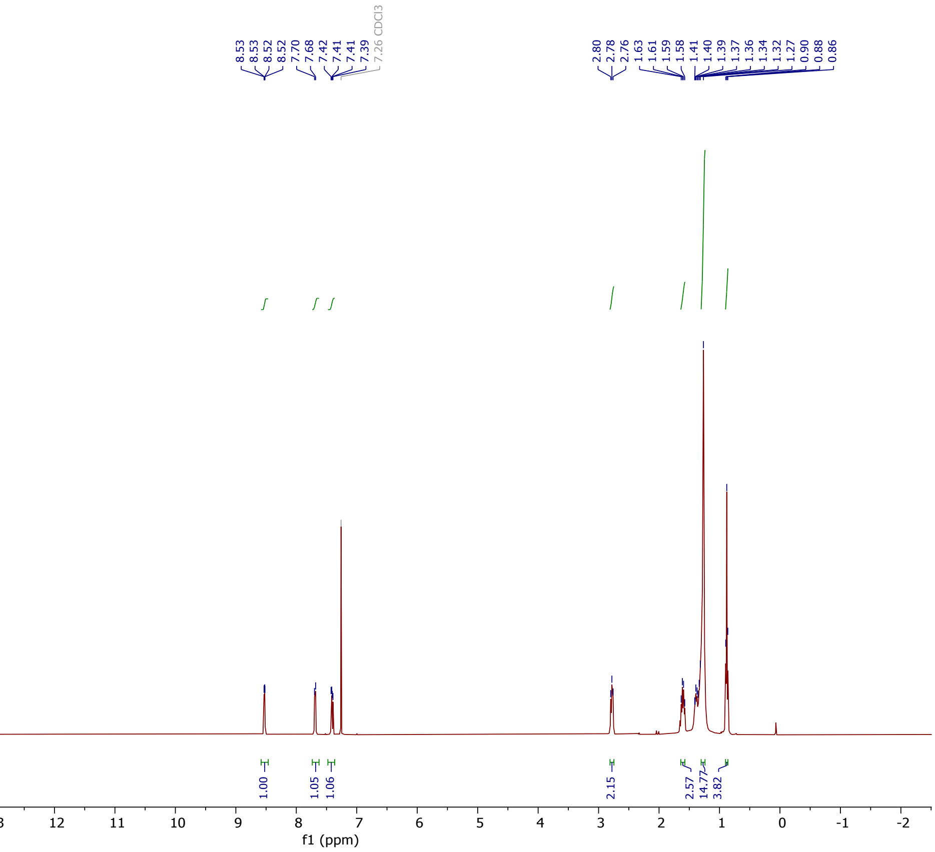

^{13}C NMR spectrum of **18b** ($CDCl_3$, 101 MHz)

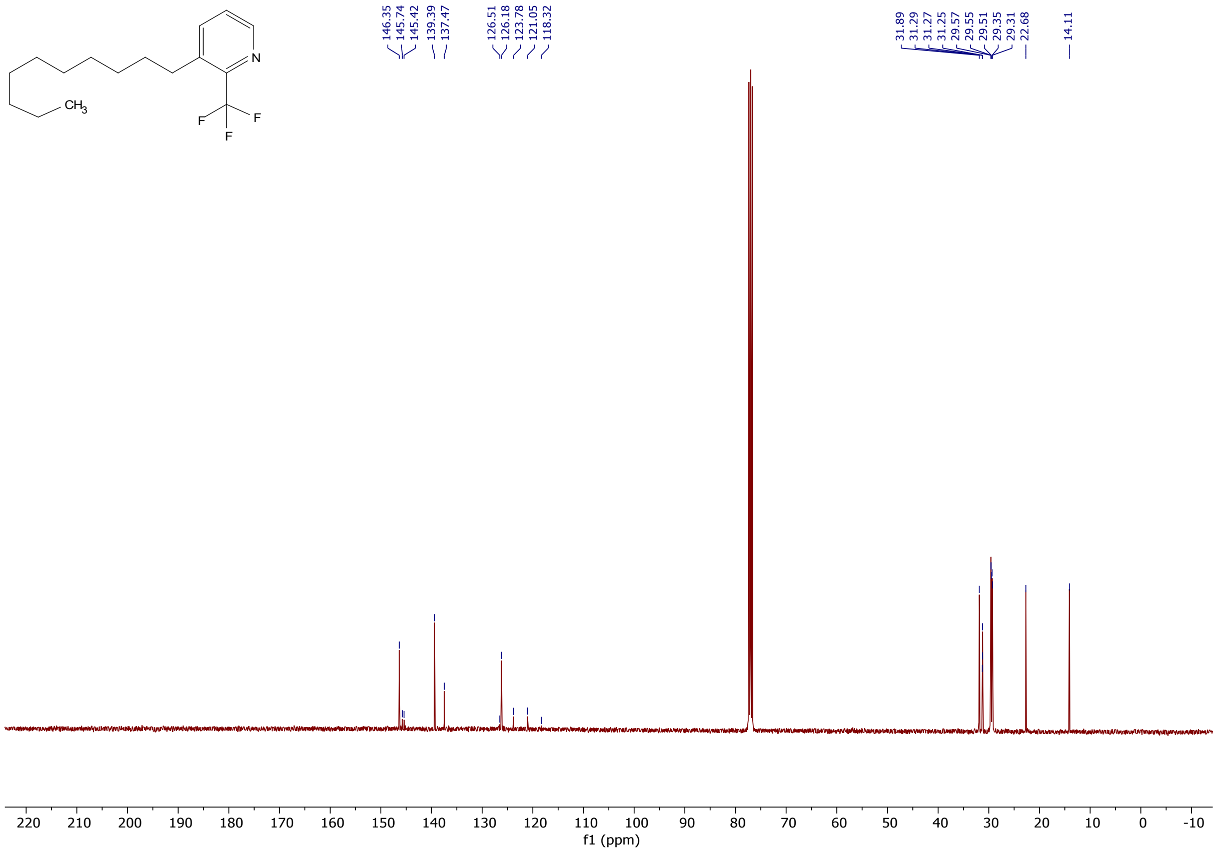

^{19}F NMR spectrum of **18b** ($CDCl_3$, 376 MHz)

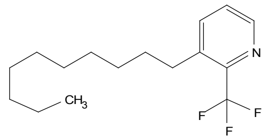

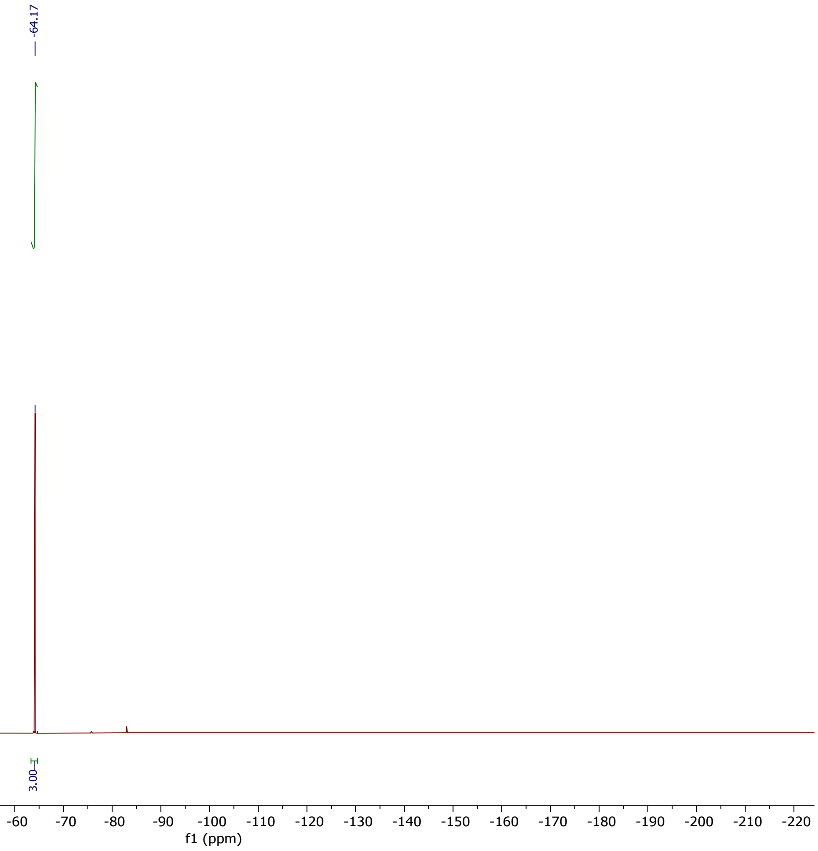

^{1}H NMR spectrum of **18c** ($CDCl_3$, 401 MHz)

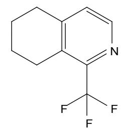

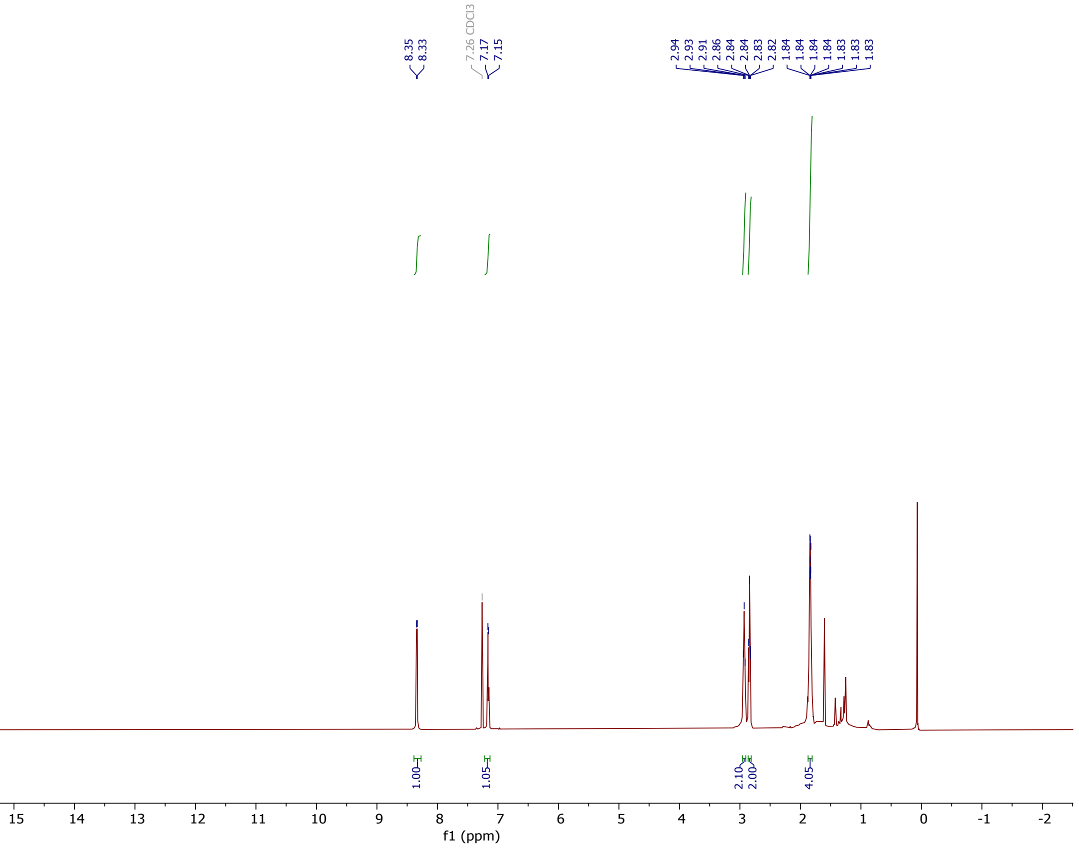

^{13}C NMR spectrum of **18c** ($CDCl_3$, 101 MHz)

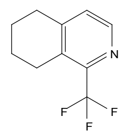

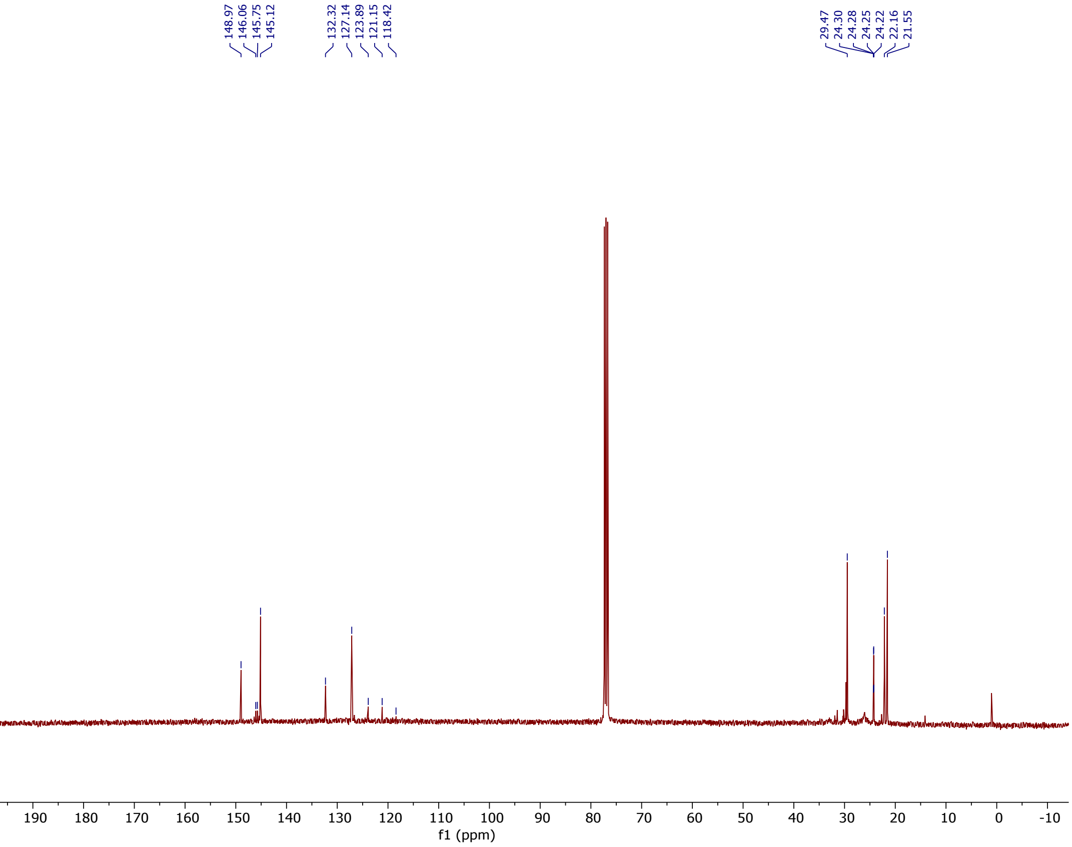

^{19}F NMR spectrum of **18c** ($CDCl_3$, 376 MHz)

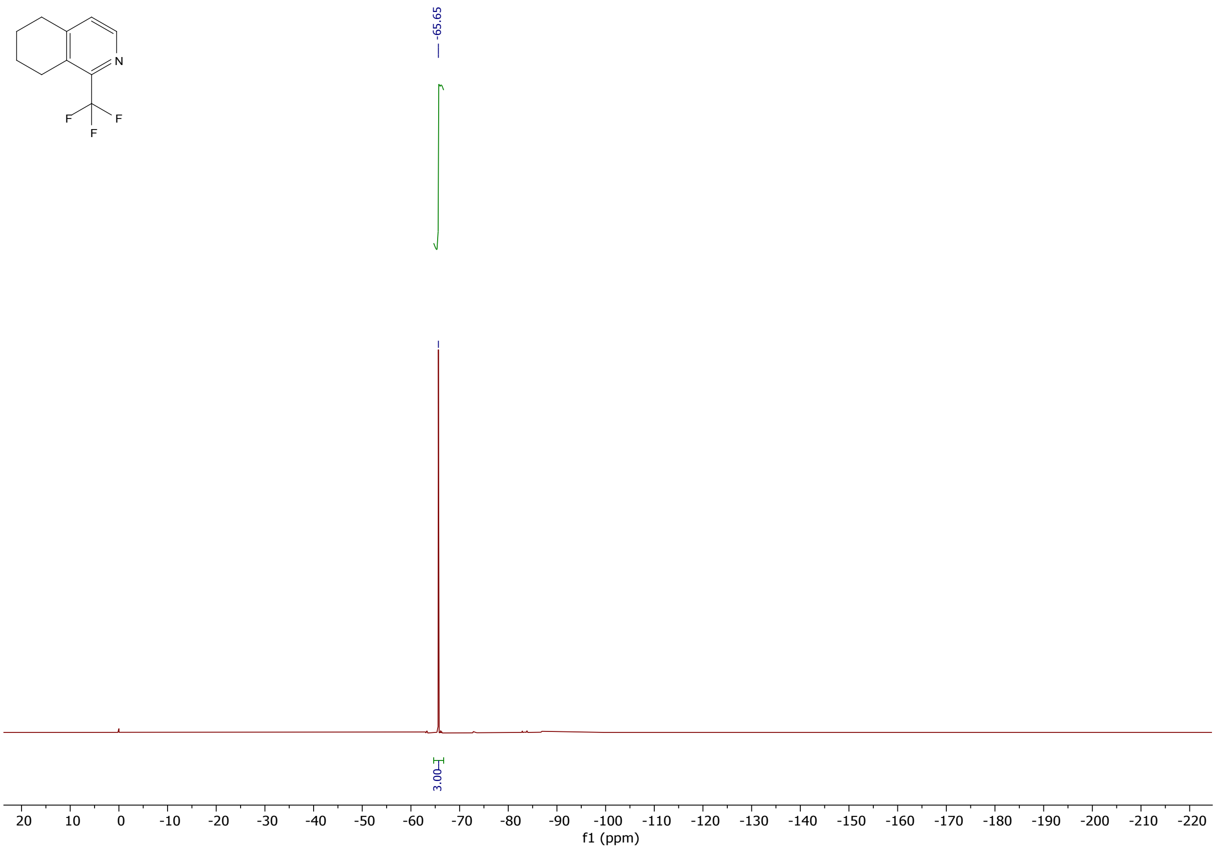

^{1}H NMR spectrum of **18d** ($CDCl_3$, 401 MHz)

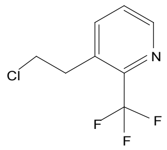

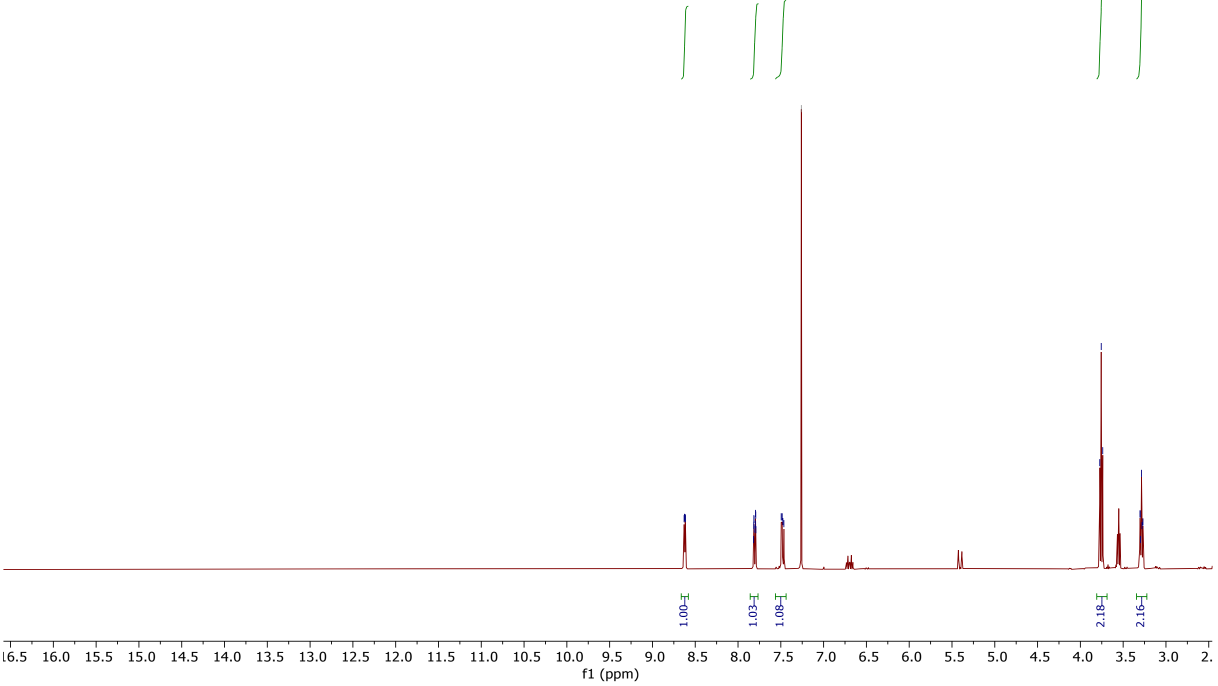

^{13}C NMR spectrum of **18d** ($CDCl_3$, 101 MHz)

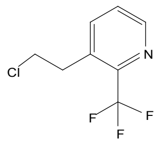

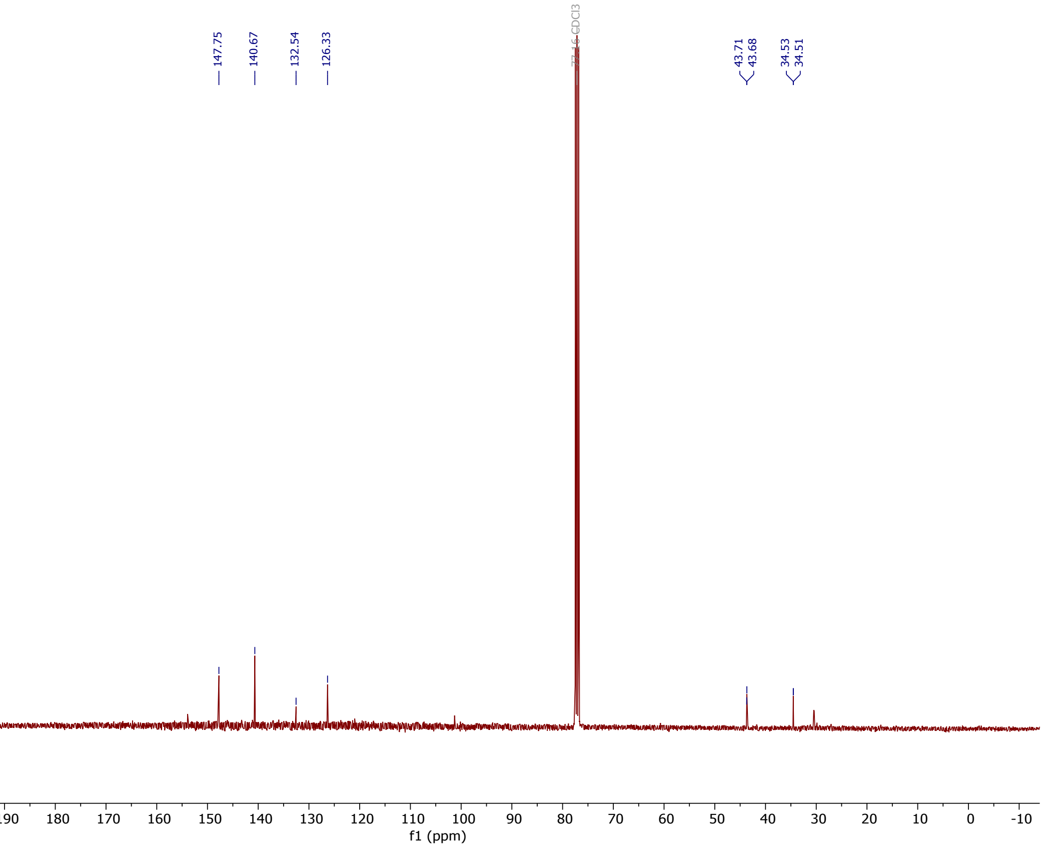

^{19}F NMR spectrum of **18d** ($CDCl_3$, 376 MHz)

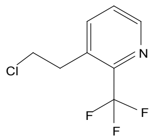

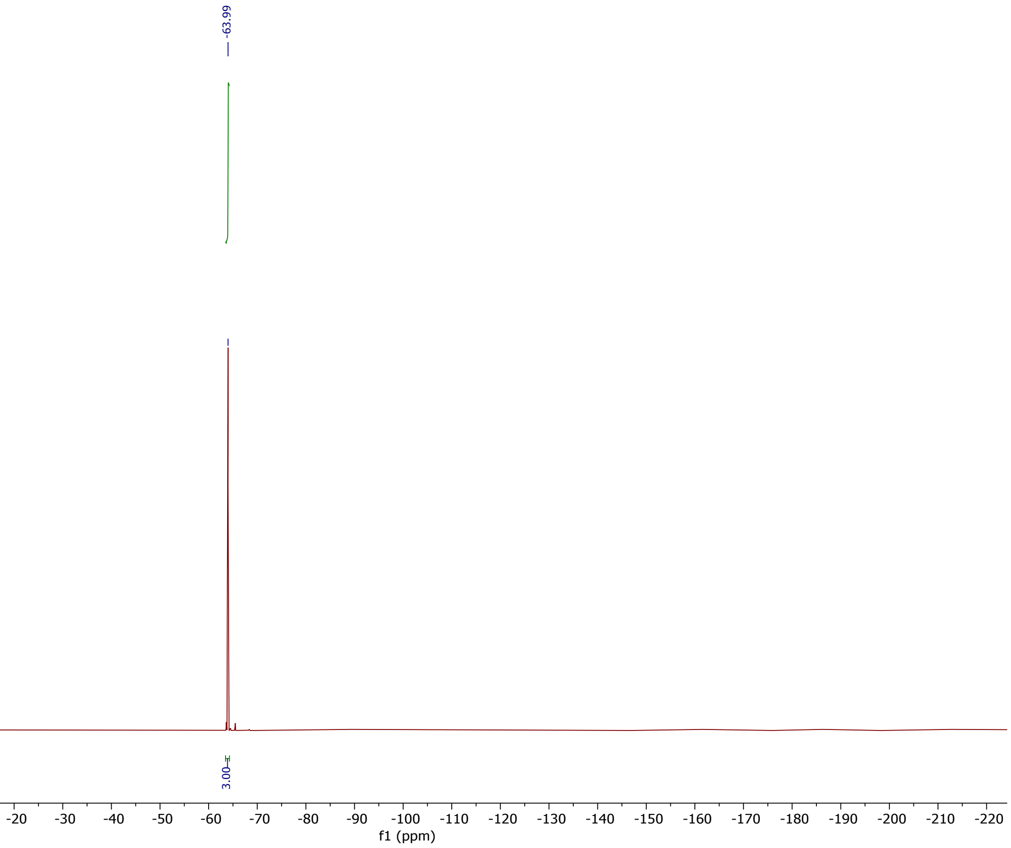

^{1}H NMR spectrum of **18e** ($CDCl_3$, 401 MHz)

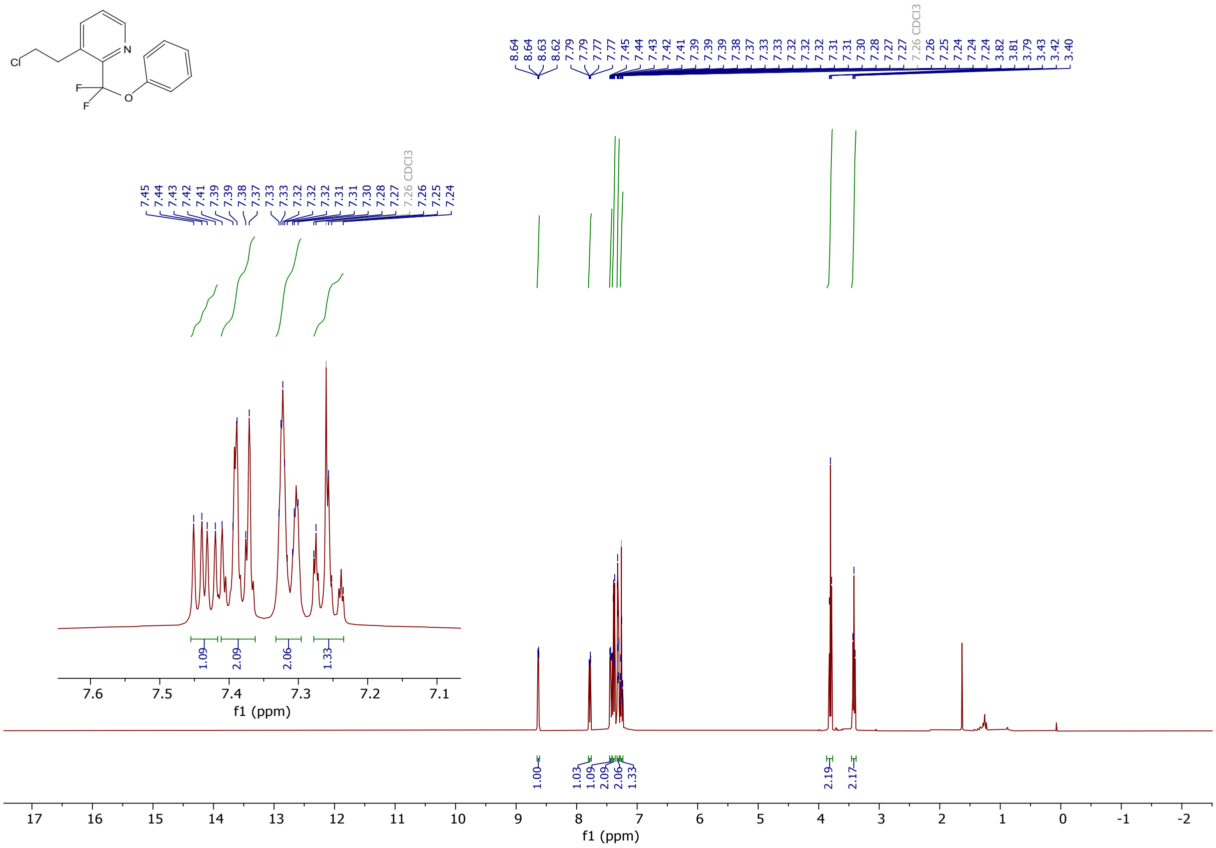

^{13}C NMR spectrum of **18e** ($CDCl_3$, 101 MHz)

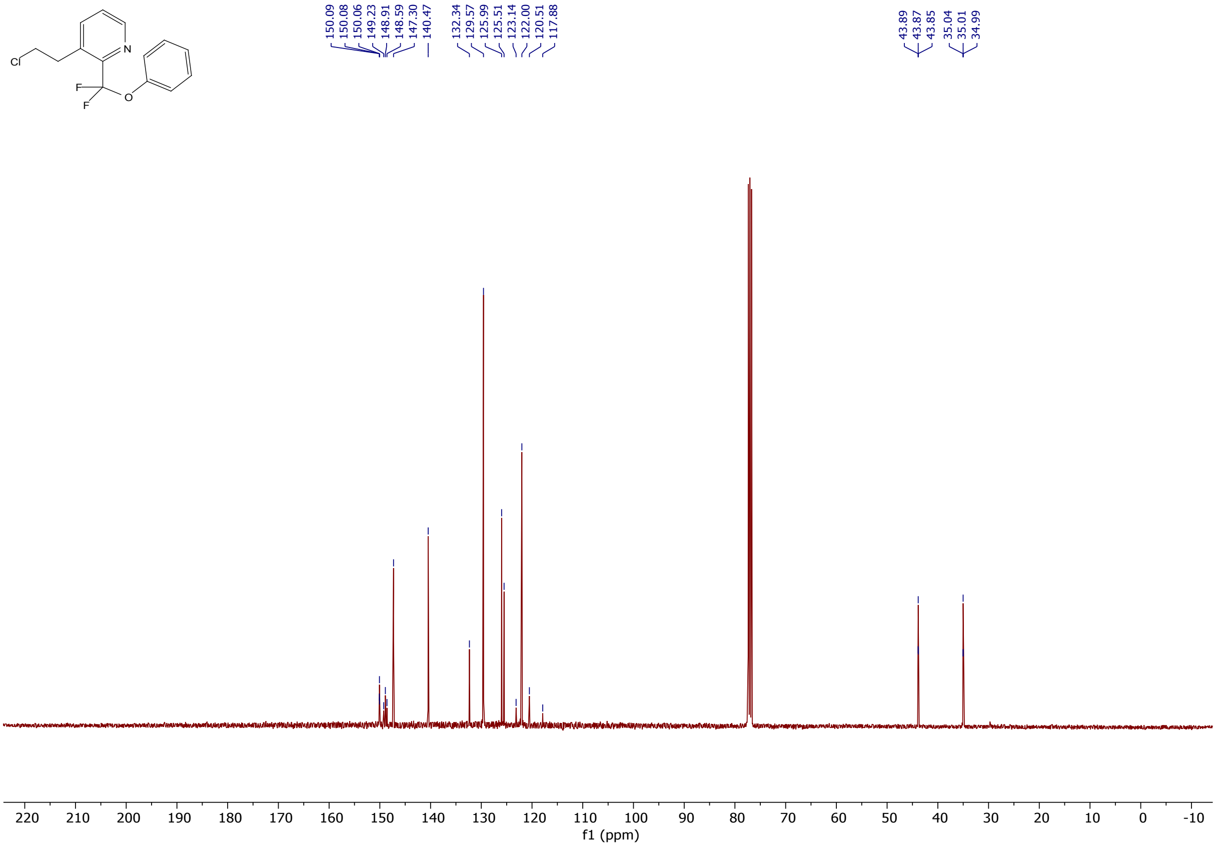

^{19}F NMR spectrum of **18e** ($CDCl_3$, 376 MHz)

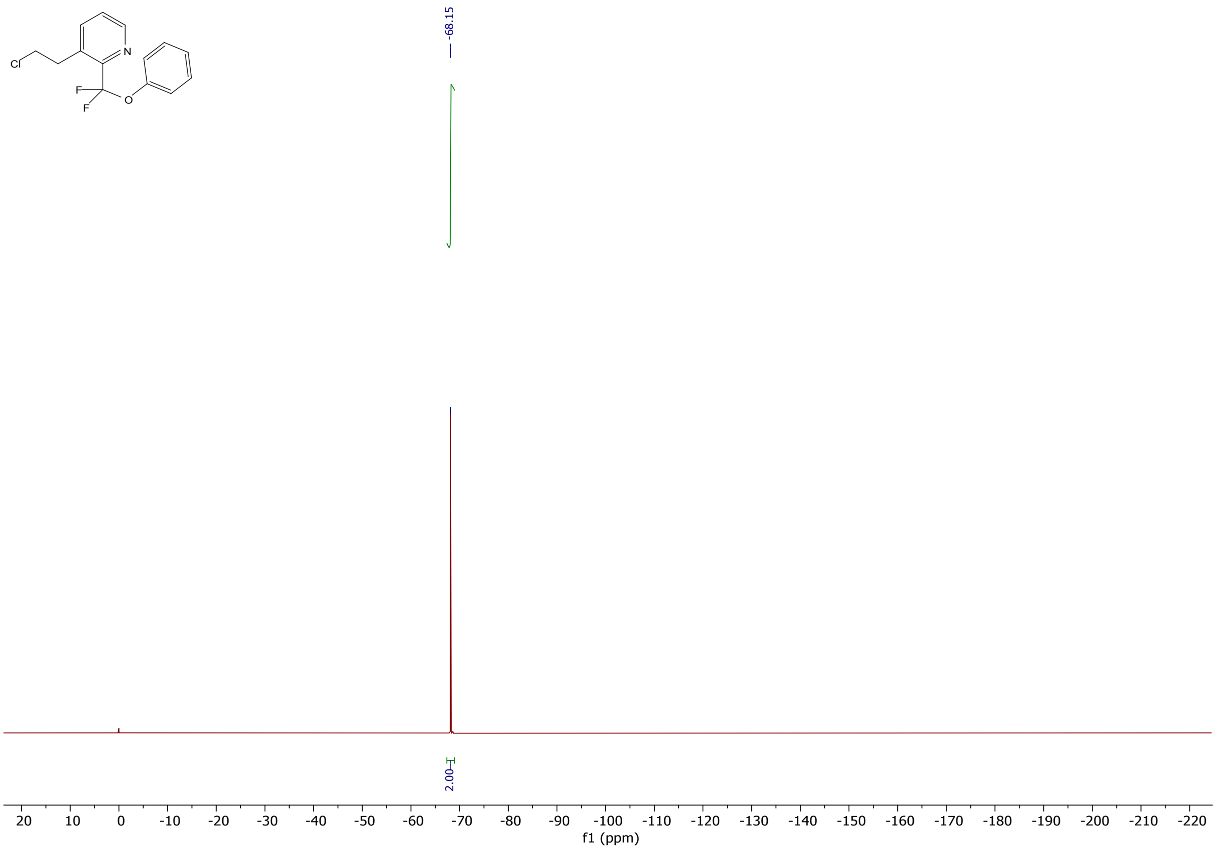

^{1}H NMR spectrum of **18f** ($CDCl_3$, 401 MHz)

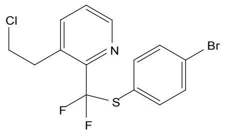

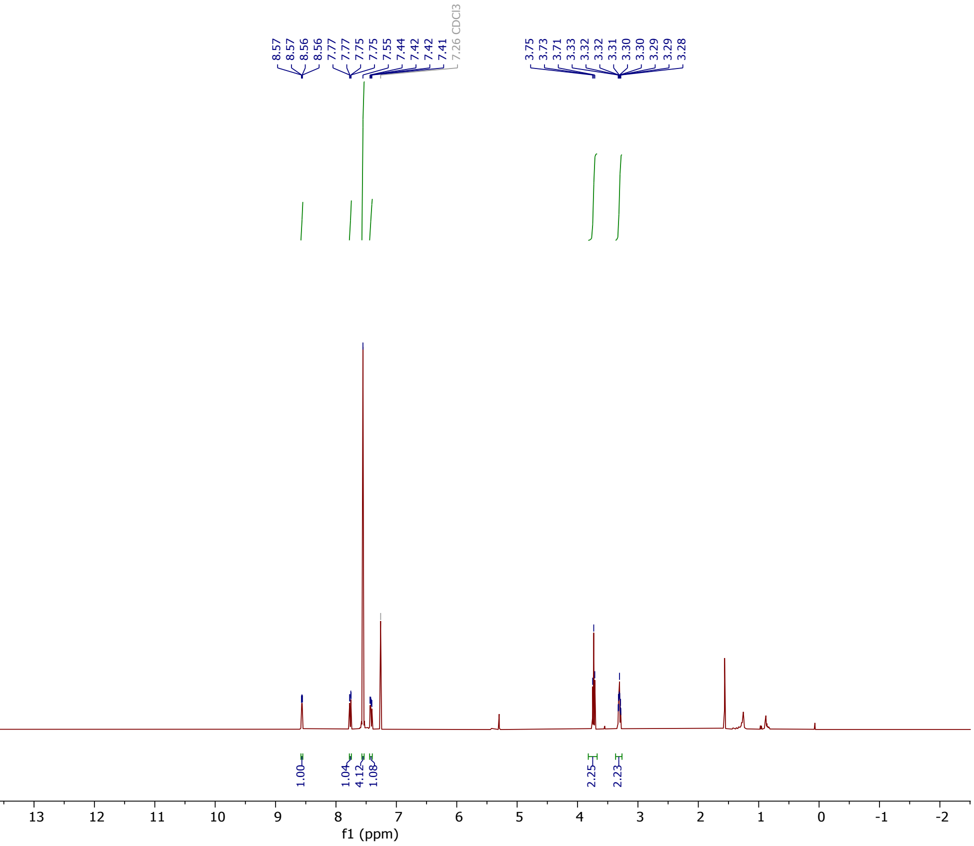

^{13}C NMR spectrum of **18f** ($CDCl_3$, 101 MHz)

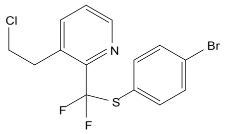

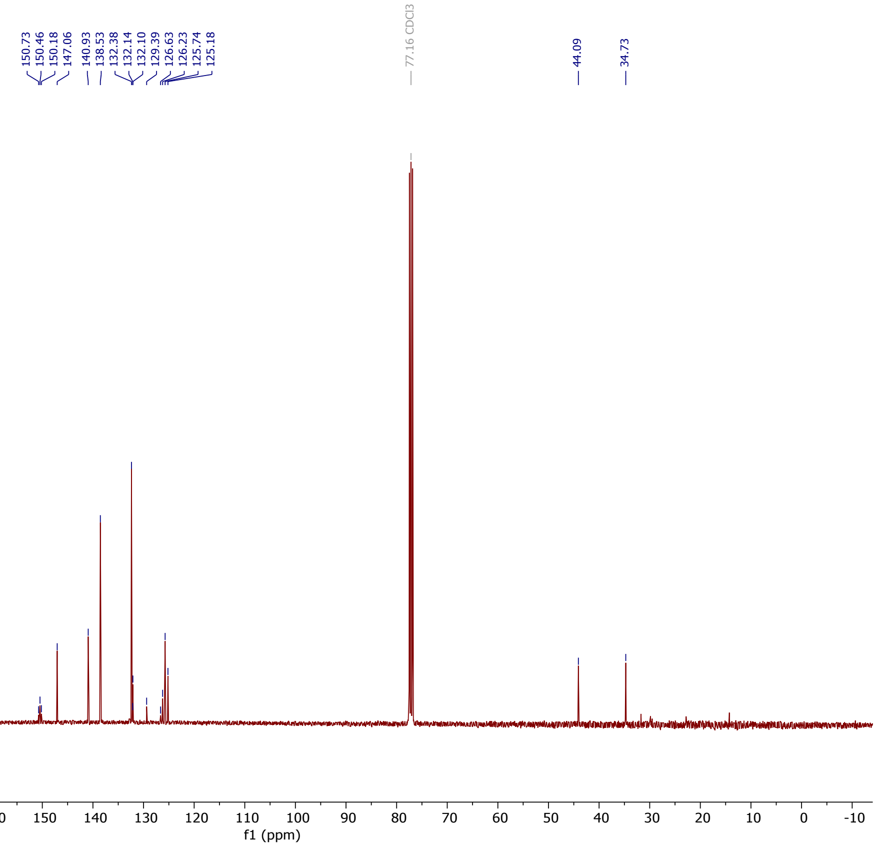

^{19}F NMR spectrum of **18f** ($CDCl_3$, 376 MHz)

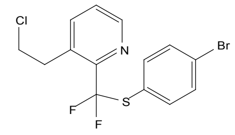

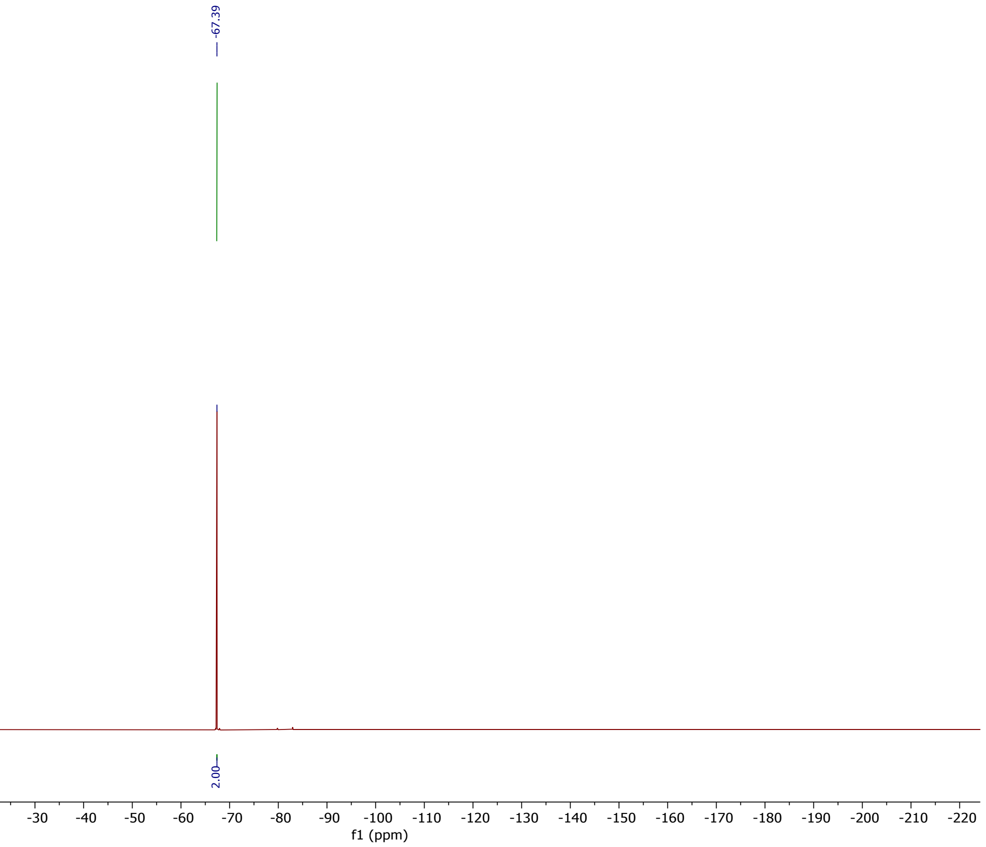

^{1}H NMR spectrum of **18g** ($CDCl_3$, 401 MHz)

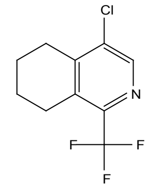

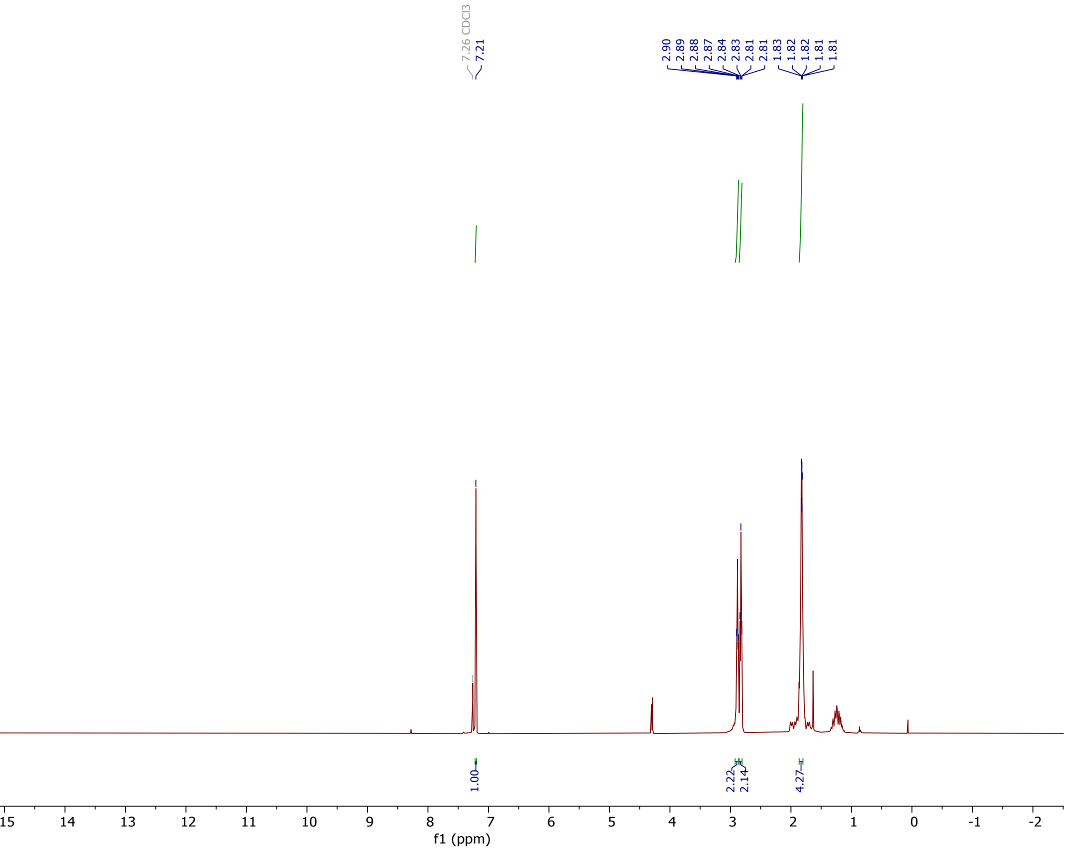

^{13}C NMR spectrum of **18g** ($CDCl_3$, 101 MHz)

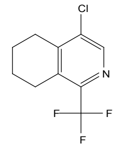

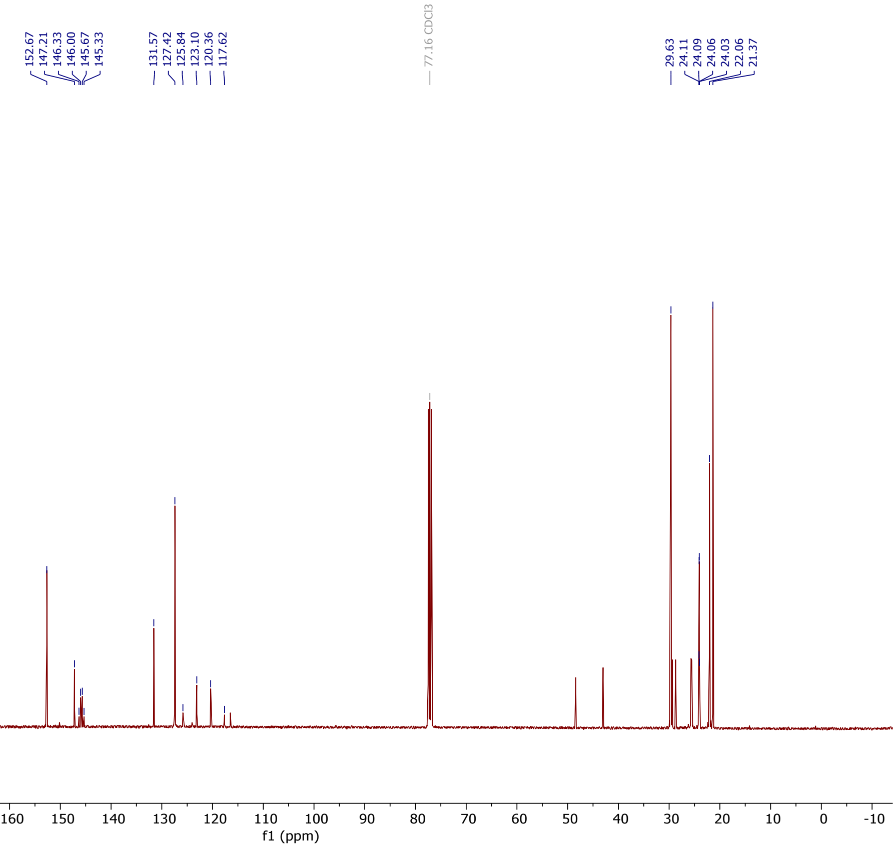

^{19}F NMR spectrum of **18g** ($CDCl_3$, 376 MHz)

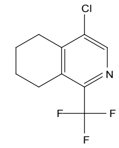

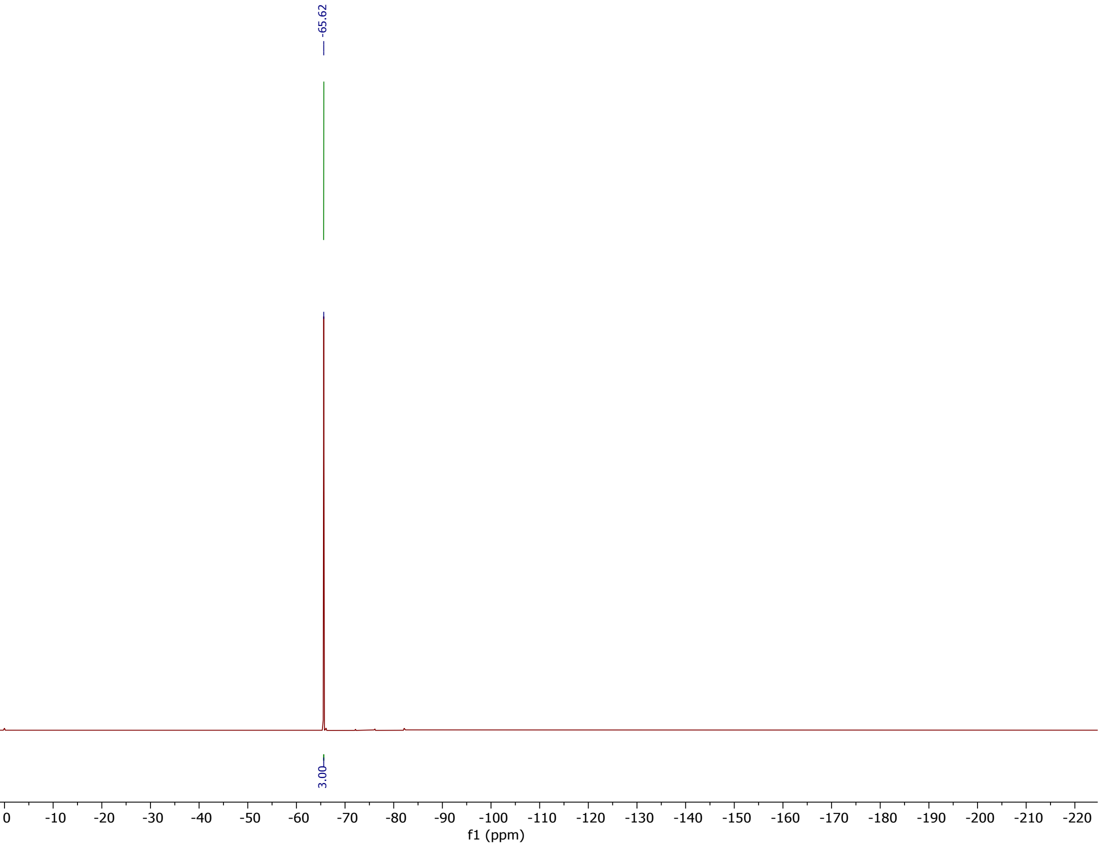

^1H NMR spectrum of **19a** ($CDCl_3$, 401 MHz)

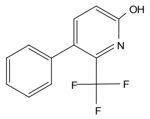

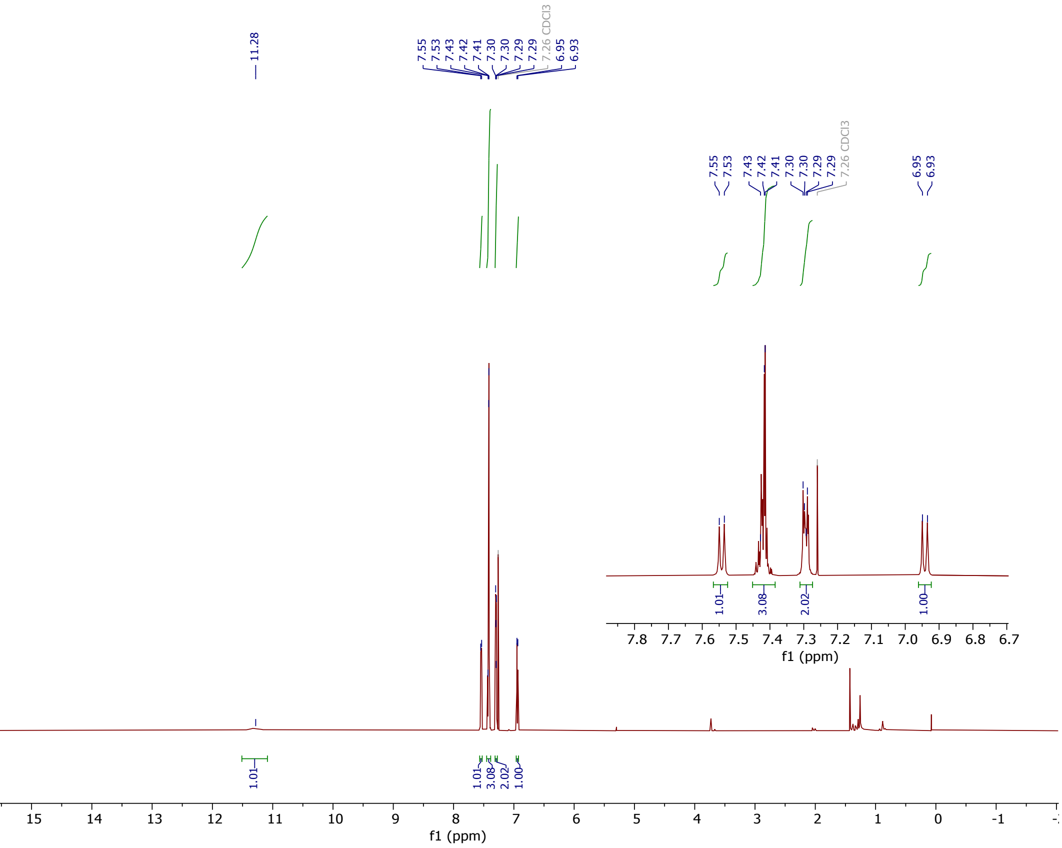

^{13}C NMR spectrum of **19a** ($CDCl_3$, 101 MHz)

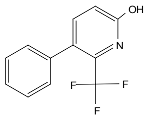

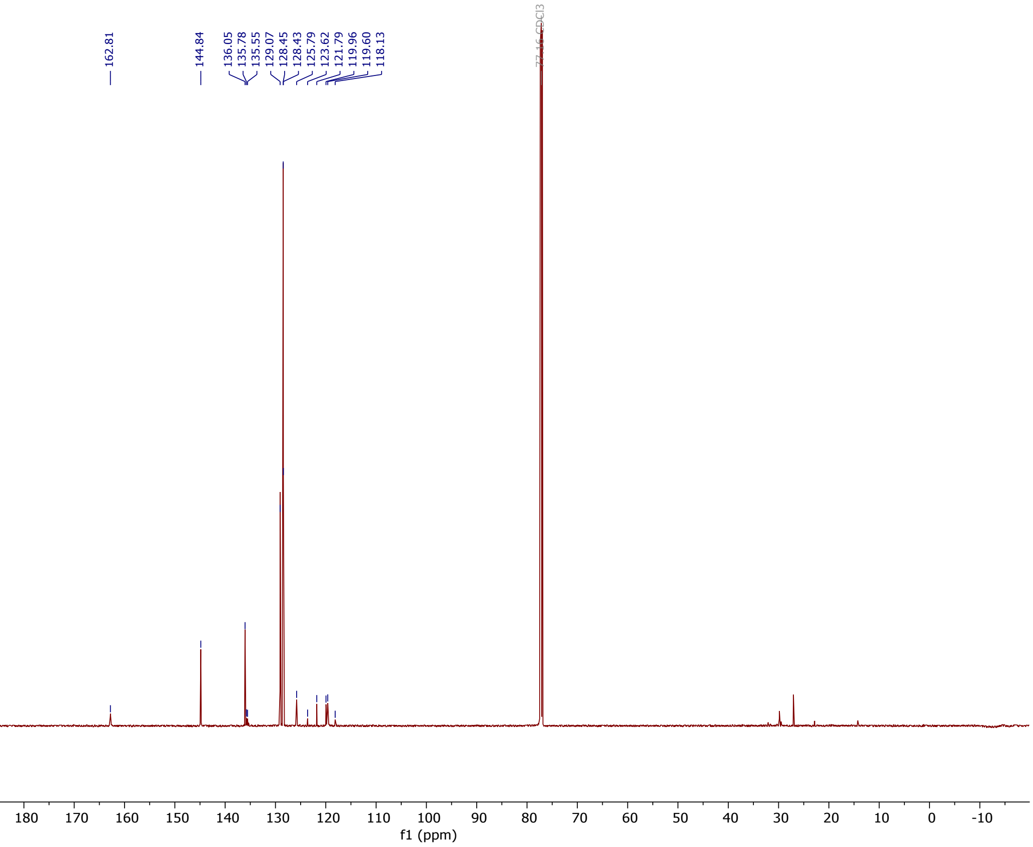

^{19}F NMR spectrum of **19a** ($CDCl_3$, 376 MHz)

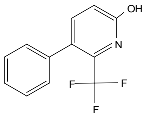

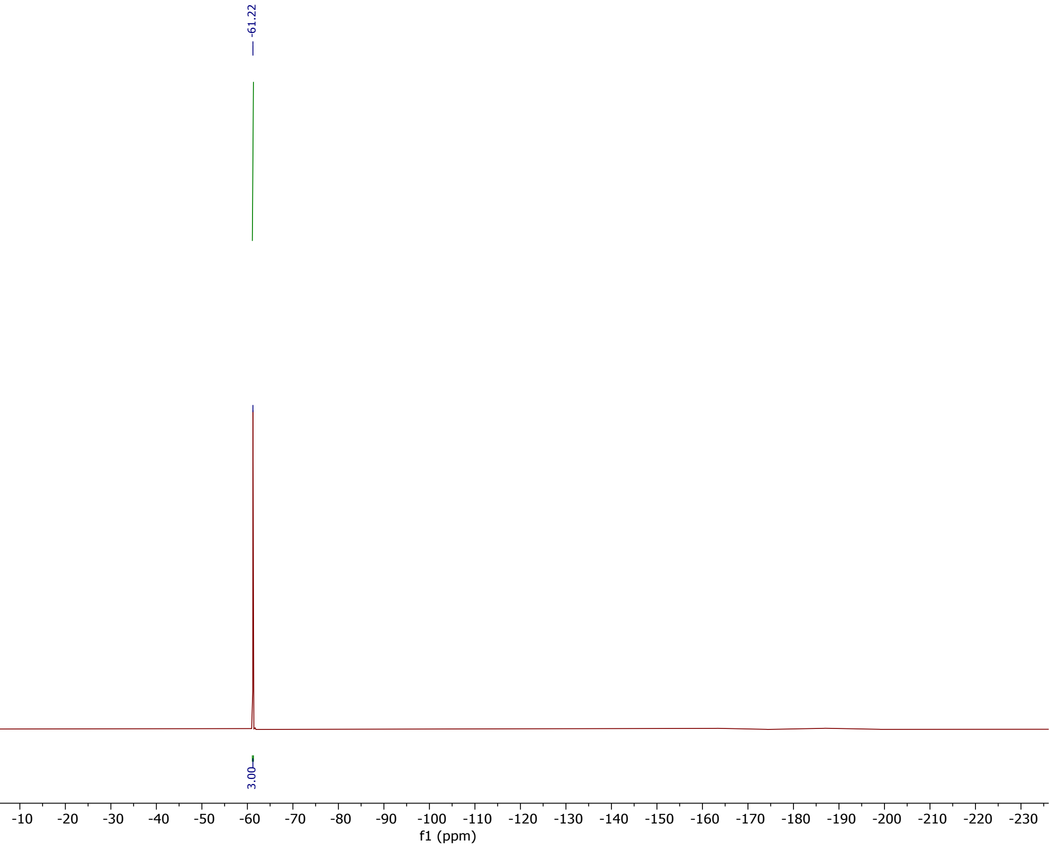

^{1}H NMR spectrum of **19b** ($CDCl_3$, 401 MHz)

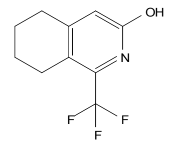

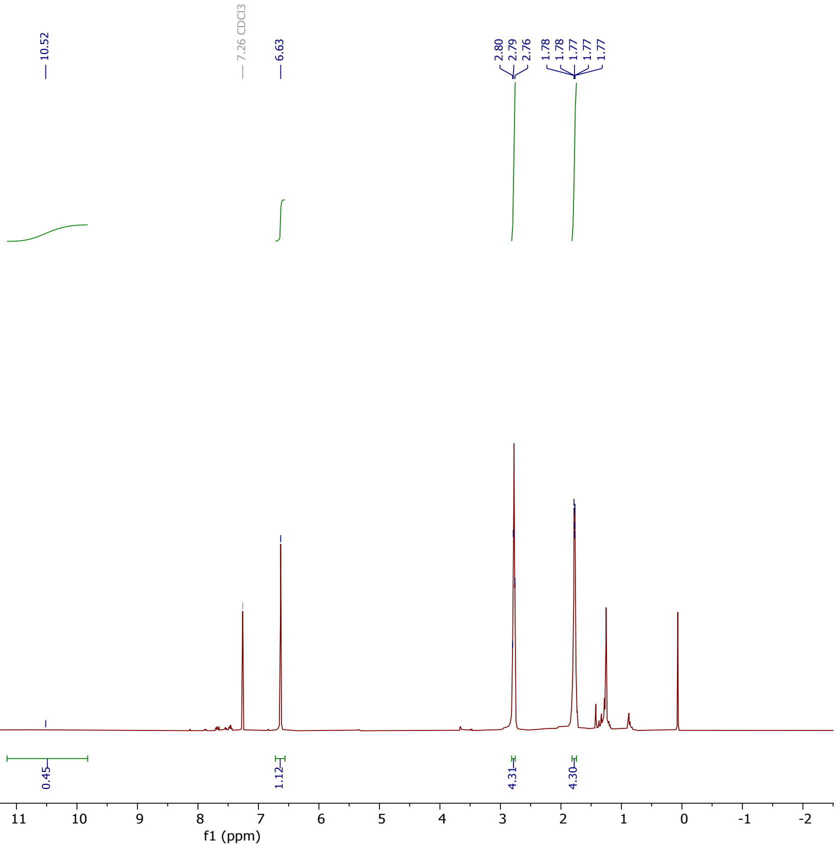

^{13}C NMR spectrum of **19b** ($CDCl_3$, 101 MHz)

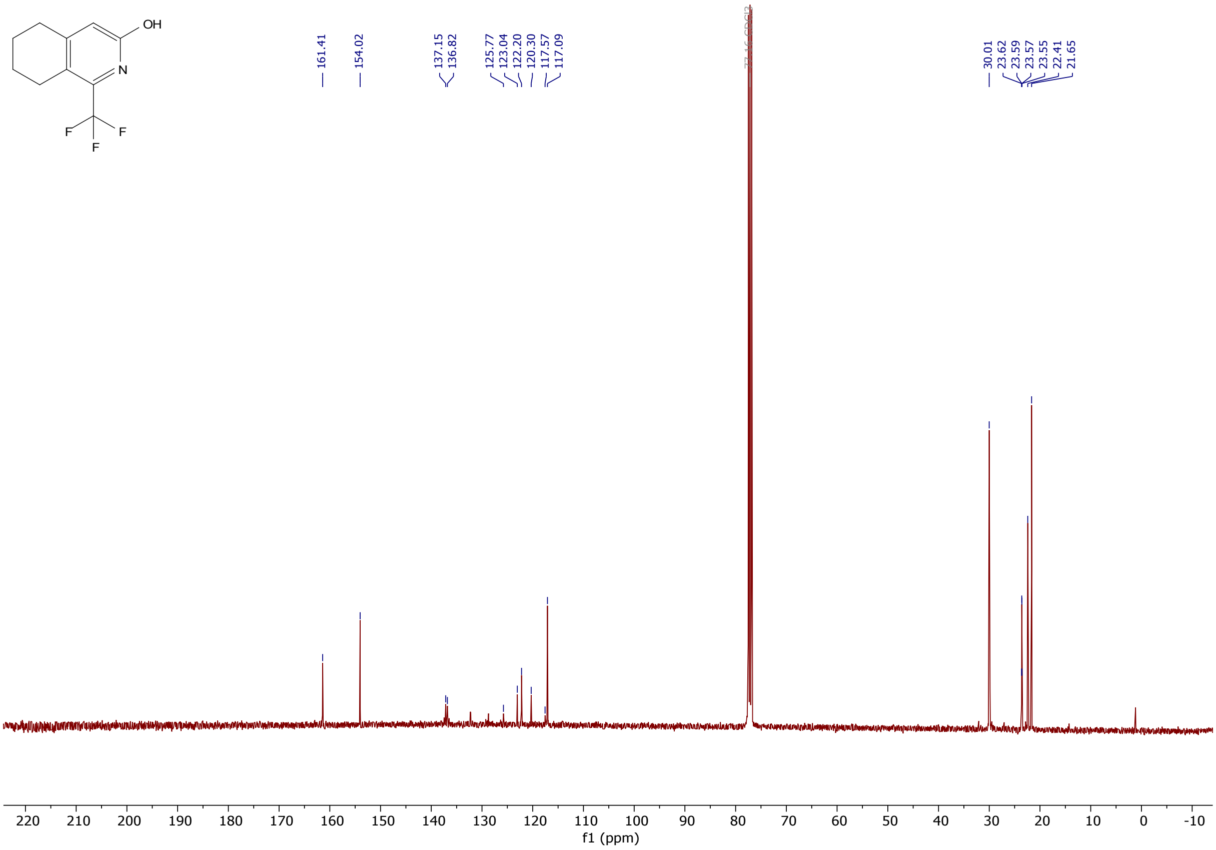

^{19}F NMR spectrum of **19b** ($CDCl_3$, 376 MHz)

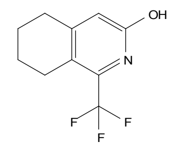

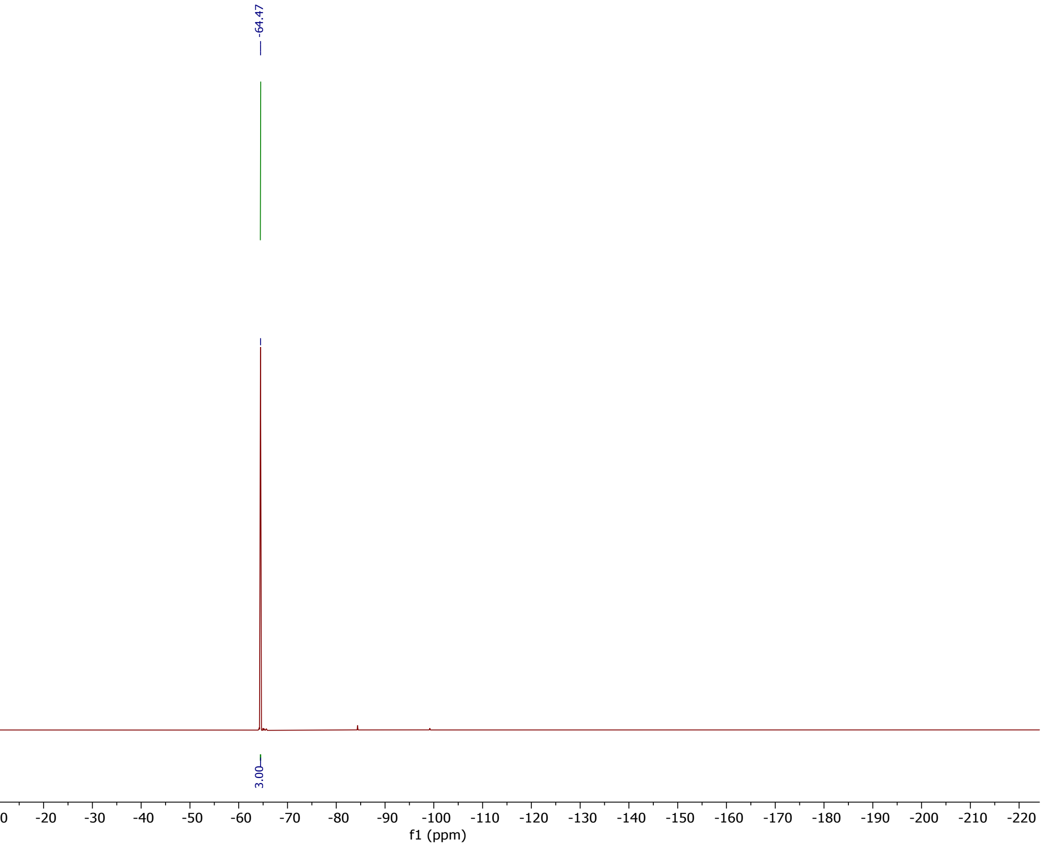

^1H NMR spectrum of **19c** ($CDCl_3$, 401 MHz)

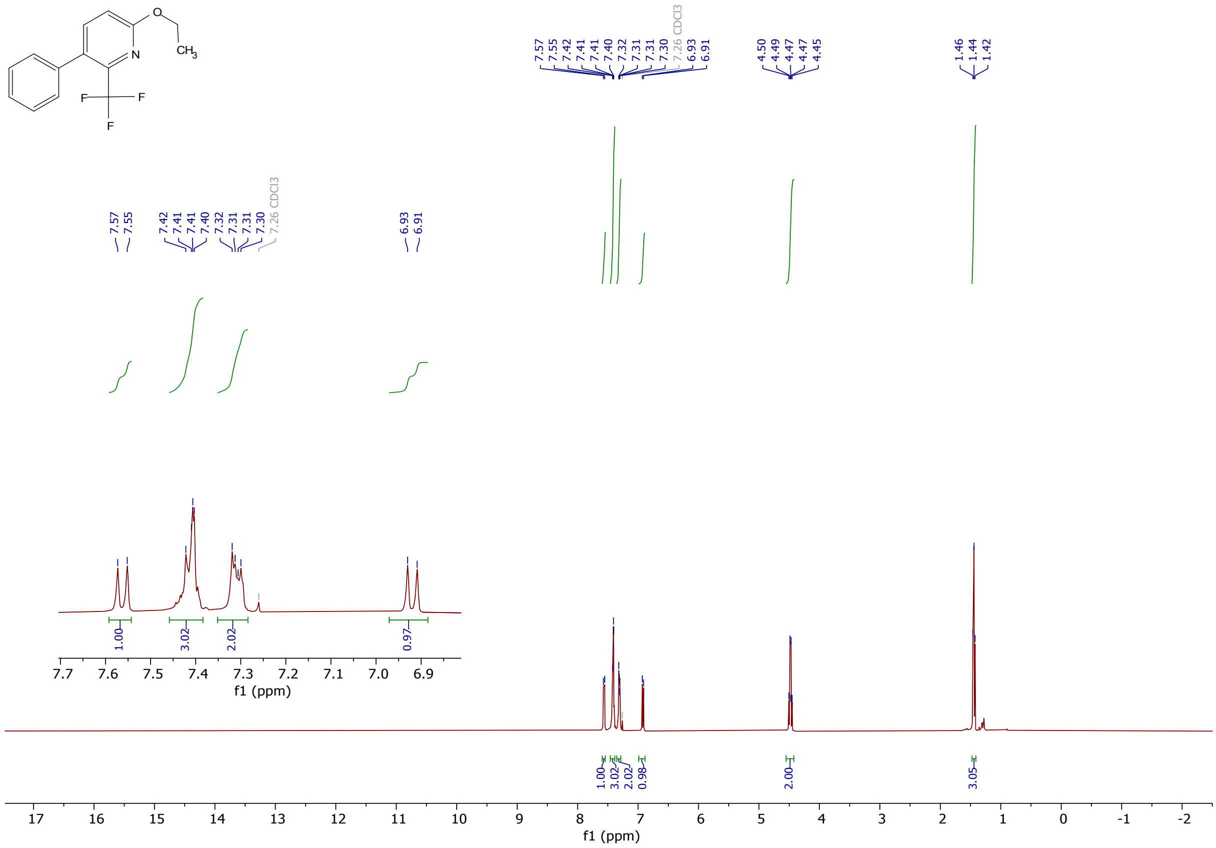

^{13}C NMR spectrum of **19c** ($CDCl_3$, 101 MHz)

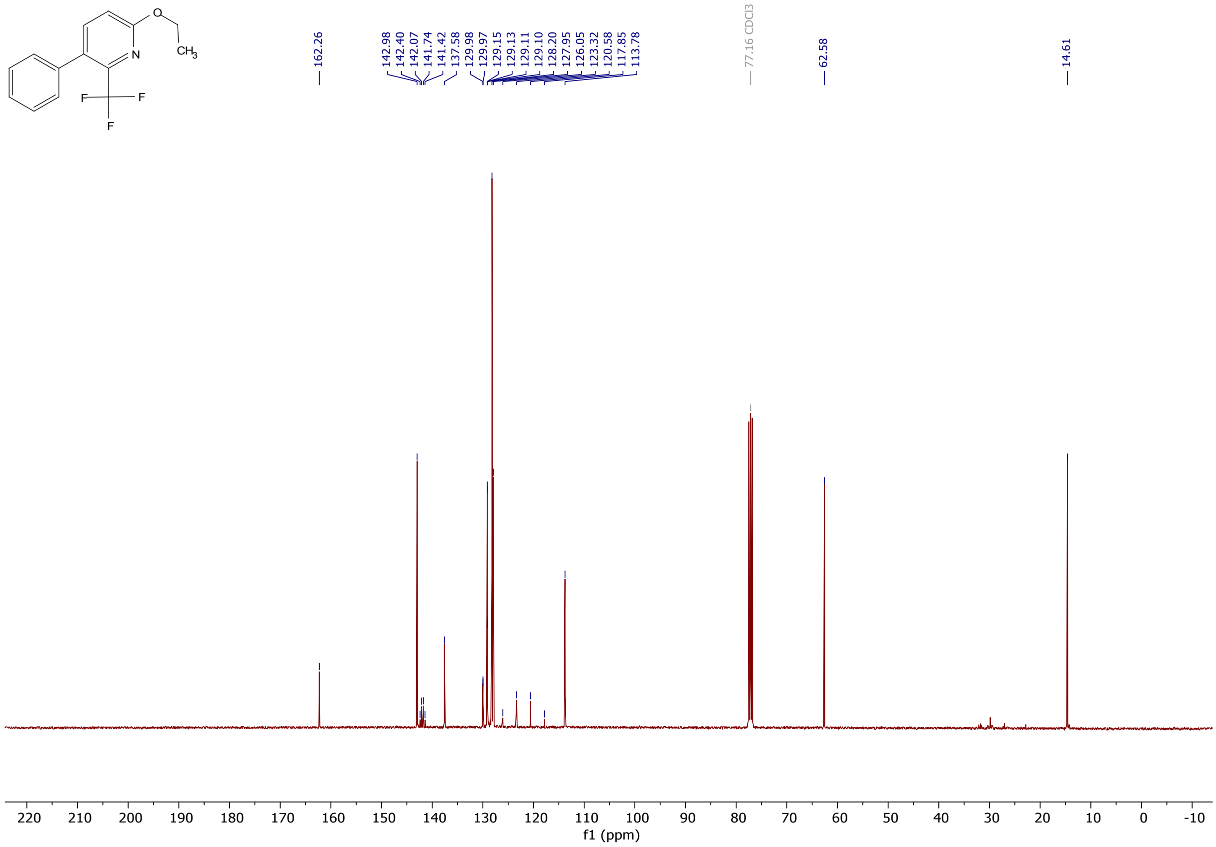

^{19}F NMR spectrum of **19c** ($CDCl_3$, 376 MHz)

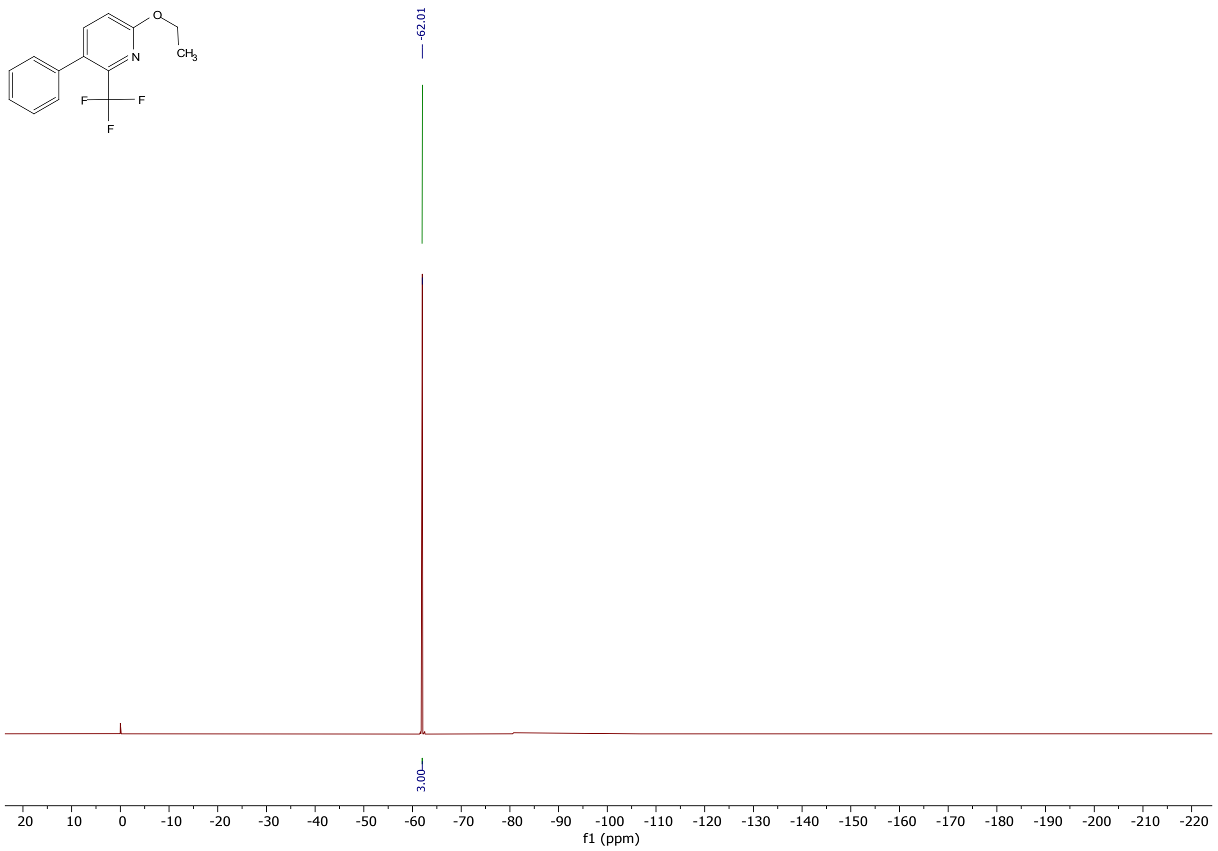

^{1}H NMR spectrum of **19d** ($CDCl_3$, 401 MHz)

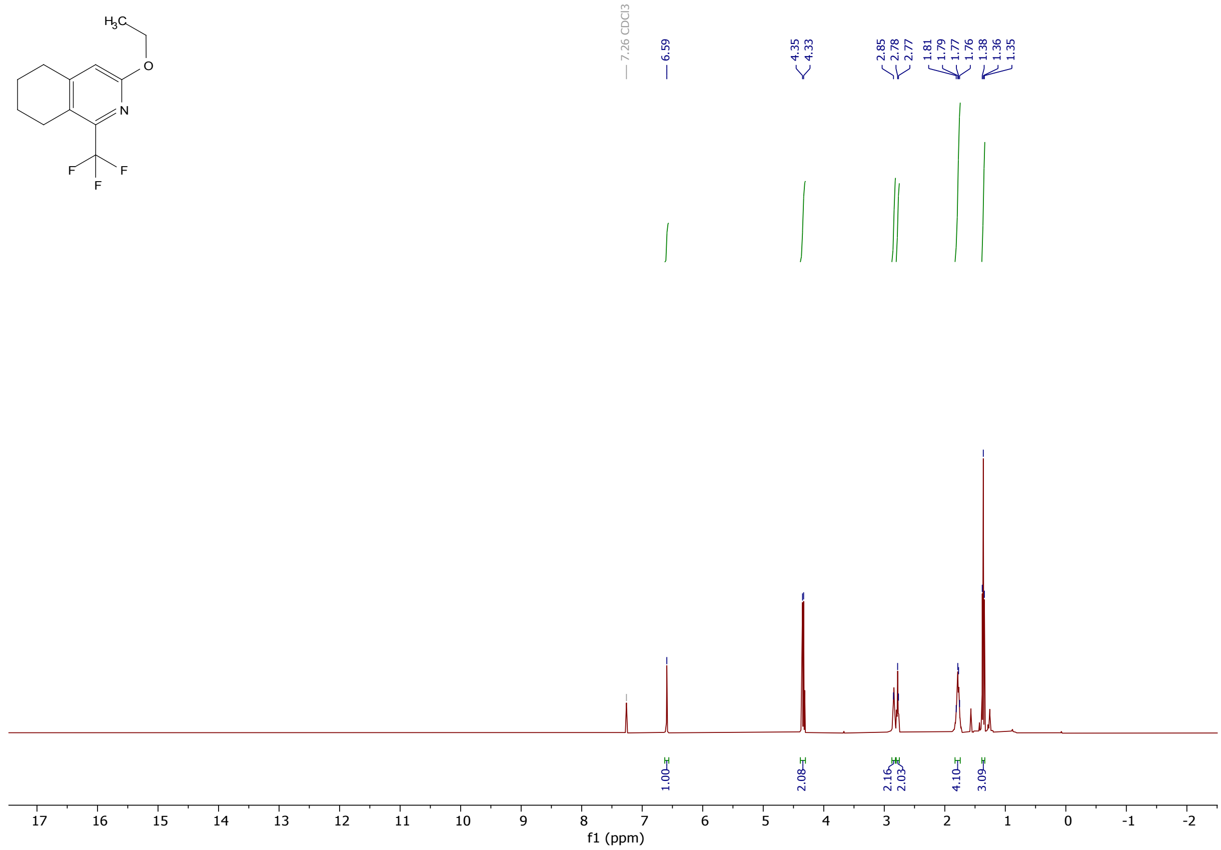

^{13}C NMR spectrum of **19d** ($CDCl_3$, 101 MHz)

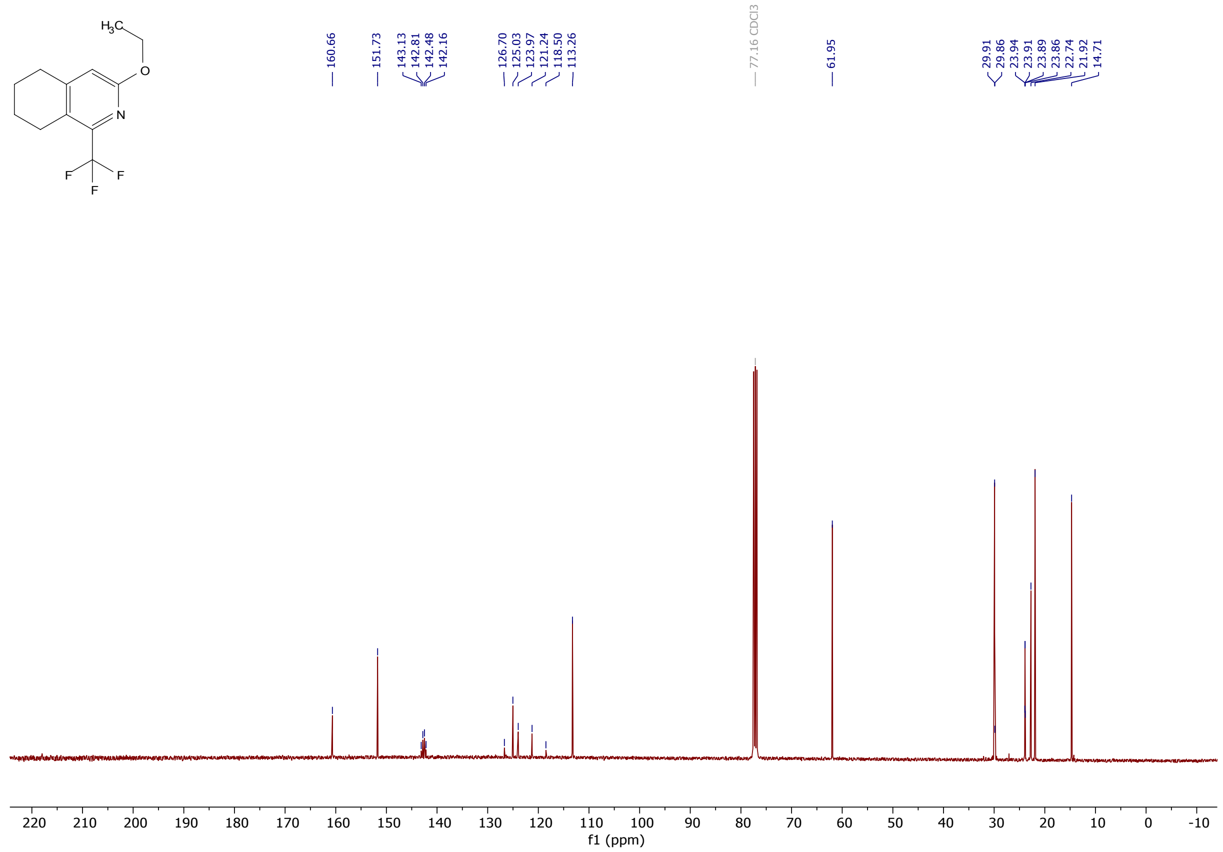

^{19}F NMR spectrum of **19d** ($CDCl_3$, 376 MHz)

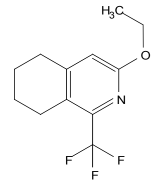

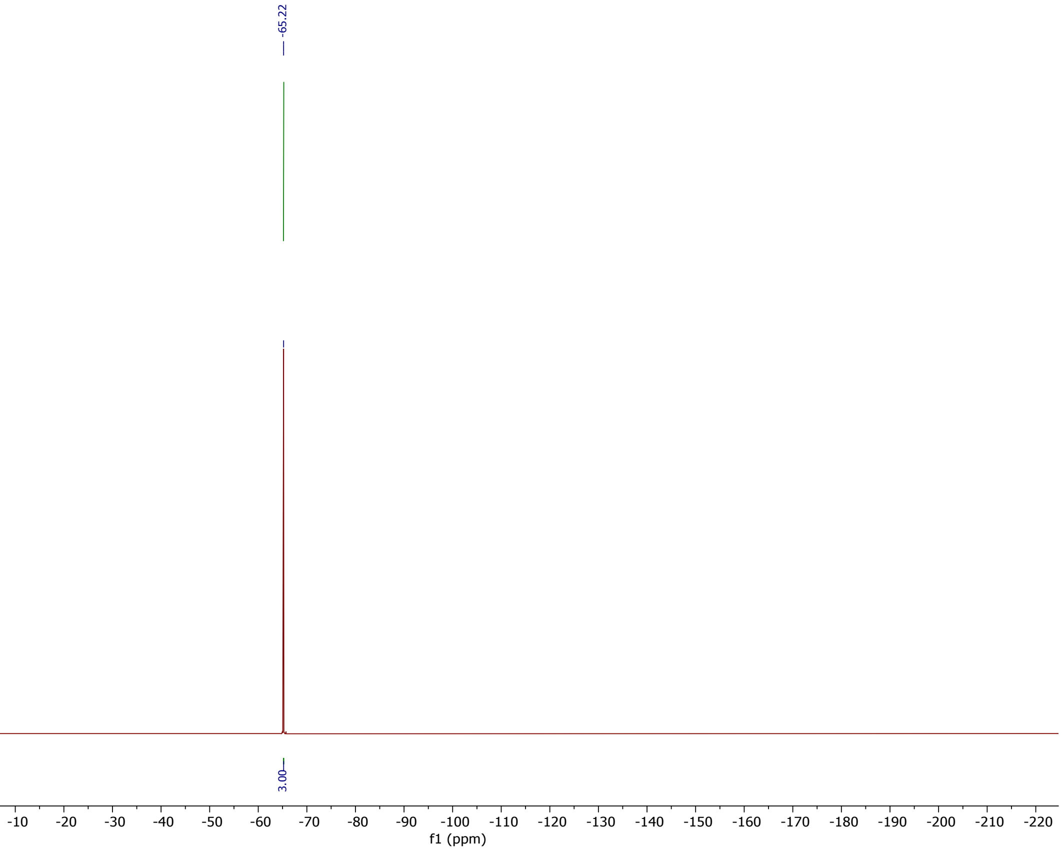

^{1}H NMR spectrum of **19e** ($CDCl_3$, 401 MHz)

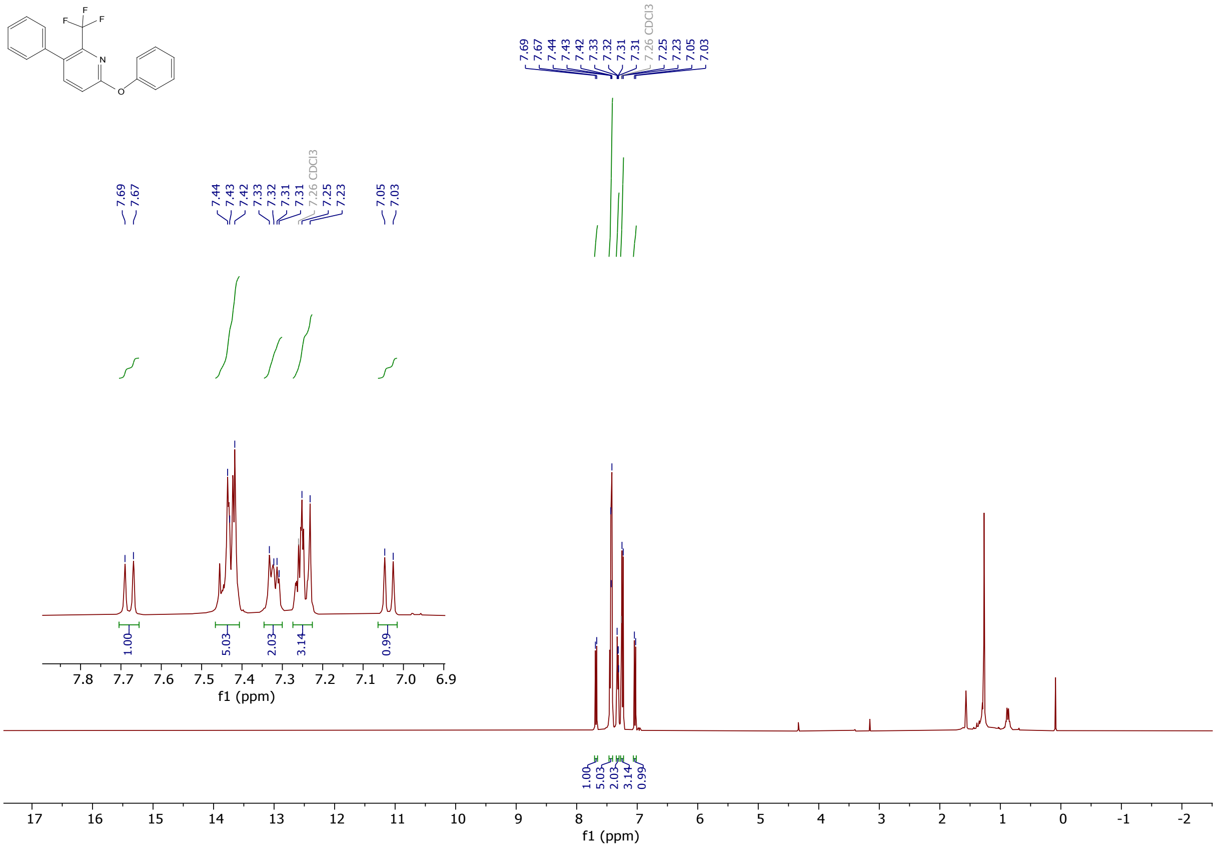

^{13}C NMR spectrum of **19e** ($CDCl_3$, 101 MHz)

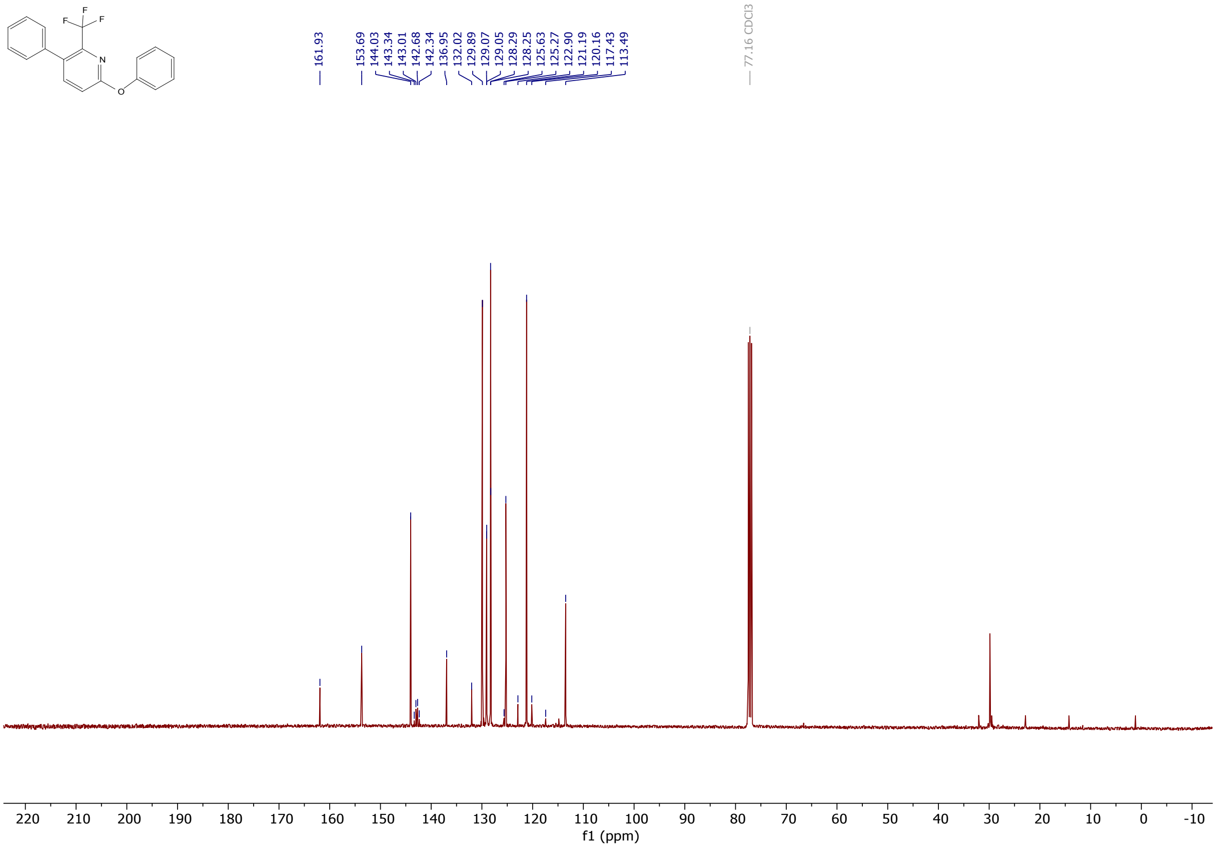

^{19}F NMR spectrum of **19e** ($CDCl_3$, 376 MHz)

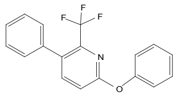

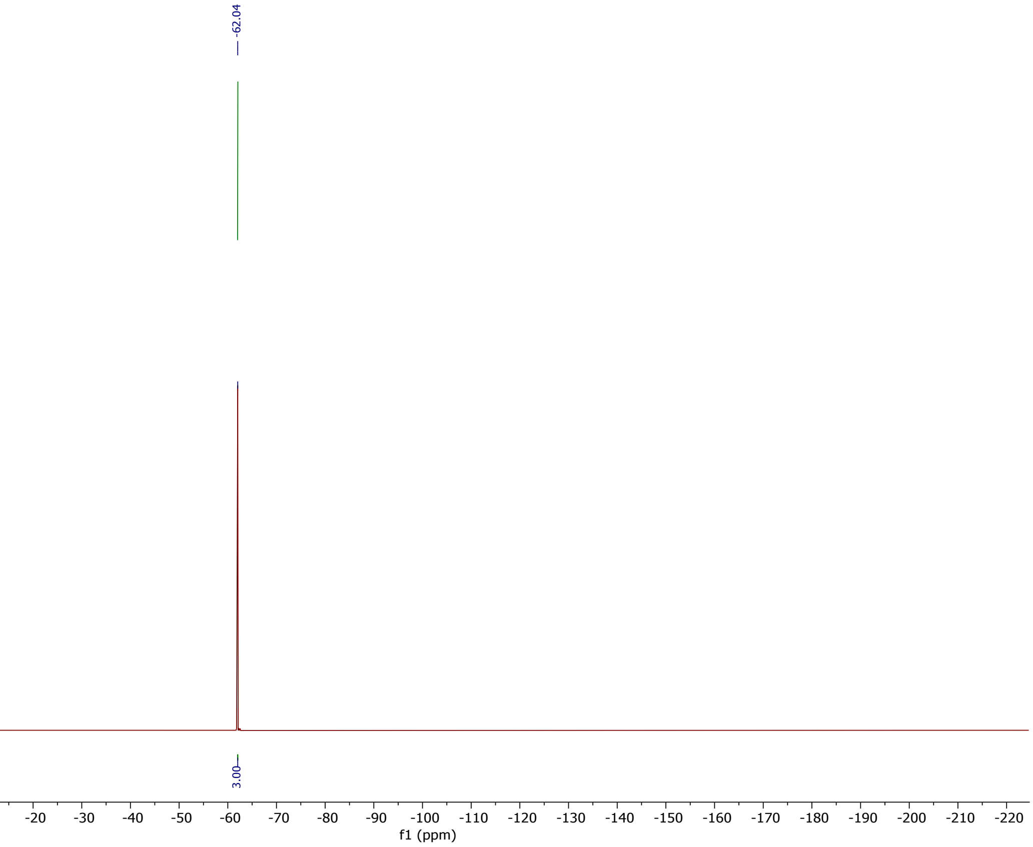

^{1}H NMR spectrum of **19f** ($CDCl_3$, 401 MHz)

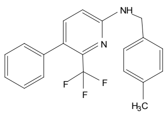

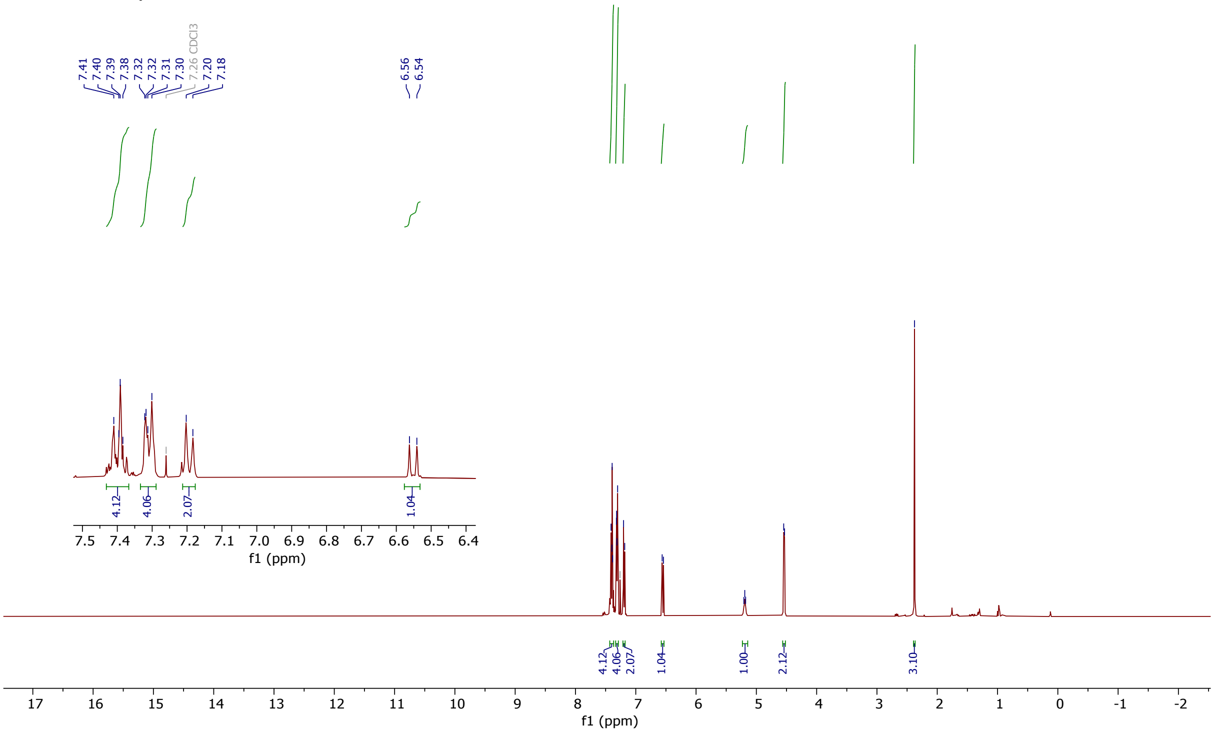

^{13}C NMR spectrum of **19f** (CDCl$_3$, 101 MHz)

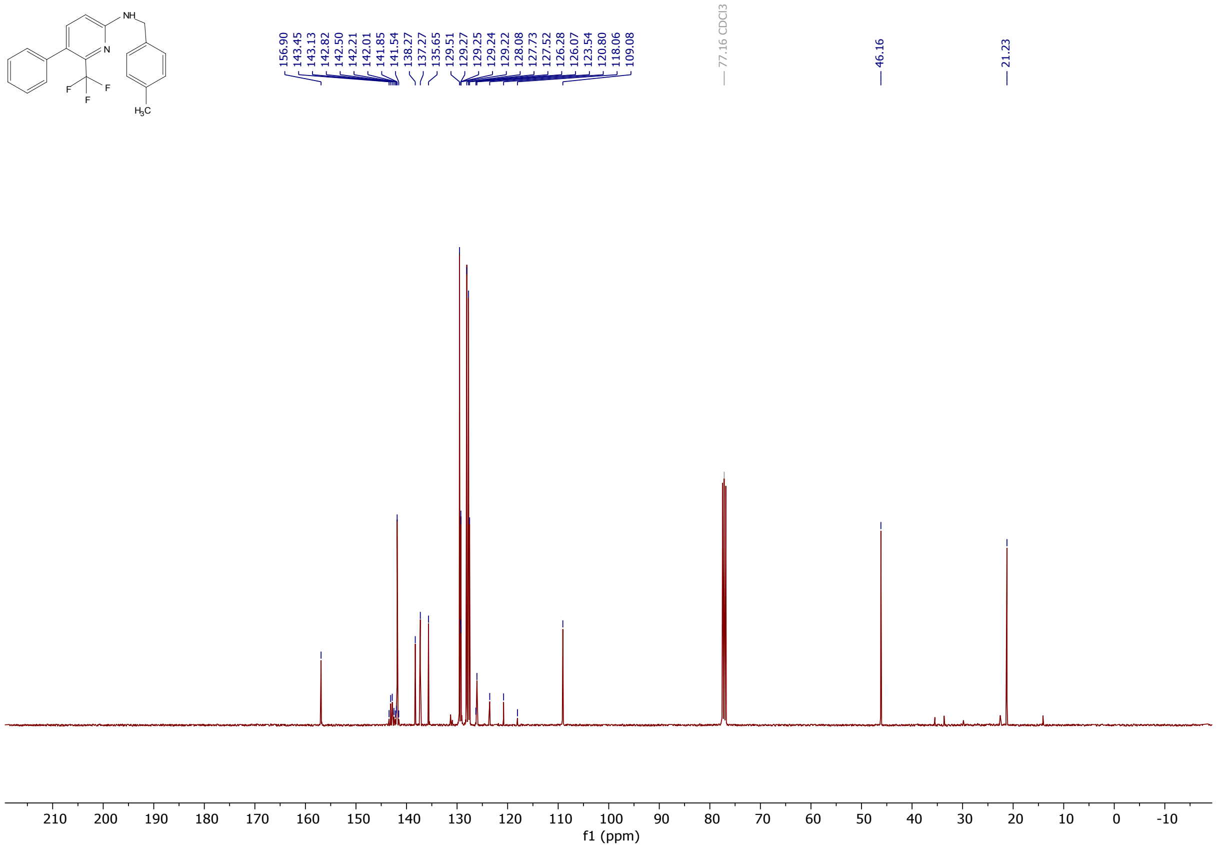

^{19}F NMR spectrum of **19f** ($CDCl_3$, 376 MHz)

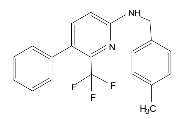

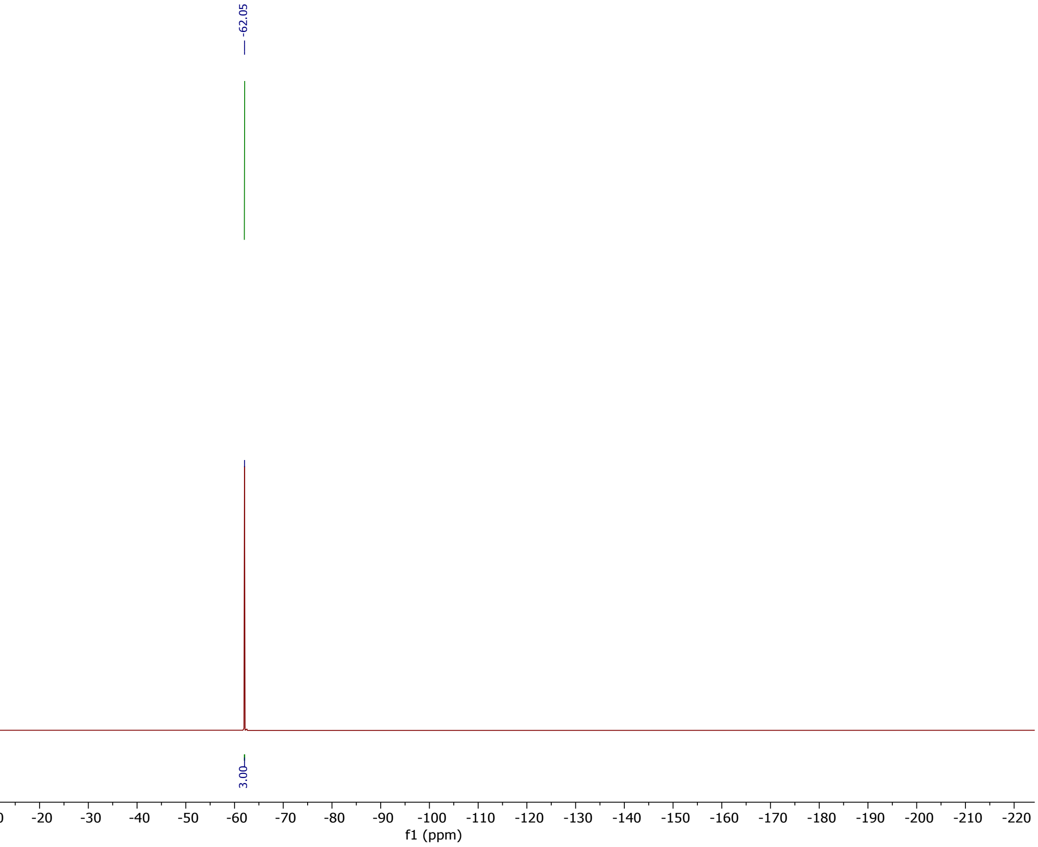

^{1}H NMR spectrum of **19g** ($CDCl_3$, 401 MHz)

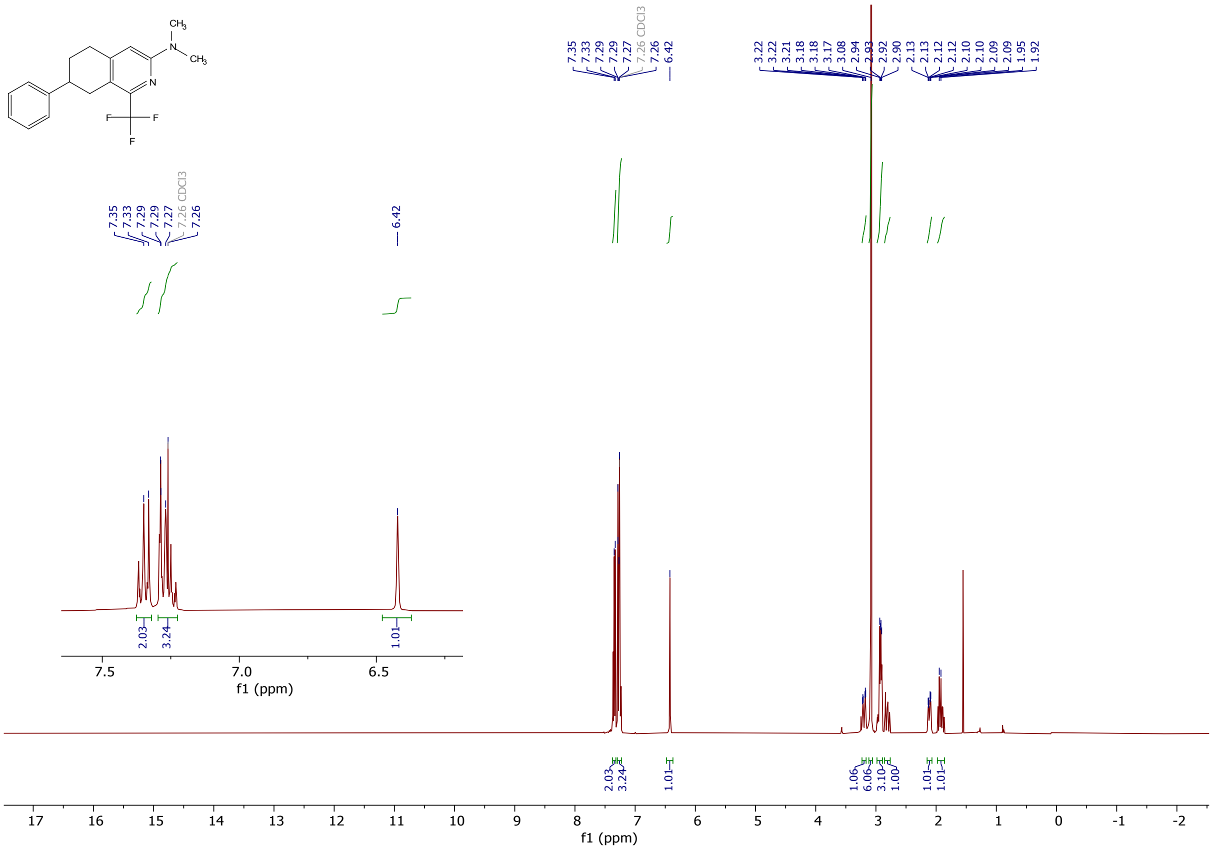

^{13}C NMR spectrum of **19g** ($CDCl_3$, 101 MHz)

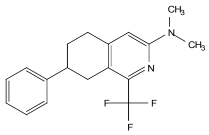

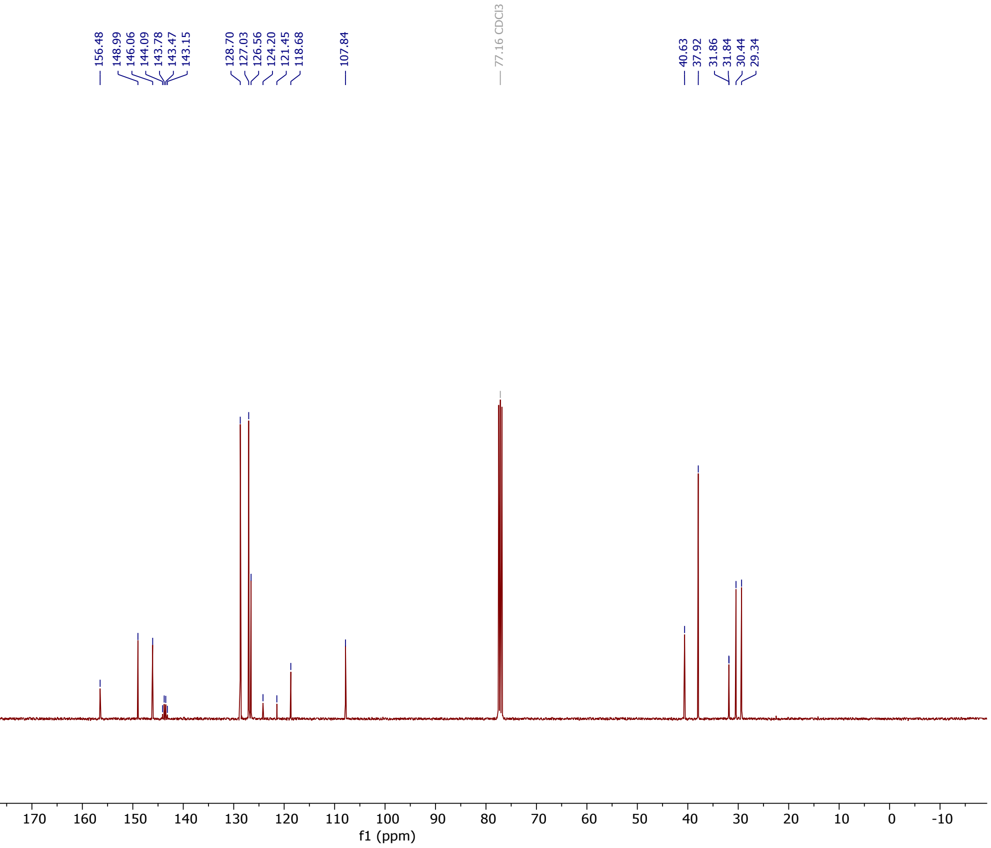

^{19}F NMR spectrum of **19g** ($CDCl_3$, 376 MHz)

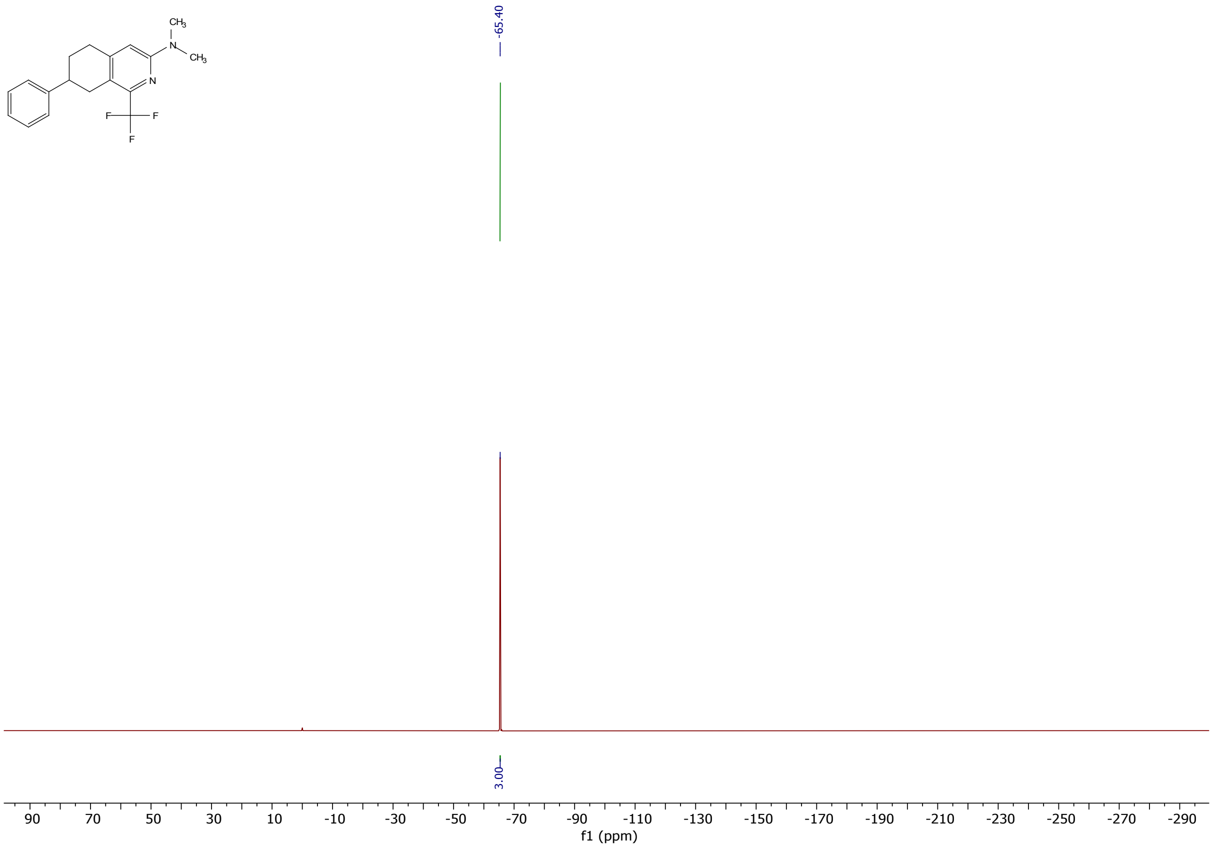

^{1}H NMR spectrum of **19h** ($CDCl_3$, 401 MHz)

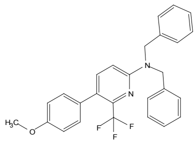

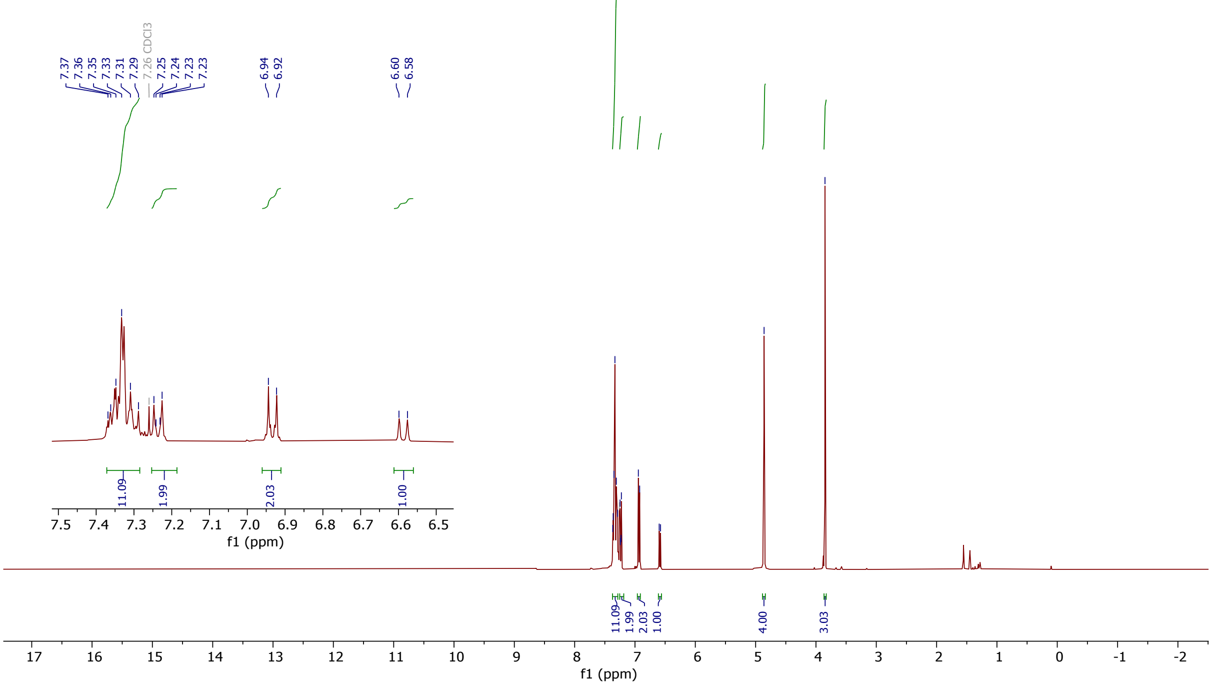

^{13}C NMR spectrum of **19h** ($CDCl_3$, 101 MHz)

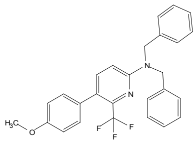

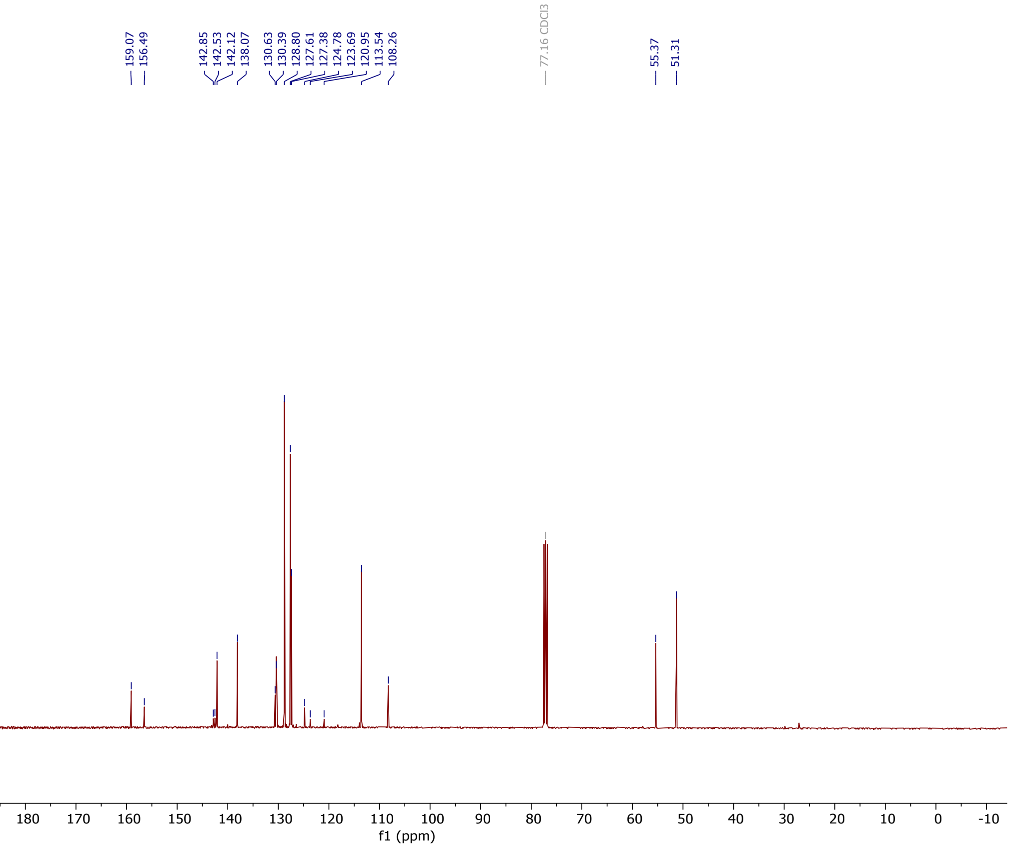

^{19}F NMR spectrum of **19h** ($CDCl_3$, 376 MHz)

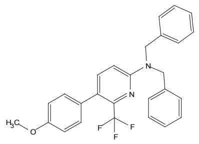

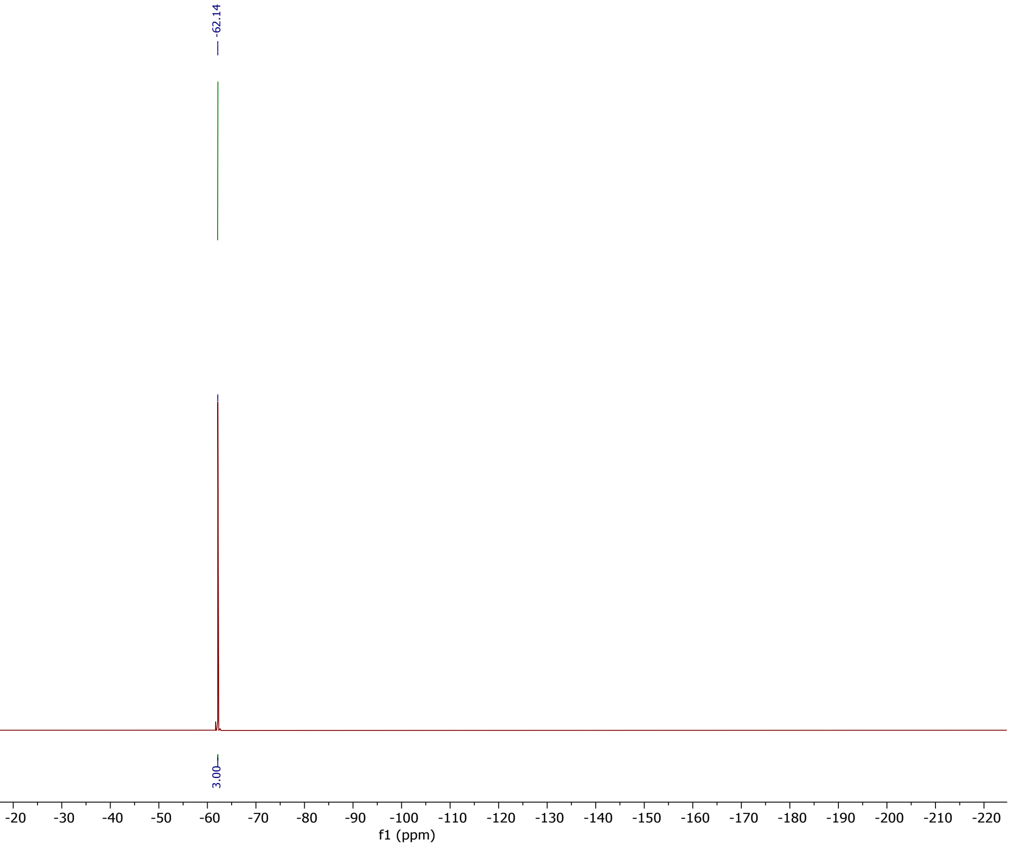

^{1}H NMR spectrum of **19i** ($CDCl_3$, 401 MHz)

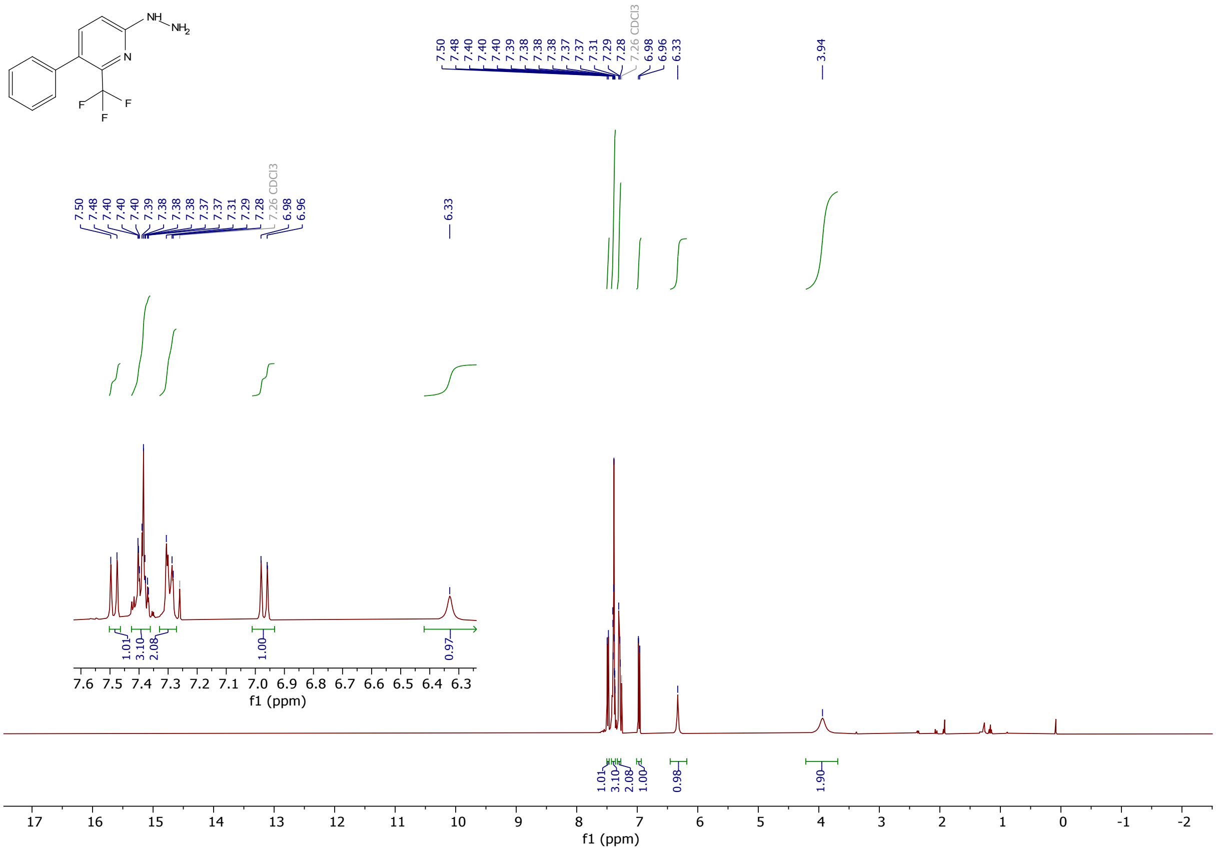

^{13}C NMR spectrum of **19i** ($CDCl_3$, 101 MHz)

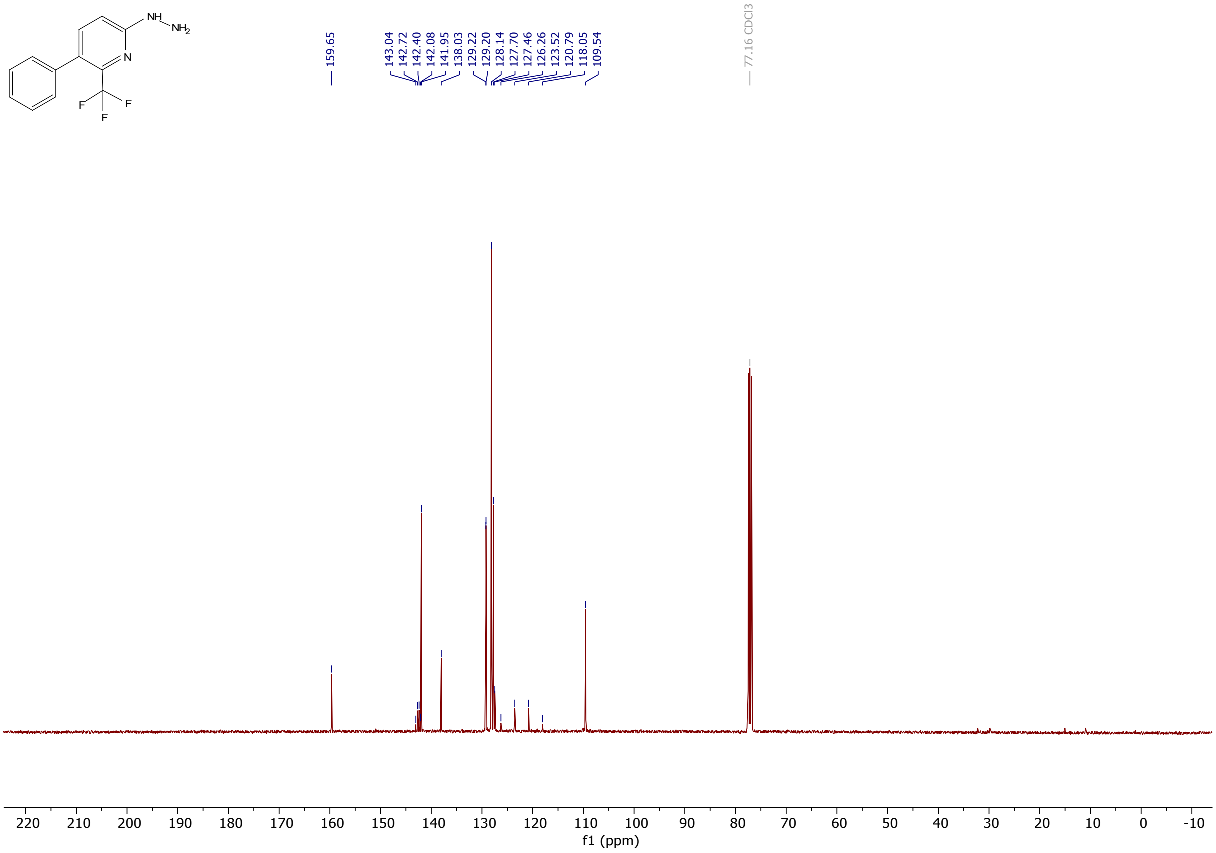

^{19}F NMR spectrum of **19i** ($CDCl_3$, 376 MHz)

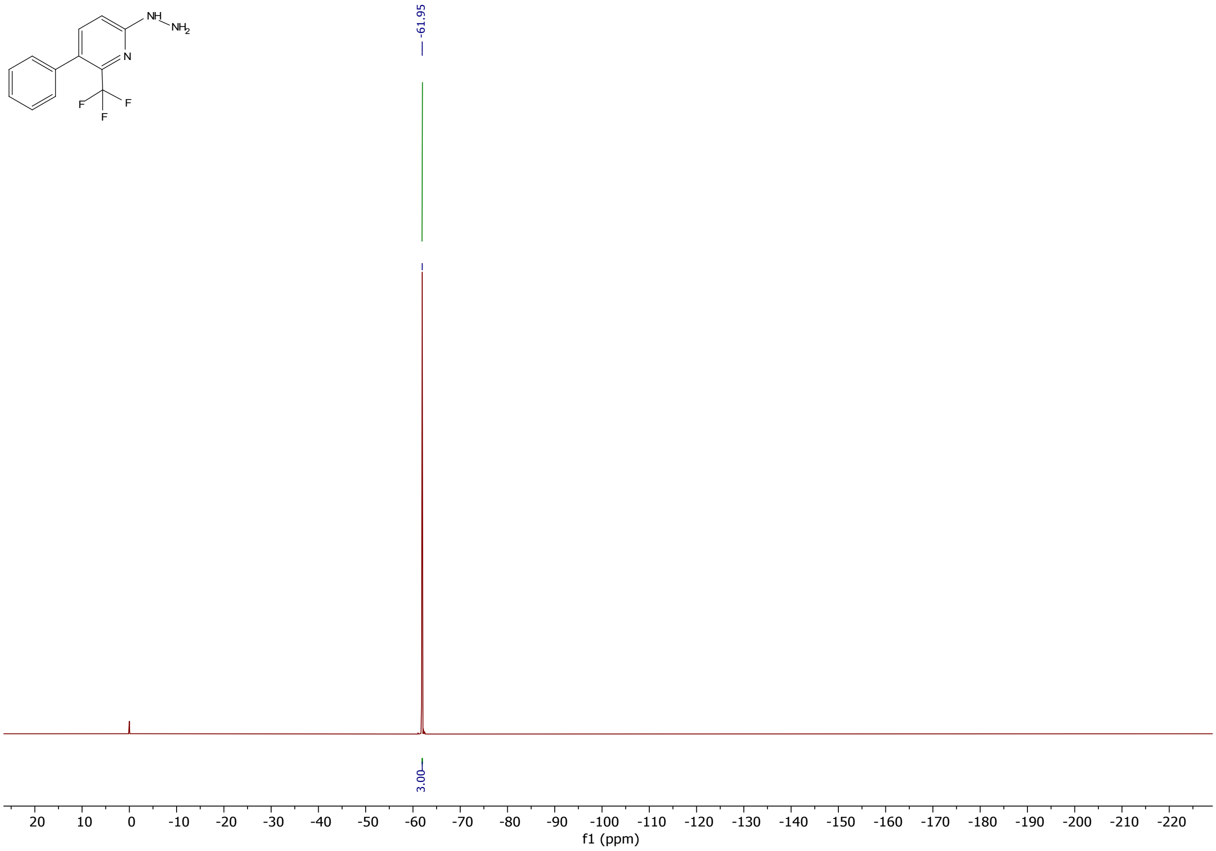

^{1}H NMR spectrum of **19j** ($CDCl_3$, 401 MHz)

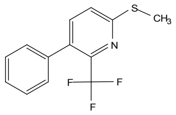

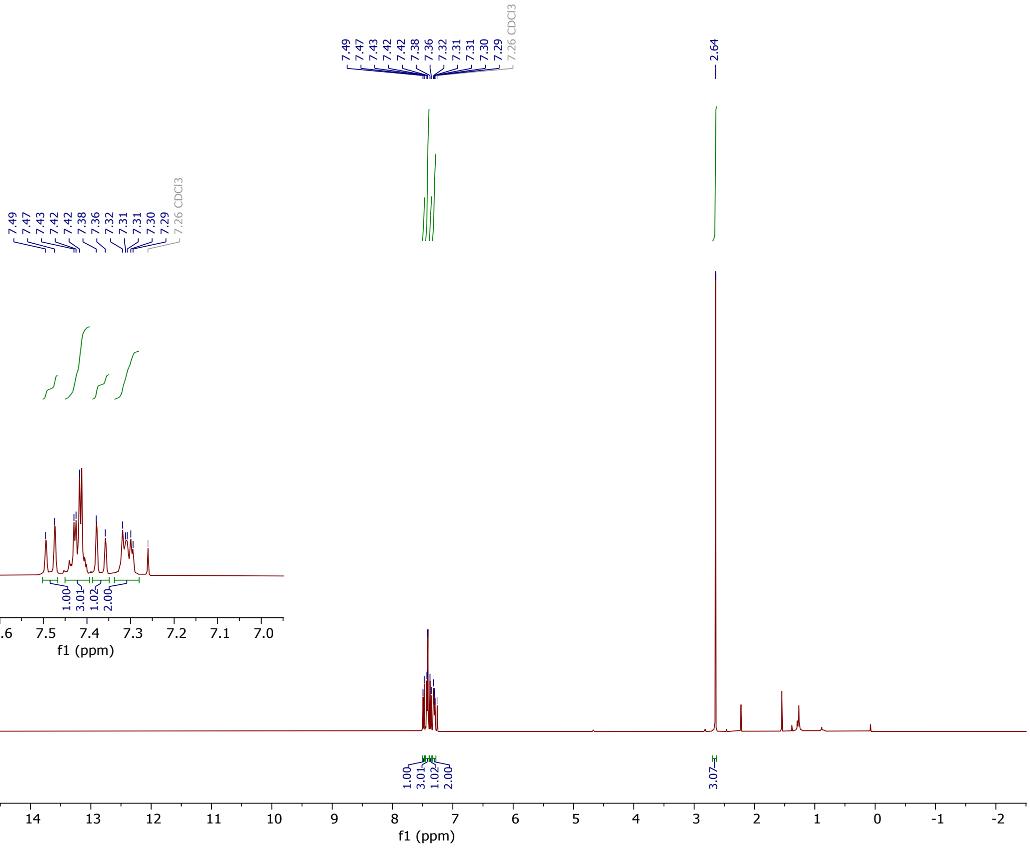

^{13}C NMR spectrum of **19j** ($CDCl_3$, 101 MHz)

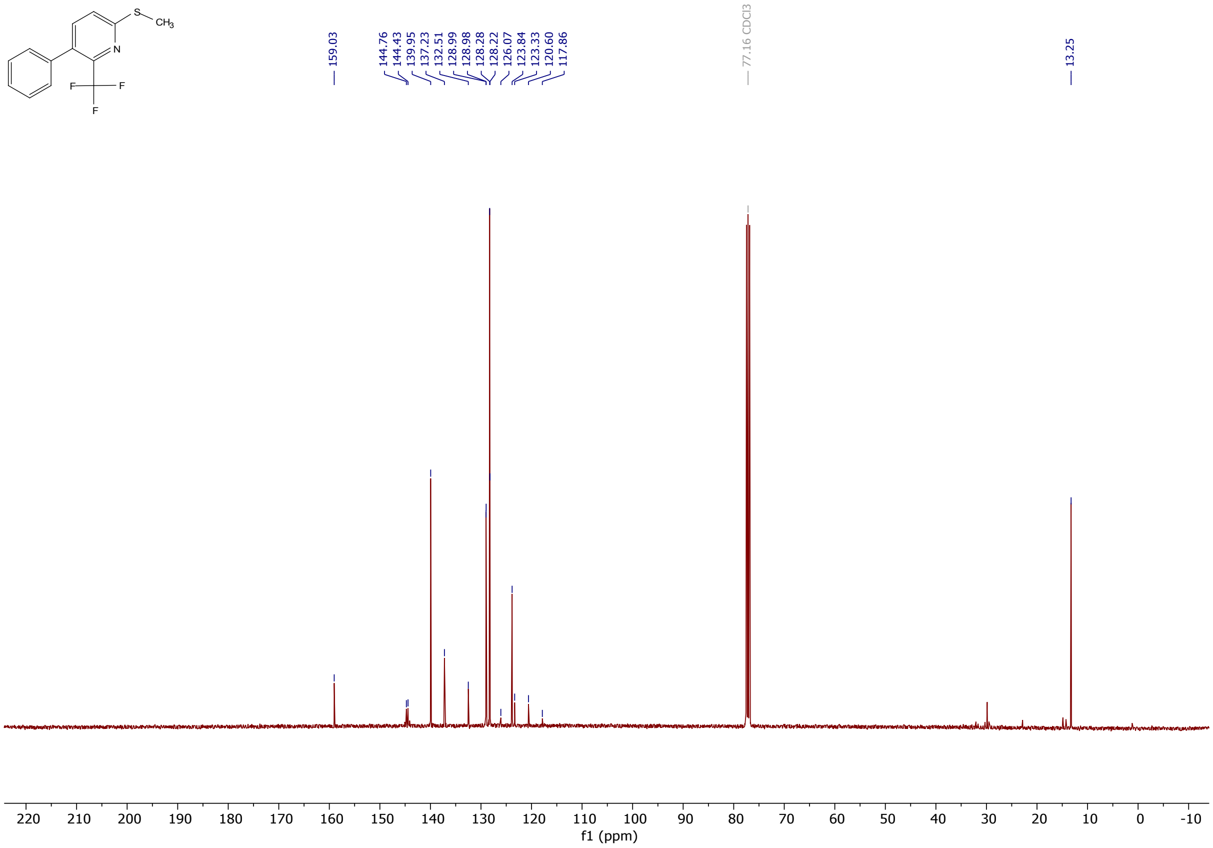

^{19}F NMR spectrum of **19j** ($CDCl_3$, 376 MHz)

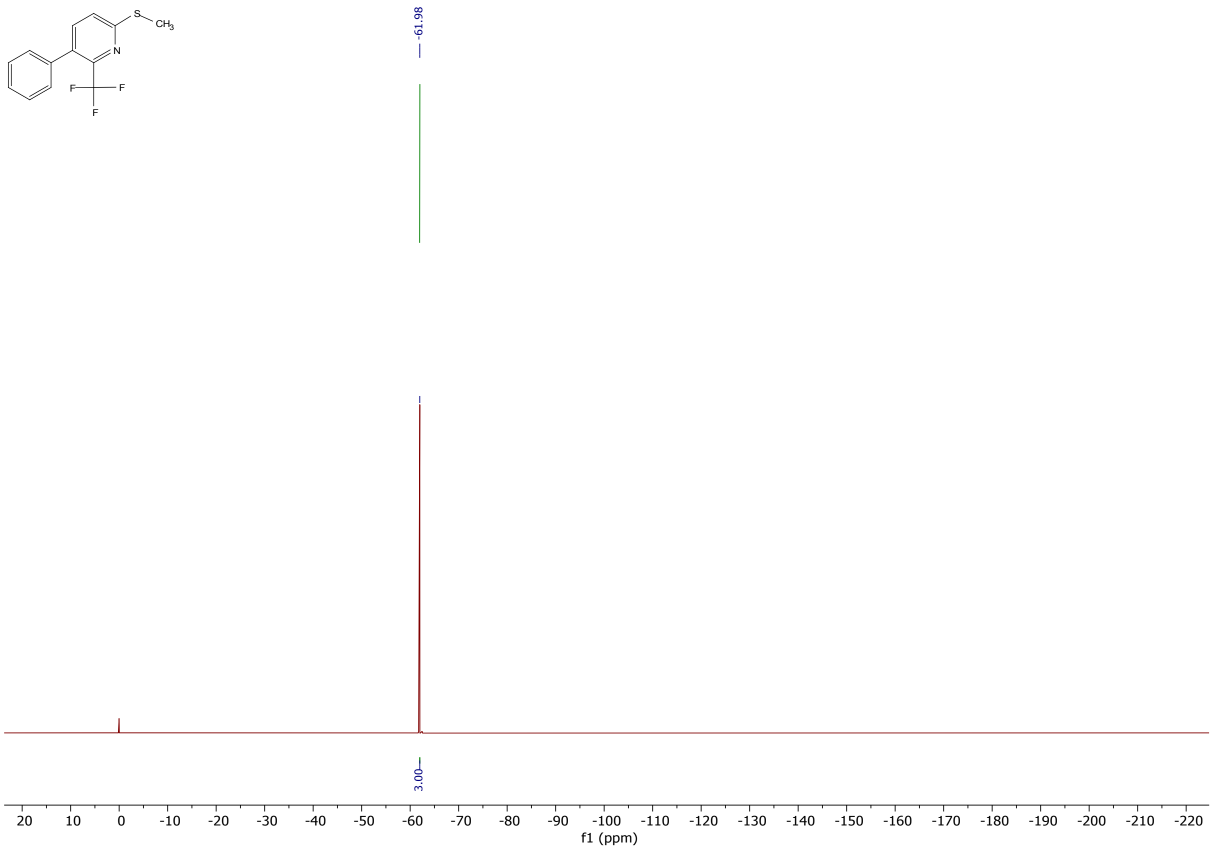

^1H NMR spectrum of **19k** ($CDCl_3$, 401 MHz)

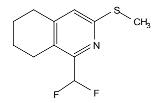

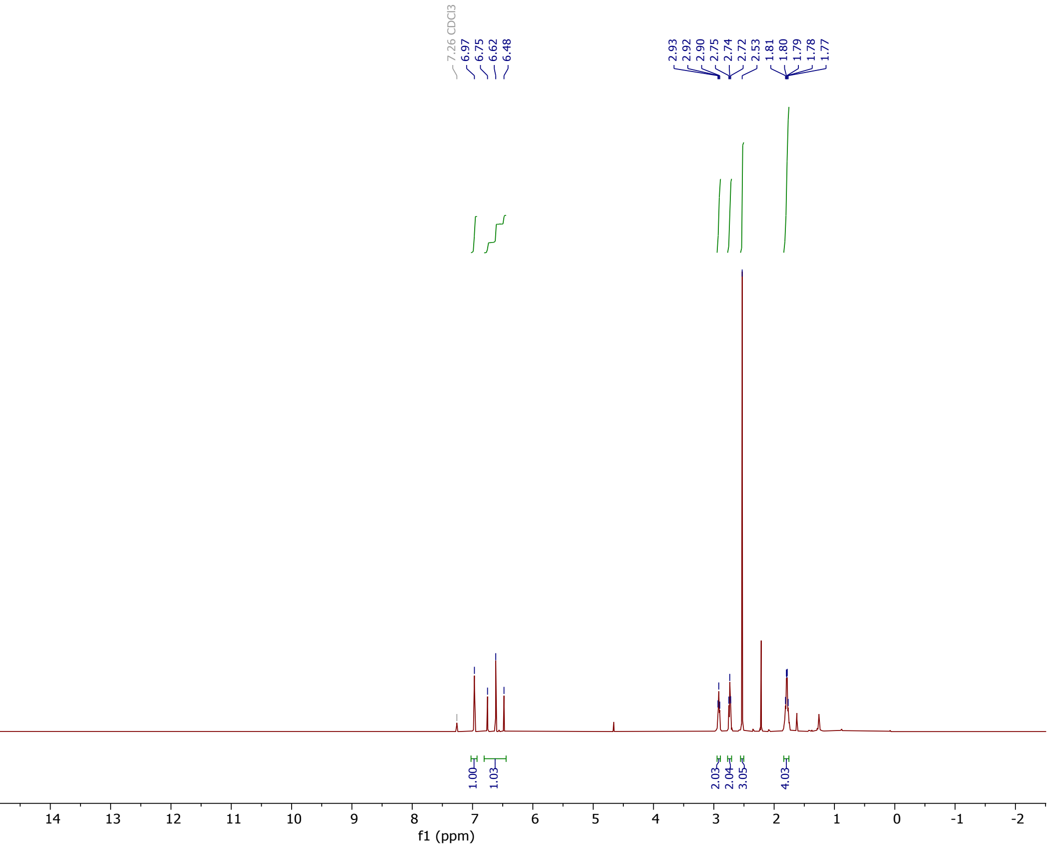

^{13}C NMR spectrum of **19k** ($CDCl_3$, 101 MHz)

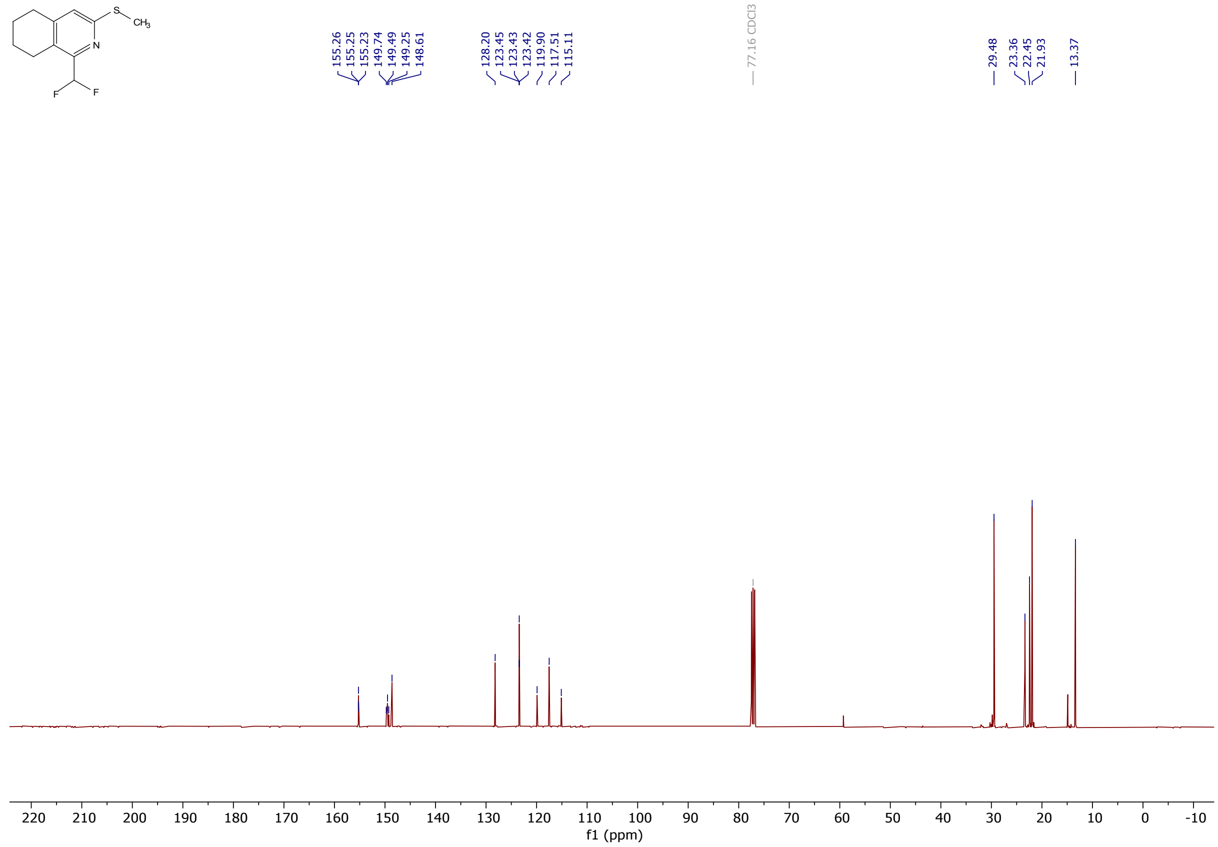

^{19}F NMR spectrum of **19k** ($CDCl_3$, 376 MHz)

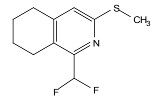

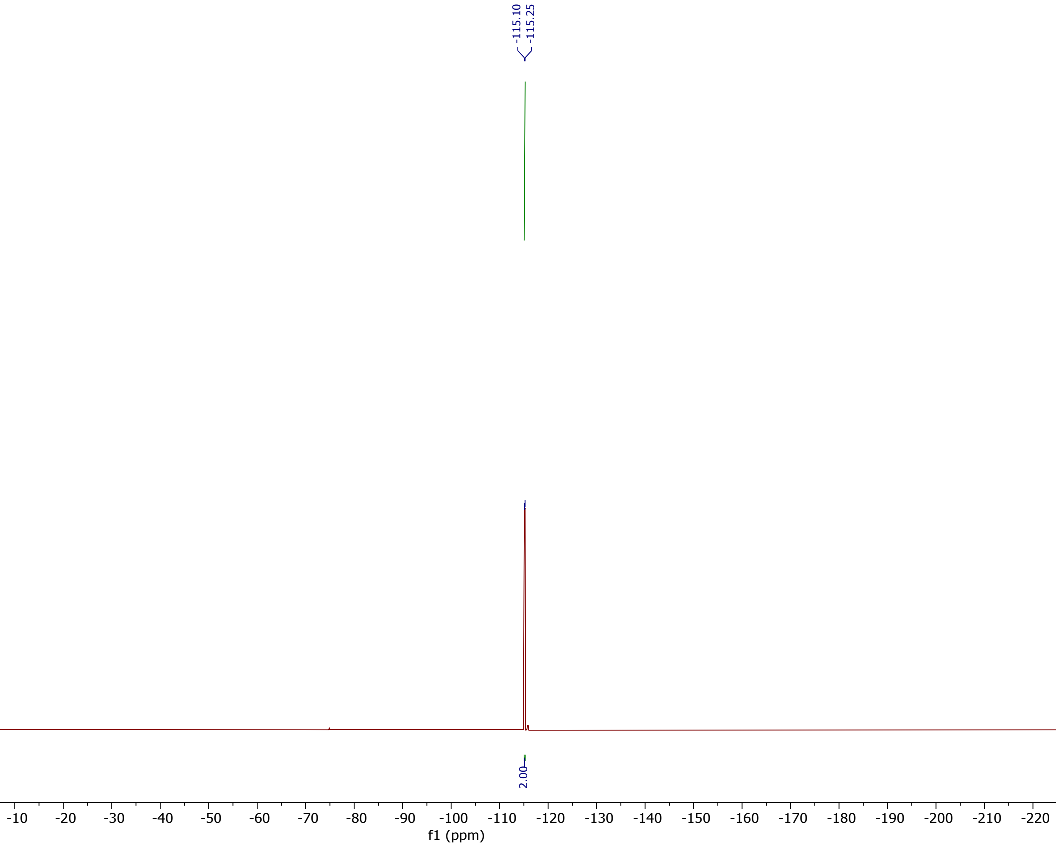

^{1}H NMR spectrum of **19l** ($CDCl_3$, 401 MHz)

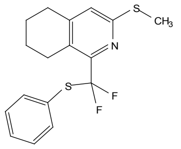

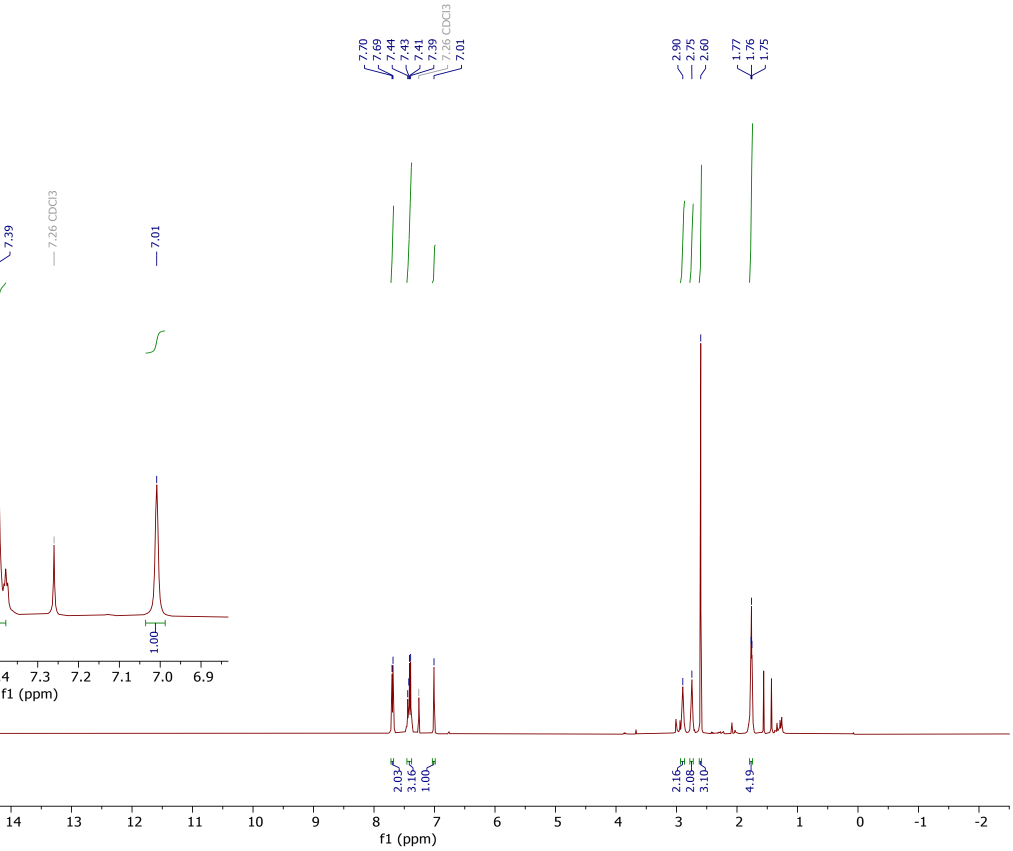

^{13}C NMR spectrum of **19l** ($CDCl_3$, 101 MHz)

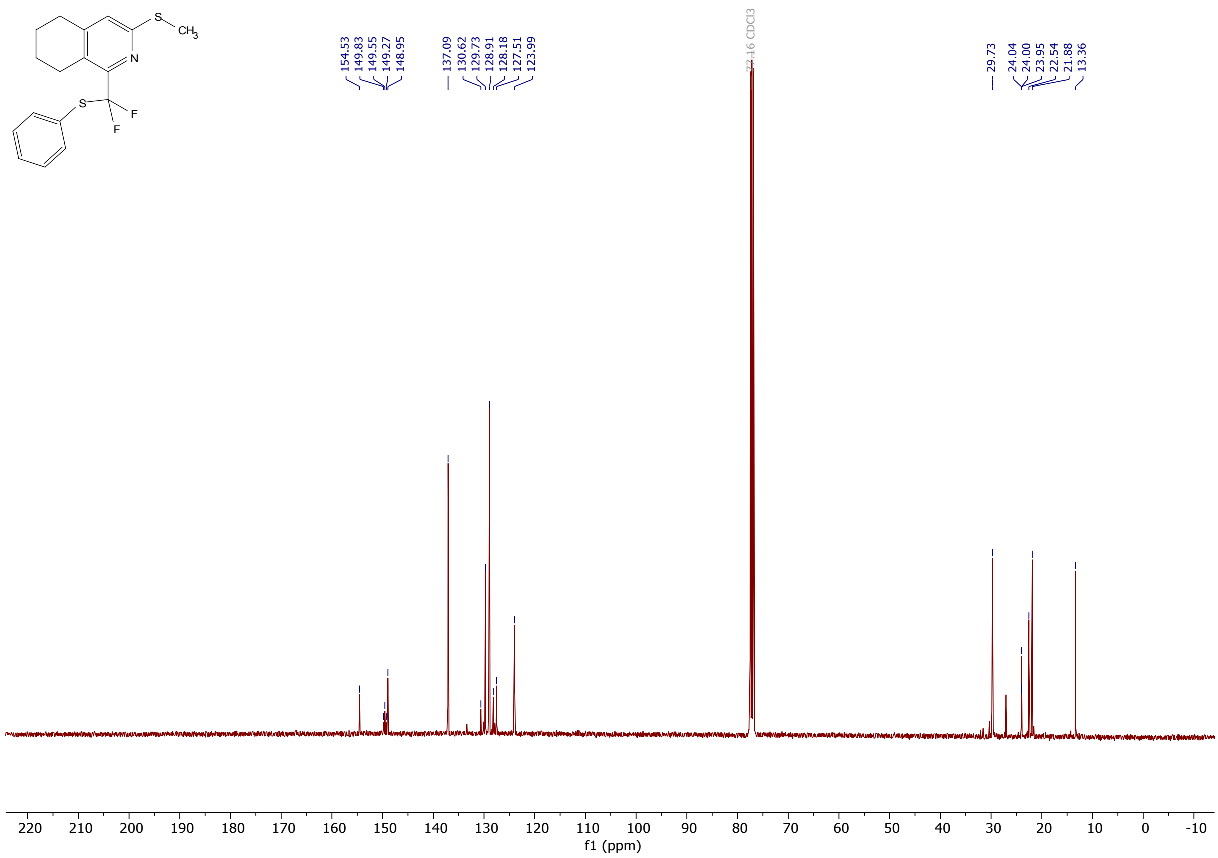

^{19}F NMR spectrum of **19l** ($CDCl_3$, 376 MHz)

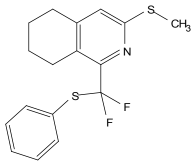

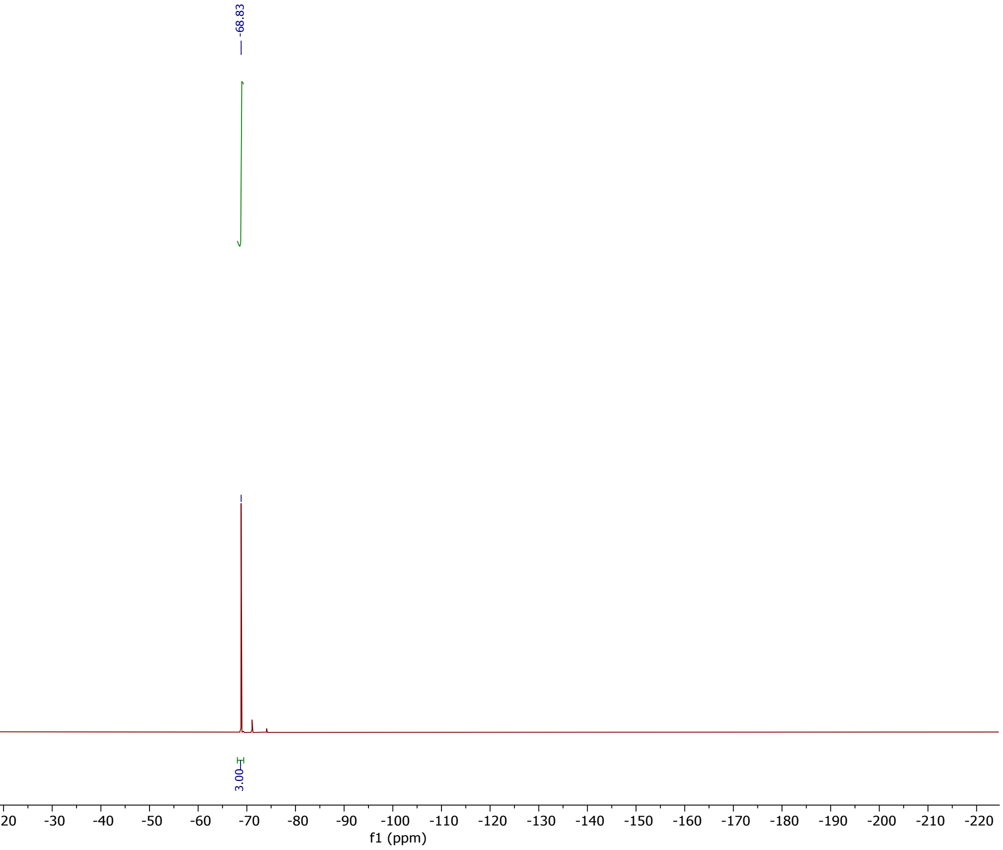

^{1}H NMR spectrum of **19m** ($CDCl_3$, 401 MHz)

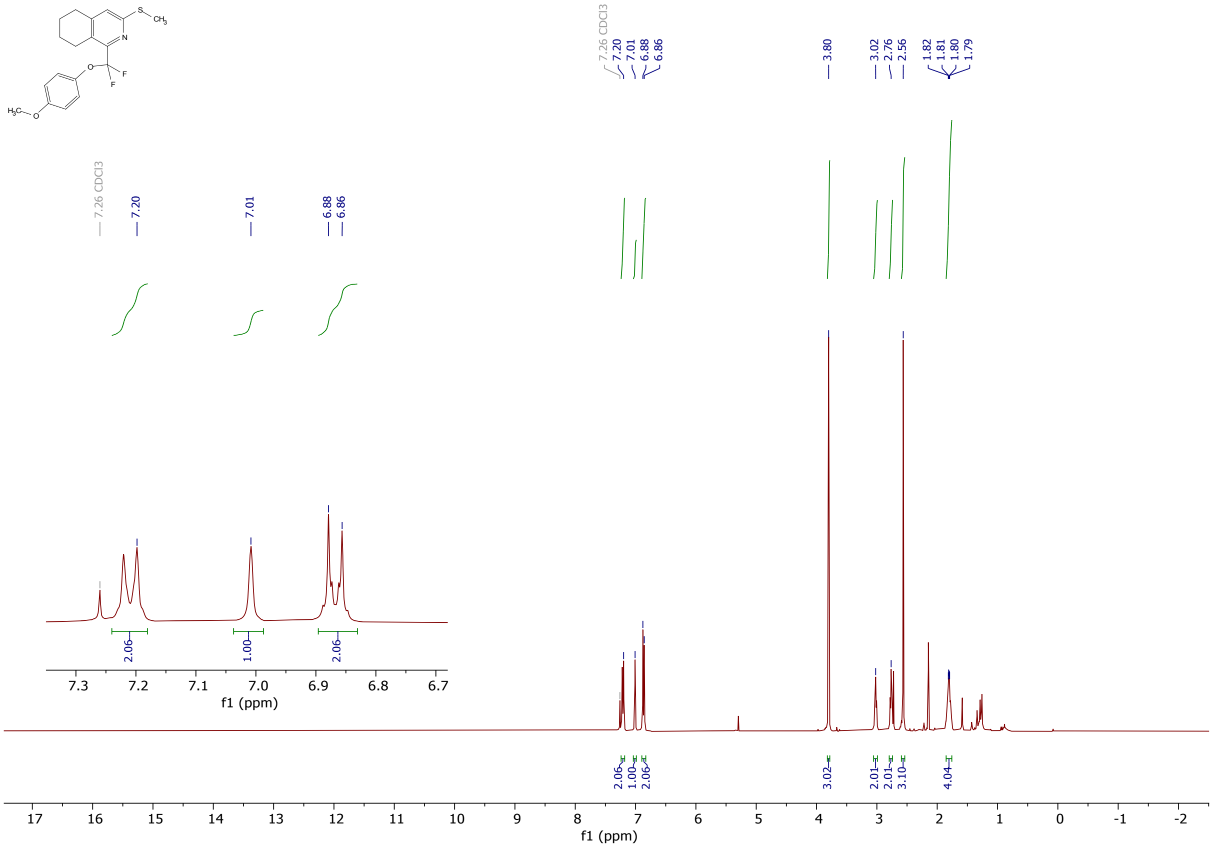

^{13}C NMR spectrum of **19m** ($CDCl_3$, 101 MHz)

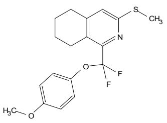

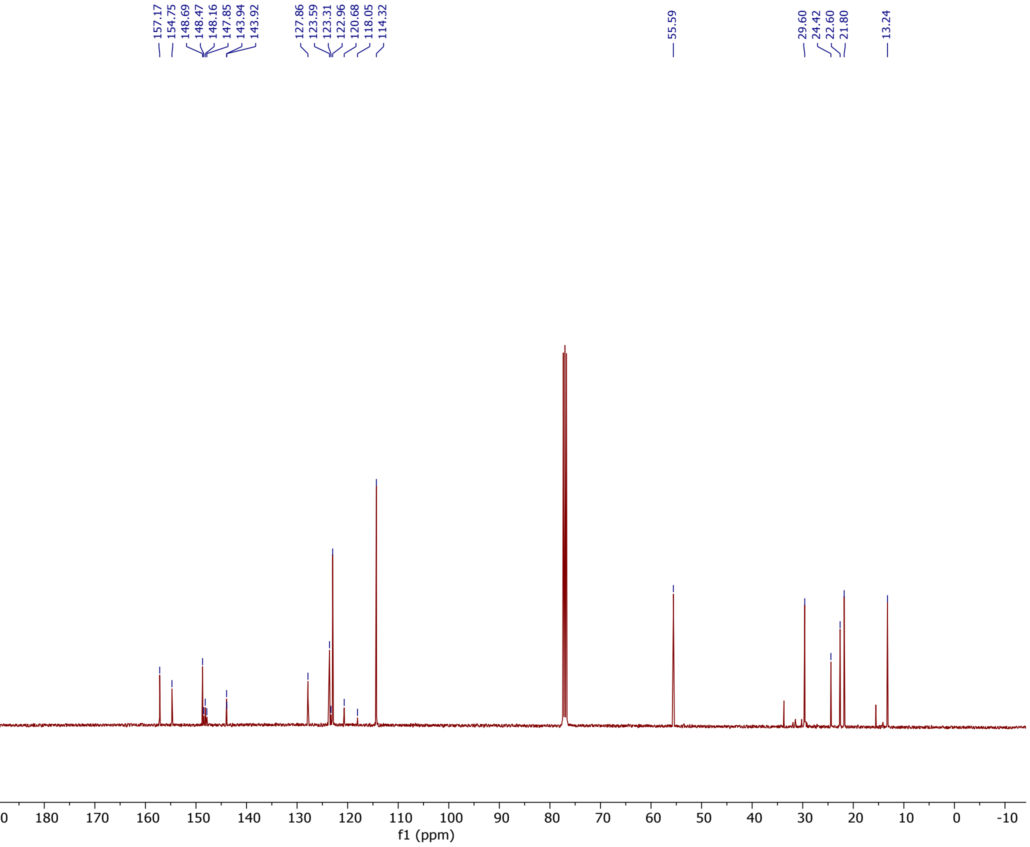

^{19}F NMR spectrum of **19m** ($CDCl_3$, 376 MHz)

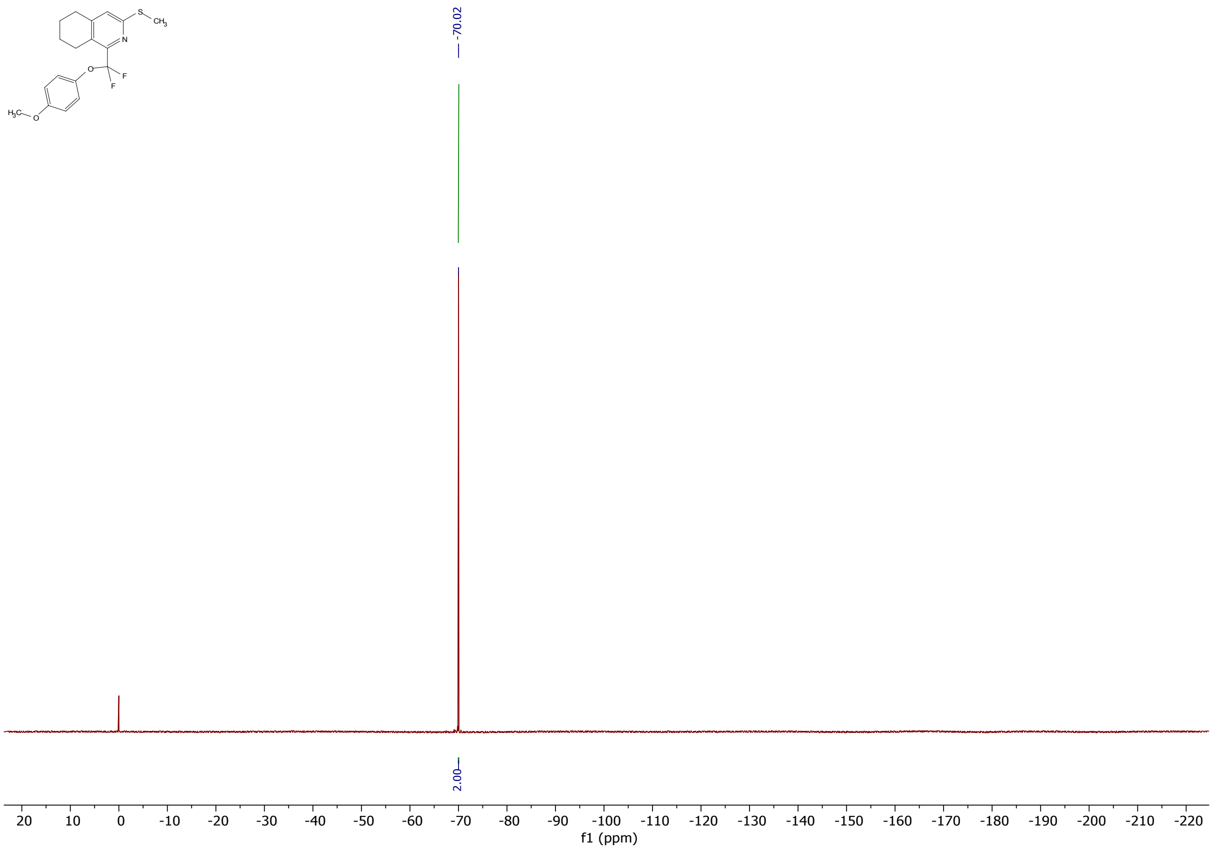

^{1}H NMR spectrum of **19n** ($CDCl_3$, 401 MHz)

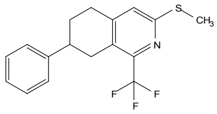

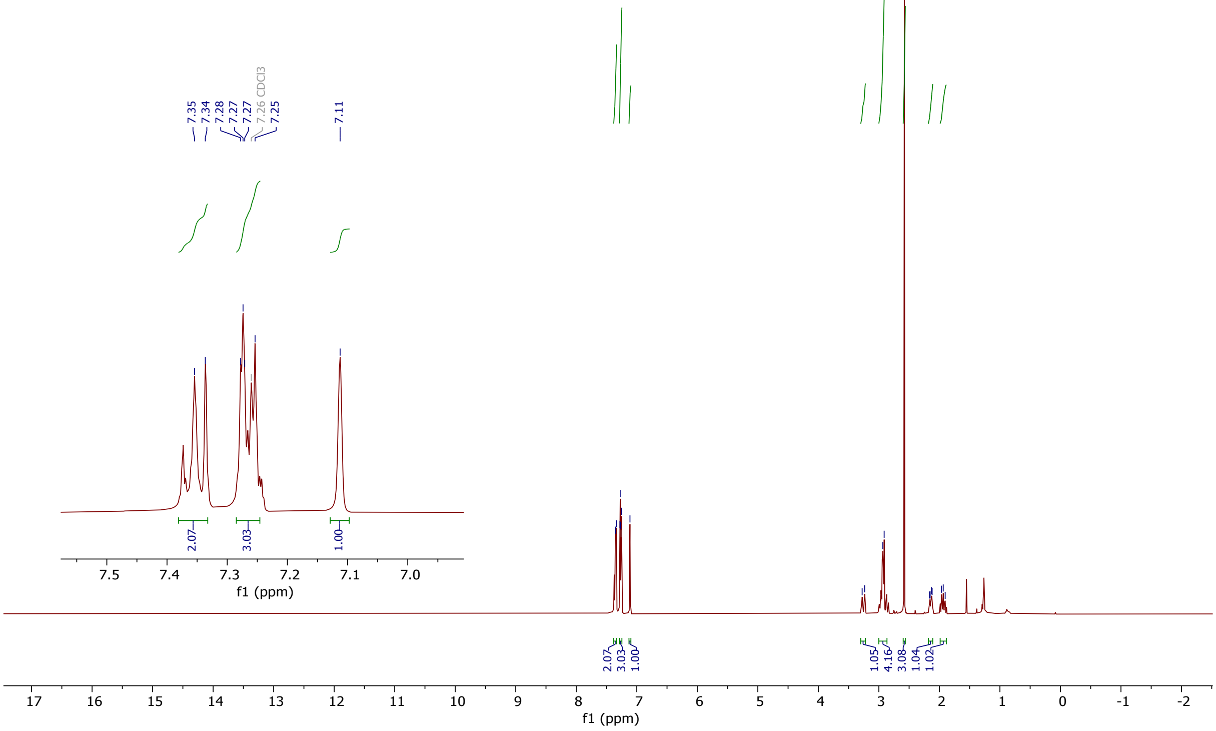

^{13}C NMR spectrum of **19n** ($CDCl_3$, 101 MHz)

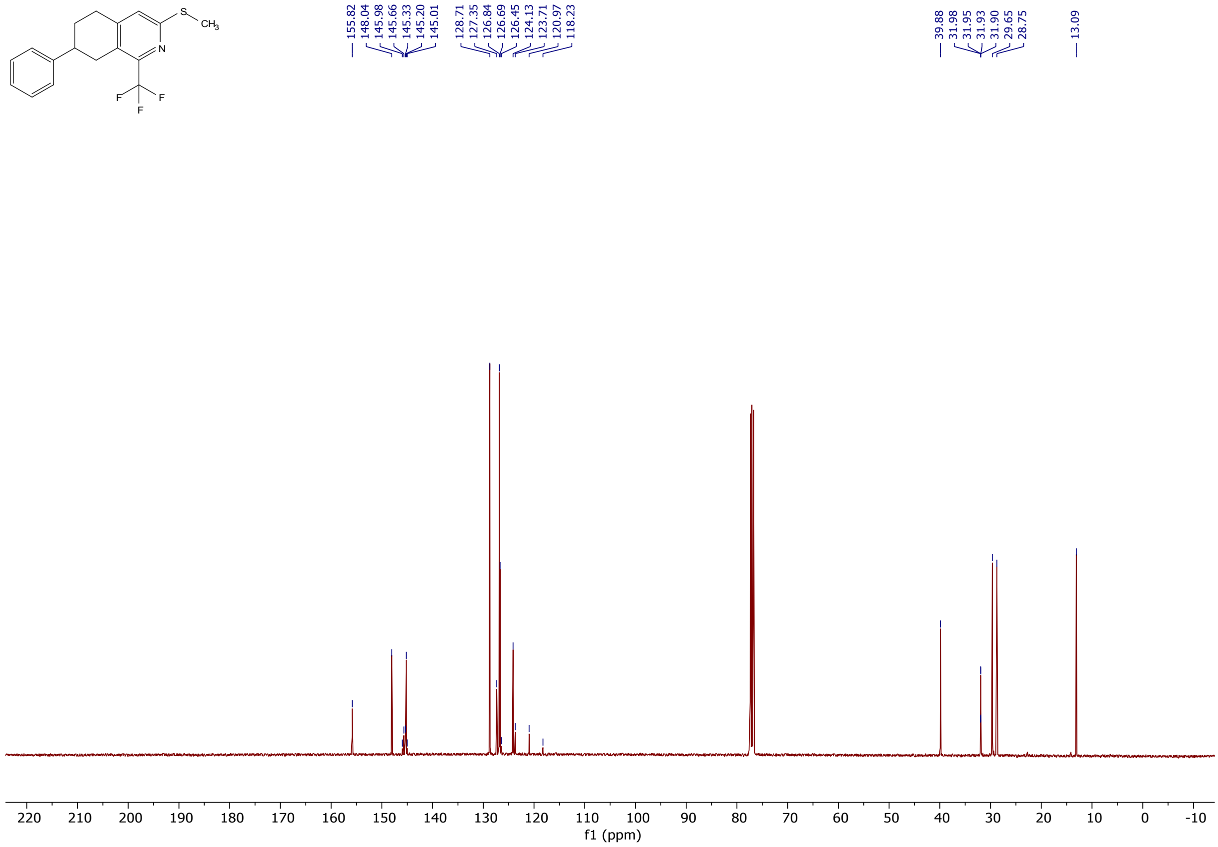

^{19}F NMR spectrum of **19n** ($CDCl_3$, 376 MHz)

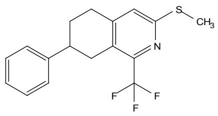

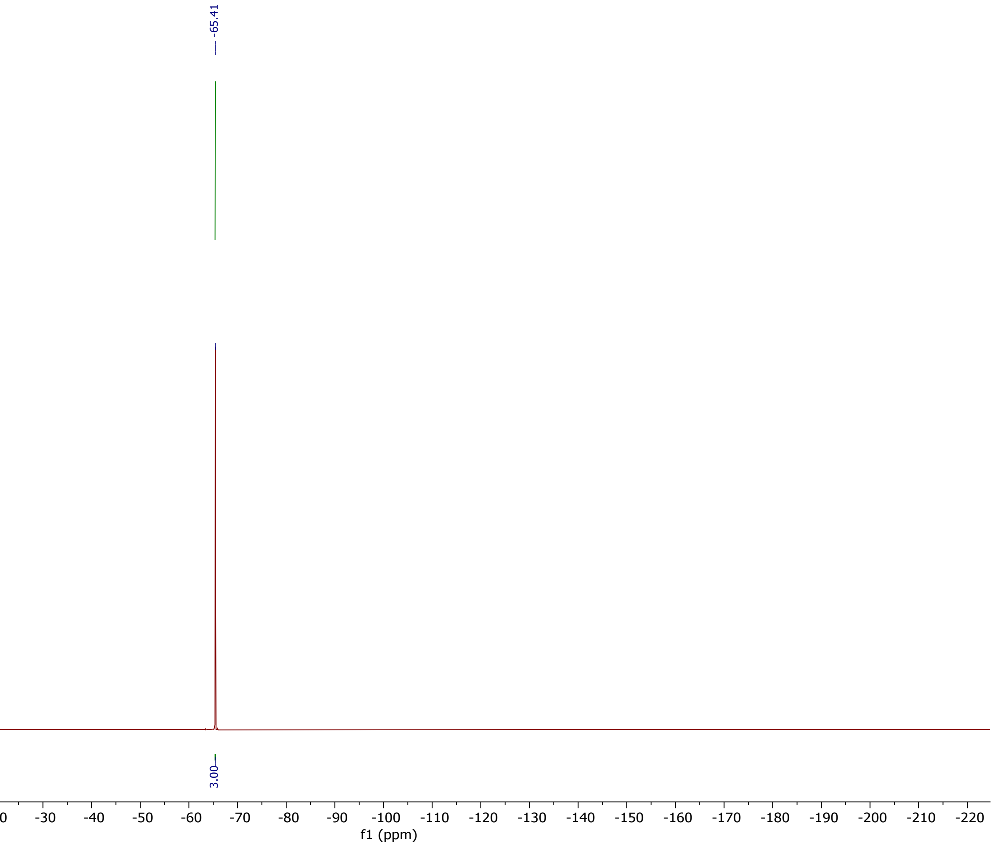

^{1}H NMR spectrum of **19o** ($CDCl_3$, 401 MHz)

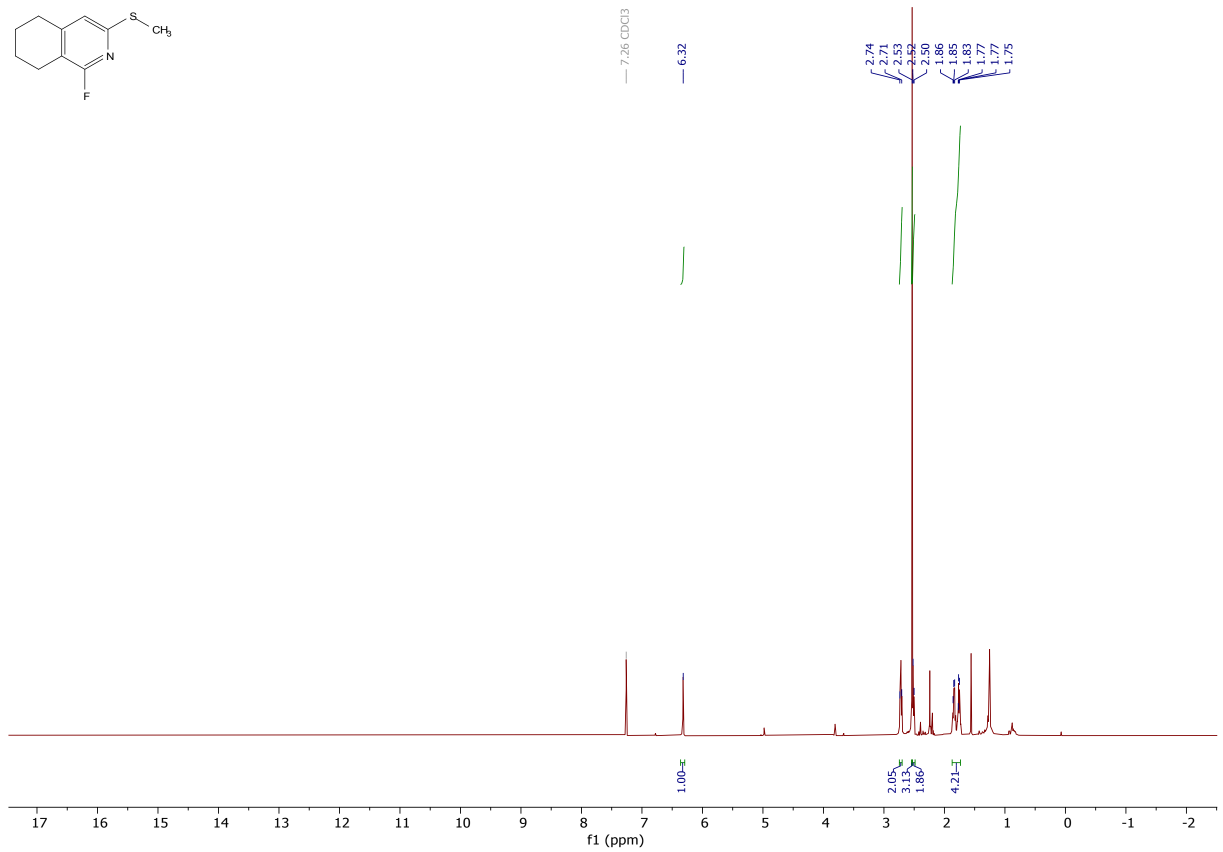

^{13}C NMR spectrum of **19o** ($CDCl_3$, 101 MHz)

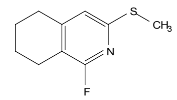

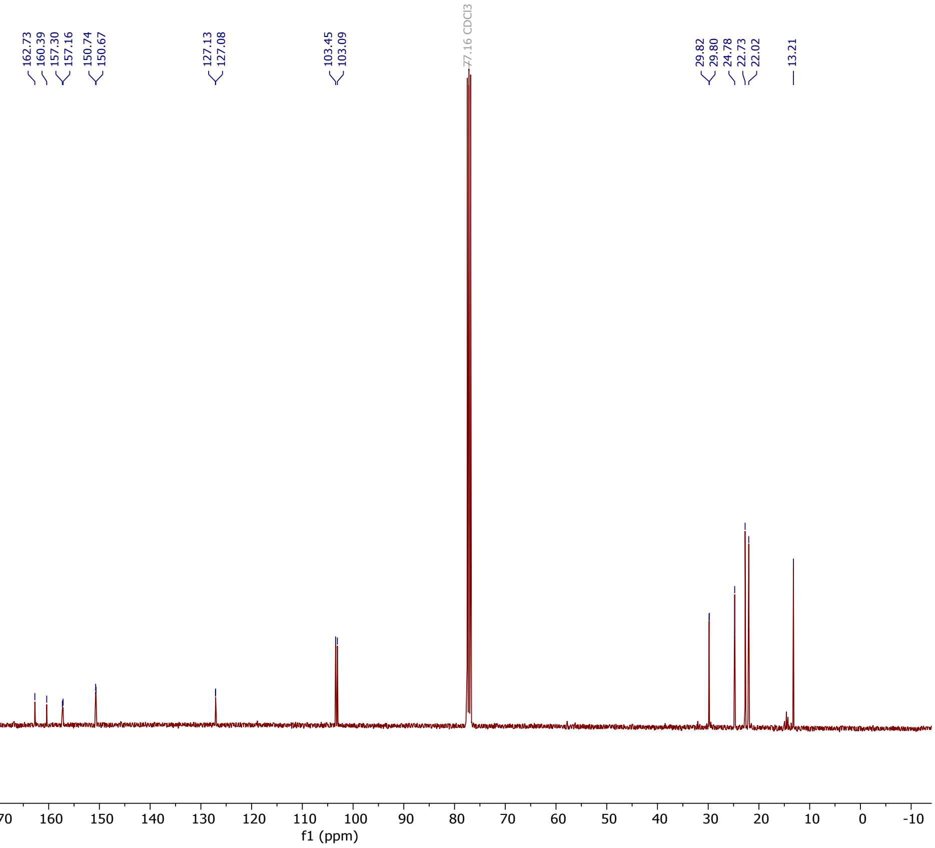

^{19}F NMR spectrum of **19o** ($CDCl_3$, 376 MHz)

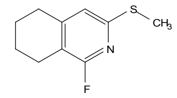

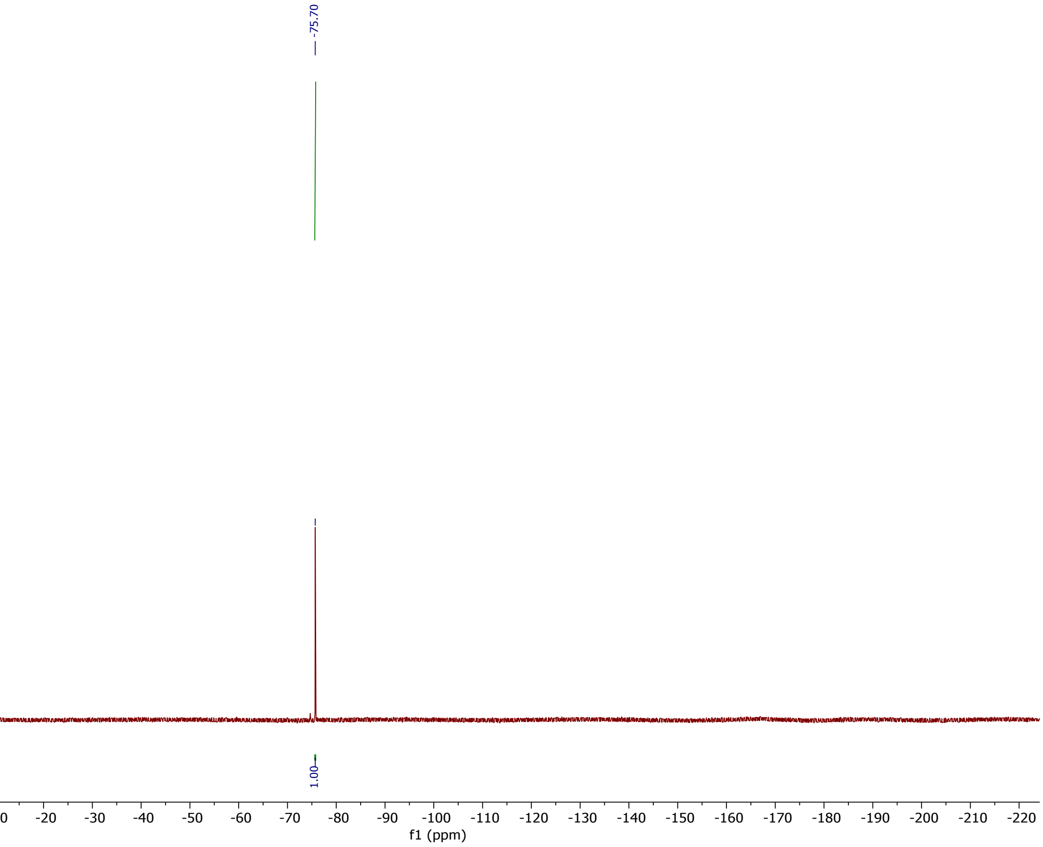

^{1}H NMR spectrum of **19p** ($CDCl_3$, 401 MHz)

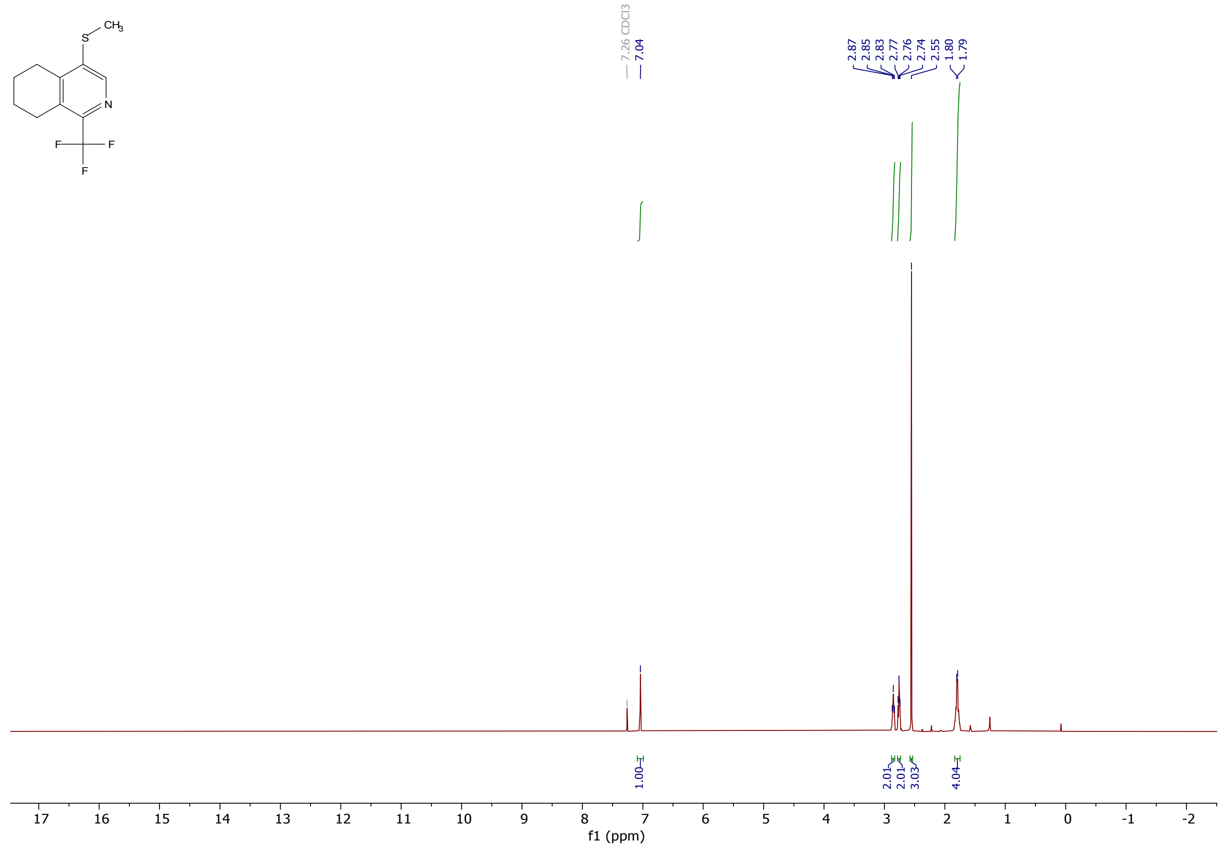

^{13}C NMR spectrum of **19p** ($CDCl_3$, 101 MHz)

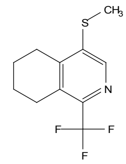

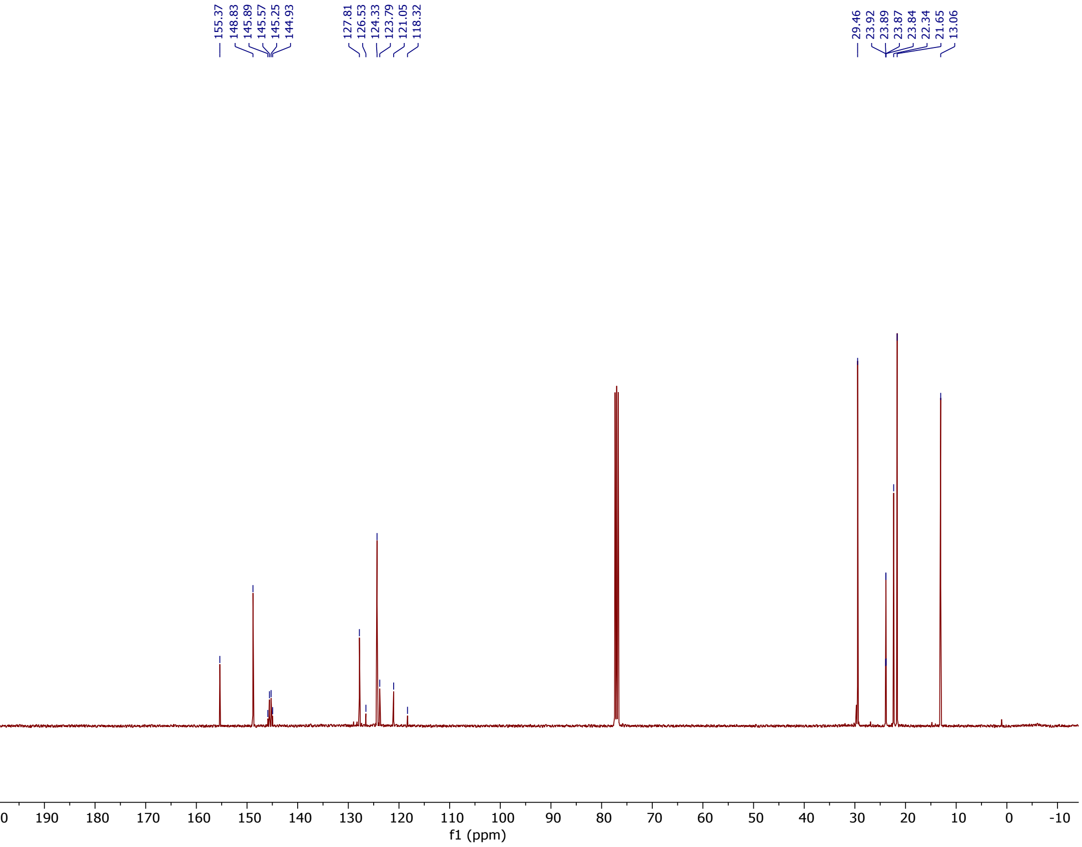

^{19}F NMR spectrum of **19p** ($CDCl_3$, 376 MHz)

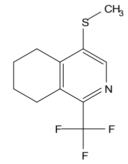

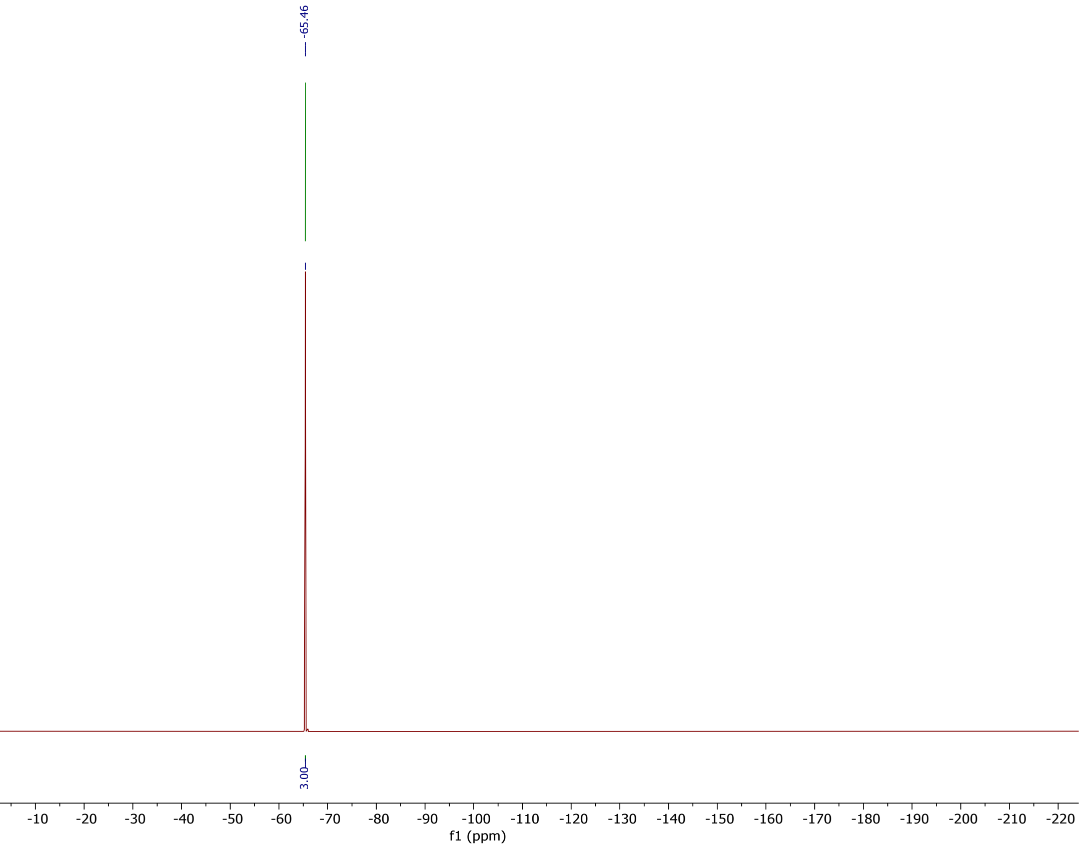

10 X-ray crystallography plots

Thermal ellipsoid plot (50% probability) of the compound **11m**

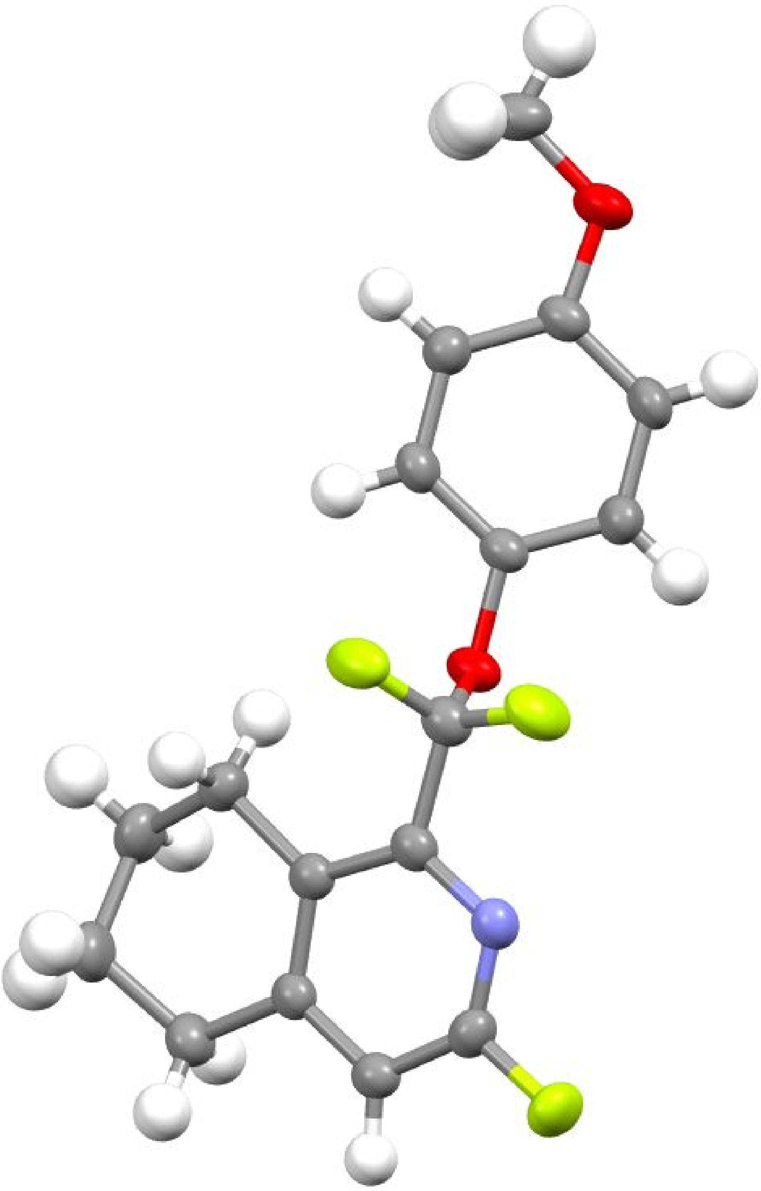

Thermal ellipsoid plot (50% probability) of the compound **18f**

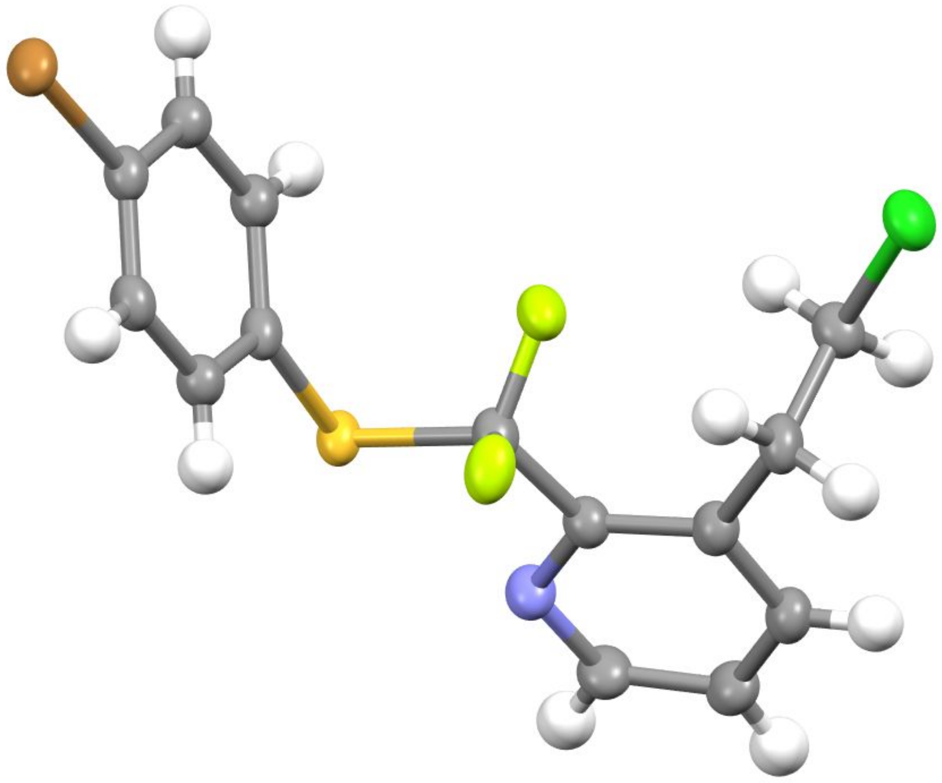

Thermal ellipsoid plot (50% probability) of the compound **19g**

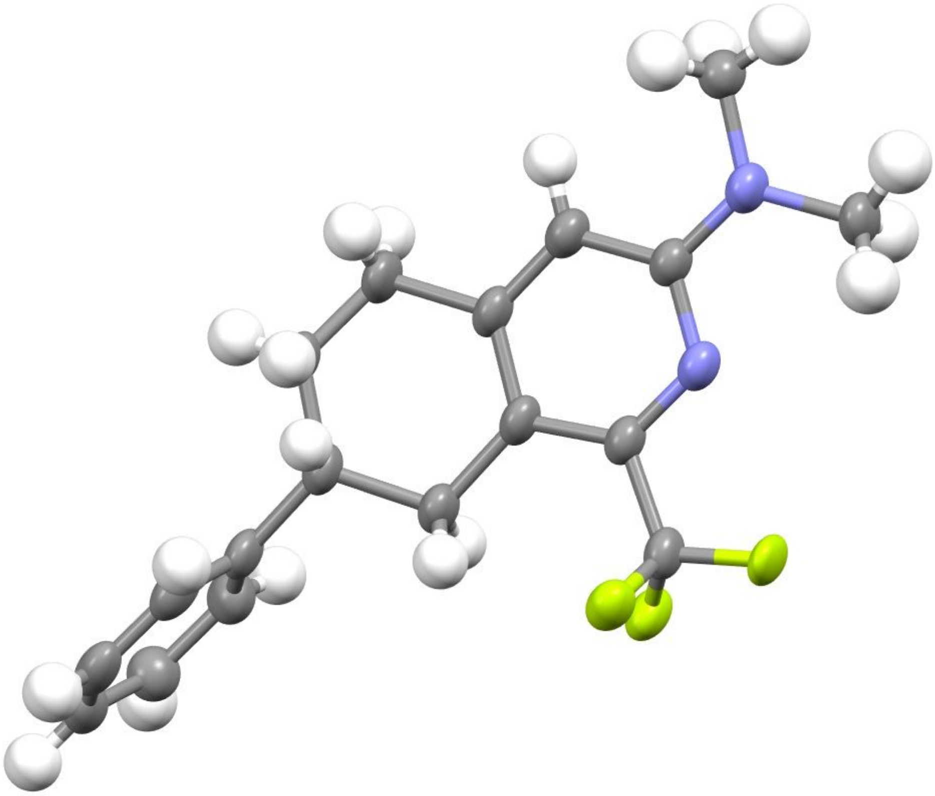

Supplement: Supplementary file 1 [file jo5c03046_si_001.pdf]
